# Evolution of Acetylcholinesterase and Butyrylcholinesterase in the Vertebrates: An Atypical Butyrylcholinesterase from the Medaka *Oryzias latipes*

**DOI:** 10.1371/journal.pone.0017396

**Published:** 2011-02-25

**Authors:** Leo Pezzementi, Florian Nachon, Arnaud Chatonnet

**Affiliations:** 1 Department of Biology, Birmingham-Southern College, Birmingham, Alabama, United States of America; 2 Département de Toxicologie, Institut de Recherche Biomédicale des Armées, Antenne de la Tronche, La Tronche, France; 3 Institut National de la Recherche Agronomique, Unité Mixte de Recherche 866, Montpellier, France; 4 Université Montpellier 1, Montpellier, France; 5 Université Montpellier 2, Montpellier, France; Biological Research Center of the Hungarian Academy of Sciences, Hungary

## Abstract

Acetylcholinesterase (AChE) and butyrylcholinesterase (BChE) are thought to be the result of a gene duplication event early in vertebrate evolution. To learn more about the evolution of these enzymes, we expressed *in vitro*, characterized, and modeled a recombinant cholinesterase (ChE) from a teleost, the medaka *Oryzias latipes*. In addition to AChE, *O. latipes* has a ChE that is different from either vertebrate AChE or BChE, which we are classifying as an atypical BChE, and which may resemble a transitional form between the two. Of the fourteen aromatic amino acids in the catalytic gorge of vertebrate AChE, ten are conserved in the atypical BChE of *O. latipes*; by contrast, only eight are conserved in vertebrate BChE. Notably, the atypical BChE has one phenylalanine in its acyl pocket, while AChE has two and BChE none. These substitutions could account for the intermediate nature of this atypical BChE. Molecular modeling supports this proposal. The atypical BChE hydrolyzes acetylthiocholine (ATCh) and propionylthiocholine (PTCh) preferentially but butyrylthiocholine (BTCh) to a considerable extent, which is different from the substrate specificity of AChE or BChE. The enzyme shows substrate inhibition with the two smaller substrates but not with the larger substrate BTCh. In comparison, AChE exhibits substrate inhibition, while BChE does not, but may instead show substrate activation. The atypical BChE from *O. latipes* also shows a mixed pattern of inhibition. It is effectively inhibited by physostigmine, typical of all ChEs. However, although the atypical BChE is efficiently inhibited by the BChE-specific inhibitor ethopropazine, it is not by another BChE inhibitor, iso-OMPA, nor by the AChE-specific inhibitor BW284c51. The atypical BChE is found as a glycophosphatidylinositol-anchored (GPI-anchored) amphiphilic dimer (G_2_^a^), which is unusual for any BChE. We classify the enzyme as an atypical BChE and discuss its implications for the evolution of AChE and BChE and for ecotoxicology.

## Introduction

Acetylcholinesterase (AChE; EC 3.1.1.7) hydrolyzes acetylcholine at the neuromuscular junction of vertebrates. Higher vertebrates also contain an evolutionarily related cholinesterase (ChE), butyrylcholinesterase (BChE, EC 3.1.1.8). The function of BChE is unknown but is suggested to play a role in growth and development and to act as a scavenger of cholinergic toxins as well as having an auxiliary role in synaptic transmission [1], [2]. The two ChEs may be distinguished functionally both kinetically and pharmacologically: AChE hydrolyzes acetylcholine (ACh) and is virtually inactive on the larger substrate butyrylcholine (BCh). BChE is less selective, hydrolyzing both substrates comparably. AChE exhibits inhibition at high substrate concentrations, while BChE shows substrate activation instead [3]. The two enzymes may also be distinguished by their susceptibility to diagnostic inhibitors [4].

Within species, AChE and BChE have ∼50% amino acid identity, and the overall tertiary structures of the two enzymes are similar [5], [6]. Individual amino acid residues involved in determining the molecular basis of the differences in substrate and inhibitor specificity of AChE and BChE have been identified in the acyl pocket, located at the bottom of a deep catalytic gorge; the peripheral site, located at the lip of the gorge; the oxyanion hole; and the choline-binding site of the hydrophobic patch, also located within the gorge [7]–[14]. Although the dichotomy between AChE and BChE is generally clear in birds and mammals [1], [15], [16], the two enzymes often more closely resemble one another functionally in fish. In the cartilaginous fish, the electric ray *Torpedo marmorata* [17], and the bony fishes, the plaice *Pleuronectes platessa* [18], the flounder *Platichthys flesus* [19], and perhaps the surgeonfish *Acanthuras dussumieri* [20], [21], ChEs with properties intermediate to and atypical of AChE and BChE are found along with AChE. These enzymes have alternatively been considered atypical ChEs [18], [19] or atypical pseudo-cholinesterases (pseudo-ChEs) [17], [20]; we are designating them as atypical BChEs, as suggested by Whittaker [22]. Although a number of cDNAs have been cloned for AChEs from these organisms, molecular information about the atypical BChEs present is unavailable. Moreover, only a single ChE, AChE, has been identified functionally and molecularly in the jawless fish, the lamprey *Petromyzon marinus* [23] and the hagfish *Myxine glutinosa* [24]. These observations suggest that AChE is the ancestral ChE in the vertebrates and that an early gene duplication event and subsequent divergent structural and functional evolution produced the AChE and BChE of higher vertebrates [23], [25].

 AChE and BChE also exist in a variety of homomeric and heteromeric molecular forms. The catalytic subunit of AChE is found in different variants as a result of alternative splicing of the C-terminus, producing R, H, and T (or AChE_R_, AChE_H_, or AChE_T_) subunits [26], [27]. The R, or read-through, transcript is rare and produces soluble non-amphiphilic monomers, G_1_^na^ [28]. AChE_H_ has a hydrophobic C-terminus, which is replaced by a glycophosphatidyl-inositol phospholipid (GPI) anchor and produces amphiphilic dimers, G_2_^a^ [29]. AChE_T_ is capable of forming G_1_^a^, G_2_^a^, and G_4_^na^ [29], as well as “tailed” forms (thus the T subunit) by associating with a transmembrane protein, the Proline-Rich Membrane Attachment (PRiMA) [30] and the triple helical collagen Q (ColQ; Q for queue, tail in French) [31], [32]. In brain and at the neuromuscular junction, PRiMA localizes AChE to the cell membrane of synapses, forming G_4_^a^ (or G_4_^P^). ColQ anchors AChE to the junctional basal lamina of the neuromuscular junction, producing A_4_, A_8_, and A_12_, which represent one, two or three tetramers attached to the ColQ triple helix. While AChE_T_ is found in all classes of vertebrates, AChE_H_ exists in cartilaginous fish (*Torpedo* spp.) [33], perhaps amphibians (*Xenopus laevis*) [34], and mammals [35], but has not been reported in jawless or bony fish, reptiles, or birds, raising questions about the evolution of this splice variant [26].

 BChE does not exhibit alternative splicing and is considered to be found solely as a T variant (BChE_T_) [36], [37] that also associates with PRiMA and ColQ [30], [36]. R and H variants of BChE have not been reported. However, according to the *Xenopus tropicalis* genome project [38] and other evidence [34], [39]–[42], an H variant of BChE appears to be present in amphibian *Xenopus* species. The atypical BChEs of *T. marmorata* and *A. dussumieri* are T variants (BChE_T_), assembling a collection of globular and asymmetric forms [17], [20]. In remarkable contrast, the atypical BChE of *P. flesus* is BChE_H_, assembling only into GPI-anchored G_2_^a^ forms [19].

The medaka *Oryzias latipes* is a teleost fish that is of interest as a vertebrate model system for developmental, genomic, and evolutionary biology [43]–[45]. It was previously reported that *O. latipes* possesses an AChE [46]. Here we report the cloning and characterization of an atypical BChE, which has properties intermediate to AChE and BChE, from *O. latipes*, and briefly discuss the implications of the structure and function of this enzyme for the evolution of the ChEs. Additionally, the presence of ChEs with anomalous inhibitor specificities has ecotoxicological implications for *O. latipes* [47], [48] and other fish [49], [50].

## Results

### Sequence Analysis Reveals Two ChEs in *O. latipes*

**Figure 1:**
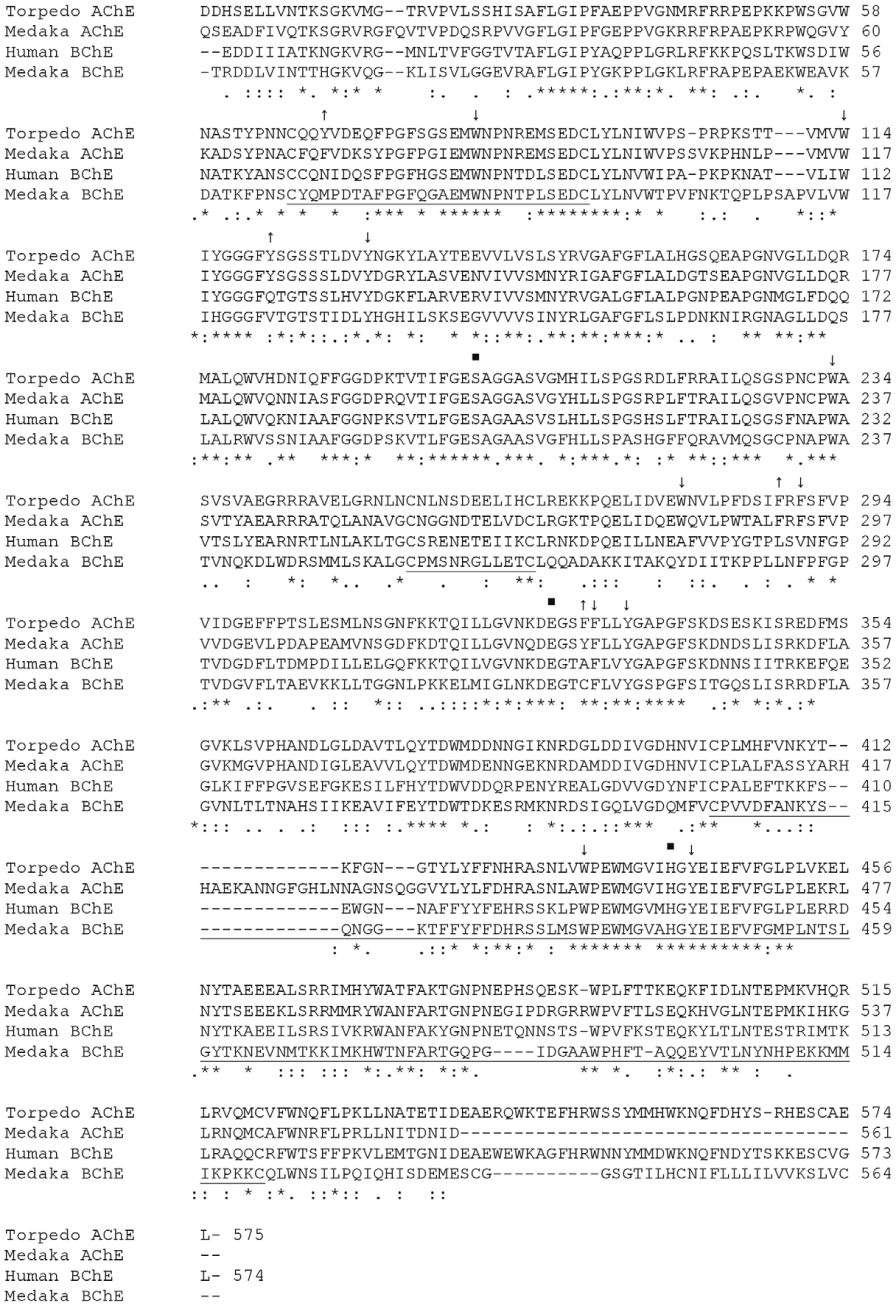
Alignment of peptide sequences of *Torpedo* AChE, Human BChE, Medaka (*O. latipes*) AChE and Atypical BChE. Numbering of the amino acid sequences is indicated on the right and starts with the amino acids of the mature polypeptide. Conserved (*) and similar (:.) residues are indicated. Locations of the three elements of the catalytic triad are indicated (•). Single underlines link the cysteines participating in intrachain disulfide bond. Sites of conserved aromatic amino acids lining the catalytic gorge in AChE are indicated with↓or↑; those not conserved in medaka (*O. latipes*) atypical BChE are marked with↑.

**Figure 2:**
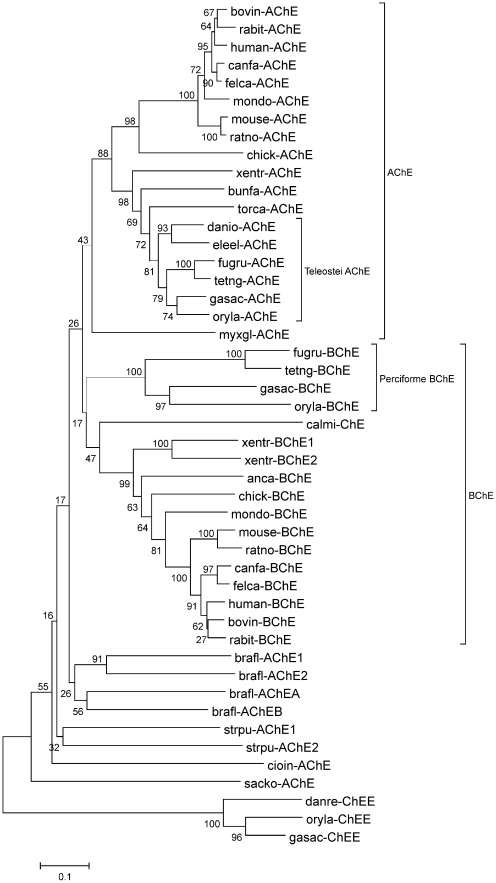
Evolutionary relationships of 47 taxa. The evolutionary history was inferred using the Neighbor-Joining method. The optimal tree with the sum of branch length  =  8.65 is shown. The percentage of replicate trees in which the associated taxa clustered together in the bootstrap test (1000 replicates) is shown next to the branches. The tree is drawn to scale, with branch lengths in the same units as those of the evolutionary distances used to infer the phylogenetic tree. The evolutionary distances were computed using the Poisson correction method [102] and are in the units of the number of amino acid substitutions per site. All positions containing gaps and missing data were eliminated from the dataset (Complete deletion option). There were a total of 257 positions in the final dataset. Phylogenetic analyses were conducted in MEGA4 [103]. Abbreviations and references found in ESTHER [104]. Mammals: bovin (*Bos taurus*) cattle, rabit (*Oryctolagus cuniculus)* rabbit, human (*Homo sapiens*), canfa (*Canis familiaris*) dog, felca (*Felis catus*) cat, mondo (*Monodelphis domestica*) possum, mouse (*Mus musculus*), ratno (*Rattus norvegicus*). Birds: chick (*Gallus gallus*) chicken. Amphibians: xentr (*Xenopus tropicalis*) toad. Reptiles: bunfa (*Bungarus fasciatus*) snake, anca (*Anolis carolinensis*) anole. Bony fish (teleosts): danio, danre (*Danio rerio*) zebrafish, eleel (*Electrophorus electricus*) electric eel, fugru (*Fugu rubripes*) puffer fish, tetng (*Tetraodon nigroviridus*) puffer fish, gasac (*Gasterosteus aculeatus*) stickleback, oryla (*Oryzias latipes*) medaka, calmi (*Callorhyncus milii*) elephant fish. Cartilaginous fish: torca (*Torpedo californica*) electric ray. Jawless fish: myxgl (*Myxine glutinosa*) hagfish. Urochordates: cioin (*Ciona intestinalis*) sea squirt, oikdi (*Oikopleura dioica*) tunicate appendicularium. Cephalochordates: brafl (*Branchiostoma floridae*) amphioxus. Hemichordates: sacko (*Saccoglossus kowalevskii*) acorn worm. Echinoderms: strpu (*Strongylocentrus purpuratus*) sea urchin.

Two expressed sequences for ChEs are present in the *O. latipes* genome: AChE (GenBank EST DK110600) and an enzyme that we are classifying as an atypical BChE [45] (GenBank cDNAs AV668390 and GU797251). The sequence for the AChE is truncated near the carboxyl terminus and contains 561 amino acids. The sequence of the mature polypeptide for the atypical BChE from *O. latipes* contains 564 amino acids (Fig. 1). Pair-wise BLAST alignments of sequences from the catalytic region of ChEs show that the AChE from *O. latipes* clearly resembles *T. californica* AChE rather than *Homo sapiens* BChE (68/80% identity/similarity to AChE compared to 54/70% for BChE), while the atypical BChE resembles both AChE and BChE more or less equally (46/68% for AChE and 49/67% for BChE). A phylogenetic tree of vertebrate and deuterostome invertebrate ChEs is shown in Fig. 2; the AChE of *O. latipes* is found in the AChE clade, while the atypical BChE of *O. latipes* is found in the BChE clade.

 The members of the catalytic triad of ChEs are found as Ser203, Glu330, and His461 in the AChE, and Ser203, Glu330, and His443 in the atypical BChE. Of the fourteen aromatic amino acids that line the catalytic gorge of vertebrate AChE, all are conserved in the *O. latipes* AChE, and ten are conserved in the atypical BChE; in contrast, eight are conserved in vertebrate BChE (Fig. 1; Table 1). The *O. latipes* atypical BChE is missing two of the three aromatic residues of the peripheral site of AChE, while BChE lacks all three. Additionally, while AChE has two Phe residues in the acyl pocket and BChE none, the *O. latipes* atypical BChE has one Phe (Fig. 1; Tables 1, 2). As the *O. latipes* AChE conserves all ten aromatic residues, it has two Phe residues in its acyl pocket.

**Table 1:** Aromatic Amino Acids in the Catalytic Gorge of Vertebrate ChEs.

Subsite	*Torpedo* AChE	*Oryzias* AChE	*Oryzias* BChE	*Homo* BChE
Peripheral Site	**Tyr70**	**Phe72**	Met69	Asn68
	**Tyr121**	**Tyr124**	Val124	Gln119
	**Trp279**	**Trp282**	**Tyr282**	Ala277
Hydrophobic Patch^1^	**Trp84**	**Trp86**	**Trp83**	**Trp82**
	**Tyr130**	**Tyr133**	**Tyr133**	**Tyr128**
	**Phe330**	**Phe333**	Cys333	Ala328
	**Phe331**	**Phe334**	**Phe334**	**Phe329**
Acyl Pocket	**Phe288**	**Phe291**	Leu291	Leu286
	**Phe290**	**Phe293**	**Phe293**	Val288
Wall of Gorge	**Trp114**	**Trp117**	**Trp119**	**Trp114**
	**Trp233**	**Trp236**	**Trp236**	**Trp231**
	**Tyr334**	**Tyr337**	**Tyr337**	**Tyr332**
	**Trp432**	**Trp453**	**Trp435**	**Trp430**
	**Tyr442**	**Tyr463**	**Tyr445**	**Tyr440**

Conserved aromatic residues are shown in bold. *Torpedo* AChE is representative

of all vertebrate AChEs and *Homo* BChE is representative of all vertebrate BChEs.

1 Includes the choline-binding site.

**Table 2:** Amino Acid Sequences in the Region of the Acyl Pocket of Vertebrate AChE and BChE.^a^

Enzyme	Class	Species	Sequence^b^
			288 290 400
AChE	Mammalia	*Felis catus*	VFRFSFVPVV…DHNVVCP
		*Bos taurus*	VFRFSFVPVV…DHNVVCP
		*Oryctolagus cuniculus*	LFRFSFVPVV…DHNVVCP
		*Homo sapiens*	VFTFSFVPVV…DHNVVCP
	Aves	*Gallus gallus*	VFRFAFVPVV…DHNVVCP
	Reptilia	*Bungarus fasciatus*	IFRFPFVPVI…DHNVICP
	Amphibia	*Xenopus tropicalis*	VFRFAFVPVP…DHNVICP
	Osteichthyes	*Electrophorus electricus*	LFRFSFVPVI…DHNVICP
		*Danio rerio*	LFRFSFVPVV…DQNVICP
		*Oryzias latipes*	LFRFSFVPVV…DHNVICP
	Chondrichthyes	*Torpedo* spp.	IFRFSFVPVI…DHNVICP
	Agnatha	*Myxine glutinosa*	I**F**RFPFVPVV…DINVICP
BChE	Mammalia	*Felis catus*	LLSVNFGPVV…DYNIICP
		*Bos taurus*	LLSVNFGPTV…DYNIICP
		*Oryctolagus cuniculus*	LLNFPFGPTV…DYNFICP
		*Homo sapiens*	PLSVNFGPTV…DYNFICP
	Aves	*Gallus gallus*	LLHIYFCPTV…DYHIICP
	Amphibia	*Xenopus tropicalis*	IIEMTFPPSV…DYNFICP
		*Xenopus tropicalis*	VIEVNFPPTV…DYNFICP
	Osteichthyes	*Gasterosteus aculeatus*	IIITPFVPYV…DQYFVCP
		*Fugu rubripes*	LGGYPFVPVV…DVLFVCP
		*Oryzias latipes*	LLNFPFGPTV…DQMFVCP

a GenBank accession numbers (unless otherwise noted) are for AChE: *F. catus* (AF053485), *B. taurus* (AF061813) *O. cuniculus* (U05036), *H. sapiens* (AK223443), *G. gallus* (U03472), *B. fasciatus* (U54591), *X. tropicalis* (ENSEMBL: ENSXETG00000017226), *E. electricus* (AF030422), *Danio rerio* (AJ251640), *O. latipes* (DK110600) *Torpedo* spp. (X03439, X05497), *M. glutinosa* (U55003). For BChE: *F. catus* (AF053483), *B. taurus* (M62410), *O. cuniculus* (X52090), *H. sapiens* (M16541), *G. gallus* (AJ306928), *X. tropicalis* (EG655516, CX359666), *G. aculeatus* (ENSEMBL: ENSGACG00000007230), *F. rubripes* (EMBL CAAB01000000), O. *latipes* (AV668390). Conserved Phe residues of acyl pockets in bold; conserved Phe residue of BChE implicated in aromatic trapping is underlined.

b Numbering of acyl pocket residues: Phe288, Phe290, and Val400 in *Torpedo* spp. AChE. Conserved Phe (F) residues of acyl pockets in bold; conserved Phe residue of BChE implicated in aromatic trapping is underlined.

**Figure 3:**
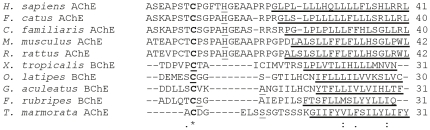
Alignment of peptide sequences of C-termini of representative vertebrate AChEs and BChEs. Conserved (*) and similar (:.) residues are indicated. Putative Ω-cleavage sites are underlined. Putative hydrophobic transmembrane regions are boldly underlined. *H. sapiens* (human), *F. catus* (cat), *C. familiaris* (dog), *M. musculus* (mouse), *R. norvegicus* (rat), *X. tropicalis* (clawed toad), *O. latipes* (medaka), *G. aculeatus* (stickleback), *F. rubripes* (fugu), *T. marmorata*, electric ray.

The three pairs of conserved cysteine residues involved in intra-chain disulfide bonding are also found as Cys69-Cys96, Cys257-Cys268, and Cys405-Cys543 in the AChE; and Cys66-Cys93, Cys257-Cys268, and Cys405-Cys520 in the atypical BChE of *O. latipes*. Another cysteine (Cys540), near the carboxyl terminal that normally mediates inter-chain disulfide bonding, is also conserved in the atypical BChE (Fig. 1). The carboxyl terminus of the enzyme is of the H-type (Fig. 3), characterized by a loosely defined GPI anchor signal, including an ω cleavage/attachment site followed by a stretch of hydrophobic amino acids [51]. The H-peptides show very little sequence homology to one another. We did not find evidence for the existence of T-type carboxyl terminus for the atypical BChE either as a T-exon in the genome or a T-type carboxyl terminus in the ESTS of *O. latipes*. The truncated AChE is missing its C-terminal sequence.

### Diagnostic Inhibitors Show the Presence of Two ChE Activities in Adult *O. latipes*

**Figure 4:**
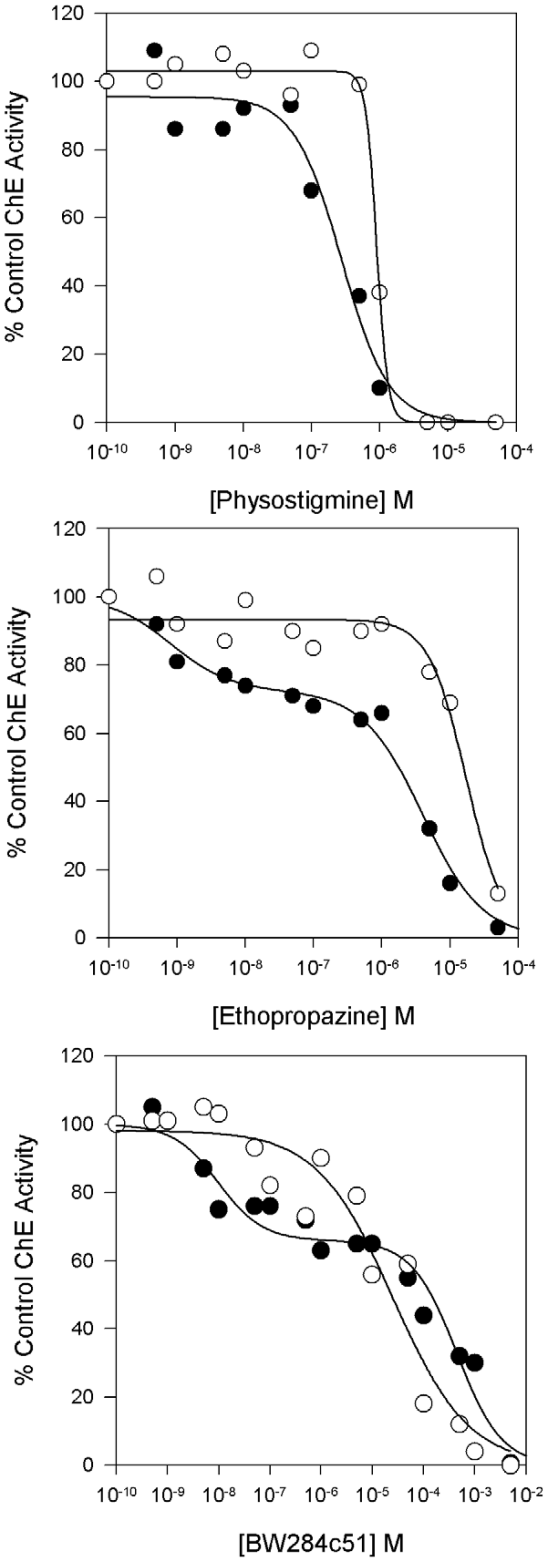
Concentration dependencies for inhibition of ATCh and PTCh hydrolysis by extract from adult *O. latipes*. Adult fish were extracted in HIS buffer and assayed with ATCh and PTCh in the presence of various concentrations of the inhibitors (A) physostigmine, (B) ethopropazine, and (C) BW284c51. Extracts were incubated with inhibitor for 20 minutes prior to being assayed for activity with ATCh (○) or PTCh (•).

To demonstrate the presence of two ChE activities in *O. latipes,* extracts from adult medaka were incubated with the inhibitors physostigmine, which inhibits all ChEs; BW284c51, which inhibits AChE selectively; and ethopropazine, which inhibits BChE preferentially, and assayed for activity with ATCh and PTCh [16], [52], [53]. Different dose-response curves were observed with the two substrates for each inhibitor, suggesting the presence of at least two ChE activities (Fig. 4). The dose-response curves for inhibition of PTCh hydrolysis by BW284c51 and ethopropazine are biphasic, clearly indicating the presence of two ChE activities, presumably the AChE and atypical BChE identified in the *O. latipes* genome project.

### Kinetic Analysis of the Atypical BChE Indicates Its Anomalous Properties

**Figure 5:**
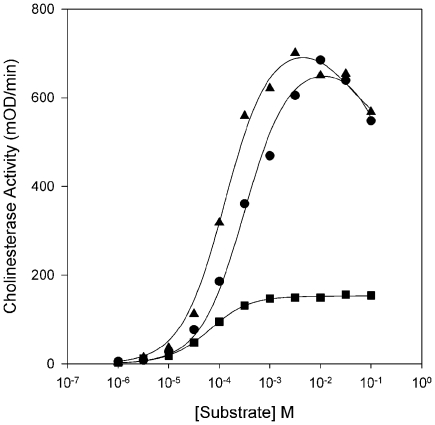
Substrate concentration dependencies for hydrolysis of ATCh, PTCh, and BTCh by recombinant BChE from *O. latipes*. Transfected COS-7 cells producing BChE were extracted in HIS buffer and assayed with ATCh (•), PTCh (□), or BTCh (▴) and fit as described in Materials and Methods.

As we were interested in the functional characteristics of the atypical BChE in *O. latipes*, we cloned and expressed *in vitro* a cDNA for the enzyme in COS-7 cells. To determine the substrate specificity of the enzyme, we assayed the hydrolysis of the substrates acetylthiocholine (ATCh), propionylthiocholine (PTCh), and butyrylthiocholine (BTCh) by the recombinant enzyme (Fig. 5). The smaller substrates ATCh and PTCh are hydrolyzed more or less equally, as indicated by the similar values of *k_cat_* ^Substrate^/*k_cat_* ^ATCh^ (Table 3); the larger BTCh is hydrolyzed at about a quarter of the rate of the other two substrates. The *K_m_*s are inversely proportional to the length of the acyl group, with BTCh having the lowest *K_m_*. The highest catalytic efficiency (*k_cat_/K_m_*) is seen with PTCh. Additionally, ATCh and PTCh produce substrate inhibition (i.e., lower enzyme activity at high substrate concentrations and *b* parameter values of <1), although the inhibition by PTCh is weak (Fig. 5; Table 3). BTCh does not produce substrate inhibition. Overall, this pattern of substrate hydrolysis is not typical of either AChE or BChE.

**Table 3:** Kinetic Parameters for Recombinant ChE from *O. latipes*.^a^

Substrate	*K_m_* µM	*K_ss_* mM	*b*	*k_cat_* min^−1^	*k_cat_* ^Substrate^/*k_cat_* ^ATCh^	*k_cat_/K_m_* M^−1^ min^−1^
ATCh	270±18	516±52	0^b^	2.76±0.11×10^4^	1.00	1.03±0.05×10^8^
PTCh	156±15	20±12	0.67±0.02	3.07±0.24×10^4^	1.11	2.00±0.05×10^8^
BTCh	72±6	39±29	0.92±0.07	0.70±0.03×10^4^	0.25	0.99±0.07×10^8^

a Data are the mean ± SE of 6–8 determinations.

b Values of *b* less than 0.02 are indistinguishable from zero.

### Pharmacological Analysis of the Atypical BChE Confirms Its Anomalous Properties

**Figure 6:**
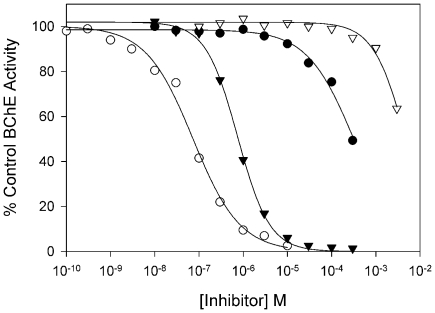
Concentration dependencies for inhibition of ATCh hydrolysis by recombinant BChE from *O. latipes*. Transfected COS-7 cells producing BChE were extracted in HIS buffer and assayed with ATCh in the presence of various concentrations of inhibitors. Extracts were incubated with inhibitor for 20 minutes prior to being assayed for activity: physostigmine (○), ethopropazine (▾) BW284c51 (•), iso-OMPA (▿). Data were fit to a three-parameter logistic function as described in Materials and Methods.

Since the recombinant enzyme from *O. latipes* exhibited anomalous kinetic properties, to characterize further this atypical BChE activity, we determined the half maximal inhibitory concentration (IC_50_) values of the enzyme for the inhibitors physostigmine, which inhibits all ChEs; BW284c51, which inhibits AChE preferentially; and iso-OMPA and ethopropazine, which inhibit BChE preferentially. Physostigmine and ethopropazine inhibit the enzyme at sub-µM concentrations; by contrast, much higher concentrations of BW284c51 and Iso-OMPA are required for inhibition under the conditions tested (Fig. 6; Table 4). This pattern of inhibition is also not characteristic of either AChE or BChE.

**Table 4:** IC_50_ Values for Inhibition of Recombinant ChE from *O. latipes*.^a^

Inhibitor	IC_50_ M
Physostigmine	5.85±0.79×10^−8^
Ethopropazine	8.98±0.75×10^−7^
BW284c51	4.80±0.82×10^−4^
Iso-OMPA	4.58±1.52×10^−3^

a Data are the mean ± SE of 3–5 determinations.

### Analysis of Molecular Forms of the Atypical BChE further Demonstrates Its Unusual Nature

**Figure 7:**
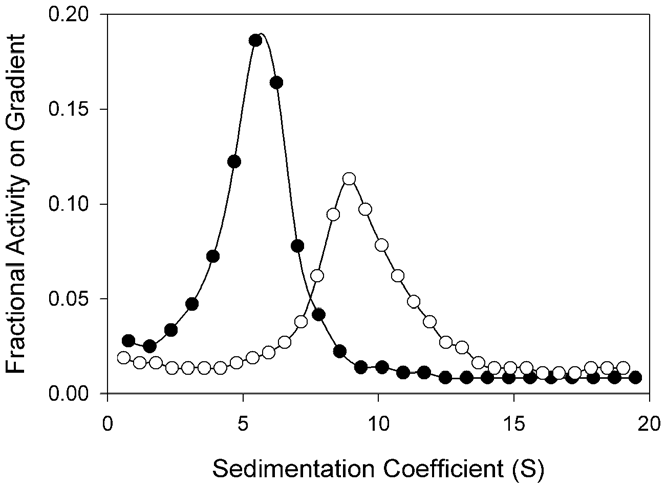
Velocity sedimentation analysis of the molecular forms of recombinant *O. latipes* BChE. HIS extracts from COS-7 cells transfected with cDNA for BChE were sedimented on gradients prepared in presence (•) and absence (○) of Triton X-100 as described in Materials and Methods. Data are presented as the fraction of total BChE activity on the gradient as a function of sedimentation coefficient.

**Figure 8:**
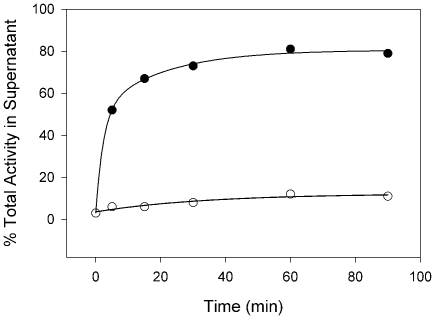
Release of BChE from transfected COS-7 cells by PIPLC. Equal aliquots of transfected cells were harvested intact and incubated at 37°C in PBS in the presence (•) or absence (○) of 4 units/ml of PIPLC, and cells and supernatants were assayed for BChE activity as described in Materials and Methods. Percent of the total activity recovered that is present in the supernatant is indicated.

 ChEs exist in various homomeric and heteromeric molecular forms depending, in part, on the nature of their carboxyl termini. Since the amino acid sequence of the atypical BChE indicates an H-type C-terminus, we performed velocity sedimentation on sucrose gradients in the presence and absence of the non-ionic detergent Triton X-100 to determine the molecular forms of the recombinant enzyme produced *in vitro* by COS-7 cells. The extract contains G_2_^a^ forms on the basis of the sedimentation coefficient (5.25±0.10 S; Mean±SE, N = 6) and its shift to higher values in the absence of detergent due to aggregation of the enzyme (8.96±0.05; Mean±SE, N = 6) (Fig. 7). Digestion of intact COS-7 cells with phosphatidylinositol-specific phospholipase C (PIPLC) releases ∼80% of the surface enzyme activity. Spontaneous release of activity into the supernatant during incubation in the absence of PIPLC was ∼10% (Fig. 8). These data indicate that the G_2_^a^ produced is a glycophosphatidylinositol-anchored (GPI-anchored) form. It is unusual for any BChE to be found as a GPI-anchored form

### Molecular Modeling of the Atypical BChE Illustrates Its Differences with AChE and BChE

**Figure 9:**
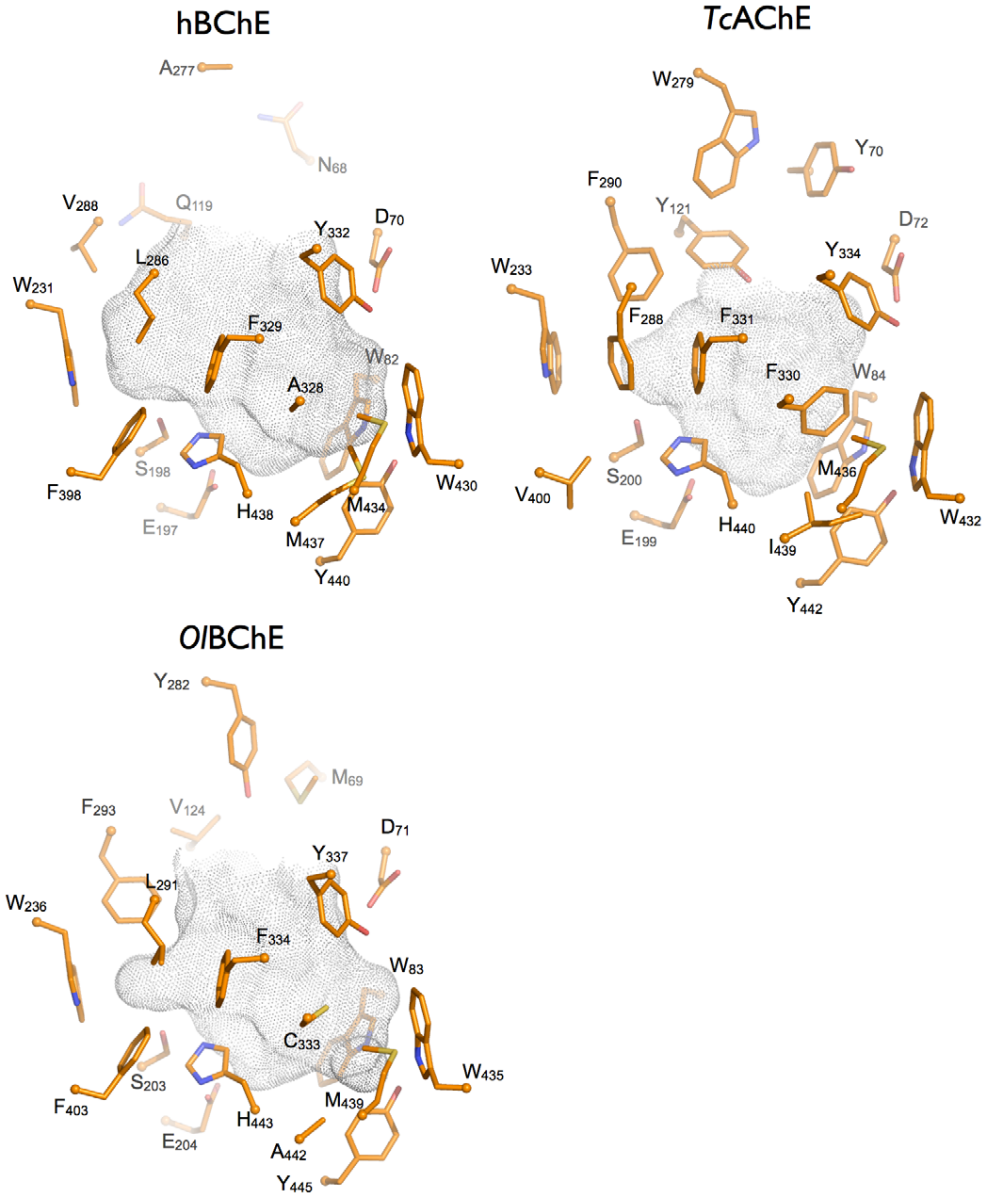
Active site gorges of *H. sapiens* BChE (hBChE; pdb code 1p0i), *T. californica* AChE (*Tc*AChE; pdb code 1ea5) and a homology model of *O. latipes* atypical BChE (*Ol*BChE). The side chains of key residues lining the gorges are represented as sticks (carbon in orange, oxygen in red, and nitrogen in blue). The top of the gorge and the entrance to and exit from the enzyme are shown at the top of the figures. The acyl pocket, which accommodates the acyl group of substrates, is comprised of residues F288 and F290 in *T. californica* AChE, L286 and V288 in *H. sapiens* BChE, and L291 and F293 in *O. latipes* AChE. The central residue of the choline binding site at the bottom of the gorge is W84 in *T. californica* AChE, W82 in *H. sapiens* BChE, and W83 in *O. latipes* atypical BChE. Substrates are bound between these two subsites. The solvent accessibility surface of the gorge was calculated by taking into account highly conserved structural water molecules, using the software “HOLLOW” and represented as grey dots [99].

We built a homology model of the atypical BChE from *O. latipes* based on the X-ray structures of *H. sapiens* BChE, and *T. californica* and *Drosophila melanogaster* AChEs, in order to get a structural understanding of the special pharmacological and enzymatic properties of the enzyme (Fig. S1). A comparison of the active site gorges is presented in Figure 9. The volume of the *O. latipes* atypical BChE catalytic gorge (630 Å^3^) is much closer to that of *H. sapiens* BChE (690 Å^3^) than that of *T. californica* AChE (410 Å^3^). The difference in volume is related to the lack of aromatic residues in three gorge subsites: the peripheral site, the acyl binding pocket, and the choline binding pocket. *T. californica* AChE possesses three conserved residues (Tyr70, Tyr121, and Trp279) that form an aromatic peripheral binding site and restrict access to the gorge. None of these aromatic residues is conserved in *H. sapiens* BChE and only one aromatic residue is present in the *O. latipes* atypical BChE (Tyr282), thus enlarging the gorge entrance (Table 1).

The acyl binding pocket of *T. californica* AChE is also smaller than that of the atypical BChE from *O. latipes,* which is in turn smaller than that of *H. sapiens* BChE, due to the respective decreasing number of the phenylalanines shaping the pocket. These differences are expected to change the selectivity of the enzymes for substrates and inhibitors bearing large chains oriented toward this pocket.

Another major difference between the *O. latipes* atypical BChE and *H. sapiens* BChE and *T. californica* AChE is located in the choline binding pocket. Phe330 (a tyrosine in mammalian AChE) is substituted by a non-aromatic residue in both BChEs (Ala328 in the *H. sapiens* BChE and Cys333 in the *O. latipes* atypical BChE). Phe330 can adopt different conformations in the presence of different ligands thus providing an efficient way to modulate the shape and size of the choline binding pocket, even acting as a molecular lid. The absence of a gating aromatic residue at this position in BChE leaves the gorge wide open at all times. Notably, just next to this position, the substitution of Met442 in *H. sapiens* BChE by Ala440 in *O. latipes* BChE results in a significantly widened choline binding pocket.

All things considered, the active site gorge of the atypical BChE from *O. latipes* shares more structural features with that of *H. sapiens* BChE than that of *T. californica AChE*, and it appears legitimate to consider this enzyme as an atypical BChE from a structural point of view.

## Discussion

### Overview

In addition to possessing an AChE, the medaka *O. latipes* has an atypical BChE that is in many respects different from either vertebrate AChE or BChE (Table 5). Of the fourteen aromatic amino acids in the catalytic gorge of vertebrate AChE, ten are conserved in the atypical BChE from *O. latipes*; by contrast, eight are conserved in vertebrate BChE. These substitutions may account for the intermediate nature of the atypical BChE. Molecular modeling supports this interpretation. The enzyme hydrolyzes ATCh and PTCh preferentially, but BTCh to a considerable extent. In contrast, AChE is highly specific for ATCh compared to BTCh and even PTCh, while BChE hydrolyzes the larger substrates preferentially. The *K_m_*s for substrate hydrolysis by the atypical BChE are inversely proportional to the length of the acyl group, which is more a characteristic of BChE than AChE. The enzyme shows substrate inhibition with the two smaller substrates but not the larger substrate BTCh. By comparison, AChE exhibits substrate inhibition, while BChE does not, but may instead show substrate activation. The *O. latipes* enzyme also shows an atypical pattern of inhibition by diagnostic inhibitors. It is effectively inhibited by the ChE inhibitor physostigmine, typical of all ChEs. However, although the atypical BChE is efficiently inhibited by the BChE-specific inhibitor ethopropazine, it is not inhibited by another BChE inhibitor, iso-OMPA, nor by the AChE-specific bis-quaternary inhibitor BW284c51. The atypical BChE is found as a GPI-anchored G_2_^a^ membrane-bound dimeric form, also unusual for a BChE. We consider the enzyme an atypical BChE that has implications for the evolution of AChE and BChE in the vertebrates.

**Table 5:** Comparison of Available Kinetic and Pharmacological Parameters and Splice Variants of Vertebrate AChE, BChE, and Atypical BChEs.

Parameter/Species	*T.marmorata* AChE^a^	*O.latipes* BChE	*P.flesus* BChE^b^	*P.platessa* BChE^c^	*A.dussumieri* BChE^d^	*T.marmorata* BChE^a^	*H.sapiens* BChE
*K_m_* ^ATCh^ mM	0.05	0.27	2.20	0.90	1.24	0.4	1.4^e^
*K_m_* ^PTCh^ mM	0.2	0.16	1.03	0.15	0.24	0.15	0.97^e^
*K_m_* ^BTCh^ mM	N.M.^j^	0.07	0.32	0.14	0.06	0.05	0.91^e^
*V_max_* ^PTCh^/*V_max_* ^ATCh^	0.25	1.11	0.29	0.41	0.61	0.59	1.66^e^
*V_max_* ^BTCh^/*V_max_* ^ATCh^	<0.01	0.25	0.22	0.58	0.03	0.28	2.41^e^
Substrate Inhibition^i^	+A,−P	+AP,−B	+APB	−AP,+B	+APB	−APB	-APB
IC_50_ Ethopropazine µM	158	0.90	12.6	N.D.^k^	N.D.^k^	100	15.3^f^
IC_50_ BW284C51 µM	0.04	480	63	0.79	Weak	100	651^g^
Splice Variant^l^	H, T-type	H-type	H-type	N.D.^k^	T-type	T-type	T-type^h^

a Data are from Toutant et al., [17]; the IC_50_ values are estimates.

b Data are from Stieger et al. [19].

c Data are from Lundin and represent titremetric measurement of the hydrolysis of the oxyesters rather than the thioesters of substrates [18].

d Data are from Leibel and inhibition by BW284c51 is based on qualitative observations of the inhibition of ATCh staining of enzyme following non-denaturing gel electrophoresis [21]. Splice variant data are from Leibel [20].

e Data are from Davies et al. [100].

f Data are from Ucar et al. [101].

g Data are from Loewenstein-Lichtenstein et al.; IC_50_ was back calculated from a calculated *K_i_* [69].

h Data are from Blong et al. [37].

i A, acetylthiocholine; P, propionylthiocholine; B, butyrylthiocholine; +, inhibition by high substrate concentrations; -, no inhibition; substrate activation is not considered.

j N.M., not measureable.

k N.D., not determined.

l Splice variant determined by molecular forms present and sequence data when available.

### Comparison of Kinetic and Pharmacological Parameters of the Atypical BChE from *O. latipes* with AChE, BChE, and other Atypical BChEs in Vertebrates

Fluck [46] characterized the acylcholine hydrolyzing activity of early embryos of *O. latipes* and found that ATCh, PTCh, and BTCh were hydrolyzed at the relative rates of 1.0, 0.4, and 0.14, respectively; that ATCh, at least, produced substrate inhibition; and that 10 µM BW284c51 inhibited 90% of the activity, while 10 µM iso-OMPA inhibited only 10% of the activity. He concluded that the enzyme present was AChE. Given the results from the *O. latipes* genome project, which indicate two ChEs in the genome; our results for BW284c51 and ethopropazine inhibition of the ChE activity of adult medaka, which also suggest two ChEs; and our data for the recombinant atypical BChE from *O. latipes*, it seems likely that both AChE and BChE were present in the embryos studied by Fluck [46].

The *K_m_*s for substrate hydrolysis by the atypical BChE from *O. latipes* are inversely proportional to the length of the acyl chain of the substrate (Table 5). This relationship is seen in *H. sapiens* BChE, which is representative of the typical vertebrate BChE; but not *T. marmorata*, which is representative of vertebrate AChE. This pattern is the most conserved characteristic of the atypical BChEs from the flounder *P. flesus*, the plaice *P. platessa*, the surgeonfish *A. dussumieri*, and the ray *T. marmorata*, suggesting that the non-covalent stabilization of binding of substrate is determined similarly in these enzymes. The acyl pocket, Phe288 and Phe290 in *Torpedo* spp. AChE, is implicated in this binding [8], [12]-[14]. A detailed discussion of the molecular basis of *K_m_* and other parameters of substrate and inhibitor specificity is given in the next section; since sequence information is only available for the atypical BChE from *O. latipes*, it alone, among the atypical BChEs, can be compared to AChE and BChE at the molecular level.

In contrast to the clear series seen for *K_m_*, the substrate hydrolysis specificities of the enzymes, as defined by relative *V_max_* or *k_cat_* values, do not show as consistent a pattern. Most of the atypical BChEs hydrolyze ATCh preferentially and BTCh the least. An exception is the *O. latipes* atypical BChE, which hydrolyzes PTCh maximally, albeit only slightly faster than ATCh. However, there are two additional exceptions to this generalization: the *P. platessa* atypical BChE hydrolyzes BTCh faster than PTCh, and the *A. dussumieri* atypical BChE hydrolyzes BTCh only marginally better than does AChE. The variation in relative *V_max_* or *k_cat_* values suggests that there are differences in the stabilization in the covalent Michaelis complexes of substrates in the various atypical BChEs. Such differences could be due to differences in the nature of the conformation of the histidine of the catalytic triad, which appears to be determined by different sets of structural interactions in AChE and BChE [9], [54].

 There are also four different patterns of substrate inhibition among the five atypical BChEs, indicating another heterogeneity among the enzymes. The physiological relevance and molecular mechanism of substrate inhibition are unclear [55]–[57]. Nevertheless, these differences in the enzymes are probably due to alterations of amino acids, probably non-aromatic substitutions, comprising the peripheral site and/or other regions of the catalytic gorge in the various enzymes that are important for substrate inhibition [9], [13], [58], [59]. It is notable that the substrate hydrolysis curve for PTCh hydrolysis by the *O. latipes* atypical BChE, particularly in the region of substrate inhibition resembles the curves for ATCh and PTCh hydrolysis by the atypical BChE from *A. dussumieri*, as the substrate inhibition levels off in both of the enzymes [21].

The atypical BChEs also show different patterns of sensitivity to inhibitors; however, given the range of inhibitors used, and focusing on the atypical BChE from *O. latipes*, it is possible to compare their inhibition only for the AChE and BChE diagnostic inhibitors BW284c51 and ethopropazine, and even for these inhibitors the data are incomplete and a consistent pattern elusive. Of the atypical BChEs, those from *O. latipes* and *P. flesus* resemble most closely BChE, being sensitive to ethopropazine, but not BW284c51; the BChE from *T. marmorata* is equally in/sensitive to both inhibitors, while the BChEs from *P. platessa* and *A. dussumieri* appear to be sensitive and resistant, respectively. These inhibitors are sensitive to the presence or absence of aromatic amino acids present in the choline-binding and peripheral sites, as well as to the volumes of the catalytic gorges [12], [14], [60], [61], which could be and probably are different in the various atypical BChEs. Overall, the variety of kinetic and pharmacological properties of these atypical BChEs could be the result of natural selection exploring the adaptive landscape for the various enzymes.

### Molecular Basis of Substrate and Inhibitor Specificity in the Atypical BChE from *O. latipes* in Comparison to AChE and BChE

The atypical BChE from *O. latipes* maximally hydrolyzes ATCh and PTCh almost equally and the larger substrate BTCh at about a quarter of the rate of the two other smaller substrates. This substrate specificity is not typical of either AChE, which hydrolyzes ATCh maximally, PTCh adequately, but not BTCh at all; nor BChE, which hydrolyzes the three substrates more or less equally [15]. An important molecular determinant of ChE specificity is the acyl pocket, which in vertebrate AChE is characterized by two Phe residues (Phe288 and Phe290 in *Torpedo* spp. AChE) [6], while in BChE these aromatic residues are replaced by smaller aliphatic amino acids (Leu286(288) and Val288(290) in *H. sapiens* BChE) [5]. Site-directed mutagenesis studies suggest that the smaller amino acids relax the steric hindrance of the aromatic rings and allow the accommodation and proper positioning of larger substrates in the catalytic gorge for nucleophilic attack by the active site serine [8], [12], [14]. The simple fact that the atypical BChE from *O. latipes* has one of the two Phe residues, Phe291 (Phe290 in *Torpedo* spp. AChE) but not the other, present as Val288 (Phe290), seems sufficient to explain the intermediate substrate specificity of the enzyme. Our molecular modeling is consistent with this explanation.

In the mouse *Mus musculus* AChE, the F295L (F288L) mutation has little effect on the *K_m_* for ATCh but lowers the *K_m_* for BTCh 30-fold [14]. In *H. sapiens* AChE, F295L has little effect on the *K_m_* for ATCh or PTCh but decreases *K_m_* for BTCh 10-fold [12]. In contrast, F295A also spares the *K_m_* for ATCh, but decreases *K_m_* for PTCh and BTCh 4-fold and 33-fold, respectively. With respect to *k_cat_*, the F295L *M. musculus* AChE mutant decreases *k_cat_* 4-fold for ATCh and increases it 14-fold for BTCh. In *H. sapiens* AChE, the F295L, A mutants do not affect appreciably the *k_cat_* for ATCh but increase *K_m_* 400-fold. For wild type *H. sapiens* and *M. musculus* AChE, the highest catalytic efficiency (*k_cat_/K_m_*) by far is seen for ATCh; whereas, for the F295L, A mutants, the highest efficiency is found for BTCh, with the efficiency for all three substrates within a factor of three. These data are consistent with, if not identical to, the results that we have obtained for the *O. latipes* atypical BChE, which lacks the homologous Phe: all three substrates are hydrolyzed appreciably, the *K_m_* is lowest for BTCh but lower for PTCh compared to ATCh, and the catalytic efficiency for all the substrates are within a factor of two. Thus, it appears that the residue corresponding to Phe288 affects both the binding and hydrolysis of substrate.

Furthermore, the two phenylalanines (Phe288 and Phe290) that shape the acyl binding pocket of AChE, along with the aromatic peripheral site residues, form an aromatic continuum with Phe330 and Phe331 of the hydrophobic patch. This aromatic network in AChE has been suggested to play an important role in the stabilization of the catalytic histidine [54]. But Phe288 and Phe290 are not conserved in *H. sapiens* BChE, and Phe288 is not conserved in the atypical BChE from *O. latipes* (Leu291). Thus, this stabilizing network is absent in BChE. However, the catalytic histidine of BChE is well stabilized by interactions with an adjacent phenylalanine, Phe398 in *H. sapiens* BChE or Phe403 in *O. latipes* atypical BChE, which is absent in AChE (Val400 in *T. californica* AChE). As a matter of fact, the catalytic histidine of *H. sapiens* BChE has never been observed in an alternate conformation, whereas such a conformation is not unusual in liganded AChE. (See X-ray structure of VX-AChE (pdb entry 2VXR), tabun-AChE (pdb entry 3DL4) and the NMR study of Masiah et al. [62]). Interestingly, when the acyl loop of a cholinesterase bears an aliphatic residue at the position equivalent to Leu291 in *O. latipes* atypical BChE, there is an aromatic residue facing it, Phe401. Reciprocally, when there is an aromatic residue at this position, as with Phe288 in *T. californica* AChE, there is an aliphatic residue facing it, Val400 (Table 2). This symmetrical situation probably translates into a difference in the stability of the catalytic histidine and the acyl pocket loop, which in turn might affect the stability of the transition state during catalysis. It should be noted that in the vast majority of invertebrate AChEs the acyl pocket appears to be constructed in a different way with additional or alternative aromatic residues playing a role in substrate specificity [63]. Thus, modifications of the acyl pocket appear to occur throughout animal evolution.

The atypical BChE of *O. latipes* is efficiently inhibited by physostigmine and ethopropazine but not BW284c51 or iso-OMPA, a pattern of inhibition different from AChE or BChE. However, we think that the pattern more closely resembles BChE rather than AChE with the inhibition by iso-OMPA being exceptional. All ChEs are inhibited by physostigmine, so its effective inhibition simply confirms that the enzyme is a ChE [16] and does not need discussion. The sensitivity of AChE to the slender, elongated bisquaternary inhibitor, BW284c51, is due to its bivalent binding via cation-π and π-π interactions to aromatic amino acids of the choline-binding site at the bottom of the gorge and the peripheral site at its rim. In contrast, BChE has a number of these residues replaced by aliphatics. In *M. musculus* AChE, the peripheral site double mutant Y72N/Y124Q (Y70N/Y121Q) increases *K_i_* for BW284c51 69-fold [13]. Comparably, in *H. sapiens* AChE the same double mutation increases *K_i_* for the inhibitor 35-fold. Moreover, the choline-binding site mutation Y337A (Phe330A) increases the *K_i_* 5-fold [60]. Making the reasonable assumption of a synergistic effect for the mutations [60], a triple Y72N/Y124Q/Y337A mutation encompassing both the peripheral and choline-binding sites could increase *K_i_* by over two orders of magnitude. Thus in the *O. latipes* atypical BChE, which has the aliphatic substitutions Met69, Val124, and Cys333 at the homologous sites, preventing the necessary cation-π and π-π interactions via the aromatic residues, one might expect inefficient inhibition by BW284c51, which is exactly what is observed. Notably, the structure of the acyl pocket does not appear to influence the binding of BW284c51 [13], [14], [60], consistent with the situation in the atypical BChE of *O. latipes*.

The atypical BChE of *O. latipes* is inhibited by a low concentration of the tricyclic amine-containing phenothiazine, ethopropazine, which contains a bulky diethylamino-2-isopropyl exocyclic group – inhibition typical of vertebrate BChE. Inhibition of AChE and BChE by ethopropazine appears to be sensitive to the nature of the residue corresponding to Phe330 of the aromatic patch in *Torpedo* spp. AChE and to the volume of the active center in BChE [13], [61]. In *M. musculus* AChE, the Y337A (F330A) mutation decreased *K_i_* almost 2700-fold, making it comparable to BChE [13], presumably because the side chain of Tyr337 sterically hinders the binding of ethopropazine via an interaction between the aromatic side chain of the residue and the diethylamino-2-isopropyl moiety of the inhibitor. By contrast, the converse mutations A328F and A328Y in *H. sapiens* BChE do not substantially affect the *K_i_* for the inhibitor [61]. There is, however, a large difference in the gorge dimensions between AChE and BChE, and this difference was used to explain the lack of effect. The volume of the lower portion of the gorge in *Torpedo* spp. AChE was reported to be 302 Å^3^, and in a modeled *H. sapiens* BChE it was measured as 502 Å^3^. The van der Waals volume of ethopropazine is 318 Å^3^, explaining why the drug does not bind well to AChE. The F330A mutation of *Torpedo* spp. AChE increases the lower gorge volume to 338 Å^3^ and allows ethopropazine to bind. In contrast, the A328Y and A328F mutations in the *H. sapiens* BChE model only decrease the volume of the lower gorge to 410 and 406 Å^3^, respectively, which allow the gorge to still be large enough to bind ethopropazine easily [61]. Our own volume calculations using the narrower part of the bottleneck to define the gorge entrance and taking into account the conserved structural water molecule, gives an overall volume of 690 Å^3^ for *H. sapiens* BChE, 630 Å^3^ for *O. latipes* atypical BChE and 410 Å^3^ for *Torpedo* spp. AChE, in good agreement with the observed trend for ethopropazine inhibition.

 Iso-OMPA is an effective organophosphate inhibitor of BChE but not AChE [52], where it is over 10,000 times less reactive [14], and this selectivity appears dependent on the dimensions of the active center of the enzyme, particularly the acyl pocket, affecting the affinity of the enzymes for inhibitor. In *M. musculus* AChE, the acyl pocket mutation F295L (F288L) increases *k_i_* 90-fold, F297I (F290I) 200-fold, and the double mutant, over 500-fold for iso-OMPA [14]. In *H. sapiens* AChE, similar, although more complex, results were seen for the less bulky organophosphates, diisopropyl phosphorofluoridate (DFP), diethyl phosphorofluoridate (DEFP), and paraoxon [64]. Replacement of aromatic amino acids in the acyl pocket with aliphatic residues increases *k_i_* up to 130-fold, with substitutions at Phe295 (Phe288) having the greater effect. The differences in *k_i_* were primarily due to decreases in *K_d_* with *k_2_* relatively unaffected, suggesting that the substitutions relieved steric interference in the binding of the inhibitors and enhanced enzyme affinity, but did not alter the rate of phosphorylation. Unlike ethopropazine, converse site-directed mutagenesis of the acyl pocket has not been reported for BChE and iso-OMPA. The atypical BChE from *O. latipes* is relatively insensitive to, but is inhibited by iso-OMPA. This result, as well as the substrate specificity of the enzyme, is in good agreement with the reduction in size of the acyl pocket, compared to BChE, associated with the presence of only one of the two aromatic residues in the subsite in our molecular modeling. This explanation, as well as the others offered on the molecular basis of substrate and inhibitor specificity could be tested by site-directed mutagenesis. In any event, it should be kept in mind that vertebrate ChEs cannot always be characterized as AChE or BChE simply on the basis of diagnostic inhibitor specificities [53], and that molecular analysis can provide additional valuable information about the nature of ChE activity. Since molecular data are not available for the atypical BChEs from *T. marmorata*, *P. flesus, P. platessa,* and *A. dussumieri*, we cannot compare at the molecular level the atypical BChE from *O. latipes* with these enzymes.

### Comparison of the Molecular Form of *O. latipes* Atypical BChE with AChE, BChE, and other Atypical BChEs in Vertebrates

The atypical BChE from *O. latipes* is a GPI-anchored G_2_^a^ membrane-bound dimer and thus resembles a BChE_H_, possessing an H-type C-terminus, which is removed upon the addition of the GPI anchor. The atypical BChE of *P. flesus* is also a GPI-anchored G_2_^a^ [19]. However, the atypical BChEs from *T. marmorata* and *A. dussumieri* are BChE_T_ types since they are capable of assembling into G_1_, G_2_, and G_4_ molecular forms, and are able to interact with the collagenic tail protein ColQ [17], [20]. The forms of the *P. platessa* enzyme are not known. While many vertebrate AChEs, including *Torpedo* spp. and mammalian AChE are found as AChE_T_ and AChE_H_ as the result of alternative splicing, all vertebrate BChEs are considered to be of the T-type [26]; the atypical BChEs from *O. latipes* and *P. flesus* are the only certain exceptions to this rule. The nature of the ChE activity in *Xenopus* spp. is perplexing. A PIPLC-sensitive G_2_ AChE_H_ has been reported in *X. laevis* muscle [34], [41], even though its substrate specificity is characteristic of BChE [41], but the enzyme has been classified as an AChE on the basis of diagnostic inhibition [39], [40]. However, the ChE in *X. laevis* tadpoles was found to be resistant to various carbamate and organophosphate inhibitors and to not show inhibition by excess substrate [42]. Moreover, the genome project for *X. tropicalis* indicates that only AChE_T_ sequences and not AChE_H_ sequences are present. In striking contrast though, a BChE_H_ sequence is found. The H-peptide is widespread in the AChEs of invertebrates, although these sequences are not homologous among the invertebrates nor with the vertebrates, containing only a few functional similarities: a cysteine near the carboxyl terminal that mediates inter-chain disulfide bonding in the dimer, and a GPI anchor signal consisting of an ω cleavage/attachment site followed by a non-conserved stretch of hydrophobic amino acids [51]. These variant kinetic, pharmacologic, and molecular form data among the atypical BChEs raise questions about their evolution and the evolution of AChE and BChE in the vertebrates.

### The Evolution of AChE and BChE

In terms of substrate and inhibitor specificity and inhibition, it is tempting to speculate that the atypical BChE of *O. latipes* and the other atypical BChEs discussed represent a transitional form of ChE as BChE gradually evolved from an ancestral AChE in the vertebrates subsequent to a gene duplication event early in vertebrate evolution. Assuming Darwinian gradualism, one would expect that if AChE were the ancestral vertebrate ChE, with two Phe residues in its acyl pocket, that upon gene duplication the two residues would be replaced sequentially with an intermediate enzyme having one of the Phe residues and decreased substrate specificity, a property that is consistent with the putative role of BChE in detoxification mechanisms and our data [1], [2], [65]–[67]. Subsequently, under selection pressure, the enzyme would lose its second acyl pocket Phe residue to obtain the substrate specificity of BChE in the higher vertebrates. Likewise, the atypical BChEs show different intermediate patterns of substrate inhibition or lack thereof, which could be interpreted as a transition to the complete loss of substrate inhibition with all substrates, and the acquisition of substrate activation, also a property consistent with the detoxifying role of BChE. While substrate inhibition may be physiologically relevant for the role of AChE in synaptic transmission, it would not be adaptive toxicologically. The issue of inhibitor sensitivity is more complicated. One might expect selective pressures on a detoxifying enzyme to be directed towards increased resistance to inhibitors. While non-aromatic substitutions decrease the sensitivity of the enzyme to some inhibitors, they increase the volume of the catalytic gorge in BChE, literally opening it up to inhibition by bulkier inhibitors that cannot gain access to AChE. Thus, there may be a tradeoff between decreased substrate specificity and substrate inhibition, and decreased inhibitor inhibition with the maximization of all three impossible. By contrast, the larger gorge and increased sensitivity to some inhibitors allows BChE to act as a stoichiometric scavenger of natural and man-made carbamate and organophosphate inhibitors [2]. Clearly, other non-aromatic substitutions in the acyl pocket, in other subsites in the catalytic gorge, and in the peripheral site, also producing kinetic and pharmacological differences between the two enzymes have occurred in this transition as the two enzymes diverged structurally and functionally subsequent to gene duplication [9], [54], [68], [69].

Because of the possibility of three extensive (even genome-wide) gene duplication events early in vertebrate evolution at (1) the origin of the vertebrates, (2) the emergence of the jawed fish, and (3) the appearance of the ray-finned fishes [70]–[72], the timing of the putative gene duplication event producing vertebrate BChE from AChE is uncertain. The jawless vertebrates, the lamprey *P. marinus* and the hagfish *M. glutinosa*, appear to have only one ChE, AChE [24], [73], and there is evidence for two ChEs in the cartilaginous jawed fish *T. marmorata*, AChE and an atypical BChE [17]. Therefore, it is possible that a duplication of an ancestral AChE gene accompanied the wide-spread gene duplication event that coincided with the emergence of the jawed fish, with the atypical and typical BChEs of bony fish and other derived vertebrates descendents of this gene. However, given the genome duplication event in the ray-finned fish lineage, it is possible that these atypical BChEs are unique to the ray-finned fish and not ancestral to the BChE of higher vertebrates, as the land vertebrates descended from the lobe-finned fishes, represented today by the lungfish and the coelacanth, which diverged from the ray-finned fish prior to the duplication event [74]. Currently there is not any information about ChEs from the coelacanth genome project, but such information could shed light on the timing of the gene duplication event and the evolutionary origins of BChE in tetrapods. In any case, for the time being, the atypical BChE of *O. latipes* can serve as a model for the evolutionarily intermediate ChE between AChE and BChE.

 Given that the poorly conserved H-transcripts are widespread in the AChEs of invertebrates, while in vertebrates they are present only in the AChE of the elasmobranch *Torpedo* spp. and mammals, and apparently in a BChE in the amphibian *Xenopus* spp., but not reported in reptiles or birds, Massoulié et al. [26] stated that sequences characteristic of a GPI-addition signal were ”invented” on several occasions during evolution. We can now add the presence of the H-peptide in the atypical BChEs of the teleosts *P. flesus* and *O. latipes*. Combes et al. [75] speculated that the conservation of splice sites at the C-terminus of AChE in insects, nematodes, and vertebrates suggests that exon shuffling has occurred at the 3′end of ChE genes at various times throughout evolution. Such shuffling is one mechanism for appearance of an alternatively spliced exon [76]. Another explanation is exonization [77], [78]; indeed, the presence of read-through or R-transcripts [79]–[81] in AChE could be considered a nascent or abortive exonization event, and the low abundance of such R-transcripts is consistent with an exonization process [78]. Thus, it is possible that the evolution of the C-termini of ChEs is independent of the evolution of the catalytic subunit. Further discussion of the evolution of ChEs can be found in Pezzementi and Chatonnet [82].

### Ecotoxicological Implications

Pesticide use is a major concern in aquatic environments, where runoff from agricultural and urban ecosystems impacts their ecology. The major pesticides in use today are organophosphate and carbamate acetylcholinesterase inhibitors, and pyrethroid ion-channel agents [83], [84]. These toxins are transported from terrestrial to aquatic ecosystems, placing the latter at risk; thus, it is important to understand the effects of these compounds on the resident vertebrate and invertebrate fauna [85]. Inhibition of ChE activity in fish generally correlates with mortality. There may also be sub-lethal behavioral and physiological effects, including reduced swimming ability, and altered feeding and social behavior. However, there are marked species differences in these effects [86]. These differences could be due to different levels of AChE and BChE in the nervous system and the blood, different catalytic abilities of the enzymes, and differential sensitivity of the ChEs to inhibitors, including the more recently used enantioselective organophosphates [48], [50], [87]. The presence of atypical BChEs in some species of fish probably contributes to these species-specific effects, and a better understanding of the kinetics and pharmacology of these atypical enzymes could provide insights into the toxic effects on fish of ChE poisoning.

## Materials and Methods

### Ethics Statement

All animal procedures were conducted in strict adherence to the European Council Directive of November 24, 1986 (86-609). Approval for this study was provided by Comité Régional d'Ethique Languedoc Roussillon C34-172-10.

### Materials

Dulbecco's modified Eagle medium, fetal bovine serum, OptiMEM medium, and phosphatidylinositol-specific phospholipase C (PIPLC) were purchased from Invitrogen, Carlsbad, California. FuGene was obtained from Roche, Indianapolis, Indiana. Acetylthiocholine (ATCh), butyrylthiocholine (BTCh), propionylthiocholine (PTCh), [4-[5-[4-(dimethyl-prop-2-enyl-ammonio)phenyl]-3-oxo-pentyl]phenyl]-dimethyl-prop-2-enyl-azanium dibromide (BW284c51), 5-(3-carboxy-4nitro-phenyl)disulfanyl-2-nitro-benzoic acid (DTNB), N-[bis(propan-2-ylamino)phosphoryloxy-(propan-2-ylamino)phosphoryl]propan-2-amine (iso-OMPA), 10-(2-diethylaminopropyl) phenothiazine hydrochloride (ethopropazine), and (3aS-cis)-1,2,3,3a,8,8a-hexahydro-1,3a,8-trimethylpyrrolo[2,3-b]indol-5-ol methylcarbamate (physostigmine) were purchased from Sigma, St. Louis, Missouri. The high-affinity ligand 7-[(diethoxyphosphoryl)oxy]-1-methylquinolinium iodide (DEPQ) was a gift from Yacov Ashani.

*O. latipes* adults and embryos were from facilities at INRA. Fish were maintained at 28°C on a 13-h light/11-h dark cycle.

### cDNA cloning and sequence analyses

The cDNA clone Ola.23452 (Genbank AV668390) from an *O. latipes* library was obtained from UniGene. The clone was extended by PCR on the basis of the gene structure on scaffold2582 in UCSC genome project, and the full cDNA sequence (Genbank GU797251) was cloned into the expression vector pCMV SPORT 6.1 (Invitrogen).

Sequences were aligned with Clustal W or Clustal X for molecular modeling or phylogenetic analysis by the neighbor-joining method [88]. Putative ω cleavage/attachment sites and downstream stretches of hydrophobic amino acids of H-type C-termini of ChEs were predicted with PredGPI [89] and ProtScale [90], respectively.

### *In vitro* expression and extraction of enzyme

COS-7 monkey cells (American Type Culture Collection) were grown in Dulbecco's modified Eagle medium containing 10% fetal calf serum. Cells were plated at a density of 2.5×10^6^ cells/75 cm^2^ culture flask, incubated overnight, and transferred to OptiMEM medium. FuGene was then used to transfect the cells with 7.8 µg of DNA. The cells were then incubated for 48 h at 37^o^C before the medium was removed and the cells extracted in high ionic strength (HIS) buffer: 10 mM NaHPO_4_, pH 7, 1 M NaCl, 1% Triton X-100, 1 mM EDTA. Extracts were centrifuged at 20,000*g* for 20 min, and the supernatants were assayed for ChE activity.

### Measurement and analysis of BChE activity and inhibition

AChE activity was measured according to the method of Ellman *et al.* [91] as modified by Doctor *et al.* [92] in 100 mM NaHPO_4_, pH 7, 0.3 mM DTNB, 167 mM NaCl, and 258 µM Triton X-100; some BChEs are inhibited by Triton X-100 [65], but the BChE from *O. latipes* is not at the concentrations used in this study (data not shown). ATCh, BTCh, and PTCh were used as substrates at various concentrations; for pharmacological analyses and assays of sucrose gradients, the concentration of ATCh was 1 mM. The kinetic parameters *K_m_*, *K_ss_*, *b*, and *V_max_*, were determined by using SigmaPlot to fit the data to the equation below as described by Radić et al. [13] and Kaplan et al. [9]. *K_ss_* is the dissociation constant for the binding of substrate to a second site on the enzyme, and the parameter *b* indicates the relative catalytic efficiency of the SES complex compared to SE. If b <1, the enzyme shows substrate inhibition; if *b*>1, the enzyme shows substrate activation, and if *b* = 1, Michaelis-Menten kinetics is observed.


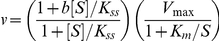


The turnover number *k_cat_* (*V_max_*/[Enzyme]) was determined by enzyme titration with DEPQ [93] as described previously [63]. Values of IC_50_ for the inhibitors used were determined by incubating enzymes with various concentrations of drug for 20 min and then assaying for enzyme activity in the presence of ATCh. SigmaPlot was then used to fit the data to a three-parameter logistic function, yielding IC_50_. Biphasic inhibition curves were fit with GOSAfit. Since we were just looking for classical diagnostic differential inhibition, it was not necessary to determine *k_i_* or *K_I_* values for the inhibitors [4], [24], [52].

### Velocity Sedimentation on Sucrose Gradients; PIPLC Digestion

The molecular forms of ChE were analyzed by velocity sedimentation in 5–25% isokinetic sucrose gradients prepared in HIS buffer (with or without Triton X-100) containing 1 mg/ml bovine serum albumin. Sedimentation was in an SW 41 rotor at 30,000–37,000 rpm for times satisfying the equation [(rpm)^2^×t (h)]  =  2.5×10^10^ as described previously [24]. Apparent sedimentation coefficients were calculated relative to the sedimentation of catalase (11.3 S). Data were plotted as fractional activity of total ChE activity on the gradient as a function of sedimentation coefficient: fractional activity on gradient  =  (activity in a given fraction/total activity on gradient); sedimentation coefficient  =  (fraction number) (11.3S/fraction number of catalase peak). PIPLC digestion was performed by a modification of the method of Gibney et al. [94]. These enzyme assays were performed in Ellman's solution prepared in phosphate-buffered saline.

### Molecular Modeling

Sequence alignment was performed with ClustalX and homology modeling was carried out using MODELLER 9v8 program [95]. MODELLER can implement comparative protein structure modeling by satisfying spatial restraints in terms of probability density functions. A 3D structural model of medaka AChE was built by using the crystal structures of *H. sapiens* BChE (pdb code 1p0i), *T. californica* AChE (pdb code 1ea5) and *D. melanogaster* AChE (pdb code 1dx4) as structural templates. Structural water molecules that are conserved among the three templates were also retained in the modeling procedure. A series of 200 runs of MODELLER were carried out using standard parameters, and the outcomes were ranked on the basis of the internal DOPE scoring function. The model with the highest score was chosen as the candidate model. Then, energy minimization was performed using GROMACS 4.05 according to the software protocol [96]. The final energy-minimized model and the templates were aligned using Theseus [97] and analyzed in PyMOL [98]. Active site gorge surfaces and volumes were calculated using the software HOLLOW 1.1 [99] and taking into account highly conserved structural water molecules. For each structure, the active site entrance forms a bottleneck that was used to delimit the volume of the gorge in the calculations.

## Supporting Information

Figure S1 The pdb dataset for the model of *O. latipes* BChE.
(DOC)
