# Correction: Evolution of Acetylcholinesterase and Butyrylcholinesterase in the Vertebrates: An Atypical Butyrylcholinesterase from the Medaka *Oryzias latipes*

**DOI:** 10.1371/annotation/938a4e59-a5d1-448c-b7c1-632bf9e7e8ef

**Published:** 2011-03-21

**Authors:** Leo Pezzementi, Florian Nachon, Arnaud Chatonnet

The graph for Figure 5 is incorrect and does not match the figure legend. See the correct Figure 5 here: [

**Figure:**
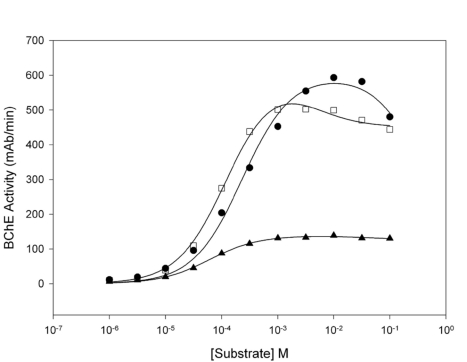


[^] 

