# Supplementary material for: Evolution of Acetylcholinesterase and Butyrylcholinesterase in the Vertebrates: An Atypical Butyrylcholinesterase from the Medaka Oryzias latipes
Source: PLoS One. 2011 Feb 25;6(2):e17396. doi: 10.1371/journal.pone.0017396 (PMC3045457; doi:10.1371/journal.pone.0017396)
Supplement: Figure S1 — The pdb dataset for the model of O. latipes BChE. (DOC) [file pone.0017396.s001.doc]

REMARK Accelrys Discovery Studio PDB file

REMARK Created: Tue Oct 05 07:17:18 Central Daylight Time 2010

SSBOND 1 CYS 64 CYS 91

SSBOND 2 CYS 403 CYS 518

SSBOND 3 CYS 255 CYS 266

ATOM 1 N ASP 1 12.900 49.860 56.460 1.00 0.00 N1+

ATOM 2 CA ASP 1 13.250 49.940 55.030 1.00 0.00 C

ATOM 3 C ASP 1 13.880 48.620 54.580 1.00 0.00 C

ATOM 4 O ASP 1 14.490 47.920 55.400 1.00 0.00 O

ATOM 5 CB ASP 1 14.260 51.080 54.880 1.00 0.00 C

ATOM 6 CG ASP 1 14.830 51.330 53.500 1.00 0.00 C

ATOM 7 OD1 ASP 1 14.130 51.060 52.500 1.00 0.00 O

ATOM 8 OD2 ASP 1 16.000 51.740 53.470 1.00 0.00 O

ATOM 9 H1 ASP 1 12.660 50.760 56.810 1.00 0.00 H

ATOM 10 H2 ASP 1 12.130 49.230 56.600 1.00 0.00 H

ATOM 11 H3 ASP 1 13.690 49.520 56.970 1.00 0.00 H

ATOM 12 N ASP 2 13.810 48.340 53.280 1.00 0.00 N

ATOM 13 CA ASP 2 14.480 47.190 52.630 1.00 0.00 C

ATOM 14 C ASP 2 16.010 47.270 52.770 1.00 0.00 C

ATOM 15 O ASP 2 16.700 46.260 52.940 1.00 0.00 O

ATOM 16 CB ASP 2 14.080 47.150 51.150 1.00 0.00 C

ATOM 17 CG ASP 2 12.600 46.790 50.950 1.00 0.00 C

ATOM 18 OD1 ASP 2 12.320 45.570 50.900 1.00 0.00 O

ATOM 19 OD2 ASP 2 11.770 47.720 50.850 1.00 0.00 O

ATOM 20 H ASP 2 13.430 49.010 52.630 1.00 0.00 H

ATOM 21 N LEU 3 16.510 48.500 52.890 1.00 0.00 N

ATOM 22 CA LEU 3 17.960 48.780 52.970 1.00 0.00 C

ATOM 23 C LEU 3 18.510 48.910 54.400 1.00 0.00 C

ATOM 24 O LEU 3 19.640 49.360 54.590 1.00 0.00 O

ATOM 25 CB LEU 3 18.240 50.030 52.150 1.00 0.00 C

ATOM 26 CG LEU 3 17.910 49.840 50.660 1.00 0.00 C

ATOM 27 CD1 LEU 3 16.470 50.230 50.290 1.00 0.00 C

ATOM 28 CD2 LEU 3 18.880 50.660 49.860 1.00 0.00 C

ATOM 29 H LEU 3 15.950 49.330 52.800 1.00 0.00 H

ATOM 30 N VAL 4 17.860 48.220 55.320 1.00 0.00 N

ATOM 31 CA VAL 4 18.290 48.210 56.740 1.00 0.00 C

ATOM 32 C VAL 4 18.790 46.800 57.080 1.00 0.00 C

ATOM 33 O VAL 4 18.190 45.800 56.710 1.00 0.00 O

ATOM 34 CB VAL 4 17.180 48.690 57.690 1.00 0.00 C

ATOM 35 CG1 VAL 4 17.640 48.680 59.150 1.00 0.00 C

ATOM 36 CG2 VAL 4 16.740 50.120 57.370 1.00 0.00 C

ATOM 37 H VAL 4 17.100 47.620 55.100 1.00 0.00 H

ATOM 38 N ILE 5 20.010 46.810 57.610 1.00 0.00 N

ATOM 39 CA ILE 5 20.650 45.620 58.190 1.00 0.00 C

ATOM 40 C ILE 5 20.960 45.920 59.670 1.00 0.00 C

ATOM 41 O ILE 5 21.400 47.010 60.020 1.00 0.00 O

ATOM 42 CB ILE 5 21.910 45.200 57.390 1.00 0.00 C

ATOM 43 CG1 ILE 5 21.450 44.640 56.040 1.00 0.00 C

ATOM 44 CG2 ILE 5 22.780 44.160 58.120 1.00 0.00 C

ATOM 45 CD ILE 5 22.560 44.410 55.010 1.00 0.00 C

ATOM 46 H ILE 5 20.530 47.660 57.690 1.00 0.00 H

ATOM 47 N ASN 6 20.630 44.940 60.490 1.00 0.00 N

ATOM 48 CA ASN 6 20.980 44.970 61.920 1.00 0.00 C

ATOM 49 C ASN 6 22.300 44.240 62.140 1.00 0.00 C

ATOM 50 O ASN 6 22.450 43.060 61.810 1.00 0.00 O

ATOM 51 CB ASN 6 19.860 44.330 62.740 1.00 0.00 C

ATOM 52 CG ASN 6 19.840 44.840 64.170 1.00 0.00 C

ATOM 53 ND2 ASN 6 18.790 44.510 64.870 1.00 0.00 N

ATOM 54 OD1 ASN 6 20.720 45.530 64.670 1.00 0.00 O

ATOM 55 H ASN 6 20.110 44.130 60.180 1.00 0.00 H

ATOM 56 1HD2 ASN 6 18.080 43.940 64.470 1.00 0.00 H

ATOM 57 2HD2 ASN 6 18.840 44.640 65.860 1.00 0.00 H

ATOM 58 N THR 7 23.300 45.030 62.520 1.00 0.00 N

ATOM 59 CA THR 7 24.630 44.520 62.880 1.00 0.00 C

ATOM 60 C THR 7 24.690 44.360 64.410 1.00 0.00 C

ATOM 61 O THR 7 23.790 44.770 65.130 1.00 0.00 O

ATOM 62 CB THR 7 25.790 45.430 62.400 1.00 0.00 C

ATOM 63 CG2 THR 7 25.690 45.750 60.900 1.00 0.00 C

ATOM 64 OG1 THR 7 25.930 46.630 63.170 1.00 0.00 O

ATOM 65 H THR 7 23.150 46.010 62.700 1.00 0.00 H

ATOM 66 HG1 THR 7 26.640 47.200 62.720 1.00 0.00 H

ATOM 67 N THR 8 25.750 43.720 64.890 1.00 0.00 N

ATOM 68 CA THR 8 26.010 43.540 66.340 1.00 0.00 C

ATOM 69 C THR 8 26.070 44.900 67.060 1.00 0.00 C

ATOM 70 O THR 8 25.680 45.010 68.230 1.00 0.00 O

ATOM 71 CB THR 8 27.320 42.760 66.560 1.00 0.00 C

ATOM 72 CG2 THR 8 27.330 42.060 67.920 1.00 0.00 C

ATOM 73 OG1 THR 8 27.470 41.800 65.520 1.00 0.00 O

ATOM 74 H THR 8 26.420 43.250 64.300 1.00 0.00 H

ATOM 75 HG1 THR 8 28.060 41.000 65.800 1.00 0.00 H

ATOM 76 N HIS 9 26.600 45.900 66.370 1.00 0.00 N

ATOM 77 CA HIS 9 26.680 47.300 66.860 1.00 0.00 C

ATOM 78 C HIS 9 25.340 48.060 66.800 1.00 0.00 C

ATOM 79 O HIS 9 25.130 48.980 67.590 1.00 0.00 O

ATOM 80 CB HIS 9 27.730 48.090 66.090 1.00 0.00 C

ATOM 81 CG HIS 9 29.180 47.680 66.330 1.00 0.00 C

ATOM 82 CD2 HIS 9 29.990 47.130 65.440 1.00 0.00 C

ATOM 83 ND1 HIS 9 29.910 48.000 67.400 1.00 0.00 N

ATOM 84 CE1 HIS 9 31.160 47.630 67.160 1.00 0.00 C

ATOM 85 NE2 HIS 9 31.220 47.100 65.950 1.00 0.00 N

ATOM 86 H HIS 9 26.940 45.760 65.440 1.00 0.00 H

ATOM 87 HE2 HIS 9 32.050 46.770 65.490 1.00 0.00 H

ATOM 88 N GLY 10 24.550 47.780 65.770 1.00 0.00 N

ATOM 89 CA GLY 10 23.190 48.320 65.620 1.00 0.00 C

ATOM 90 C GLY 10 22.710 48.310 64.160 1.00 0.00 C

ATOM 91 O GLY 10 23.310 47.710 63.280 1.00 0.00 O

ATOM 92 H GLY 10 24.850 47.190 65.010 1.00 0.00 H

ATOM 93 N LYS 11 21.690 49.120 63.900 1.00 0.00 N

ATOM 94 CA LYS 11 21.060 49.170 62.570 1.00 0.00 C

ATOM 95 C LYS 11 21.740 50.200 61.640 1.00 0.00 C

ATOM 96 O LYS 11 22.180 51.270 62.060 1.00 0.00 O

ATOM 97 CB LYS 11 19.560 49.440 62.700 1.00 0.00 C

ATOM 98 CG LYS 11 18.860 48.290 63.430 1.00 0.00 C

ATOM 99 CD LYS 11 17.370 48.570 63.690 1.00 0.00 C

ATOM 100 CE LYS 11 16.480 48.210 62.500 1.00 0.00 C

ATOM 101 NZ LYS 11 15.090 48.640 62.750 1.00 0.00 N1+

ATOM 102 H LYS 11 21.270 49.700 64.620 1.00 0.00 H

ATOM 103 HZ1 LYS 11 14.500 48.350 62.000 1.00 0.00 H

ATOM 104 HZ2 LYS 11 14.760 48.240 63.600 1.00 0.00 H

ATOM 105 HZ3 LYS 11 15.070 49.650 62.810 1.00 0.00 H

ATOM 106 N VAL 12 21.960 49.730 60.420 1.00 0.00 N

ATOM 107 CA VAL 12 22.510 50.550 59.310 1.00 0.00 C

ATOM 108 C VAL 12 21.570 50.580 58.100 1.00 0.00 C

ATOM 109 O VAL 12 20.970 49.560 57.740 1.00 0.00 O

ATOM 110 CB VAL 12 23.930 50.100 58.870 1.00 0.00 C

ATOM 111 CG1 VAL 12 24.950 50.400 59.970 1.00 0.00 C

ATOM 112 CG2 VAL 12 24.020 48.630 58.450 1.00 0.00 C

ATOM 113 H VAL 12 21.780 48.770 60.200 1.00 0.00 H

ATOM 114 N GLN 13 21.470 51.760 57.510 1.00 0.00 N

ATOM 115 CA GLN 13 20.690 51.980 56.270 1.00 0.00 C

ATOM 116 C GLN 13 21.650 52.210 55.100 1.00 0.00 C

ATOM 117 O GLN 13 22.500 53.090 55.110 1.00 0.00 O

ATOM 118 CB GLN 13 19.750 53.180 56.420 1.00 0.00 C

ATOM 119 CG GLN 13 18.680 53.190 55.320 1.00 0.00 C

ATOM 120 CD GLN 13 17.820 54.470 55.360 1.00 0.00 C

ATOM 121 NE2 GLN 13 17.310 54.870 54.210 1.00 0.00 N

ATOM 122 OE1 GLN 13 17.440 54.980 56.400 1.00 0.00 O

ATOM 123 H GLN 13 21.860 52.590 57.920 1.00 0.00 H

ATOM 124 1HE2 GLN 13 17.360 54.310 53.370 1.00 0.00 H

ATOM 125 2HE2 GLN 13 16.940 55.790 54.190 1.00 0.00 H

ATOM 126 N GLY 14 21.450 51.370 54.100 1.00 0.00 N

ATOM 127 CA GLY 14 22.180 51.360 52.820 1.00 0.00 C

ATOM 128 C GLY 14 21.370 52.060 51.730 1.00 0.00 C

ATOM 129 O GLY 14 20.250 52.530 51.950 1.00 0.00 O

ATOM 130 H GLY 14 20.710 50.680 54.160 1.00 0.00 H

ATOM 131 N LYS 15 22.050 52.270 50.610 1.00 0.00 N

ATOM 132 CA LYS 15 21.450 52.890 49.410 1.00 0.00 C

ATOM 133 C LYS 15 21.480 51.960 48.190 1.00 0.00 C

ATOM 134 O LYS 15 22.420 51.190 47.990 1.00 0.00 O

ATOM 135 CB LYS 15 22.090 54.240 49.080 1.00 0.00 C

ATOM 136 CG LYS 15 23.600 54.170 48.840 1.00 0.00 C

ATOM 137 CD LYS 15 23.970 55.290 47.880 1.00 0.00 C

ATOM 138 CE LYS 15 25.460 55.300 47.540 1.00 0.00 C

ATOM 139 NZ LYS 15 25.700 56.230 46.430 1.00 0.00 N1+

ATOM 140 H LYS 15 23.040 52.110 50.580 1.00 0.00 H

ATOM 141 HZ1 LYS 15 26.660 56.200 46.130 1.00 0.00 H

ATOM 142 HZ2 LYS 15 25.110 56.010 45.660 1.00 0.00 H

ATOM 143 HZ3 LYS 15 25.470 57.160 46.710 1.00 0.00 H

ATOM 144 N LEU 16 20.400 52.070 47.410 1.00 0.00 N

ATOM 145 CA LEU 16 20.230 51.320 46.150 1.00 0.00 C

ATOM 146 C LEU 16 20.880 52.090 45.000 1.00 0.00 C

ATOM 147 O LEU 16 20.900 53.330 45.000 1.00 0.00 O

ATOM 148 CB LEU 16 18.750 51.080 45.790 1.00 0.00 C

ATOM 149 CG LEU 16 18.020 50.050 46.660 1.00 0.00 C

ATOM 150 CD1 LEU 16 16.550 49.980 46.260 1.00 0.00 C

ATOM 151 CD2 LEU 16 18.650 48.650 46.580 1.00 0.00 C

ATOM 152 H LEU 16 19.700 52.760 47.600 1.00 0.00 H

ATOM 153 N ILE 17 21.690 51.330 44.290 1.00 0.00 N

ATOM 154 CA ILE 17 22.420 51.780 43.090 1.00 0.00 C

ATOM 155 C ILE 17 22.120 50.810 41.950 1.00 0.00 C

ATOM 156 O ILE 17 22.460 49.630 42.030 1.00 0.00 O

ATOM 157 CB ILE 17 23.940 51.850 43.380 1.00 0.00 C

ATOM 158 CG1 ILE 17 24.270 52.730 44.590 1.00 0.00 C

ATOM 159 CG2 ILE 17 24.760 52.260 42.140 1.00 0.00 C

ATOM 160 CD ILE 17 23.900 54.210 44.450 1.00 0.00 C

ATOM 161 H ILE 17 21.840 50.360 44.530 1.00 0.00 H

ATOM 162 N SER 18 21.860 51.440 40.810 1.00 0.00 N

ATOM 163 CA SER 18 21.630 50.760 39.530 1.00 0.00 C

ATOM 164 C SER 18 22.950 50.600 38.760 1.00 0.00 C

ATOM 165 O SER 18 23.690 51.550 38.510 1.00 0.00 O

ATOM 166 CB SER 18 20.600 51.560 38.740 1.00 0.00 C

ATOM 167 OG SER 18 21.120 52.870 38.490 1.00 0.00 O

ATOM 168 H SER 18 21.700 52.420 40.790 1.00 0.00 H

ATOM 169 HG SER 18 20.370 53.500 38.230 1.00 0.00 H

ATOM 170 N VAL 19 23.320 49.340 38.590 1.00 0.00 N

ATOM 171 CA VAL 19 24.580 48.910 37.940 1.00 0.00 C

ATOM 172 C VAL 19 24.270 47.870 36.850 1.00 0.00 C

ATOM 173 O VAL 19 23.700 46.820 37.130 1.00 0.00 O

ATOM 174 CB VAL 19 25.570 48.340 38.990 1.00 0.00 C

ATOM 175 CG1 VAL 19 26.910 47.920 38.370 1.00 0.00 C

ATOM 176 CG2 VAL 19 25.860 49.350 40.100 1.00 0.00 C

ATOM 177 H VAL 19 22.700 48.600 38.860 1.00 0.00 H

ATOM 178 N LEU 20 24.590 48.230 35.600 1.00 0.00 N

ATOM 179 CA LEU 20 24.440 47.350 34.420 1.00 0.00 C

ATOM 180 C LEU 20 23.030 46.730 34.250 1.00 0.00 C

ATOM 181 O LEU 20 22.890 45.550 33.940 1.00 0.00 O

ATOM 182 CB LEU 20 25.510 46.240 34.420 1.00 0.00 C

ATOM 183 CG LEU 20 26.950 46.730 34.240 1.00 0.00 C

ATOM 184 CD1 LEU 20 27.920 45.600 34.560 1.00 0.00 C

ATOM 185 CD2 LEU 20 27.190 47.250 32.820 1.00 0.00 C

ATOM 186 H LEU 20 24.910 49.170 35.400 1.00 0.00 H

ATOM 187 N GLY 21 22.020 47.580 34.430 1.00 0.00 N

ATOM 188 CA GLY 21 20.590 47.180 34.370 1.00 0.00 C

ATOM 189 C GLY 21 20.210 46.090 35.390 1.00 0.00 C

ATOM 190 O GLY 21 19.770 45.000 35.010 1.00 0.00 O

ATOM 191 H GLY 21 22.180 48.570 34.490 1.00 0.00 H

ATOM 192 N GLY 22 20.770 46.290 36.570 1.00 0.00 N

ATOM 193 CA GLY 22 20.510 45.540 37.800 1.00 0.00 C

ATOM 194 C GLY 22 20.800 46.470 38.980 1.00 0.00 C

ATOM 195 O GLY 22 21.230 47.610 38.790 1.00 0.00 O

ATOM 196 H GLY 22 21.430 47.030 36.700 1.00 0.00 H

ATOM 197 N GLU 23 20.720 45.910 40.170 1.00 0.00 N

ATOM 198 CA GLU 23 20.880 46.690 41.410 1.00 0.00 C

ATOM 199 C GLU 23 21.900 46.130 42.400 1.00 0.00 C

ATOM 200 O GLU 23 22.160 44.920 42.460 1.00 0.00 O

ATOM 201 CB GLU 23 19.510 46.860 42.060 1.00 0.00 C

ATOM 202 CG GLU 23 18.690 47.940 41.350 1.00 0.00 C

ATOM 203 CD GLU 23 18.530 49.160 42.250 1.00 0.00 C

ATOM 204 OE1 GLU 23 18.050 48.960 43.390 1.00 0.00 O

ATOM 205 OE2 GLU 23 18.820 50.280 41.780 1.00 0.00 O

ATOM 206 H GLU 23 20.490 44.950 40.300 1.00 0.00 H

ATOM 207 N VAL 24 22.570 47.070 43.060 1.00 0.00 N

ATOM 208 CA VAL 24 23.540 46.820 44.140 1.00 0.00 C

ATOM 209 C VAL 24 23.160 47.730 45.320 1.00 0.00 C

ATOM 210 O VAL 24 22.910 48.920 45.150 1.00 0.00 O

ATOM 211 CB VAL 24 24.990 47.100 43.700 1.00 0.00 C

ATOM 212 CG1 VAL 24 26.010 46.810 44.800 1.00 0.00 C

ATOM 213 CG2 VAL 24 25.380 46.270 42.470 1.00 0.00 C

ATOM 214 H VAL 24 22.420 48.040 42.830 1.00 0.00 H

ATOM 215 N ARG 25 23.270 47.170 46.520 1.00 0.00 N

ATOM 216 CA ARG 25 23.120 47.960 47.750 1.00 0.00 C

ATOM 217 C ARG 25 24.500 48.240 48.340 1.00 0.00 C

ATOM 218 O ARG 25 25.340 47.360 48.510 1.00 0.00 O

ATOM 219 CB ARG 25 22.160 47.260 48.720 1.00 0.00 C

ATOM 220 CG ARG 25 22.080 47.990 50.070 1.00 0.00 C

ATOM 221 CD ARG 25 20.800 47.690 50.850 1.00 0.00 C

ATOM 222 NE ARG 25 20.460 46.260 50.890 1.00 0.00 N

ATOM 223 CZ ARG 25 19.310 45.730 50.430 1.00 0.00 C

ATOM 224 NH1 ARG 25 18.360 46.470 49.860 1.00 0.00 N

ATOM 225 NH2 ARG 25 19.090 44.430 50.630 1.00 0.00 N

ATOM 226 H ARG 25 23.540 46.210 46.650 1.00 0.00 H

ATOM 227 HE ARG 25 21.120 45.650 51.330 1.00 0.00 H

ATOM 228 1HH1 ARG 25 18.490 47.460 49.760 1.00 0.00 H

ATOM 229 2HH1 ARG 25 17.510 46.050 49.540 1.00 0.00 H

ATOM 230 1HH2 ARG 25 19.760 43.880 51.110 1.00 0.00 H

ATOM 231 2HH2 ARG 25 18.240 44.000 50.280 1.00 0.00 H

ATOM 232 N ALA 26 24.680 49.530 48.590 1.00 0.00 N

ATOM 233 CA ALA 26 25.930 50.070 49.140 1.00 0.00 C

ATOM 234 C ALA 26 25.690 50.680 50.520 1.00 0.00 C

ATOM 235 O ALA 26 24.750 51.450 50.730 1.00 0.00 O

ATOM 236 CB ALA 26 26.490 51.130 48.180 1.00 0.00 C

ATOM 237 H ALA 26 23.940 50.190 48.440 1.00 0.00 H

ATOM 238 N PHE 27 26.520 50.230 51.450 1.00 0.00 N

ATOM 239 CA PHE 27 26.590 50.730 52.830 1.00 0.00 C

ATOM 240 C PHE 27 27.960 51.390 53.000 1.00 0.00 C

ATOM 241 O PHE 27 28.990 50.720 53.030 1.00 0.00 O

ATOM 242 CB PHE 27 26.470 49.580 53.840 1.00 0.00 C

ATOM 243 CG PHE 27 25.130 48.860 53.770 1.00 0.00 C

ATOM 244 CD1 PHE 27 24.960 47.820 52.870 1.00 0.00 C

ATOM 245 CD2 PHE 27 24.110 49.210 54.650 1.00 0.00 C

ATOM 246 CE1 PHE 27 23.760 47.140 52.830 1.00 0.00 C

ATOM 247 CE2 PHE 27 22.900 48.530 54.600 1.00 0.00 C

ATOM 248 CZ PHE 27 22.720 47.500 53.690 1.00 0.00 C

ATOM 249 H PHE 27 27.140 49.460 51.240 1.00 0.00 H

ATOM 250 HD1 PHE 27 25.770 47.540 52.200 1.00 0.00 H

ATOM 251 HD2 PHE 27 24.230 50.060 55.320 1.00 0.00 H

ATOM 252 HE1 PHE 27 23.620 46.320 52.120 1.00 0.00 H

ATOM 253 HE2 PHE 27 22.070 48.840 55.240 1.00 0.00 H

ATOM 254 HZ PHE 27 21.760 47.000 53.620 1.00 0.00 H

ATOM 255 N LEU 28 27.950 52.710 52.930 1.00 0.00 N

ATOM 256 CA LEU 28 29.180 53.510 52.950 1.00 0.00 C

ATOM 257 C LEU 28 29.320 54.260 54.280 1.00 0.00 C

ATOM 258 O LEU 28 28.390 54.880 54.780 1.00 0.00 O

ATOM 259 CB LEU 28 29.180 54.520 51.800 1.00 0.00 C

ATOM 260 CG LEU 28 28.870 53.990 50.390 1.00 0.00 C

ATOM 261 CD1 LEU 28 28.870 55.170 49.430 1.00 0.00 C

ATOM 262 CD2 LEU 28 29.860 52.920 49.910 1.00 0.00 C

ATOM 263 H LEU 28 27.090 53.240 52.940 1.00 0.00 H

ATOM 264 N GLY 29 30.500 54.090 54.870 1.00 0.00 N

ATOM 265 CA GLY 29 30.880 54.710 56.150 1.00 0.00 C

ATOM 266 C GLY 29 30.240 54.100 57.410 1.00 0.00 C

ATOM 267 O GLY 29 29.710 54.820 58.260 1.00 0.00 O

ATOM 268 H GLY 29 31.240 53.620 54.380 1.00 0.00 H

ATOM 269 N ILE 30 30.370 52.780 57.550 1.00 0.00 N

ATOM 270 CA ILE 30 30.010 52.090 58.810 1.00 0.00 C

ATOM 271 C ILE 30 31.250 52.090 59.730 1.00 0.00 C

ATOM 272 O ILE 30 32.260 51.470 59.390 1.00 0.00 O

ATOM 273 CB ILE 30 29.510 50.640 58.580 1.00 0.00 C

ATOM 274 CG1 ILE 30 28.400 50.570 57.520 1.00 0.00 C

ATOM 275 CG2 ILE 30 28.990 50.070 59.910 1.00 0.00 C

ATOM 276 CD ILE 30 28.120 49.150 57.030 1.00 0.00 C

ATOM 277 H ILE 30 30.710 52.200 56.800 1.00 0.00 H

ATOM 278 N PRO 31 31.190 52.790 60.880 1.00 0.00 N

ATOM 279 CA PRO 31 32.300 52.840 61.850 1.00 0.00 C

ATOM 280 C PRO 31 32.540 51.480 62.500 1.00 0.00 C

ATOM 281 O PRO 31 31.620 50.850 63.020 1.00 0.00 O

ATOM 282 CB PRO 31 31.870 53.870 62.900 1.00 0.00 C

ATOM 283 CG PRO 31 30.340 53.810 62.840 1.00 0.00 C

ATOM 284 CD PRO 31 30.040 53.580 61.360 1.00 0.00 C

ATOM 285 N TYR 32 33.800 51.060 62.430 1.00 0.00 N

ATOM 286 CA TYR 32 34.210 49.760 63.020 1.00 0.00 C

ATOM 287 C TYR 32 34.910 49.870 64.390 1.00 0.00 C

ATOM 288 O TYR 32 34.850 48.940 65.190 1.00 0.00 O

ATOM 289 CB TYR 32 35.040 48.950 62.010 1.00 0.00 C

ATOM 290 CG TYR 32 36.380 49.570 61.630 1.00 0.00 C

ATOM 291 CD1 TYR 32 36.500 50.400 60.510 1.00 0.00 C

ATOM 292 CD2 TYR 32 37.490 49.320 62.420 1.00 0.00 C

ATOM 293 CE1 TYR 32 37.730 50.960 60.220 1.00 0.00 C

ATOM 294 CE2 TYR 32 38.710 49.890 62.140 1.00 0.00 C

ATOM 295 CZ TYR 32 38.830 50.710 61.040 1.00 0.00 C

ATOM 296 OH TYR 32 40.030 51.250 60.720 1.00 0.00 O

ATOM 297 H TYR 32 34.500 51.560 61.920 1.00 0.00 H

ATOM 298 HD1 TYR 32 35.650 50.570 59.850 1.00 0.00 H

ATOM 299 HD2 TYR 32 37.380 48.640 63.260 1.00 0.00 H

ATOM 300 HE1 TYR 32 37.840 51.600 59.340 1.00 0.00 H

ATOM 301 HE2 TYR 32 39.580 49.670 62.770 1.00 0.00 H

ATOM 302 HH TYR 32 40.680 51.170 61.480 1.00 0.00 H

ATOM 303 N GLY 33 35.490 51.040 64.650 1.00 0.00 N

ATOM 304 CA GLY 33 36.200 51.350 65.900 1.00 0.00 C

ATOM 305 C GLY 33 35.710 52.680 66.500 1.00 0.00 C

ATOM 306 O GLY 33 34.840 53.340 65.950 1.00 0.00 O

ATOM 307 H GLY 33 35.350 51.840 64.060 1.00 0.00 H

ATOM 308 N LYS 34 36.150 52.920 67.740 1.00 0.00 N

ATOM 309 CA LYS 34 36.040 54.260 68.360 1.00 0.00 C

ATOM 310 C LYS 34 36.950 55.230 67.580 1.00 0.00 C

ATOM 311 O LYS 34 38.100 54.860 67.310 1.00 0.00 O

ATOM 312 CB LYS 34 36.500 54.240 69.820 1.00 0.00 C

ATOM 313 CG LYS 34 35.570 53.510 70.790 1.00 0.00 C

ATOM 314 CD LYS 34 36.160 53.600 72.200 1.00 0.00 C

ATOM 315 CE LYS 34 35.460 52.710 73.240 1.00 0.00 C

ATOM 316 NZ LYS 34 36.240 52.650 74.480 1.00 0.00 N1+

ATOM 317 H LYS 34 36.620 52.220 68.280 1.00 0.00 H

ATOM 318 HZ1 LYS 34 35.720 52.210 75.210 1.00 0.00 H

ATOM 319 HZ2 LYS 34 37.080 52.110 74.350 1.00 0.00 H

ATOM 320 HZ3 LYS 34 36.530 53.570 74.760 1.00 0.00 H

ATOM 321 N PRO 35 36.490 56.450 67.270 1.00 0.00 N

ATOM 322 CA PRO 35 37.330 57.500 66.660 1.00 0.00 C

ATOM 323 C PRO 35 38.620 57.700 67.490 1.00 0.00 C

ATOM 324 O PRO 35 38.510 57.980 68.680 1.00 0.00 O

ATOM 325 CB PRO 35 36.460 58.760 66.720 1.00 0.00 C

ATOM 326 CG PRO 35 35.040 58.210 66.580 1.00 0.00 C

ATOM 327 CD PRO 35 35.080 56.880 67.340 1.00 0.00 C

ATOM 328 N PRO 36 39.800 57.370 66.940 1.00 0.00 N

ATOM 329 CA PRO 36 41.070 57.470 67.680 1.00 0.00 C

ATOM 330 C PRO 36 41.490 58.930 67.850 1.00 0.00 C

ATOM 331 O PRO 36 42.340 59.470 67.150 1.00 0.00 O

ATOM 332 CB PRO 36 42.040 56.600 66.880 1.00 0.00 C

ATOM 333 CG PRO 36 41.550 56.740 65.440 1.00 0.00 C

ATOM 334 CD PRO 36 40.020 56.830 65.580 1.00 0.00 C

ATOM 335 N LEU 37 40.810 59.580 68.790 1.00 0.00 N

ATOM 336 CA LEU 37 41.010 61.010 69.090 1.00 0.00 C

ATOM 337 C LEU 37 41.480 61.230 70.540 1.00 0.00 C

ATOM 338 O LEU 37 41.350 60.350 71.390 1.00 0.00 O

ATOM 339 CB LEU 37 39.720 61.810 68.860 1.00 0.00 C

ATOM 340 CG LEU 37 39.070 61.690 67.480 1.00 0.00 C

ATOM 341 CD1 LEU 37 37.950 62.720 67.360 1.00 0.00 C

ATOM 342 CD2 LEU 37 40.040 61.940 66.330 1.00 0.00 C

ATOM 343 H LEU 37 40.040 59.150 69.250 1.00 0.00 H

ATOM 344 N GLY 38 42.190 62.340 70.700 1.00 0.00 N

ATOM 345 CA GLY 38 42.740 62.820 71.980 1.00 0.00 C

ATOM 346 C GLY 38 43.720 61.810 72.570 1.00 0.00 C

ATOM 347 O GLY 38 44.690 61.430 71.910 1.00 0.00 O

ATOM 348 H GLY 38 42.420 62.910 69.900 1.00 0.00 H

ATOM 349 N LYS 39 43.280 61.230 73.680 1.00 0.00 N

ATOM 350 CA LYS 39 44.000 60.160 74.400 1.00 0.00 C

ATOM 351 C LYS 39 44.250 58.910 73.540 1.00 0.00 C

ATOM 352 O LYS 39 45.060 58.060 73.900 1.00 0.00 O

ATOM 353 CB LYS 39 43.240 59.700 75.660 1.00 0.00 C

ATOM 354 CG LYS 39 42.830 60.790 76.660 1.00 0.00 C

ATOM 355 CD LYS 39 43.970 61.630 77.250 1.00 0.00 C

ATOM 356 CE LYS 39 44.990 60.860 78.100 1.00 0.00 C

ATOM 357 NZ LYS 39 44.510 60.570 79.450 1.00 0.00 N1+

ATOM 358 H LYS 39 42.440 61.560 74.130 1.00 0.00 H

ATOM 359 HZ1 LYS 39 45.160 59.960 79.910 1.00 0.00 H

ATOM 360 HZ2 LYS 39 44.420 61.430 79.970 1.00 0.00 H

ATOM 361 HZ3 LYS 39 43.610 60.130 79.420 1.00 0.00 H

ATOM 362 N LEU 40 43.350 58.680 72.580 1.00 0.00 N

ATOM 363 CA LEU 40 43.390 57.520 71.670 1.00 0.00 C

ATOM 364 C LEU 40 44.360 57.630 70.480 1.00 0.00 C

ATOM 365 O LEU 40 44.650 56.610 69.860 1.00 0.00 O

ATOM 366 CB LEU 40 41.990 57.160 71.150 1.00 0.00 C

ATOM 367 CG LEU 40 40.980 56.720 72.220 1.00 0.00 C

ATOM 368 CD1 LEU 40 39.640 56.420 71.550 1.00 0.00 C

ATOM 369 CD2 LEU 40 41.470 55.490 72.990 1.00 0.00 C

ATOM 370 H LEU 40 42.620 59.340 72.380 1.00 0.00 H

ATOM 371 N ARG 41 44.840 58.840 70.170 1.00 0.00 N

ATOM 372 CA ARG 41 45.810 59.010 69.070 1.00 0.00 C

ATOM 373 C ARG 41 47.070 58.160 69.370 1.00 0.00 C

ATOM 374 O ARG 41 47.450 57.970 70.520 1.00 0.00 O

ATOM 375 CB ARG 41 46.200 60.480 68.870 1.00 0.00 C

ATOM 376 CG ARG 41 46.910 60.640 67.520 1.00 0.00 C

ATOM 377 CD ARG 41 47.620 61.970 67.300 1.00 0.00 C

ATOM 378 NE ARG 41 46.690 63.080 67.110 1.00 0.00 N

ATOM 379 CZ ARG 41 46.940 64.370 67.390 1.00 0.00 C

ATOM 380 NH1 ARG 41 48.110 64.770 67.870 1.00 0.00 N

ATOM 381 NH2 ARG 41 46.000 65.280 67.150 1.00 0.00 N

ATOM 382 H ARG 41 44.700 59.640 70.750 1.00 0.00 H

ATOM 383 HE ARG 41 45.810 62.860 66.650 1.00 0.00 H

ATOM 384 1HH1 ARG 41 48.850 64.130 68.060 1.00 0.00 H

ATOM 385 2HH1 ARG 41 48.250 65.750 68.020 1.00 0.00 H

ATOM 386 1HH2 ARG 41 45.160 64.990 66.700 1.00 0.00 H

ATOM 387 2HH2 ARG 41 46.140 66.250 67.370 1.00 0.00 H

ATOM 388 N PHE 42 47.520 57.490 68.310 1.00 0.00 N

ATOM 389 CA PHE 42 48.670 56.560 68.280 1.00 0.00 C

ATOM 390 C PHE 42 48.430 55.190 68.940 1.00 0.00 C

ATOM 391 O PHE 42 49.240 54.280 68.830 1.00 0.00 O

ATOM 392 CB PHE 42 49.940 57.190 68.860 1.00 0.00 C

ATOM 393 CG PHE 42 50.350 58.470 68.140 1.00 0.00 C

ATOM 394 CD1 PHE 42 50.850 58.410 66.840 1.00 0.00 C

ATOM 395 CD2 PHE 42 50.280 59.690 68.800 1.00 0.00 C

ATOM 396 CE1 PHE 42 51.260 59.580 66.210 1.00 0.00 C

ATOM 397 CE2 PHE 42 50.700 60.850 68.170 1.00 0.00 C

ATOM 398 CZ PHE 42 51.180 60.800 66.870 1.00 0.00 C

ATOM 399 H PHE 42 47.060 57.600 67.420 1.00 0.00 H

ATOM 400 HD1 PHE 42 50.930 57.450 66.330 1.00 0.00 H

ATOM 401 HD2 PHE 42 49.860 59.730 69.810 1.00 0.00 H

ATOM 402 HE1 PHE 42 51.710 59.520 65.220 1.00 0.00 H

ATOM 403 HE2 PHE 42 50.650 61.810 68.690 1.00 0.00 H

ATOM 404 HZ PHE 42 51.500 61.710 66.360 1.00 0.00 H

ATOM 405 N ARG 43 47.300 55.050 69.640 1.00 0.00 N

ATOM 406 CA ARG 43 46.880 53.780 70.260 1.00 0.00 C

ATOM 407 C ARG 43 46.250 52.800 69.280 1.00 0.00 C

ATOM 408 O ARG 43 45.750 53.180 68.220 1.00 0.00 O

ATOM 409 CB ARG 43 45.880 54.040 71.380 1.00 0.00 C

ATOM 410 CG ARG 43 46.550 54.710 72.560 1.00 0.00 C

ATOM 411 CD ARG 43 45.510 54.790 73.660 1.00 0.00 C

ATOM 412 NE ARG 43 46.080 55.540 74.780 1.00 0.00 N

ATOM 413 CZ ARG 43 45.390 55.900 75.870 1.00 0.00 C

ATOM 414 NH1 ARG 43 44.080 55.650 75.980 1.00 0.00 N

ATOM 415 NH2 ARG 43 46.030 56.550 76.830 1.00 0.00 N

ATOM 416 H ARG 43 46.710 55.840 69.820 1.00 0.00 H

ATOM 417 HE ARG 43 47.000 55.890 74.640 1.00 0.00 H

ATOM 418 1HH1 ARG 43 43.610 55.190 75.230 1.00 0.00 H

ATOM 419 2HH1 ARG 43 43.580 55.840 76.820 1.00 0.00 H

ATOM 420 1HH2 ARG 43 46.990 56.820 76.720 1.00 0.00 H

ATOM 421 2HH2 ARG 43 45.630 56.600 77.750 1.00 0.00 H

ATOM 422 N ALA 44 46.210 51.530 69.680 1.00 0.00 N

ATOM 423 CA ALA 44 45.470 50.470 68.980 1.00 0.00 C

ATOM 424 C ALA 44 43.980 50.860 68.910 1.00 0.00 C

ATOM 425 O ALA 44 43.480 51.460 69.870 1.00 0.00 O

ATOM 426 CB ALA 44 45.610 49.170 69.770 1.00 0.00 C

ATOM 427 H ALA 44 46.630 51.260 70.560 1.00 0.00 H

ATOM 428 N PRO 45 43.280 50.550 67.810 1.00 0.00 N

ATOM 429 CA PRO 45 41.840 50.840 67.710 1.00 0.00 C

ATOM 430 C PRO 45 41.090 50.140 68.850 1.00 0.00 C

ATOM 431 O PRO 45 41.530 49.110 69.360 1.00 0.00 O

ATOM 432 CB PRO 45 41.420 50.320 66.340 1.00 0.00 C

ATOM 433 CG PRO 45 42.440 49.220 66.050 1.00 0.00 C

ATOM 434 CD PRO 45 43.730 49.780 66.640 1.00 0.00 C

ATOM 435 N GLU 46 40.220 50.940 69.450 1.00 0.00 N

ATOM 436 CA GLU 46 39.270 50.470 70.480 1.00 0.00 C

ATOM 437 C GLU 46 37.970 50.040 69.780 1.00 0.00 C

ATOM 438 O GLU 46 37.540 50.730 68.850 1.00 0.00 O

ATOM 439 CB GLU 46 38.950 51.620 71.430 1.00 0.00 C

ATOM 440 CG GLU 46 40.140 52.110 72.260 1.00 0.00 C

ATOM 441 CD GLU 46 40.180 51.460 73.640 1.00 0.00 C

ATOM 442 OE1 GLU 46 40.980 50.520 73.820 1.00 0.00 O

ATOM 443 OE2 GLU 46 39.520 52.030 74.540 1.00 0.00 O

ATOM 444 H GLU 46 40.230 51.930 69.300 1.00 0.00 H

ATOM 445 N PRO 47 37.310 48.970 70.240 1.00 0.00 N

ATOM 446 CA PRO 47 36.030 48.530 69.660 1.00 0.00 C

ATOM 447 C PRO 47 34.980 49.640 69.840 1.00 0.00 C

ATOM 448 O PRO 47 35.010 50.370 70.830 1.00 0.00 O

ATOM 449 CB PRO 47 35.640 47.290 70.470 1.00 0.00 C

ATOM 450 CG PRO 47 36.980 46.750 70.960 1.00 0.00 C

ATOM 451 CD PRO 47 37.780 48.020 71.270 1.00 0.00 C

ATOM 452 N ALA 48 34.160 49.820 68.810 1.00 0.00 N

ATOM 453 CA ALA 48 33.040 50.780 68.880 1.00 0.00 C

ATOM 454 C ALA 48 31.910 50.240 69.780 1.00 0.00 C

ATOM 455 O ALA 48 31.690 49.030 69.850 1.00 0.00 O

ATOM 456 CB ALA 48 32.500 51.050 67.470 1.00 0.00 C

ATOM 457 H ALA 48 34.230 49.300 67.960 1.00 0.00 H

ATOM 458 N GLU 49 31.380 51.150 70.590 1.00 0.00 N

ATOM 459 CA GLU 49 30.180 50.880 71.400 1.00 0.00 C

ATOM 460 C GLU 49 28.940 50.880 70.490 1.00 0.00 C

ATOM 461 O GLU 49 28.960 51.420 69.380 1.00 0.00 O

ATOM 462 CB GLU 49 30.050 51.920 72.530 1.00 0.00 C

ATOM 463 CG GLU 49 31.170 51.770 73.570 1.00 0.00 C

ATOM 464 CD GLU 49 31.080 52.760 74.730 1.00 0.00 C

ATOM 465 OE1 GLU 49 29.940 53.070 75.140 1.00 0.00 O

ATOM 466 OE2 GLU 49 32.160 53.180 75.220 1.00 0.00 O

ATOM 467 H GLU 49 31.730 52.080 70.670 1.00 0.00 H

ATOM 468 N LYS 50 27.880 50.250 70.990 1.00 0.00 N

ATOM 469 CA LYS 50 26.650 50.060 70.190 1.00 0.00 C

ATOM 470 C LYS 50 25.850 51.360 70.120 1.00 0.00 C

ATOM 471 O LYS 50 26.050 52.270 70.940 1.00 0.00 O

ATOM 472 CB LYS 50 25.780 48.940 70.740 1.00 0.00 C

ATOM 473 CG LYS 50 26.430 47.570 70.550 1.00 0.00 C

ATOM 474 CD LYS 50 25.680 46.520 71.360 1.00 0.00 C

ATOM 475 CE LYS 50 26.500 45.240 71.490 1.00 0.00 C

ATOM 476 NZ LYS 50 27.730 45.440 72.260 1.00 0.00 N1+

ATOM 477 H LYS 50 27.710 50.230 71.980 1.00 0.00 H

ATOM 478 HZ1 LYS 50 28.230 44.570 72.350 1.00 0.00 H

ATOM 479 HZ2 LYS 50 28.320 46.090 71.780 1.00 0.00 H

ATOM 480 HZ3 LYS 50 27.530 45.830 73.160 1.00 0.00 H

ATOM 481 N TRP 51 25.110 51.470 69.030 1.00 0.00 N

ATOM 482 CA TRP 51 24.250 52.630 68.780 1.00 0.00 C

ATOM 483 C TRP 51 22.780 52.240 68.570 1.00 0.00 C

ATOM 484 O TRP 51 22.440 51.250 67.930 1.00 0.00 O

ATOM 485 CB TRP 51 24.750 53.440 67.580 1.00 0.00 C

ATOM 486 CG TRP 51 24.660 52.660 66.260 1.00 0.00 C

ATOM 487 CD1 TRP 51 23.570 52.520 65.510 1.00 0.00 C

ATOM 488 CD2 TRP 51 25.670 51.900 65.700 1.00 0.00 C

ATOM 489 CE2 TRP 51 25.120 51.320 64.570 1.00 0.00 C

ATOM 490 CE3 TRP 51 27.020 51.800 65.980 1.00 0.00 C

ATOM 491 NE1 TRP 51 23.850 51.710 64.490 1.00 0.00 N

ATOM 492 CZ2 TRP 51 25.950 50.640 63.690 1.00 0.00 C

ATOM 493 CZ3 TRP 51 27.860 51.160 65.080 1.00 0.00 C

ATOM 494 CH2 TRP 51 27.320 50.590 63.930 1.00 0.00 C

ATOM 495 H TRP 51 25.000 50.700 68.390 1.00 0.00 H

ATOM 496 HD1 TRP 51 22.580 52.880 65.770 1.00 0.00 H

ATOM 497 HE1 TRP 51 23.190 51.420 63.790 1.00 0.00 H

ATOM 498 HE3 TRP 51 27.420 52.180 66.930 1.00 0.00 H

ATOM 499 HZ2 TRP 51 25.520 50.070 62.870 1.00 0.00 H

ATOM 500 HZ3 TRP 51 28.920 51.080 65.290 1.00 0.00 H

ATOM 501 HH2 TRP 51 27.990 50.270 63.130 1.00 0.00 H

ATOM 502 N GLU 52 21.930 53.090 69.130 1.00 0.00 N

ATOM 503 CA GLU 52 20.480 53.100 68.870 1.00 0.00 C

ATOM 504 C GLU 52 20.200 54.080 67.730 1.00 0.00 C

ATOM 505 O GLU 52 20.740 55.190 67.720 1.00 0.00 O

ATOM 506 CB GLU 52 19.720 53.570 70.120 1.00 0.00 C

ATOM 507 CG GLU 52 19.980 52.770 71.410 1.00 0.00 C

ATOM 508 CD GLU 52 21.400 52.910 71.980 1.00 0.00 C

ATOM 509 OE1 GLU 52 22.050 53.970 71.830 1.00 0.00 O

ATOM 510 OE2 GLU 52 21.870 51.870 72.480 1.00 0.00 O

ATOM 511 H GLU 52 22.190 53.580 69.980 1.00 0.00 H

ATOM 512 N ALA 53 19.110 53.780 67.020 1.00 0.00 N

ATOM 513 CA ALA 53 18.680 54.350 65.730 1.00 0.00 C

ATOM 514 C ALA 53 19.500 53.770 64.570 1.00 0.00 C

ATOM 515 O ALA 53 20.380 52.930 64.750 1.00 0.00 O

ATOM 516 CB ALA 53 18.660 55.890 65.690 1.00 0.00 C

ATOM 517 H ALA 53 18.520 53.000 67.290 1.00 0.00 H

ATOM 518 N VAL 54 19.040 54.080 63.360 1.00 0.00 N

ATOM 519 CA VAL 54 19.720 53.600 62.150 1.00 0.00 C

ATOM 520 C VAL 54 20.820 54.610 61.780 1.00 0.00 C

ATOM 521 O VAL 54 20.610 55.820 61.800 1.00 0.00 O

ATOM 522 CB VAL 54 18.690 53.350 61.040 1.00 0.00 C

ATOM 523 CG1 VAL 54 18.210 54.610 60.320 1.00 0.00 C

ATOM 524 CG2 VAL 54 19.200 52.300 60.050 1.00 0.00 C

ATOM 525 H VAL 54 18.400 54.830 63.220 1.00 0.00 H

ATOM 526 N LYS 55 22.020 54.070 61.580 1.00 0.00 N

ATOM 527 CA LYS 55 23.100 54.850 60.970 1.00 0.00 C

ATOM 528 C LYS 55 22.870 54.910 59.450 1.00 0.00 C

ATOM 529 O LYS 55 22.800 53.880 58.780 1.00 0.00 O

ATOM 530 CB LYS 55 24.480 54.250 61.260 1.00 0.00 C

ATOM 531 CG LYS 55 25.630 55.050 60.630 1.00 0.00 C

ATOM 532 CD LYS 55 25.830 56.440 61.260 1.00 0.00 C

ATOM 533 CE LYS 55 26.960 57.190 60.570 1.00 0.00 C

ATOM 534 NZ LYS 55 27.330 58.420 61.280 1.00 0.00 N1+

ATOM 535 H LYS 55 22.220 53.130 61.850 1.00 0.00 H

ATOM 536 HZ1 LYS 55 28.100 58.860 60.820 1.00 0.00 H

ATOM 537 HZ2 LYS 55 26.560 59.050 61.300 1.00 0.00 H

ATOM 538 HZ3 LYS 55 27.600 58.180 62.220 1.00 0.00 H

ATOM 539 N ASP 56 22.800 56.140 58.970 1.00 0.00 N

ATOM 540 CA ASP 56 22.740 56.420 57.530 1.00 0.00 C

ATOM 541 C ASP 56 24.110 56.240 56.870 1.00 0.00 C

ATOM 542 O ASP 56 24.990 57.100 56.930 1.00 0.00 O

ATOM 543 CB ASP 56 22.190 57.820 57.270 1.00 0.00 C

ATOM 544 CG ASP 56 20.750 58.040 57.740 1.00 0.00 C

ATOM 545 OD1 ASP 56 19.970 57.070 57.710 1.00 0.00 O

ATOM 546 OD2 ASP 56 20.470 59.190 58.150 1.00 0.00 O

ATOM 547 H ASP 56 22.580 56.920 59.550 1.00 0.00 H

ATOM 548 N ALA 57 24.280 55.050 56.300 1.00 0.00 N

ATOM 549 CA ALA 57 25.490 54.660 55.560 1.00 0.00 C

ATOM 550 C ALA 57 25.360 55.020 54.060 1.00 0.00 C

ATOM 551 O ALA 57 25.690 54.250 53.160 1.00 0.00 O

ATOM 552 CB ALA 57 25.730 53.160 55.810 1.00 0.00 C

ATOM 553 H ALA 57 23.540 54.370 56.290 1.00 0.00 H

ATOM 554 N THR 58 24.910 56.260 53.830 1.00 0.00 N

ATOM 555 CA THR 58 24.560 56.760 52.480 1.00 0.00 C

ATOM 556 C THR 58 25.780 57.140 51.640 1.00 0.00 C

ATOM 557 O THR 58 25.810 56.870 50.430 1.00 0.00 O

ATOM 558 CB THR 58 23.560 57.950 52.460 1.00 0.00 C

ATOM 559 CG2 THR 58 22.320 57.720 53.330 1.00 0.00 C

ATOM 560 OG1 THR 58 24.180 59.180 52.850 1.00 0.00 O

ATOM 561 H THR 58 24.800 56.910 54.580 1.00 0.00 H

ATOM 562 HG1 THR 58 23.890 59.410 53.820 1.00 0.00 H

ATOM 563 N LYS 59 26.700 57.850 52.270 1.00 0.00 N

ATOM 564 CA LYS 59 27.900 58.410 51.620 1.00 0.00 C

ATOM 565 C LYS 59 29.200 57.890 52.230 1.00 0.00 C

ATOM 566 O LYS 59 29.220 57.260 53.280 1.00 0.00 O

ATOM 567 CB LYS 59 27.880 59.940 51.630 1.00 0.00 C

ATOM 568 CG LYS 59 28.080 60.580 53.010 1.00 0.00 C

ATOM 569 CD LYS 59 28.470 62.070 52.950 1.00 0.00 C

ATOM 570 CE LYS 59 27.410 62.930 52.270 1.00 0.00 C

ATOM 571 NZ LYS 59 27.840 64.320 52.120 1.00 0.00 N1+

ATOM 572 H LYS 59 26.590 58.110 53.240 1.00 0.00 H

ATOM 573 HZ1 LYS 59 27.170 64.810 51.560 1.00 0.00 H

ATOM 574 HZ2 LYS 59 27.900 64.760 53.020 1.00 0.00 H

ATOM 575 HZ3 LYS 59 28.720 64.350 51.650 1.00 0.00 H

ATOM 576 N PHE 60 30.280 58.130 51.490 1.00 0.00 N

ATOM 577 CA PHE 60 31.640 57.840 51.970 1.00 0.00 C

ATOM 578 C PHE 60 32.030 58.700 53.190 1.00 0.00 C

ATOM 579 O PHE 60 31.720 59.900 53.220 1.00 0.00 O

ATOM 580 CB PHE 60 32.670 58.040 50.870 1.00 0.00 C

ATOM 581 CG PHE 60 32.560 56.950 49.810 1.00 0.00 C

ATOM 582 CD1 PHE 60 32.990 55.650 50.030 1.00 0.00 C

ATOM 583 CD2 PHE 60 32.080 57.320 48.580 1.00 0.00 C

ATOM 584 CE1 PHE 60 32.940 54.730 48.990 1.00 0.00 C

ATOM 585 CE2 PHE 60 32.020 56.410 47.530 1.00 0.00 C

ATOM 586 CZ PHE 60 32.460 55.110 47.740 1.00 0.00 C

ATOM 587 H PHE 60 30.200 58.570 50.590 1.00 0.00 H

ATOM 588 HD1 PHE 60 33.350 55.340 51.020 1.00 0.00 H

ATOM 589 HD2 PHE 60 31.780 58.350 48.450 1.00 0.00 H

ATOM 590 HE1 PHE 60 33.300 53.710 49.150 1.00 0.00 H

ATOM 591 HE2 PHE 60 31.660 56.720 46.550 1.00 0.00 H

ATOM 592 HZ PHE 60 32.460 54.400 46.910 1.00 0.00 H

ATOM 593 N PRO 61 32.750 58.080 54.130 1.00 0.00 N

ATOM 594 CA PRO 61 33.270 58.750 55.330 1.00 0.00 C

ATOM 595 C PRO 61 34.510 59.600 55.020 1.00 0.00 C

ATOM 596 O PRO 61 35.050 59.590 53.910 1.00 0.00 O

ATOM 597 CB PRO 61 33.610 57.590 56.260 1.00 0.00 C

ATOM 598 CG PRO 61 34.120 56.530 55.290 1.00 0.00 C

ATOM 599 CD PRO 61 33.140 56.650 54.140 1.00 0.00 C

ATOM 600 N ASN 62 35.030 60.200 56.090 1.00 0.00 N

ATOM 601 CA ASN 62 36.270 60.990 56.090 1.00 0.00 C

ATOM 602 C ASN 62 37.470 60.070 55.850 1.00 0.00 C

ATOM 603 O ASN 62 37.500 58.910 56.270 1.00 0.00 O

ATOM 604 CB ASN 62 36.420 61.680 57.450 1.00 0.00 C

ATOM 605 CG ASN 62 35.300 62.650 57.840 1.00 0.00 C

ATOM 606 ND2 ASN 62 35.510 63.380 58.910 1.00 0.00 N

ATOM 607 OD1 ASN 62 34.210 62.730 57.280 1.00 0.00 O

ATOM 608 H ASN 62 34.580 60.120 56.990 1.00 0.00 H

ATOM 609 1HD2 ASN 62 36.370 63.300 59.400 1.00 0.00 H

ATOM 610 2HD2 ASN 62 34.740 63.890 59.300 1.00 0.00 H

ATOM 611 N SER 63 38.400 60.570 55.030 1.00 0.00 N

ATOM 612 CA SER 63 39.700 59.920 54.810 1.00 0.00 C

ATOM 613 C SER 63 40.650 60.260 55.970 1.00 0.00 C

ATOM 614 O SER 63 40.590 61.360 56.530 1.00 0.00 O

ATOM 615 CB SER 63 40.350 60.410 53.510 1.00 0.00 C

ATOM 616 OG SER 63 39.640 59.950 52.350 1.00 0.00 O

ATOM 617 H SER 63 38.280 61.470 54.600 1.00 0.00 H

ATOM 618 HG SER 63 39.960 60.500 51.580 1.00 0.00 H

ATOM 619 N CYS 64 41.540 59.320 56.290 1.00 0.00 N

ATOM 620 CA CYS 64 42.540 59.530 57.360 1.00 0.00 C

ATOM 621 C CYS 64 43.530 60.640 56.990 1.00 0.00 C

ATOM 622 O CYS 64 43.910 60.780 55.830 1.00 0.00 O

ATOM 623 CB CYS 64 43.300 58.230 57.640 1.00 0.00 C

ATOM 624 SG CYS 64 42.250 56.860 58.270 1.00 0.00 S

ATOM 625 H CYS 64 41.600 58.450 55.780 1.00 0.00 H

ATOM 626 N TYR 65 43.950 61.410 58.000 1.00 0.00 N

ATOM 627 CA TYR 65 44.980 62.460 57.800 1.00 0.00 C

ATOM 628 C TYR 65 46.240 61.940 57.110 1.00 0.00 C

ATOM 629 O TYR 65 46.870 60.960 57.530 1.00 0.00 O

ATOM 630 CB TYR 65 45.440 63.170 59.080 1.00 0.00 C

ATOM 631 CG TYR 65 44.540 64.360 59.420 1.00 0.00 C

ATOM 632 CD1 TYR 65 43.440 64.130 60.210 1.00 0.00 C

ATOM 633 CD2 TYR 65 44.820 65.650 58.980 1.00 0.00 C

ATOM 634 CE1 TYR 65 42.610 65.190 60.550 1.00 0.00 C

ATOM 635 CE2 TYR 65 43.990 66.710 59.330 1.00 0.00 C

ATOM 636 CZ TYR 65 42.870 66.480 60.120 1.00 0.00 C

ATOM 637 OH TYR 65 42.090 67.480 60.600 1.00 0.00 O

ATOM 638 H TYR 65 43.490 61.380 58.890 1.00 0.00 H

ATOM 639 HD1 TYR 65 43.240 63.130 60.580 1.00 0.00 H

ATOM 640 HD2 TYR 65 45.730 65.850 58.410 1.00 0.00 H

ATOM 641 HE1 TYR 65 41.670 64.970 61.030 1.00 0.00 H

ATOM 642 HE2 TYR 65 44.230 67.720 58.990 1.00 0.00 H

ATOM 643 HH TYR 65 42.140 68.260 59.960 1.00 0.00 H

ATOM 644 N GLN 66 46.550 62.660 56.040 1.00 0.00 N

ATOM 645 CA GLN 66 47.650 62.360 55.110 1.00 0.00 C

ATOM 646 C GLN 66 48.000 63.590 54.260 1.00 0.00 C

ATOM 647 O GLN 66 47.240 64.560 54.250 1.00 0.00 O

ATOM 648 CB GLN 66 47.250 61.200 54.190 1.00 0.00 C

ATOM 649 CG GLN 66 46.050 61.530 53.310 1.00 0.00 C

ATOM 650 CD GLN 66 45.530 60.270 52.620 1.00 0.00 C

ATOM 651 NE2 GLN 66 44.580 59.620 53.260 1.00 0.00 N

ATOM 652 OE1 GLN 66 45.940 59.890 51.540 1.00 0.00 O

ATOM 653 H GLN 66 45.950 63.400 55.740 1.00 0.00 H

ATOM 654 1HE2 GLN 66 44.230 59.970 54.130 1.00 0.00 H

ATOM 655 2HE2 GLN 66 44.290 58.740 52.880 1.00 0.00 H

ATOM 656 N MET 67 49.260 63.640 53.870 1.00 0.00 N

ATOM 657 CA MET 67 49.770 64.610 52.870 1.00 0.00 C

ATOM 658 C MET 67 49.350 64.210 51.440 1.00 0.00 C

ATOM 659 O MET 67 49.440 63.030 51.110 1.00 0.00 O

ATOM 660 CB MET 67 51.290 64.710 53.000 1.00 0.00 C

ATOM 661 CG MET 67 52.060 63.440 52.610 1.00 0.00 C

ATOM 662 SD MET 67 52.390 63.240 50.810 1.00 0.00 S

ATOM 663 CE MET 67 53.360 64.690 50.470 1.00 0.00 C

ATOM 664 H MET 67 49.930 62.990 54.250 1.00 0.00 H

ATOM 665 N PRO 68 48.640 65.090 50.740 1.00 0.00 N

ATOM 666 CA PRO 68 48.430 64.910 49.290 1.00 0.00 C

ATOM 667 C PRO 68 49.680 65.090 48.410 1.00 0.00 C

ATOM 668 O PRO 68 50.670 65.720 48.780 1.00 0.00 O

ATOM 669 CB PRO 68 47.370 65.950 48.940 1.00 0.00 C

ATOM 670 CG PRO 68 46.510 65.940 50.200 1.00 0.00 C

ATOM 671 CD PRO 68 47.550 65.900 51.310 1.00 0.00 C

ATOM 672 N ASP 69 49.590 64.460 47.240 1.00 0.00 N

ATOM 673 CA ASP 69 50.530 64.640 46.120 1.00 0.00 C

ATOM 674 C ASP 69 50.190 65.970 45.430 1.00 0.00 C

ATOM 675 O ASP 69 49.090 66.140 44.910 1.00 0.00 O

ATOM 676 CB ASP 69 50.410 63.460 45.140 1.00 0.00 C

ATOM 677 CG ASP 69 51.450 63.440 44.000 1.00 0.00 C

ATOM 678 OD1 ASP 69 51.940 64.520 43.610 1.00 0.00 O

ATOM 679 OD2 ASP 69 51.750 62.330 43.510 1.00 0.00 O

ATOM 680 H ASP 69 48.760 63.940 47.000 1.00 0.00 H

ATOM 681 N THR 70 51.040 66.950 45.710 1.00 0.00 N

ATOM 682 CA THR 70 50.930 68.300 45.100 1.00 0.00 C

ATOM 683 C THR 70 51.750 68.440 43.800 1.00 0.00 C

ATOM 684 O THR 70 51.740 69.500 43.170 1.00 0.00 O

ATOM 685 CB THR 70 51.330 69.390 46.110 1.00 0.00 C

ATOM 686 CG2 THR 70 52.840 69.560 46.300 1.00 0.00 C

ATOM 687 OG1 THR 70 50.750 70.640 45.710 1.00 0.00 O

ATOM 688 H THR 70 51.800 66.820 46.350 1.00 0.00 H

ATOM 689 HG1 THR 70 51.250 71.410 46.140 1.00 0.00 H

ATOM 690 N ALA 71 52.480 67.400 43.430 1.00 0.00 N

ATOM 691 CA ALA 71 53.520 67.490 42.380 1.00 0.00 C

ATOM 692 C ALA 71 52.980 67.960 41.020 1.00 0.00 C

ATOM 693 O ALA 71 53.540 68.890 40.440 1.00 0.00 O

ATOM 694 CB ALA 71 54.200 66.130 42.260 1.00 0.00 C

ATOM 695 H ALA 71 52.240 66.450 43.690 1.00 0.00 H

ATOM 696 N PHE 72 51.800 67.490 40.640 1.00 0.00 N

ATOM 697 CA PHE 72 51.130 67.880 39.380 1.00 0.00 C

ATOM 698 C PHE 72 49.680 68.350 39.670 1.00 0.00 C

ATOM 699 O PHE 72 48.730 67.580 39.530 1.00 0.00 O

ATOM 700 CB PHE 72 51.120 66.710 38.390 1.00 0.00 C

ATOM 701 CG PHE 72 52.450 65.970 38.310 1.00 0.00 C

ATOM 702 CD1 PHE 72 53.540 66.550 37.680 1.00 0.00 C

ATOM 703 CD2 PHE 72 52.600 64.780 39.010 1.00 0.00 C

ATOM 704 CE1 PHE 72 54.780 65.960 37.780 1.00 0.00 C

ATOM 705 CE2 PHE 72 53.830 64.150 39.060 1.00 0.00 C

ATOM 706 CZ PHE 72 54.930 64.740 38.440 1.00 0.00 C

ATOM 707 H PHE 72 51.230 66.920 41.270 1.00 0.00 H

ATOM 708 HD1 PHE 72 53.400 67.380 36.990 1.00 0.00 H

ATOM 709 HD2 PHE 72 51.790 64.410 39.640 1.00 0.00 H

ATOM 710 HE1 PHE 72 55.660 66.470 37.390 1.00 0.00 H

ATOM 711 HE2 PHE 72 53.930 63.180 39.560 1.00 0.00 H

ATOM 712 HZ PHE 72 55.890 64.240 38.430 1.00 0.00 H

ATOM 713 N PRO 73 49.510 69.600 40.130 1.00 0.00 N

ATOM 714 CA PRO 73 48.180 70.170 40.430 1.00 0.00 C

ATOM 715 C PRO 73 47.380 70.250 39.120 1.00 0.00 C

ATOM 716 O PRO 73 47.960 70.520 38.070 1.00 0.00 O

ATOM 717 CB PRO 73 48.490 71.570 40.960 1.00 0.00 C

ATOM 718 CG PRO 73 49.910 71.470 41.510 1.00 0.00 C

ATOM 719 CD PRO 73 50.580 70.560 40.490 1.00 0.00 C

ATOM 720 N GLY 74 46.130 69.780 39.170 1.00 0.00 N

ATOM 721 CA GLY 74 45.230 69.720 38.000 1.00 0.00 C

ATOM 722 C GLY 74 45.600 68.670 36.940 1.00 0.00 C

ATOM 723 O GLY 74 45.280 68.810 35.760 1.00 0.00 O

ATOM 724 H GLY 74 45.690 69.460 40.020 1.00 0.00 H

ATOM 725 N PHE 75 46.360 67.670 37.370 1.00 0.00 N

ATOM 726 CA PHE 75 46.710 66.520 36.530 1.00 0.00 C

ATOM 727 C PHE 75 46.040 65.240 37.050 1.00 0.00 C

ATOM 728 O PHE 75 46.450 64.650 38.050 1.00 0.00 O

ATOM 729 CB PHE 75 48.230 66.340 36.440 1.00 0.00 C

ATOM 730 CG PHE 75 48.640 65.190 35.520 1.00 0.00 C

ATOM 731 CD1 PHE 75 48.430 65.290 34.150 1.00 0.00 C

ATOM 732 CD2 PHE 75 49.200 64.040 36.060 1.00 0.00 C

ATOM 733 CE1 PHE 75 48.760 64.220 33.320 1.00 0.00 C

ATOM 734 CE2 PHE 75 49.520 62.980 35.220 1.00 0.00 C

ATOM 735 CZ PHE 75 49.300 63.060 33.860 1.00 0.00 C

ATOM 736 H PHE 75 46.770 67.690 38.280 1.00 0.00 H

ATOM 737 HD1 PHE 75 48.010 66.200 33.720 1.00 0.00 H

ATOM 738 HD2 PHE 75 49.470 64.010 37.110 1.00 0.00 H

ATOM 739 HE1 PHE 75 48.600 64.300 32.240 1.00 0.00 H

ATOM 740 HE2 PHE 75 49.980 62.090 35.640 1.00 0.00 H

ATOM 741 HZ PHE 75 49.530 62.220 33.210 1.00 0.00 H

ATOM 742 N GLN 76 45.200 64.720 36.160 1.00 0.00 N

ATOM 743 CA GLN 76 44.380 63.520 36.400 1.00 0.00 C

ATOM 744 C GLN 76 45.140 62.280 36.870 1.00 0.00 C

ATOM 745 O GLN 76 44.700 61.660 37.830 1.00 0.00 O

ATOM 746 CB GLN 76 43.550 63.160 35.170 1.00 0.00 C

ATOM 747 CG GLN 76 42.340 64.090 35.020 1.00 0.00 C

ATOM 748 CD GLN 76 41.250 63.970 36.100 1.00 0.00 C

ATOM 749 NE2 GLN 76 41.450 63.220 37.160 1.00 0.00 N

ATOM 750 OE1 GLN 76 40.170 64.540 35.960 1.00 0.00 O

ATOM 751 H GLN 76 45.030 65.200 35.280 1.00 0.00 H

ATOM 752 1HE2 GLN 76 42.310 62.740 37.310 1.00 0.00 H

ATOM 753 2HE2 GLN 76 40.650 63.010 37.750 1.00 0.00 H

ATOM 754 N GLY 77 46.330 62.080 36.300 1.00 0.00 N

ATOM 755 CA GLY 77 47.200 60.920 36.590 1.00 0.00 C

ATOM 756 C GLY 77 47.650 60.860 38.060 1.00 0.00 C

ATOM 757 O GLY 77 47.720 59.770 38.630 1.00 0.00 O

ATOM 758 H GLY 77 46.730 62.760 35.680 1.00 0.00 H

ATOM 759 N ALA 78 47.970 62.020 38.620 1.00 0.00 N

ATOM 760 CA ALA 78 48.300 62.160 40.050 1.00 0.00 C

ATOM 761 C ALA 78 47.050 62.370 40.930 1.00 0.00 C

ATOM 762 O ALA 78 46.700 61.480 41.710 1.00 0.00 O

ATOM 763 CB ALA 78 49.300 63.310 40.250 1.00 0.00 C

ATOM 764 H ALA 78 48.020 62.860 38.090 1.00 0.00 H

ATOM 765 N GLU 79 46.220 63.360 40.580 1.00 0.00 N

ATOM 766 CA GLU 79 45.050 63.760 41.390 1.00 0.00 C

ATOM 767 C GLU 79 44.040 62.640 41.700 1.00 0.00 C

ATOM 768 O GLU 79 43.530 62.590 42.820 1.00 0.00 O

ATOM 769 CB GLU 79 44.310 64.950 40.790 1.00 0.00 C

ATOM 770 CG GLU 79 45.090 66.250 40.980 1.00 0.00 C

ATOM 771 CD GLU 79 44.270 67.510 40.660 1.00 0.00 C

ATOM 772 OE1 GLU 79 43.380 67.450 39.790 1.00 0.00 O

ATOM 773 OE2 GLU 79 44.570 68.550 41.280 1.00 0.00 O

ATOM 774 H GLU 79 46.350 63.880 39.730 1.00 0.00 H

ATOM 775 N MET 80 43.910 61.680 40.790 1.00 0.00 N

ATOM 776 CA MET 80 43.070 60.480 40.980 1.00 0.00 C

ATOM 777 C MET 80 43.330 59.730 42.300 1.00 0.00 C

ATOM 778 O MET 80 42.390 59.280 42.940 1.00 0.00 O

ATOM 779 CB MET 80 43.190 59.530 39.780 1.00 0.00 C

ATOM 780 CG MET 80 44.620 59.050 39.500 1.00 0.00 C

ATOM 781 SD MET 80 44.860 58.290 37.850 1.00 0.00 S

ATOM 782 CE MET 80 44.390 56.620 38.200 1.00 0.00 C

ATOM 783 H MET 80 44.390 61.720 39.910 1.00 0.00 H

ATOM 784 N TRP 81 44.600 59.690 42.710 1.00 0.00 N

ATOM 785 CA TRP 81 45.070 59.030 43.940 1.00 0.00 C

ATOM 786 C TRP 81 44.800 59.830 45.230 1.00 0.00 C

ATOM 787 O TRP 81 44.460 59.260 46.270 1.00 0.00 O

ATOM 788 CB TRP 81 46.560 58.710 43.790 1.00 0.00 C

ATOM 789 CG TRP 81 46.840 57.790 42.590 1.00 0.00 C

ATOM 790 CD1 TRP 81 47.380 58.130 41.420 1.00 0.00 C

ATOM 791 CD2 TRP 81 46.530 56.450 42.510 1.00 0.00 C

ATOM 792 CE2 TRP 81 46.910 56.010 41.250 1.00 0.00 C

ATOM 793 CE3 TRP 81 45.910 55.580 43.400 1.00 0.00 C

ATOM 794 NE1 TRP 81 47.420 57.070 40.620 1.00 0.00 N

ATOM 795 CZ2 TRP 81 46.700 54.700 40.880 1.00 0.00 C

ATOM 796 CZ3 TRP 81 45.700 54.260 43.040 1.00 0.00 C

ATOM 797 CH2 TRP 81 46.100 53.820 41.780 1.00 0.00 C

ATOM 798 H TRP 81 45.340 60.110 42.180 1.00 0.00 H

ATOM 799 HD1 TRP 81 47.720 59.130 41.150 1.00 0.00 H

ATOM 800 HE1 TRP 81 47.730 57.080 39.670 1.00 0.00 H

ATOM 801 HE3 TRP 81 45.440 55.980 44.300 1.00 0.00 H

ATOM 802 HZ2 TRP 81 46.970 54.340 39.890 1.00 0.00 H

ATOM 803 HZ3 TRP 81 45.150 53.590 43.690 1.00 0.00 H

ATOM 804 HH2 TRP 81 45.960 52.770 41.500 1.00 0.00 H

ATOM 805 N ASN 82 44.810 61.150 45.120 1.00 0.00 N

ATOM 806 CA ASN 82 44.550 62.080 46.240 1.00 0.00 C

ATOM 807 C ASN 82 43.150 61.870 46.850 1.00 0.00 C

ATOM 808 O ASN 82 42.240 61.460 46.130 1.00 0.00 O

ATOM 809 CB ASN 82 44.720 63.510 45.740 1.00 0.00 C

ATOM 810 CG ASN 82 46.180 63.900 45.490 1.00 0.00 C

ATOM 811 ND2 ASN 82 46.430 64.600 44.400 1.00 0.00 N

ATOM 812 OD1 ASN 82 47.080 63.640 46.280 1.00 0.00 O

ATOM 813 H ASN 82 44.840 61.590 44.210 1.00 0.00 H

ATOM 814 1HD2 ASN 82 45.690 64.820 43.760 1.00 0.00 H

ATOM 815 2HD2 ASN 82 47.350 64.970 44.300 1.00 0.00 H

ATOM 816 N PRO 83 42.970 62.020 48.170 1.00 0.00 N

ATOM 817 CA PRO 83 41.680 61.740 48.840 1.00 0.00 C

ATOM 818 C PRO 83 40.550 62.630 48.330 1.00 0.00 C

ATOM 819 O PRO 83 40.630 63.860 48.360 1.00 0.00 O

ATOM 820 CB PRO 83 41.930 61.990 50.320 1.00 0.00 C

ATOM 821 CG PRO 83 43.410 61.690 50.460 1.00 0.00 C

ATOM 822 CD PRO 83 44.020 62.240 49.180 1.00 0.00 C

ATOM 823 N ASN 84 39.440 61.980 48.010 1.00 0.00 N

ATOM 824 CA ASN 84 38.190 62.640 47.590 1.00 0.00 C

ATOM 825 C ASN 84 37.280 63.160 48.710 1.00 0.00 C

ATOM 826 O ASN 84 36.520 64.100 48.500 1.00 0.00 O

ATOM 827 CB ASN 84 37.420 61.730 46.620 1.00 0.00 C

ATOM 828 CG ASN 84 37.150 60.340 47.170 1.00 0.00 C

ATOM 829 ND2 ASN 84 35.890 60.010 47.340 1.00 0.00 N

ATOM 830 OD1 ASN 84 38.050 59.580 47.490 1.00 0.00 O

ATOM 831 H ASN 84 39.460 60.990 47.820 1.00 0.00 H

ATOM 832 1HD2 ASN 84 35.170 60.660 47.090 1.00 0.00 H

ATOM 833 2HD2 ASN 84 35.710 59.030 47.450 1.00 0.00 H

ATOM 834 N THR 85 37.430 62.630 49.930 1.00 0.00 N

ATOM 835 CA THR 85 36.630 63.070 51.090 1.00 0.00 C

ATOM 836 C THR 85 37.520 63.800 52.120 1.00 0.00 C

ATOM 837 O THR 85 38.720 63.540 52.170 1.00 0.00 O

ATOM 838 CB THR 85 35.880 61.920 51.790 1.00 0.00 C

ATOM 839 CG2 THR 85 34.810 61.310 50.880 1.00 0.00 C

ATOM 840 OG1 THR 85 36.790 60.920 52.260 1.00 0.00 O

ATOM 841 H THR 85 38.180 62.010 50.150 1.00 0.00 H

ATOM 842 HG1 THR 85 36.290 60.340 52.930 1.00 0.00 H

ATOM 843 N PRO 86 36.960 64.760 52.880 1.00 0.00 N

ATOM 844 CA PRO 86 37.670 65.490 53.940 1.00 0.00 C

ATOM 845 C PRO 86 38.500 64.610 54.870 1.00 0.00 C

ATOM 846 O PRO 86 38.100 63.510 55.240 1.00 0.00 O

ATOM 847 CB PRO 86 36.570 66.210 54.710 1.00 0.00 C

ATOM 848 CG PRO 86 35.650 66.630 53.570 1.00 0.00 C

ATOM 849 CD PRO 86 35.650 65.410 52.640 1.00 0.00 C

ATOM 850 N LEU 87 39.710 65.110 55.110 1.00 0.00 N

ATOM 851 CA LEU 87 40.630 64.480 56.070 1.00 0.00 C

ATOM 852 C LEU 87 40.170 64.700 57.510 1.00 0.00 C

ATOM 853 O LEU 87 39.690 65.780 57.890 1.00 0.00 O

ATOM 854 CB LEU 87 42.060 65.010 55.920 1.00 0.00 C

ATOM 855 CG LEU 87 42.710 64.730 54.560 1.00 0.00 C

ATOM 856 CD1 LEU 87 44.140 65.260 54.590 1.00 0.00 C

ATOM 857 CD2 LEU 87 42.710 63.240 54.200 1.00 0.00 C

ATOM 858 H LEU 87 40.070 65.910 54.640 1.00 0.00 H

ATOM 859 N SER 88 40.240 63.600 58.240 1.00 0.00 N

ATOM 860 CA SER 88 39.960 63.550 59.680 1.00 0.00 C

ATOM 861 C SER 88 40.630 62.340 60.320 1.00 0.00 C

ATOM 862 O SER 88 41.020 61.390 59.640 1.00 0.00 O

ATOM 863 CB SER 88 38.450 63.470 59.930 1.00 0.00 C

ATOM 864 OG SER 88 38.150 63.960 61.230 1.00 0.00 O

ATOM 865 H SER 88 40.430 62.710 57.800 1.00 0.00 H

ATOM 866 HG SER 88 37.390 63.430 61.630 1.00 0.00 H

ATOM 867 N GLU 89 40.990 62.530 61.590 1.00 0.00 N

ATOM 868 CA GLU 89 41.480 61.450 62.450 1.00 0.00 C

ATOM 869 C GLU 89 40.340 60.480 62.800 1.00 0.00 C

ATOM 870 O GLU 89 40.570 59.280 62.970 1.00 0.00 O

ATOM 871 CB GLU 89 42.170 62.010 63.700 1.00 0.00 C

ATOM 872 CG GLU 89 43.530 62.660 63.430 1.00 0.00 C

ATOM 873 CD GLU 89 44.200 63.230 64.690 1.00 0.00 C

ATOM 874 OE1 GLU 89 44.070 62.640 65.780 1.00 0.00 O

ATOM 875 OE2 GLU 89 44.870 64.270 64.540 1.00 0.00 O

ATOM 876 H GLU 89 40.860 63.410 62.060 1.00 0.00 H

ATOM 877 N ASP 90 39.110 61.000 62.840 1.00 0.00 N

ATOM 878 CA ASP 90 37.880 60.190 62.820 1.00 0.00 C

ATOM 879 C ASP 90 37.620 59.720 61.380 1.00 0.00 C

ATOM 880 O ASP 90 37.090 60.450 60.540 1.00 0.00 O

ATOM 881 CB ASP 90 36.720 61.030 63.380 1.00 0.00 C

ATOM 882 CG ASP 90 35.390 60.270 63.540 1.00 0.00 C

ATOM 883 OD1 ASP 90 35.400 59.030 63.630 1.00 0.00 O

ATOM 884 OD2 ASP 90 34.380 60.970 63.730 1.00 0.00 O

ATOM 885 H ASP 90 38.950 61.990 62.910 1.00 0.00 H

ATOM 886 N CYS 91 38.390 58.680 61.060 1.00 0.00 N

ATOM 887 CA CYS 91 38.380 58.020 59.740 1.00 0.00 C

ATOM 888 C CYS 91 38.140 56.500 59.730 1.00 0.00 C

ATOM 889 O CYS 91 38.010 55.920 58.650 1.00 0.00 O

ATOM 890 CB CYS 91 39.700 58.330 59.010 1.00 0.00 C

ATOM 891 SG CYS 91 41.210 57.680 59.820 1.00 0.00 S

ATOM 892 H CYS 91 39.120 58.400 61.680 1.00 0.00 H

ATOM 893 N LEU 92 37.990 55.880 60.900 1.00 0.00 N

ATOM 894 CA LEU 92 37.880 54.410 61.020 1.00 0.00 C

ATOM 895 C LEU 92 36.480 53.860 60.650 1.00 0.00 C

ATOM 896 O LEU 92 35.660 53.450 61.470 1.00 0.00 O

ATOM 897 CB LEU 92 38.330 53.880 62.390 1.00 0.00 C

ATOM 898 CG LEU 92 39.750 54.250 62.870 1.00 0.00 C

ATOM 899 CD1 LEU 92 40.090 53.350 64.060 1.00 0.00 C

ATOM 900 CD2 LEU 92 40.840 54.150 61.790 1.00 0.00 C

ATOM 901 H LEU 92 37.850 56.380 61.770 1.00 0.00 H

ATOM 902 N TYR 93 36.300 53.810 59.340 1.00 0.00 N

ATOM 903 CA TYR 93 35.050 53.350 58.710 1.00 0.00 C

ATOM 904 C TYR 93 35.310 52.360 57.560 1.00 0.00 C

ATOM 905 O TYR 93 36.390 52.320 56.970 1.00 0.00 O

ATOM 906 CB TYR 93 34.280 54.550 58.140 1.00 0.00 C

ATOM 907 CG TYR 93 33.910 55.620 59.170 1.00 0.00 C

ATOM 908 CD1 TYR 93 34.800 56.650 59.460 1.00 0.00 C

ATOM 909 CD2 TYR 93 32.680 55.560 59.810 1.00 0.00 C

ATOM 910 CE1 TYR 93 34.460 57.610 60.410 1.00 0.00 C

ATOM 911 CE2 TYR 93 32.350 56.510 60.750 1.00 0.00 C

ATOM 912 CZ TYR 93 33.230 57.530 61.050 1.00 0.00 C

ATOM 913 OH TYR 93 32.780 58.510 61.870 1.00 0.00 O

ATOM 914 H TYR 93 36.990 54.180 58.720 1.00 0.00 H

ATOM 915 HD1 TYR 93 35.680 56.790 58.840 1.00 0.00 H

ATOM 916 HD2 TYR 93 31.930 54.860 59.470 1.00 0.00 H

ATOM 917 HE1 TYR 93 35.180 58.390 60.670 1.00 0.00 H

ATOM 918 HE2 TYR 93 31.400 56.440 61.280 1.00 0.00 H

ATOM 919 HH TYR 93 32.450 58.060 62.720 1.00 0.00 H

ATOM 920 N LEU 94 34.290 51.550 57.330 1.00 0.00 N

ATOM 921 CA LEU 94 34.240 50.590 56.210 1.00 0.00 C

ATOM 922 C LEU 94 33.000 50.760 55.320 1.00 0.00 C

ATOM 923 O LEU 94 31.990 51.340 55.720 1.00 0.00 O

ATOM 924 CB LEU 94 34.440 49.150 56.710 1.00 0.00 C

ATOM 925 CG LEU 94 33.480 48.640 57.800 1.00 0.00 C

ATOM 926 CD1 LEU 94 32.110 48.260 57.260 1.00 0.00 C

ATOM 927 CD2 LEU 94 34.130 47.440 58.490 1.00 0.00 C

ATOM 928 H LEU 94 33.470 51.560 57.920 1.00 0.00 H

ATOM 929 N ASN 95 33.170 50.280 54.090 1.00 0.00 N

ATOM 930 CA ASN 95 32.130 50.270 53.040 1.00 0.00 C

ATOM 931 C ASN 95 31.870 48.830 52.600 1.00 0.00 C

ATOM 932 O ASN 95 32.790 48.030 52.460 1.00 0.00 O

ATOM 933 CB ASN 95 32.600 51.070 51.830 1.00 0.00 C

ATOM 934 CG ASN 95 33.050 52.490 52.170 1.00 0.00 C

ATOM 935 ND2 ASN 95 34.270 52.790 51.810 1.00 0.00 N

ATOM 936 OD1 ASN 95 32.370 53.280 52.820 1.00 0.00 O

ATOM 937 H ASN 95 34.060 49.890 53.820 1.00 0.00 H

ATOM 938 1HD2 ASN 95 34.860 52.110 51.360 1.00 0.00 H

ATOM 939 2HD2 ASN 95 34.650 53.680 52.030 1.00 0.00 H

ATOM 940 N VAL 96 30.580 48.540 52.420 1.00 0.00 N

ATOM 941 CA VAL 96 30.080 47.220 51.990 1.00 0.00 C

ATOM 942 C VAL 96 29.180 47.400 50.760 1.00 0.00 C

ATOM 943 O VAL 96 28.250 48.200 50.760 1.00 0.00 O

ATOM 944 CB VAL 96 29.300 46.520 53.140 1.00 0.00 C

ATOM 945 CG1 VAL 96 28.860 45.100 52.740 1.00 0.00 C

ATOM 946 CG2 VAL 96 30.120 46.400 54.430 1.00 0.00 C

ATOM 947 H VAL 96 29.880 49.240 52.580 1.00 0.00 H

ATOM 948 N TRP 97 29.480 46.600 49.740 1.00 0.00 N

ATOM 949 CA TRP 97 28.660 46.450 48.530 1.00 0.00 C

ATOM 950 C TRP 97 28.090 45.030 48.460 1.00 0.00 C

ATOM 951 O TRP 97 28.830 44.050 48.380 1.00 0.00 O

ATOM 952 CB TRP 97 29.500 46.710 47.270 1.00 0.00 C

ATOM 953 CG TRP 97 29.820 48.190 47.090 1.00 0.00 C

ATOM 954 CD1 TRP 97 29.100 49.050 46.390 1.00 0.00 C

ATOM 955 CD2 TRP 97 30.970 48.850 47.500 1.00 0.00 C

ATOM 956 CE2 TRP 97 30.890 50.130 46.980 1.00 0.00 C

ATOM 957 CE3 TRP 97 32.080 48.460 48.240 1.00 0.00 C

ATOM 958 NE1 TRP 97 29.750 50.210 46.290 1.00 0.00 N

ATOM 959 CZ2 TRP 97 31.900 51.040 47.220 1.00 0.00 C

ATOM 960 CZ3 TRP 97 33.090 49.370 48.480 1.00 0.00 C

ATOM 961 CH2 TRP 97 33.000 50.670 47.980 1.00 0.00 C

ATOM 962 H TRP 97 30.350 46.100 49.740 1.00 0.00 H

ATOM 963 HD1 TRP 97 28.100 48.850 46.010 1.00 0.00 H

ATOM 964 HE1 TRP 97 29.530 50.920 45.620 1.00 0.00 H

ATOM 965 HE3 TRP 97 32.160 47.430 48.600 1.00 0.00 H

ATOM 966 HZ2 TRP 97 31.850 52.050 46.800 1.00 0.00 H

ATOM 967 HZ3 TRP 97 33.970 49.070 49.060 1.00 0.00 H

ATOM 968 HH2 TRP 97 33.750 51.410 48.250 1.00 0.00 H

ATOM 969 N THR 98 26.760 44.960 48.520 1.00 0.00 N

ATOM 970 CA THR 98 26.030 43.680 48.470 1.00 0.00 C

ATOM 971 C THR 98 25.150 43.570 47.210 1.00 0.00 C

ATOM 972 O THR 98 24.490 44.540 46.820 1.00 0.00 O

ATOM 973 CB THR 98 25.170 43.380 49.710 1.00 0.00 C

ATOM 974 CG2 THR 98 26.030 43.240 50.970 1.00 0.00 C

ATOM 975 OG1 THR 98 24.130 44.340 49.910 1.00 0.00 O

ATOM 976 H THR 98 26.180 45.780 48.600 1.00 0.00 H

ATOM 977 HG1 THR 98 23.440 43.880 50.510 1.00 0.00 H

ATOM 978 N PRO 99 25.200 42.400 46.550 1.00 0.00 N

ATOM 979 CA PRO 99 24.360 42.110 45.370 1.00 0.00 C

ATOM 980 C PRO 99 22.860 42.170 45.710 1.00 0.00 C

ATOM 981 O PRO 99 22.460 42.030 46.860 1.00 0.00 O

ATOM 982 CB PRO 99 24.770 40.700 44.950 1.00 0.00 C

ATOM 983 CG PRO 99 26.230 40.630 45.380 1.00 0.00 C

ATOM 984 CD PRO 99 26.230 41.360 46.720 1.00 0.00 C

ATOM 985 N VAL 100 22.090 42.610 44.720 1.00 0.00 N

ATOM 986 CA VAL 100 20.610 42.700 44.800 1.00 0.00 C

ATOM 987 C VAL 100 20.000 41.820 43.680 1.00 0.00 C

ATOM 988 O VAL 100 20.450 41.900 42.540 1.00 0.00 O

ATOM 989 CB VAL 100 20.190 44.170 44.630 1.00 0.00 C

ATOM 990 CG1 VAL 100 18.690 44.400 44.760 1.00 0.00 C

ATOM 991 CG2 VAL 100 20.830 45.110 45.650 1.00 0.00 C

ATOM 992 H VAL 100 22.480 43.030 43.900 1.00 0.00 H

ATOM 993 N PHE 101 19.160 40.830 43.970 1.00 0.00 N

ATOM 994 CA PHE 101 18.780 40.350 45.320 1.00 0.00 C

ATOM 995 C PHE 101 19.260 38.900 45.510 1.00 0.00 C

ATOM 996 O PHE 101 20.440 38.700 45.810 1.00 0.00 O

ATOM 997 CB PHE 101 17.260 40.390 45.540 1.00 0.00 C

ATOM 998 CG PHE 101 16.580 41.720 45.310 1.00 0.00 C

ATOM 999 CD1 PHE 101 16.240 42.110 44.030 1.00 0.00 C

ATOM 1000 CD2 PHE 101 16.270 42.510 46.400 1.00 0.00 C

ATOM 1001 CE1 PHE 101 15.590 43.320 43.820 1.00 0.00 C

ATOM 1002 CE2 PHE 101 15.650 43.740 46.200 1.00 0.00 C

ATOM 1003 CZ PHE 101 15.300 44.130 44.910 1.00 0.00 C

ATOM 1004 H PHE 101 18.680 40.380 43.220 1.00 0.00 H

ATOM 1005 HD1 PHE 101 16.530 41.490 43.190 1.00 0.00 H

ATOM 1006 HD2 PHE 101 16.350 42.090 47.400 1.00 0.00 H

ATOM 1007 HE1 PHE 101 15.370 43.670 42.810 1.00 0.00 H

ATOM 1008 HE2 PHE 101 15.590 44.450 47.010 1.00 0.00 H

ATOM 1009 HZ PHE 101 14.870 45.120 44.760 1.00 0.00 H

ATOM 1010 N ASN 102 18.430 37.990 44.990 1.00 0.00 N

ATOM 1011 CA ASN 102 18.220 36.540 45.200 1.00 0.00 C

ATOM 1012 C ASN 102 16.770 36.460 45.730 1.00 0.00 C

ATOM 1013 O ASN 102 15.910 37.100 45.120 1.00 0.00 O

ATOM 1014 CB ASN 102 19.300 35.810 46.020 1.00 0.00 C

ATOM 1015 CG ASN 102 20.650 35.810 45.290 1.00 0.00 C

ATOM 1016 ND2 ASN 102 20.640 35.580 43.990 1.00 0.00 N

ATOM 1017 OD1 ASN 102 21.660 36.280 45.780 1.00 0.00 O

ATOM 1018 H ASN 102 17.670 38.360 44.440 1.00 0.00 H

ATOM 1019 1HD2 ASN 102 19.780 35.450 43.500 1.00 0.00 H

ATOM 1020 2HD2 ASN 102 21.520 35.650 43.520 1.00 0.00 H

ATOM 1021 N LYS 103 16.430 35.700 46.770 1.00 0.00 N

ATOM 1022 CA LYS 103 15.130 35.900 47.460 1.00 0.00 C

ATOM 1023 C LYS 103 15.050 37.320 48.070 1.00 0.00 C

ATOM 1024 O LYS 103 14.030 38.000 48.060 1.00 0.00 O

ATOM 1025 CB LYS 103 14.950 34.830 48.540 1.00 0.00 C

ATOM 1026 CG LYS 103 13.470 34.580 48.830 1.00 0.00 C

ATOM 1027 CD LYS 103 13.280 33.170 49.400 1.00 0.00 C

ATOM 1028 CE LYS 103 11.810 32.780 49.340 1.00 0.00 C

ATOM 1029 NZ LYS 103 11.560 31.460 49.930 1.00 0.00 N1+

ATOM 1030 H LYS 103 16.960 34.880 47.020 1.00 0.00 H

ATOM 1031 HZ1 LYS 103 10.590 31.250 49.890 1.00 0.00 H

ATOM 1032 HZ2 LYS 103 12.070 30.760 49.420 1.00 0.00 H

ATOM 1033 HZ3 LYS 103 11.870 31.440 50.880 1.00 0.00 H

ATOM 1034 N THR 104 16.210 37.660 48.610 1.00 0.00 N

ATOM 1035 CA THR 104 16.720 38.960 49.100 1.00 0.00 C

ATOM 1036 C THR 104 18.210 38.940 48.670 1.00 0.00 C

ATOM 1037 O THR 104 18.620 37.970 48.040 1.00 0.00 O

ATOM 1038 CB THR 104 16.530 39.050 50.630 1.00 0.00 C

ATOM 1039 CG2 THR 104 17.240 37.960 51.430 1.00 0.00 C

ATOM 1040 OG1 THR 104 16.910 40.350 51.100 1.00 0.00 O

ATOM 1041 H THR 104 16.960 37.000 48.550 1.00 0.00 H

ATOM 1042 HG1 THR 104 16.160 40.690 51.680 1.00 0.00 H

ATOM 1043 N GLN 105 19.040 39.880 49.120 1.00 0.00 N

ATOM 1044 CA GLN 105 20.510 39.840 48.870 1.00 0.00 C

ATOM 1045 C GLN 105 21.110 38.440 49.210 1.00 0.00 C

ATOM 1046 O GLN 105 20.480 37.720 49.990 1.00 0.00 O

ATOM 1047 CB GLN 105 21.170 40.970 49.670 1.00 0.00 C

ATOM 1048 CG GLN 105 21.140 40.790 51.190 1.00 0.00 C

ATOM 1049 CD GLN 105 21.730 41.980 51.940 1.00 0.00 C

ATOM 1050 NE2 GLN 105 21.210 42.180 53.120 1.00 0.00 N

ATOM 1051 OE1 GLN 105 22.510 42.800 51.470 1.00 0.00 O

ATOM 1052 H GLN 105 18.710 40.600 49.730 1.00 0.00 H

ATOM 1053 1HE2 GLN 105 20.440 41.620 53.430 1.00 0.00 H

ATOM 1054 2HE2 GLN 105 21.710 42.770 53.740 1.00 0.00 H

ATOM 1055 N PRO 106 22.260 38.030 48.640 1.00 0.00 N

ATOM 1056 CA PRO 106 22.780 36.640 48.690 1.00 0.00 C

ATOM 1057 C PRO 106 22.520 35.870 49.990 1.00 0.00 C

ATOM 1058 O PRO 106 23.040 36.200 51.050 1.00 0.00 O

ATOM 1059 CB PRO 106 24.270 36.770 48.420 1.00 0.00 C

ATOM 1060 CG PRO 106 24.310 37.930 47.420 1.00 0.00 C

ATOM 1061 CD PRO 106 23.250 38.890 47.960 1.00 0.00 C

ATOM 1062 N LEU 107 21.560 34.960 49.910 1.00 0.00 N

ATOM 1063 CA LEU 107 21.210 34.050 51.030 1.00 0.00 C

ATOM 1064 C LEU 107 22.260 32.970 51.300 1.00 0.00 C

ATOM 1065 O LEU 107 22.740 32.900 52.430 1.00 0.00 O

ATOM 1066 CB LEU 107 19.820 33.420 50.880 1.00 0.00 C

ATOM 1067 CG LEU 107 18.670 34.290 51.370 1.00 0.00 C

ATOM 1068 CD1 LEU 107 17.360 33.610 50.970 1.00 0.00 C

ATOM 1069 CD2 LEU 107 18.720 34.420 52.890 1.00 0.00 C

ATOM 1070 H LEU 107 21.070 34.810 49.060 1.00 0.00 H

ATOM 1071 N PRO 108 22.640 32.120 50.320 1.00 0.00 N

ATOM 1072 CA PRO 108 23.750 31.180 50.510 1.00 0.00 C

ATOM 1073 C PRO 108 24.990 32.070 50.510 1.00 0.00 C

ATOM 1074 O PRO 108 25.230 32.810 49.560 1.00 0.00 O

ATOM 1075 CB PRO 108 23.760 30.270 49.290 1.00 0.00 C

ATOM 1076 CG PRO 108 22.460 30.590 48.560 1.00 0.00 C

ATOM 1077 CD PRO 108 22.150 32.030 48.930 1.00 0.00 C

ATOM 1078 N SER 109 25.610 32.100 51.670 1.00 0.00 N

ATOM 1079 CA SER 109 26.670 33.050 52.030 1.00 0.00 C

ATOM 1080 C SER 109 27.680 33.340 50.910 1.00 0.00 C

ATOM 1081 O SER 109 28.390 32.460 50.420 1.00 0.00 O

ATOM 1082 CB SER 109 27.330 32.510 53.290 1.00 0.00 C

ATOM 1083 OG SER 109 27.780 31.170 53.080 1.00 0.00 O

ATOM 1084 H SER 109 25.460 31.390 52.370 1.00 0.00 H

ATOM 1085 HG SER 109 28.670 31.050 53.580 1.00 0.00 H

ATOM 1086 N ALA 110 27.530 34.550 50.380 1.00 0.00 N

ATOM 1087 CA ALA 110 28.360 35.070 49.280 1.00 0.00 C

ATOM 1088 C ALA 110 29.830 35.220 49.700 1.00 0.00 C

ATOM 1089 O ALA 110 30.100 35.630 50.830 1.00 0.00 O

ATOM 1090 CB ALA 110 27.840 36.430 48.810 1.00 0.00 C

ATOM 1091 H ALA 110 26.800 35.170 50.690 1.00 0.00 H

ATOM 1092 N PRO 111 30.770 34.870 48.820 1.00 0.00 N

ATOM 1093 CA PRO 111 32.210 35.120 49.050 1.00 0.00 C

ATOM 1094 C PRO 111 32.430 36.630 49.200 1.00 0.00 C

ATOM 1095 O PRO 111 31.770 37.450 48.570 1.00 0.00 O

ATOM 1096 CB PRO 111 32.910 34.550 47.820 1.00 0.00 C

ATOM 1097 CG PRO 111 31.990 33.390 47.470 1.00 0.00 C

ATOM 1098 CD PRO 111 30.600 33.970 47.670 1.00 0.00 C

ATOM 1099 N VAL 112 33.320 36.940 50.130 1.00 0.00 N

ATOM 1100 CA VAL 112 33.560 38.330 50.580 1.00 0.00 C

ATOM 1101 C VAL 112 35.000 38.720 50.230 1.00 0.00 C

ATOM 1102 O VAL 112 35.970 38.160 50.740 1.00 0.00 O

ATOM 1103 CB VAL 112 33.310 38.480 52.090 1.00 0.00 C

ATOM 1104 CG1 VAL 112 33.410 39.950 52.520 1.00 0.00 C

ATOM 1105 CG2 VAL 112 31.940 37.930 52.500 1.00 0.00 C

ATOM 1106 H VAL 112 33.820 36.230 50.640 1.00 0.00 H

ATOM 1107 N LEU 113 35.100 39.790 49.460 1.00 0.00 N

ATOM 1108 CA LEU 113 36.380 40.330 49.000 1.00 0.00 C

ATOM 1109 C LEU 113 36.630 41.680 49.670 1.00 0.00 C

ATOM 1110 O LEU 113 35.840 42.620 49.570 1.00 0.00 O

ATOM 1111 CB LEU 113 36.400 40.460 47.470 1.00 0.00 C

ATOM 1112 CG LEU 113 36.500 39.130 46.700 1.00 0.00 C

ATOM 1113 CD1 LEU 113 35.210 38.300 46.760 1.00 0.00 C

ATOM 1114 CD2 LEU 113 36.820 39.390 45.240 1.00 0.00 C

ATOM 1115 H LEU 113 34.280 40.280 49.150 1.00 0.00 H

ATOM 1116 N VAL 114 37.700 41.700 50.470 1.00 0.00 N

ATOM 1117 CA VAL 114 38.070 42.880 51.270 1.00 0.00 C

ATOM 1118 C VAL 114 39.340 43.520 50.700 1.00 0.00 C

ATOM 1119 O VAL 114 40.400 42.890 50.610 1.00 0.00 O

ATOM 1120 CB VAL 114 38.200 42.540 52.770 1.00 0.00 C

ATOM 1121 CG1 VAL 114 38.540 43.770 53.620 1.00 0.00 C

ATOM 1122 CG2 VAL 114 36.900 41.940 53.310 1.00 0.00 C

ATOM 1123 H VAL 114 38.310 40.910 50.540 1.00 0.00 H

ATOM 1124 N TRP 115 39.120 44.740 50.240 1.00 0.00 N

ATOM 1125 CA TRP 115 40.160 45.610 49.660 1.00 0.00 C

ATOM 1126 C TRP 115 40.890 46.420 50.740 1.00 0.00 C

ATOM 1127 O TRP 115 40.290 47.060 51.610 1.00 0.00 O

ATOM 1128 CB TRP 115 39.520 46.540 48.630 1.00 0.00 C

ATOM 1129 CG TRP 115 40.550 47.510 48.030 1.00 0.00 C

ATOM 1130 CD1 TRP 115 40.630 48.820 48.250 1.00 0.00 C

ATOM 1131 CD2 TRP 115 41.580 47.170 47.160 1.00 0.00 C

ATOM 1132 CE2 TRP 115 42.240 48.350 46.880 1.00 0.00 C

ATOM 1133 CE3 TRP 115 41.940 45.990 46.540 1.00 0.00 C

ATOM 1134 NE1 TRP 115 41.650 49.320 47.550 1.00 0.00 N

ATOM 1135 CZ2 TRP 115 43.270 48.340 45.930 1.00 0.00 C

ATOM 1136 CZ3 TRP 115 42.940 45.990 45.570 1.00 0.00 C

ATOM 1137 CH2 TRP 115 43.600 47.180 45.270 1.00 0.00 C

ATOM 1138 H TRP 115 38.220 45.170 50.330 1.00 0.00 H

ATOM 1139 HD1 TRP 115 39.980 49.390 48.910 1.00 0.00 H

ATOM 1140 HE1 TRP 115 41.890 50.290 47.500 1.00 0.00 H

ATOM 1141 HE3 TRP 115 41.560 45.050 46.920 1.00 0.00 H

ATOM 1142 HZ2 TRP 115 43.860 49.240 45.770 1.00 0.00 H

ATOM 1143 HZ3 TRP 115 43.200 45.080 45.040 1.00 0.00 H

ATOM 1144 HH2 TRP 115 44.330 47.200 44.460 1.00 0.00 H

ATOM 1145 N ILE 116 42.210 46.380 50.610 1.00 0.00 N

ATOM 1146 CA ILE 116 43.150 47.210 51.400 1.00 0.00 C

ATOM 1147 C ILE 116 43.950 48.070 50.400 1.00 0.00 C

ATOM 1148 O ILE 116 44.720 47.550 49.610 1.00 0.00 O

ATOM 1149 CB ILE 116 44.070 46.370 52.300 1.00 0.00 C

ATOM 1150 CG1 ILE 116 43.260 45.410 53.200 1.00 0.00 C

ATOM 1151 CG2 ILE 116 44.920 47.300 53.170 1.00 0.00 C

ATOM 1152 CD ILE 116 44.080 44.290 53.840 1.00 0.00 C

ATOM 1153 H ILE 116 42.640 45.780 49.930 1.00 0.00 H

ATOM 1154 N HIS 117 43.600 49.360 50.370 1.00 0.00 N

ATOM 1155 CA HIS 117 44.270 50.360 49.510 1.00 0.00 C

ATOM 1156 C HIS 117 45.780 50.510 49.820 1.00 0.00 C

ATOM 1157 O HIS 117 46.200 50.330 50.970 1.00 0.00 O

ATOM 1158 CB HIS 117 43.550 51.710 49.650 1.00 0.00 C

ATOM 1159 CG HIS 117 43.780 52.430 50.980 1.00 0.00 C

ATOM 1160 CD2 HIS 117 42.920 52.520 52.000 1.00 0.00 C

ATOM 1161 ND1 HIS 117 44.900 53.050 51.330 1.00 0.00 N

ATOM 1162 CE1 HIS 117 44.740 53.500 52.580 1.00 0.00 C

ATOM 1163 NE2 HIS 117 43.520 53.190 52.990 1.00 0.00 N

ATOM 1164 H HIS 117 42.780 49.690 50.860 1.00 0.00 H

ATOM 1165 HD1 HIS 117 45.700 53.200 50.740 1.00 0.00 H

ATOM 1166 N GLY 118 46.550 50.840 48.790 1.00 0.00 N

ATOM 1167 CA GLY 118 47.980 51.210 48.920 1.00 0.00 C

ATOM 1168 C GLY 118 48.210 52.690 49.290 1.00 0.00 C

ATOM 1169 O GLY 118 47.310 53.380 49.770 1.00 0.00 O

ATOM 1170 H GLY 118 46.250 50.700 47.830 1.00 0.00 H

ATOM 1171 N GLY 119 49.430 53.140 49.010 1.00 0.00 N

ATOM 1172 CA GLY 119 49.910 54.500 49.330 1.00 0.00 C

ATOM 1173 C GLY 119 51.090 54.630 50.300 1.00 0.00 C

ATOM 1174 O GLY 119 51.200 55.590 51.060 1.00 0.00 O

ATOM 1175 H GLY 119 50.060 52.610 48.430 1.00 0.00 H

ATOM 1176 N GLY 120 51.960 53.630 50.230 1.00 0.00 N

ATOM 1177 CA GLY 120 53.210 53.480 51.010 1.00 0.00 C

ATOM 1178 C GLY 120 53.070 53.730 52.510 1.00 0.00 C

ATOM 1179 O GLY 120 53.920 54.400 53.100 1.00 0.00 O

ATOM 1180 H GLY 120 51.890 52.960 49.470 1.00 0.00 H

ATOM 1181 N PHE 121 51.920 53.350 53.060 1.00 0.00 N

ATOM 1182 CA PHE 121 51.560 53.550 54.480 1.00 0.00 C

ATOM 1183 C PHE 121 51.480 55.010 54.950 1.00 0.00 C

ATOM 1184 O PHE 121 51.050 55.280 56.070 1.00 0.00 O

ATOM 1185 CB PHE 121 52.490 52.740 55.390 1.00 0.00 C

ATOM 1186 CG PHE 121 52.420 51.250 55.050 1.00 0.00 C

ATOM 1187 CD1 PHE 121 51.370 50.490 55.550 1.00 0.00 C

ATOM 1188 CD2 PHE 121 53.390 50.660 54.250 1.00 0.00 C

ATOM 1189 CE1 PHE 121 51.290 49.130 55.260 1.00 0.00 C

ATOM 1190 CE2 PHE 121 53.310 49.300 53.950 1.00 0.00 C

ATOM 1191 CZ PHE 121 52.260 48.540 54.450 1.00 0.00 C

ATOM 1192 H PHE 121 51.220 52.890 52.510 1.00 0.00 H

ATOM 1193 HD1 PHE 121 50.590 50.970 56.130 1.00 0.00 H

ATOM 1194 HD2 PHE 121 54.200 51.260 53.830 1.00 0.00 H

ATOM 1195 HE1 PHE 121 50.510 48.530 55.700 1.00 0.00 H

ATOM 1196 HE2 PHE 121 54.120 48.820 53.410 1.00 0.00 H

ATOM 1197 HZ PHE 121 52.200 47.480 54.220 1.00 0.00 H

ATOM 1198 N VAL 122 51.670 55.940 54.010 1.00 0.00 N

ATOM 1199 CA VAL 122 51.640 57.400 54.230 1.00 0.00 C

ATOM 1200 C VAL 122 50.270 57.970 53.850 1.00 0.00 C

ATOM 1201 O VAL 122 49.770 58.880 54.520 1.00 0.00 O

ATOM 1202 CB VAL 122 52.790 58.040 53.400 1.00 0.00 C

ATOM 1203 CG1 VAL 122 52.810 59.570 53.450 1.00 0.00 C

ATOM 1204 CG2 VAL 122 54.160 57.550 53.870 1.00 0.00 C

ATOM 1205 H VAL 122 51.990 55.660 53.100 1.00 0.00 H

ATOM 1206 N THR 123 49.730 57.480 52.730 1.00 0.00 N

ATOM 1207 CA THR 123 48.480 57.970 52.100 1.00 0.00 C

ATOM 1208 C THR 123 47.540 56.830 51.690 1.00 0.00 C

ATOM 1209 O THR 123 47.830 55.650 51.930 1.00 0.00 O

ATOM 1210 CB THR 123 48.830 58.830 50.880 1.00 0.00 C

ATOM 1211 CG2 THR 123 49.620 60.080 51.250 1.00 0.00 C

ATOM 1212 OG1 THR 123 49.580 58.070 49.930 1.00 0.00 O

ATOM 1213 H THR 123 50.180 56.770 52.190 1.00 0.00 H

ATOM 1214 HG1 THR 123 49.090 58.110 49.020 1.00 0.00 H

ATOM 1215 N GLY 124 46.360 57.230 51.220 1.00 0.00 N

ATOM 1216 CA GLY 124 45.310 56.330 50.720 1.00 0.00 C

ATOM 1217 C GLY 124 43.910 56.530 51.300 1.00 0.00 C

ATOM 1218 O GLY 124 43.720 57.160 52.340 1.00 0.00 O

ATOM 1219 H GLY 124 46.160 58.210 51.100 1.00 0.00 H

ATOM 1220 N THR 125 42.940 56.010 50.570 1.00 0.00 N

ATOM 1221 CA THR 125 41.530 55.970 51.010 1.00 0.00 C

ATOM 1222 C THR 125 40.690 54.970 50.210 1.00 0.00 C

ATOM 1223 O THR 125 40.790 54.890 48.990 1.00 0.00 O

ATOM 1224 CB THR 125 40.880 57.370 51.020 1.00 0.00 C

ATOM 1225 CG2 THR 125 40.780 58.040 49.640 1.00 0.00 C

ATOM 1226 OG1 THR 125 39.600 57.300 51.650 1.00 0.00 O

ATOM 1227 H THR 125 43.120 55.660 49.640 1.00 0.00 H

ATOM 1228 HG1 THR 125 39.340 58.250 51.910 1.00 0.00 H

ATOM 1229 N SER 126 39.760 54.340 50.920 1.00 0.00 N

ATOM 1230 CA SER 126 38.770 53.400 50.330 1.00 0.00 C

ATOM 1231 C SER 126 37.770 54.090 49.390 1.00 0.00 C

ATOM 1232 O SER 126 37.090 53.440 48.590 1.00 0.00 O

ATOM 1233 CB SER 126 37.990 52.690 51.440 1.00 0.00 C

ATOM 1234 OG SER 126 37.090 53.570 52.130 1.00 0.00 O

ATOM 1235 H SER 126 39.620 54.540 51.900 1.00 0.00 H

ATOM 1236 HG SER 126 37.200 53.430 53.120 1.00 0.00 H

ATOM 1237 N THR 127 37.670 55.400 49.540 1.00 0.00 N

ATOM 1238 CA THR 127 36.640 56.250 48.920 1.00 0.00 C

ATOM 1239 C THR 127 36.880 56.610 47.440 1.00 0.00 C

ATOM 1240 O THR 127 35.960 57.120 46.790 1.00 0.00 O

ATOM 1241 CB THR 127 36.380 57.500 49.790 1.00 0.00 C

ATOM 1242 CG2 THR 127 36.000 57.120 51.230 1.00 0.00 C

ATOM 1243 OG1 THR 127 37.550 58.300 49.870 1.00 0.00 O

ATOM 1244 H THR 127 38.310 55.900 50.140 1.00 0.00 H

ATOM 1245 HG1 THR 127 37.300 59.270 49.750 1.00 0.00 H

ATOM 1246 N ILE 128 38.090 56.340 46.940 1.00 0.00 N

ATOM 1247 CA ILE 128 38.540 56.510 45.540 1.00 0.00 C

ATOM 1248 C ILE 128 37.610 55.850 44.510 1.00 0.00 C

ATOM 1249 O ILE 128 37.170 54.710 44.680 1.00 0.00 O

ATOM 1250 CB ILE 128 39.990 55.970 45.530 1.00 0.00 C

ATOM 1251 CG1 ILE 128 41.010 56.910 46.190 1.00 0.00 C

ATOM 1252 CG2 ILE 128 40.520 55.370 44.210 1.00 0.00 C

ATOM 1253 CD ILE 128 41.070 58.340 45.640 1.00 0.00 C

ATOM 1254 H ILE 128 38.810 56.010 47.550 1.00 0.00 H

ATOM 1255 N ASP 129 37.480 56.510 43.360 1.00 0.00 N

ATOM 1256 CA ASP 129 36.660 56.060 42.210 1.00 0.00 C

ATOM 1257 C ASP 129 37.090 54.690 41.670 1.00 0.00 C

ATOM 1258 O ASP 129 36.240 53.820 41.470 1.00 0.00 O

ATOM 1259 CB ASP 129 36.670 57.060 41.050 1.00 0.00 C

ATOM 1260 CG ASP 129 36.020 58.420 41.330 1.00 0.00 C

ATOM 1261 OD1 ASP 129 35.500 58.640 42.440 1.00 0.00 O

ATOM 1262 OD2 ASP 129 36.040 59.260 40.410 1.00 0.00 O

ATOM 1263 H ASP 129 37.890 57.430 43.230 1.00 0.00 H

ATOM 1264 N LEU 130 38.400 54.480 41.560 1.00 0.00 N

ATOM 1265 CA LEU 130 38.970 53.170 41.140 1.00 0.00 C

ATOM 1266 C LEU 130 38.590 52.010 42.070 1.00 0.00 C

ATOM 1267 O LEU 130 38.340 50.910 41.590 1.00 0.00 O

ATOM 1268 CB LEU 130 40.500 53.220 41.030 1.00 0.00 C

ATOM 1269 CG LEU 130 41.090 54.320 40.130 1.00 0.00 C

ATOM 1270 CD1 LEU 130 42.600 54.150 40.050 1.00 0.00 C

ATOM 1271 CD2 LEU 130 40.550 54.270 38.710 1.00 0.00 C

ATOM 1272 H LEU 130 39.070 55.230 41.650 1.00 0.00 H

ATOM 1273 N TYR 131 38.380 52.310 43.360 1.00 0.00 N

ATOM 1274 CA TYR 131 38.000 51.330 44.390 1.00 0.00 C

ATOM 1275 C TYR 131 36.500 50.990 44.500 1.00 0.00 C

ATOM 1276 O TYR 131 36.120 50.170 45.340 1.00 0.00 O

ATOM 1277 CB TYR 131 38.500 51.780 45.770 1.00 0.00 C

ATOM 1278 CG TYR 131 40.020 51.890 45.900 1.00 0.00 C

ATOM 1279 CD1 TYR 131 40.890 51.050 45.200 1.00 0.00 C

ATOM 1280 CD2 TYR 131 40.540 52.970 46.590 1.00 0.00 C

ATOM 1281 CE1 TYR 131 42.250 51.330 45.170 1.00 0.00 C

ATOM 1282 CE2 TYR 131 41.890 53.260 46.560 1.00 0.00 C

ATOM 1283 CZ TYR 131 42.750 52.440 45.840 1.00 0.00 C

ATOM 1284 OH TYR 131 44.070 52.730 45.810 1.00 0.00 O

ATOM 1285 H TYR 131 38.500 53.250 43.690 1.00 0.00 H

ATOM 1286 HD1 TYR 131 40.520 50.140 44.730 1.00 0.00 H

ATOM 1287 HD2 TYR 131 39.860 53.640 47.120 1.00 0.00 H

ATOM 1288 HE1 TYR 131 42.890 50.740 44.520 1.00 0.00 H

ATOM 1289 HE2 TYR 131 42.250 54.180 47.000 1.00 0.00 H

ATOM 1290 HH TYR 131 44.130 53.730 45.730 1.00 0.00 H

ATOM 1291 N HIS 132 35.640 51.660 43.730 1.00 0.00 N

ATOM 1292 CA HIS 132 34.180 51.410 43.730 1.00 0.00 C

ATOM 1293 C HIS 132 33.780 49.940 43.510 1.00 0.00 C

ATOM 1294 O HIS 132 33.770 49.410 42.400 1.00 0.00 O

ATOM 1295 CB HIS 132 33.460 52.280 42.700 1.00 0.00 C

ATOM 1296 CG HIS 132 32.960 53.620 43.240 1.00 0.00 C

ATOM 1297 CD2 HIS 132 33.420 54.800 42.840 1.00 0.00 C

ATOM 1298 ND1 HIS 132 31.790 53.820 43.840 1.00 0.00 N

ATOM 1299 CE1 HIS 132 31.520 55.130 43.750 1.00 0.00 C

ATOM 1300 NE2 HIS 132 32.530 55.730 43.140 1.00 0.00 N

ATOM 1301 H HIS 132 35.960 52.360 43.090 1.00 0.00 H

ATOM 1302 HE2 HIS 132 32.690 56.720 43.090 1.00 0.00 H

ATOM 1303 N GLY 133 33.340 49.340 44.610 1.00 0.00 N

ATOM 1304 CA GLY 133 32.950 47.920 44.700 1.00 0.00 C

ATOM 1305 C GLY 133 31.750 47.440 43.870 1.00 0.00 C

ATOM 1306 O GLY 133 31.630 46.240 43.610 1.00 0.00 O

ATOM 1307 H GLY 133 33.380 49.820 45.490 1.00 0.00 H

ATOM 1308 N HIS 134 30.910 48.360 43.380 1.00 0.00 N

ATOM 1309 CA HIS 134 29.630 47.990 42.730 1.00 0.00 C

ATOM 1310 C HIS 134 29.750 47.020 41.550 1.00 0.00 C

ATOM 1311 O HIS 134 29.010 46.040 41.510 1.00 0.00 O

ATOM 1312 CB HIS 134 28.840 49.180 42.190 1.00 0.00 C

ATOM 1313 CG HIS 134 28.410 50.170 43.250 1.00 0.00 C

ATOM 1314 CD2 HIS 134 27.220 50.180 43.820 1.00 0.00 C

ATOM 1315 ND1 HIS 134 29.020 51.320 43.510 1.00 0.00 N

ATOM 1316 CE1 HIS 134 28.160 52.060 44.200 1.00 0.00 C

ATOM 1317 NE2 HIS 134 27.070 51.350 44.420 1.00 0.00 N

ATOM 1318 H HIS 134 31.150 49.330 43.340 1.00 0.00 H

ATOM 1319 HE2 HIS 134 26.260 51.630 44.940 1.00 0.00 H

ATOM 1320 N ILE 135 30.660 47.310 40.610 1.00 0.00 N

ATOM 1321 CA ILE 135 30.810 46.450 39.410 1.00 0.00 C

ATOM 1322 C ILE 135 31.110 44.980 39.760 1.00 0.00 C

ATOM 1323 O ILE 135 30.350 44.110 39.330 1.00 0.00 O

ATOM 1324 CB ILE 135 31.780 47.050 38.370 1.00 0.00 C

ATOM 1325 CG1 ILE 135 31.260 48.430 37.920 1.00 0.00 C

ATOM 1326 CG2 ILE 135 31.930 46.110 37.150 1.00 0.00 C

ATOM 1327 CD ILE 135 32.200 49.230 37.000 1.00 0.00 C

ATOM 1328 H ILE 135 31.160 48.170 40.600 1.00 0.00 H

ATOM 1329 N LEU 136 32.080 44.730 40.640 1.00 0.00 N

ATOM 1330 CA LEU 136 32.410 43.360 41.080 1.00 0.00 C

ATOM 1331 C LEU 136 31.240 42.720 41.830 1.00 0.00 C

ATOM 1332 O LEU 136 30.790 41.640 41.440 1.00 0.00 O

ATOM 1333 CB LEU 136 33.650 43.380 41.980 1.00 0.00 C

ATOM 1334 CG LEU 136 34.370 42.020 41.970 1.00 0.00 C

ATOM 1335 CD1 LEU 136 35.230 41.870 40.720 1.00 0.00 C

ATOM 1336 CD2 LEU 136 35.230 41.890 43.210 1.00 0.00 C

ATOM 1337 H LEU 136 32.710 45.440 40.990 1.00 0.00 H

ATOM 1338 N SER 137 30.610 43.490 42.730 1.00 0.00 N

ATOM 1339 CA SER 137 29.380 43.050 43.430 1.00 0.00 C

ATOM 1340 C SER 137 28.270 42.610 42.460 1.00 0.00 C

ATOM 1341 O SER 137 27.930 41.430 42.430 1.00 0.00 O

ATOM 1342 CB SER 137 28.890 44.170 44.360 1.00 0.00 C

ATOM 1343 OG SER 137 27.640 43.840 44.970 1.00 0.00 O

ATOM 1344 H SER 137 30.980 44.380 43.000 1.00 0.00 H

ATOM 1345 HG SER 137 27.640 44.150 45.920 1.00 0.00 H

ATOM 1346 N LYS 138 27.970 43.470 41.480 1.00 0.00 N

ATOM 1347 CA LYS 138 26.970 43.200 40.430 1.00 0.00 C

ATOM 1348 C LYS 138 27.370 42.020 39.520 1.00 0.00 C

ATOM 1349 O LYS 138 26.590 41.080 39.380 1.00 0.00 O

ATOM 1350 CB LYS 138 26.710 44.470 39.600 1.00 0.00 C

ATOM 1351 CG LYS 138 25.430 44.440 38.750 1.00 0.00 C

ATOM 1352 CD LYS 138 25.590 43.610 37.460 1.00 0.00 C

ATOM 1353 CE LYS 138 24.260 43.350 36.760 1.00 0.00 C

ATOM 1354 NZ LYS 138 24.440 42.260 35.790 1.00 0.00 N1+

ATOM 1355 H LYS 138 28.380 44.390 41.460 1.00 0.00 H

ATOM 1356 HZ1 LYS 138 23.610 42.110 35.250 1.00 0.00 H

ATOM 1357 HZ2 LYS 138 25.190 42.470 35.160 1.00 0.00 H

ATOM 1358 HZ3 LYS 138 24.670 41.410 36.270 1.00 0.00 H

ATOM 1359 N SER 139 28.510 42.140 38.840 1.00 0.00 N

ATOM 1360 CA SER 139 28.960 41.160 37.820 1.00 0.00 C

ATOM 1361 C SER 139 29.210 39.760 38.390 1.00 0.00 C

ATOM 1362 O SER 139 28.620 38.800 37.910 1.00 0.00 O

ATOM 1363 CB SER 139 30.250 41.620 37.130 1.00 0.00 C

ATOM 1364 OG SER 139 30.010 42.770 36.330 1.00 0.00 O

ATOM 1365 H SER 139 29.140 42.910 39.000 1.00 0.00 H

ATOM 1366 HG SER 139 30.890 43.220 36.160 1.00 0.00 H

ATOM 1367 N GLU 140 29.850 39.750 39.560 1.00 0.00 N

ATOM 1368 CA GLU 140 30.360 38.520 40.180 1.00 0.00 C

ATOM 1369 C GLU 140 29.470 37.880 41.250 1.00 0.00 C

ATOM 1370 O GLU 140 29.540 36.670 41.470 1.00 0.00 O

ATOM 1371 CB GLU 140 31.760 38.810 40.720 1.00 0.00 C

ATOM 1372 CG GLU 140 32.740 39.150 39.590 1.00 0.00 C

ATOM 1373 CD GLU 140 32.740 38.050 38.540 1.00 0.00 C

ATOM 1374 OE1 GLU 140 33.100 36.900 38.890 1.00 0.00 O

ATOM 1375 OE2 GLU 140 32.170 38.290 37.460 1.00 0.00 O

ATOM 1376 H GLU 140 30.180 40.590 39.990 1.00 0.00 H

ATOM 1377 N GLY 141 28.580 38.680 41.830 1.00 0.00 N

ATOM 1378 CA GLY 141 27.670 38.240 42.910 1.00 0.00 C

ATOM 1379 C GLY 141 28.410 38.010 44.240 1.00 0.00 C

ATOM 1380 O GLY 141 28.050 37.120 45.010 1.00 0.00 O

ATOM 1381 H GLY 141 28.500 39.650 41.600 1.00 0.00 H

ATOM 1382 N VAL 142 29.420 38.840 44.480 1.00 0.00 N

ATOM 1383 CA VAL 142 30.240 38.800 45.710 1.00 0.00 C

ATOM 1384 C VAL 142 30.060 40.070 46.540 1.00 0.00 C

ATOM 1385 O VAL 142 29.800 41.150 46.020 1.00 0.00 O

ATOM 1386 CB VAL 142 31.740 38.560 45.420 1.00 0.00 C

ATOM 1387 CG1 VAL 142 31.960 37.140 44.910 1.00 0.00 C

ATOM 1388 CG2 VAL 142 32.360 39.590 44.470 1.00 0.00 C

ATOM 1389 H VAL 142 29.630 39.600 43.860 1.00 0.00 H

ATOM 1390 N VAL 143 30.260 39.890 47.840 1.00 0.00 N

ATOM 1391 CA VAL 143 30.240 41.030 48.790 1.00 0.00 C

ATOM 1392 C VAL 143 31.640 41.670 48.780 1.00 0.00 C

ATOM 1393 O VAL 143 32.660 41.000 48.970 1.00 0.00 O

ATOM 1394 CB VAL 143 29.800 40.590 50.200 1.00 0.00 C

ATOM 1395 CG1 VAL 143 29.750 41.790 51.150 1.00 0.00 C

ATOM 1396 CG2 VAL 143 28.410 39.950 50.180 1.00 0.00 C

ATOM 1397 H VAL 143 30.570 39.010 48.220 1.00 0.00 H

ATOM 1398 N VAL 144 31.640 42.970 48.550 1.00 0.00 N

ATOM 1399 CA VAL 144 32.870 43.790 48.510 1.00 0.00 C

ATOM 1400 C VAL 144 32.900 44.680 49.760 1.00 0.00 C

ATOM 1401 O VAL 144 31.950 45.420 50.020 1.00 0.00 O

ATOM 1402 CB VAL 144 32.950 44.630 47.220 1.00 0.00 C

ATOM 1403 CG1 VAL 144 34.230 45.480 47.170 1.00 0.00 C

ATOM 1404 CG2 VAL 144 32.870 43.770 45.960 1.00 0.00 C

ATOM 1405 H VAL 144 30.780 43.460 48.350 1.00 0.00 H

ATOM 1406 N VAL 145 33.980 44.560 50.510 1.00 0.00 N

ATOM 1407 CA VAL 145 34.240 45.400 51.700 1.00 0.00 C

ATOM 1408 C VAL 145 35.590 46.120 51.520 1.00 0.00 C

ATOM 1409 O VAL 145 36.550 45.570 50.990 1.00 0.00 O

ATOM 1410 CB VAL 145 34.240 44.570 53.010 1.00 0.00 C

ATOM 1411 CG1 VAL 145 34.430 45.440 54.250 1.00 0.00 C

ATOM 1412 CG2 VAL 145 32.930 43.780 53.190 1.00 0.00 C

ATOM 1413 H VAL 145 34.710 43.890 50.280 1.00 0.00 H

ATOM 1414 N SER 146 35.580 47.400 51.880 1.00 0.00 N

ATOM 1415 CA SER 146 36.810 48.210 52.020 1.00 0.00 C

ATOM 1416 C SER 146 36.810 49.040 53.300 1.00 0.00 C

ATOM 1417 O SER 146 35.760 49.410 53.830 1.00 0.00 O

ATOM 1418 CB SER 146 37.060 49.110 50.810 1.00 0.00 C

ATOM 1419 OG SER 146 36.070 50.130 50.660 1.00 0.00 O

ATOM 1420 H SER 146 34.710 47.880 52.080 1.00 0.00 H

ATOM 1421 HG SER 146 36.150 50.430 49.670 1.00 0.00 H

ATOM 1422 N ILE 147 38.020 49.280 53.770 1.00 0.00 N

ATOM 1423 CA ILE 147 38.320 49.980 55.040 1.00 0.00 C

ATOM 1424 C ILE 147 39.210 51.220 54.790 1.00 0.00 C

ATOM 1425 O ILE 147 39.990 51.230 53.840 1.00 0.00 O

ATOM 1426 CB ILE 147 39.010 48.990 56.010 1.00 0.00 C

ATOM 1427 CG1 ILE 147 39.230 49.670 57.380 1.00 0.00 C

ATOM 1428 CG2 ILE 147 40.230 48.290 55.380 1.00 0.00 C

ATOM 1429 CD ILE 147 40.340 49.170 58.300 1.00 0.00 C

ATOM 1430 H ILE 147 38.830 48.970 53.270 1.00 0.00 H

ATOM 1431 N ASN 148 39.050 52.220 55.650 1.00 0.00 N

ATOM 1432 CA ASN 148 40.080 53.260 55.880 1.00 0.00 C

ATOM 1433 C ASN 148 40.830 52.940 57.180 1.00 0.00 C

ATOM 1434 O ASN 148 40.210 52.740 58.220 1.00 0.00 O

ATOM 1435 CB ASN 148 39.490 54.660 56.050 1.00 0.00 C

ATOM 1436 CG ASN 148 38.920 55.260 54.760 1.00 0.00 C

ATOM 1437 ND2 ASN 148 38.180 56.330 54.890 1.00 0.00 N

ATOM 1438 OD1 ASN 148 39.170 54.800 53.650 1.00 0.00 O

ATOM 1439 H ASN 148 38.230 52.300 56.220 1.00 0.00 H

ATOM 1440 1HD2 ASN 148 38.010 56.750 55.790 1.00 0.00 H

ATOM 1441 2HD2 ASN 148 37.910 56.800 54.060 1.00 0.00 H

ATOM 1442 N TYR 149 42.150 52.860 57.060 1.00 0.00 N

ATOM 1443 CA TYR 149 43.050 52.600 58.200 1.00 0.00 C

ATOM 1444 C TYR 149 44.070 53.740 58.350 1.00 0.00 C

ATOM 1445 O TYR 149 44.400 54.400 57.360 1.00 0.00 O

ATOM 1446 CB TYR 149 43.750 51.240 58.030 1.00 0.00 C

ATOM 1447 CG TYR 149 44.740 51.160 56.860 1.00 0.00 C

ATOM 1448 CD1 TYR 149 44.280 50.930 55.570 1.00 0.00 C

ATOM 1449 CD2 TYR 149 46.100 51.280 57.090 1.00 0.00 C

ATOM 1450 CE1 TYR 149 45.180 50.820 54.520 1.00 0.00 C

ATOM 1451 CE2 TYR 149 47.010 51.170 56.040 1.00 0.00 C

ATOM 1452 CZ TYR 149 46.550 50.940 54.750 1.00 0.00 C

ATOM 1453 OH TYR 149 47.450 50.580 53.800 1.00 0.00 O

ATOM 1454 H TYR 149 42.620 53.100 56.210 1.00 0.00 H

ATOM 1455 HD1 TYR 149 43.210 50.930 55.360 1.00 0.00 H

ATOM 1456 HD2 TYR 149 46.470 51.490 58.100 1.00 0.00 H

ATOM 1457 HE1 TYR 149 44.800 50.720 53.500 1.00 0.00 H

ATOM 1458 HE2 TYR 149 48.070 51.280 56.240 1.00 0.00 H

ATOM 1459 HH TYR 149 48.080 51.350 53.600 1.00 0.00 H

ATOM 1460 N ARG 150 44.550 53.940 59.570 1.00 0.00 N

ATOM 1461 CA ARG 150 45.500 55.040 59.850 1.00 0.00 C

ATOM 1462 C ARG 150 46.850 54.920 59.130 1.00 0.00 C

ATOM 1463 O ARG 150 47.490 53.870 59.090 1.00 0.00 O

ATOM 1464 CB ARG 150 45.700 55.200 61.360 1.00 0.00 C

ATOM 1465 CG ARG 150 44.480 55.860 62.000 1.00 0.00 C

ATOM 1466 CD ARG 150 44.660 55.930 63.510 1.00 0.00 C

ATOM 1467 NE ARG 150 44.520 54.580 64.090 1.00 0.00 N

ATOM 1468 CZ ARG 150 44.710 54.250 65.370 1.00 0.00 C

ATOM 1469 NH1 ARG 150 45.110 55.130 66.290 1.00 0.00 N

ATOM 1470 NH2 ARG 150 44.470 53.000 65.750 1.00 0.00 N

ATOM 1471 H ARG 150 44.310 53.360 60.360 1.00 0.00 H

ATOM 1472 HE ARG 150 44.300 53.850 63.430 1.00 0.00 H

ATOM 1473 1HH1 ARG 150 45.330 56.080 66.040 1.00 0.00 H

ATOM 1474 2HH1 ARG 150 45.220 54.830 67.240 1.00 0.00 H

ATOM 1475 1HH2 ARG 150 44.100 52.340 65.080 1.00 0.00 H

ATOM 1476 2HH2 ARG 150 44.710 52.710 66.680 1.00 0.00 H

ATOM 1477 N LEU 151 47.280 56.090 58.700 1.00 0.00 N

ATOM 1478 CA LEU 151 48.450 56.290 57.810 1.00 0.00 C

ATOM 1479 C LEU 151 49.450 57.320 58.370 1.00 0.00 C

ATOM 1480 O LEU 151 49.130 58.090 59.290 1.00 0.00 O

ATOM 1481 CB LEU 151 47.930 56.730 56.440 1.00 0.00 C

ATOM 1482 CG LEU 151 46.960 55.700 55.830 1.00 0.00 C

ATOM 1483 CD1 LEU 151 46.120 56.350 54.740 1.00 0.00 C

ATOM 1484 CD2 LEU 151 47.710 54.480 55.290 1.00 0.00 C

ATOM 1485 H LEU 151 46.780 56.930 58.930 1.00 0.00 H

ATOM 1486 N GLY 152 50.680 57.220 57.880 1.00 0.00 N

ATOM 1487 CA GLY 152 51.830 58.050 58.300 1.00 0.00 C

ATOM 1488 C GLY 152 52.120 57.830 59.790 1.00 0.00 C

ATOM 1489 O GLY 152 51.810 56.780 60.350 1.00 0.00 O

ATOM 1490 H GLY 152 50.900 56.510 57.190 1.00 0.00 H

ATOM 1491 N ALA 153 52.540 58.910 60.450 1.00 0.00 N

ATOM 1492 CA ALA 153 52.830 58.870 61.900 1.00 0.00 C

ATOM 1493 C ALA 153 51.630 58.430 62.760 1.00 0.00 C

ATOM 1494 O ALA 153 51.780 57.550 63.610 1.00 0.00 O

ATOM 1495 CB ALA 153 53.360 60.220 62.380 1.00 0.00 C

ATOM 1496 H ALA 153 52.780 59.760 59.970 1.00 0.00 H

ATOM 1497 N PHE 154 50.420 58.850 62.360 1.00 0.00 N

ATOM 1498 CA PHE 154 49.160 58.520 63.060 1.00 0.00 C

ATOM 1499 C PHE 154 48.900 57.010 63.170 1.00 0.00 C

ATOM 1500 O PHE 154 48.410 56.540 64.200 1.00 0.00 O

ATOM 1501 CB PHE 154 47.960 59.150 62.340 1.00 0.00 C

ATOM 1502 CG PHE 154 48.100 60.660 62.160 1.00 0.00 C

ATOM 1503 CD1 PHE 154 47.690 61.520 63.180 1.00 0.00 C

ATOM 1504 CD2 PHE 154 48.640 61.170 60.980 1.00 0.00 C

ATOM 1505 CE1 PHE 154 47.840 62.890 63.020 1.00 0.00 C

ATOM 1506 CE2 PHE 154 48.800 62.540 60.840 1.00 0.00 C

ATOM 1507 CZ PHE 154 48.410 63.400 61.860 1.00 0.00 C

ATOM 1508 H PHE 154 50.310 59.410 61.550 1.00 0.00 H

ATOM 1509 HD1 PHE 154 47.190 61.130 64.060 1.00 0.00 H

ATOM 1510 HD2 PHE 154 48.830 60.510 60.140 1.00 0.00 H

ATOM 1511 HE1 PHE 154 47.420 63.570 63.770 1.00 0.00 H

ATOM 1512 HE2 PHE 154 49.160 62.960 59.890 1.00 0.00 H

ATOM 1513 HZ PHE 154 48.530 64.470 61.740 1.00 0.00 H

ATOM 1514 N GLY 155 49.220 56.290 62.090 1.00 0.00 N

ATOM 1515 CA GLY 155 49.100 54.820 62.020 1.00 0.00 C

ATOM 1516 C GLY 155 50.370 54.030 62.380 1.00 0.00 C

ATOM 1517 O GLY 155 50.260 52.870 62.790 1.00 0.00 O

ATOM 1518 H GLY 155 49.460 56.730 61.230 1.00 0.00 H

ATOM 1519 N PHE 156 51.530 54.620 62.150 1.00 0.00 N

ATOM 1520 CA PHE 156 52.810 53.880 62.260 1.00 0.00 C

ATOM 1521 C PHE 156 53.910 54.360 63.210 1.00 0.00 C

ATOM 1522 O PHE 156 54.900 53.650 63.380 1.00 0.00 O

ATOM 1523 CB PHE 156 53.390 53.680 60.860 1.00 0.00 C

ATOM 1524 CG PHE 156 52.490 52.790 60.020 1.00 0.00 C

ATOM 1525 CD1 PHE 156 52.470 51.420 60.260 1.00 0.00 C

ATOM 1526 CD2 PHE 156 51.560 53.360 59.160 1.00 0.00 C

ATOM 1527 CE1 PHE 156 51.510 50.620 59.660 1.00 0.00 C

ATOM 1528 CE2 PHE 156 50.590 52.570 58.560 1.00 0.00 C

ATOM 1529 CZ PHE 156 50.570 51.200 58.820 1.00 0.00 C

ATOM 1530 H PHE 156 51.590 55.490 61.660 1.00 0.00 H

ATOM 1531 HD1 PHE 156 53.210 50.970 60.920 1.00 0.00 H

ATOM 1532 HD2 PHE 156 51.630 54.430 58.910 1.00 0.00 H

ATOM 1533 HE1 PHE 156 51.540 49.550 59.800 1.00 0.00 H

ATOM 1534 HE2 PHE 156 49.860 53.020 57.900 1.00 0.00 H

ATOM 1535 HZ PHE 156 49.830 50.570 58.320 1.00 0.00 H

ATOM 1536 N LEU 157 53.740 55.500 63.870 1.00 0.00 N

ATOM 1537 CA LEU 157 54.730 55.970 64.860 1.00 0.00 C

ATOM 1538 C LEU 157 54.950 54.860 65.920 1.00 0.00 C

ATOM 1539 O LEU 157 54.010 54.140 66.260 1.00 0.00 O

ATOM 1540 CB LEU 157 54.220 57.260 65.510 1.00 0.00 C

ATOM 1541 CG LEU 157 55.240 57.970 66.390 1.00 0.00 C

ATOM 1542 CD1 LEU 157 56.360 58.600 65.560 1.00 0.00 C

ATOM 1543 CD2 LEU 157 54.540 59.080 67.170 1.00 0.00 C

ATOM 1544 H LEU 157 52.910 56.070 63.800 1.00 0.00 H

ATOM 1545 N SER 158 56.210 54.560 66.170 1.00 0.00 N

ATOM 1546 CA SER 158 56.560 53.470 67.100 1.00 0.00 C

ATOM 1547 C SER 158 57.550 53.810 68.220 1.00 0.00 C

ATOM 1548 O SER 158 58.520 54.550 68.060 1.00 0.00 O

ATOM 1549 CB SER 158 57.060 52.240 66.320 1.00 0.00 C

ATOM 1550 OG SER 158 57.330 51.160 67.220 1.00 0.00 O

ATOM 1551 H SER 158 56.970 54.940 65.620 1.00 0.00 H

ATOM 1552 HG SER 158 57.560 50.320 66.710 1.00 0.00 H

ATOM 1553 N LEU 159 57.110 53.380 69.390 1.00 0.00 N

ATOM 1554 CA LEU 159 57.960 53.160 70.580 1.00 0.00 C

ATOM 1555 C LEU 159 57.690 51.710 71.030 1.00 0.00 C

ATOM 1556 O LEU 159 56.740 51.490 71.770 1.00 0.00 O

ATOM 1557 CB LEU 159 57.580 54.110 71.730 1.00 0.00 C

ATOM 1558 CG LEU 159 57.770 55.610 71.470 1.00 0.00 C

ATOM 1559 CD1 LEU 159 57.140 56.360 72.640 1.00 0.00 C

ATOM 1560 CD2 LEU 159 59.250 55.970 71.360 1.00 0.00 C

ATOM 1561 H LEU 159 56.140 53.170 69.530 1.00 0.00 H

ATOM 1562 N PRO 160 58.500 50.730 70.580 1.00 0.00 N

ATOM 1563 CA PRO 160 58.290 49.310 70.900 1.00 0.00 C

ATOM 1564 C PRO 160 58.190 49.100 72.420 1.00 0.00 C

ATOM 1565 O PRO 160 58.860 49.790 73.200 1.00 0.00 O

ATOM 1566 CB PRO 160 59.500 48.580 70.320 1.00 0.00 C

ATOM 1567 CG PRO 160 59.880 49.460 69.140 1.00 0.00 C

ATOM 1568 CD PRO 160 59.660 50.880 69.690 1.00 0.00 C

ATOM 1569 N ASP 161 57.190 48.290 72.760 1.00 0.00 N

ATOM 1570 CA ASP 161 56.680 47.970 74.110 1.00 0.00 C

ATOM 1571 C ASP 161 56.070 49.100 74.970 1.00 0.00 C

ATOM 1572 O ASP 161 55.720 48.920 76.140 1.00 0.00 O

ATOM 1573 CB ASP 161 57.730 47.170 74.880 1.00 0.00 C

ATOM 1574 CG ASP 161 57.970 45.770 74.320 1.00 0.00 C

ATOM 1575 OD1 ASP 161 57.000 45.120 73.860 1.00 0.00 O

ATOM 1576 OD2 ASP 161 59.100 45.290 74.520 1.00 0.00 O

ATOM 1577 H ASP 161 56.720 47.780 72.030 1.00 0.00 H

ATOM 1578 N ASN 162 55.690 50.180 74.300 1.00 0.00 N

ATOM 1579 CA ASN 162 54.900 51.270 74.890 1.00 0.00 C

ATOM 1580 C ASN 162 53.510 51.350 74.240 1.00 0.00 C

ATOM 1581 O ASN 162 53.310 51.980 73.200 1.00 0.00 O

ATOM 1582 CB ASN 162 55.610 52.630 74.780 1.00 0.00 C

ATOM 1583 CG ASN 162 54.920 53.700 75.630 1.00 0.00 C

ATOM 1584 ND2 ASN 162 55.710 54.590 76.180 1.00 0.00 N

ATOM 1585 OD1 ASN 162 53.750 53.620 75.990 1.00 0.00 O

ATOM 1586 H ASN 162 55.760 50.200 73.300 1.00 0.00 H

ATOM 1587 1HD2 ASN 162 56.710 54.500 76.050 1.00 0.00 H

ATOM 1588 2HD2 ASN 162 55.310 55.330 76.710 1.00 0.00 H

ATOM 1589 N LYS 163 52.540 50.860 75.010 1.00 0.00 N

ATOM 1590 CA LYS 163 51.090 50.920 74.710 1.00 0.00 C

ATOM 1591 C LYS 163 50.550 52.240 74.140 1.00 0.00 C

ATOM 1592 O LYS 163 49.680 52.220 73.280 1.00 0.00 O

ATOM 1593 CB LYS 163 50.280 50.470 75.940 1.00 0.00 C

ATOM 1594 CG LYS 163 50.010 51.500 77.050 1.00 0.00 C

ATOM 1595 CD LYS 163 51.220 52.250 77.620 1.00 0.00 C

ATOM 1596 CE LYS 163 52.230 51.330 78.290 1.00 0.00 C

ATOM 1597 NZ LYS 163 53.540 51.970 78.490 1.00 0.00 N1+

ATOM 1598 H LYS 163 52.790 50.250 75.750 1.00 0.00 H

ATOM 1599 HZ1 LYS 163 54.150 51.310 78.930 1.00 0.00 H

ATOM 1600 HZ2 LYS 163 53.920 52.310 77.630 1.00 0.00 H

ATOM 1601 HZ3 LYS 163 53.450 52.750 79.120 1.00 0.00 H

ATOM 1602 N ASN 164 51.130 53.370 74.550 1.00 0.00 N

ATOM 1603 CA ASN 164 50.710 54.700 74.070 1.00 0.00 C

ATOM 1604 C ASN 164 50.910 54.830 72.550 1.00 0.00 C

ATOM 1605 O ASN 164 49.930 54.900 71.820 1.00 0.00 O

ATOM 1606 CB ASN 164 51.480 55.800 74.800 1.00 0.00 C

ATOM 1607 CG ASN 164 51.370 55.810 76.340 1.00 0.00 C

ATOM 1608 ND2 ASN 164 52.310 56.470 76.970 1.00 0.00 N

ATOM 1609 OD1 ASN 164 50.470 55.260 76.960 1.00 0.00 O

ATOM 1610 H ASN 164 51.930 53.380 75.150 1.00 0.00 H

ATOM 1611 1HD2 ASN 164 53.010 56.950 76.430 1.00 0.00 H

ATOM 1612 2HD2 ASN 164 52.180 56.720 77.920 1.00 0.00 H

ATOM 1613 N ILE 165 52.120 54.480 72.120 1.00 0.00 N

ATOM 1614 CA ILE 165 52.480 54.460 70.680 1.00 0.00 C

ATOM 1615 C ILE 165 53.220 53.140 70.400 1.00 0.00 C

ATOM 1616 O ILE 165 54.450 53.040 70.480 1.00 0.00 O

ATOM 1617 CB ILE 165 53.340 55.670 70.250 1.00 0.00 C

ATOM 1618 CG1 ILE 165 52.830 57.000 70.830 1.00 0.00 C

ATOM 1619 CG2 ILE 165 53.290 55.710 68.730 1.00 0.00 C

ATOM 1620 CD ILE 165 53.740 58.200 70.530 1.00 0.00 C

ATOM 1621 H ILE 165 52.800 54.060 72.720 1.00 0.00 H

ATOM 1622 N ARG 166 52.410 52.120 70.180 1.00 0.00 N

ATOM 1623 CA ARG 166 52.890 50.750 69.940 1.00 0.00 C

ATOM 1624 C ARG 166 53.620 50.490 68.610 1.00 0.00 C

ATOM 1625 O ARG 166 54.680 49.860 68.620 1.00 0.00 O

ATOM 1626 CB ARG 166 51.780 49.730 70.190 1.00 0.00 C

ATOM 1627 CG ARG 166 51.600 49.390 71.680 1.00 0.00 C

ATOM 1628 CD ARG 166 50.950 48.020 71.890 1.00 0.00 C

ATOM 1629 NE ARG 166 51.040 47.610 73.300 1.00 0.00 N

ATOM 1630 CZ ARG 166 51.940 46.760 73.830 1.00 0.00 C

ATOM 1631 NH1 ARG 166 52.890 46.170 73.100 1.00 0.00 N

ATOM 1632 NH2 ARG 166 51.880 46.460 75.130 1.00 0.00 N

ATOM 1633 H ARG 166 51.410 52.200 70.240 1.00 0.00 H

ATOM 1634 HE ARG 166 50.280 47.920 73.890 1.00 0.00 H

ATOM 1635 1HH1 ARG 166 52.960 46.360 72.120 1.00 0.00 H

ATOM 1636 2HH1 ARG 166 53.510 45.490 73.520 1.00 0.00 H

ATOM 1637 1HH2 ARG 166 51.170 46.840 75.740 1.00 0.00 H

ATOM 1638 2HH2 ARG 166 52.480 45.750 75.500 1.00 0.00 H

ATOM 1639 N GLY 167 53.140 51.140 67.560 1.00 0.00 N

ATOM 1640 CA GLY 167 53.520 50.820 66.170 1.00 0.00 C

ATOM 1641 C GLY 167 52.470 49.880 65.580 1.00 0.00 C

ATOM 1642 O GLY 167 51.730 49.210 66.310 1.00 0.00 O

ATOM 1643 H GLY 167 52.360 51.770 67.640 1.00 0.00 H

ATOM 1644 N ASN 168 52.380 49.860 64.250 1.00 0.00 N

ATOM 1645 CA ASN 168 51.400 49.030 63.500 1.00 0.00 C

ATOM 1646 C ASN 168 49.910 49.270 63.850 1.00 0.00 C

ATOM 1647 O ASN 168 49.060 48.430 63.540 1.00 0.00 O

ATOM 1648 CB ASN 168 51.770 47.540 63.640 1.00 0.00 C

ATOM 1649 CG ASN 168 53.150 47.190 63.080 1.00 0.00 C

ATOM 1650 ND2 ASN 168 53.810 46.240 63.710 1.00 0.00 N

ATOM 1651 OD1 ASN 168 53.630 47.750 62.110 1.00 0.00 O

ATOM 1652 H ASN 168 53.130 50.250 63.690 1.00 0.00 H

ATOM 1653 1HD2 ASN 168 53.440 45.800 64.540 1.00 0.00 H

ATOM 1654 2HD2 ASN 168 54.740 46.030 63.420 1.00 0.00 H

ATOM 1655 N ALA 169 49.580 50.480 64.310 1.00 0.00 N

ATOM 1656 CA ALA 169 48.190 50.890 64.610 1.00 0.00 C

ATOM 1657 C ALA 169 47.280 50.790 63.370 1.00 0.00 C

ATOM 1658 O ALA 169 46.260 50.110 63.450 1.00 0.00 O

ATOM 1659 CB ALA 169 48.140 52.320 65.170 1.00 0.00 C

ATOM 1660 H ALA 169 50.280 51.190 64.460 1.00 0.00 H

ATOM 1661 N GLY 170 47.810 51.210 62.220 1.00 0.00 N

ATOM 1662 CA GLY 170 47.150 51.080 60.900 1.00 0.00 C

ATOM 1663 C GLY 170 46.800 49.630 60.520 1.00 0.00 C

ATOM 1664 O GLY 170 45.690 49.320 60.100 1.00 0.00 O

ATOM 1665 H GLY 170 48.660 51.740 62.210 1.00 0.00 H

ATOM 1666 N LEU 171 47.740 48.730 60.800 1.00 0.00 N

ATOM 1667 CA LEU 171 47.560 47.280 60.590 1.00 0.00 C

ATOM 1668 C LEU 171 46.530 46.660 61.550 1.00 0.00 C

ATOM 1669 O LEU 171 45.740 45.800 61.160 1.00 0.00 O

ATOM 1670 CB LEU 171 48.900 46.550 60.720 1.00 0.00 C

ATOM 1671 CG LEU 171 49.890 47.010 59.640 1.00 0.00 C

ATOM 1672 CD1 LEU 171 51.290 46.530 60.030 1.00 0.00 C

ATOM 1673 CD2 LEU 171 49.540 46.450 58.260 1.00 0.00 C

ATOM 1674 H LEU 171 48.620 49.000 61.190 1.00 0.00 H

ATOM 1675 N LEU 172 46.530 47.160 62.790 1.00 0.00 N

ATOM 1676 CA LEU 172 45.500 46.820 63.790 1.00 0.00 C

ATOM 1677 C LEU 172 44.100 47.300 63.390 1.00 0.00 C

ATOM 1678 O LEU 172 43.150 46.540 63.520 1.00 0.00 O

ATOM 1679 CB LEU 172 45.880 47.400 65.160 1.00 0.00 C

ATOM 1680 CG LEU 172 47.170 46.820 65.760 1.00 0.00 C

ATOM 1681 CD1 LEU 172 47.520 47.610 67.020 1.00 0.00 C

ATOM 1682 CD2 LEU 172 47.010 45.340 66.110 1.00 0.00 C

ATOM 1683 H LEU 172 47.260 47.770 63.110 1.00 0.00 H

ATOM 1684 N ASP 173 44.010 48.510 62.820 1.00 0.00 N

ATOM 1685 CA ASP 173 42.770 49.060 62.230 1.00 0.00 C

ATOM 1686 C ASP 173 42.190 48.120 61.160 1.00 0.00 C

ATOM 1687 O ASP 173 41.090 47.600 61.320 1.00 0.00 O

ATOM 1688 CB ASP 173 43.030 50.420 61.590 1.00 0.00 C

ATOM 1689 CG ASP 173 43.460 51.520 62.560 1.00 0.00 C

ATOM 1690 OD1 ASP 173 43.020 51.480 63.730 1.00 0.00 O

ATOM 1691 OD2 ASP 173 44.200 52.410 62.120 1.00 0.00 O

ATOM 1692 H ASP 173 44.740 49.200 62.920 1.00 0.00 H

ATOM 1693 N GLN 174 43.070 47.720 60.240 1.00 0.00 N

ATOM 1694 CA GLN 174 42.780 46.710 59.200 1.00 0.00 C

ATOM 1695 C GLN 174 42.240 45.400 59.810 1.00 0.00 C

ATOM 1696 O GLN 174 41.140 44.970 59.490 1.00 0.00 O

ATOM 1697 CB GLN 174 44.040 46.390 58.390 1.00 0.00 C

ATOM 1698 CG GLN 174 44.510 47.510 57.460 1.00 0.00 C

ATOM 1699 CD GLN 174 45.820 47.110 56.760 1.00 0.00 C

ATOM 1700 NE2 GLN 174 46.560 48.080 56.270 1.00 0.00 N

ATOM 1701 OE1 GLN 174 46.170 45.940 56.640 1.00 0.00 O

ATOM 1702 H GLN 174 43.950 48.210 60.130 1.00 0.00 H

ATOM 1703 1HE2 GLN 174 46.290 49.040 56.320 1.00 0.00 H

ATOM 1704 2HE2 GLN 174 47.390 47.810 55.760 1.00 0.00 H

ATOM 1705 N SER 175 42.960 44.890 60.820 1.00 0.00 N

ATOM 1706 CA SER 175 42.560 43.680 61.570 1.00 0.00 C

ATOM 1707 C SER 175 41.200 43.810 62.270 1.00 0.00 C

ATOM 1708 O SER 175 40.420 42.860 62.290 1.00 0.00 O

ATOM 1709 CB SER 175 43.570 43.300 62.650 1.00 0.00 C

ATOM 1710 OG SER 175 44.830 42.940 62.070 1.00 0.00 O

ATOM 1711 H SER 175 43.860 45.270 61.060 1.00 0.00 H

ATOM 1712 HG SER 175 45.530 43.140 62.760 1.00 0.00 H

ATOM 1713 N LEU 176 40.920 44.990 62.840 1.00 0.00 N

ATOM 1714 CA LEU 176 39.620 45.260 63.480 1.00 0.00 C

ATOM 1715 C LEU 176 38.450 45.290 62.480 1.00 0.00 C

ATOM 1716 O LEU 176 37.410 44.700 62.760 1.00 0.00 O

ATOM 1717 CB LEU 176 39.640 46.520 64.360 1.00 0.00 C

ATOM 1718 CG LEU 176 38.320 46.740 65.110 1.00 0.00 C

ATOM 1719 CD1 LEU 176 38.000 45.610 66.100 1.00 0.00 C

ATOM 1720 CD2 LEU 176 38.320 48.100 65.810 1.00 0.00 C

ATOM 1721 H LEU 176 41.570 45.760 62.820 1.00 0.00 H

ATOM 1722 N ALA 177 38.670 45.850 61.290 1.00 0.00 N

ATOM 1723 CA ALA 177 37.680 45.740 60.190 1.00 0.00 C

ATOM 1724 C ALA 177 37.470 44.280 59.760 1.00 0.00 C

ATOM 1725 O ALA 177 36.330 43.850 59.610 1.00 0.00 O

ATOM 1726 CB ALA 177 38.100 46.550 58.980 1.00 0.00 C

ATOM 1727 H ALA 177 39.450 46.460 61.110 1.00 0.00 H

ATOM 1728 N LEU 178 38.560 43.510 59.710 1.00 0.00 N

ATOM 1729 CA LEU 178 38.480 42.050 59.450 1.00 0.00 C

ATOM 1730 C LEU 178 37.650 41.330 60.540 1.00 0.00 C

ATOM 1731 O LEU 178 36.680 40.660 60.220 1.00 0.00 O

ATOM 1732 CB LEU 178 39.870 41.400 59.330 1.00 0.00 C

ATOM 1733 CG LEU 178 40.910 42.120 58.450 1.00 0.00 C

ATOM 1734 CD1 LEU 178 42.140 41.220 58.310 1.00 0.00 C

ATOM 1735 CD2 LEU 178 40.410 42.560 57.080 1.00 0.00 C

ATOM 1736 H LEU 178 39.480 43.900 59.690 1.00 0.00 H

ATOM 1737 N ARG 179 37.870 41.720 61.800 1.00 0.00 N

ATOM 1738 CA ARG 179 37.040 41.270 62.950 1.00 0.00 C

ATOM 1739 C ARG 179 35.550 41.570 62.720 1.00 0.00 C

ATOM 1740 O ARG 179 34.710 40.680 62.870 1.00 0.00 O

ATOM 1741 CB ARG 179 37.390 41.950 64.280 1.00 0.00 C

ATOM 1742 CG ARG 179 38.780 41.620 64.800 1.00 0.00 C

ATOM 1743 CD ARG 179 38.750 41.460 66.320 1.00 0.00 C

ATOM 1744 NE ARG 179 40.100 41.130 66.830 1.00 0.00 N

ATOM 1745 CZ ARG 179 41.170 41.940 66.800 1.00 0.00 C

ATOM 1746 NH1 ARG 179 41.100 43.170 66.290 1.00 0.00 N

ATOM 1747 NH2 ARG 179 42.320 41.550 67.340 1.00 0.00 N

ATOM 1748 H ARG 179 38.680 42.260 62.040 1.00 0.00 H

ATOM 1749 HE ARG 179 40.180 40.220 67.250 1.00 0.00 H

ATOM 1750 1HH1 ARG 179 40.260 43.490 65.860 1.00 0.00 H

ATOM 1751 2HH1 ARG 179 41.830 43.830 66.510 1.00 0.00 H

ATOM 1752 1HH2 ARG 179 42.410 40.700 67.860 1.00 0.00 H

ATOM 1753 2HH2 ARG 179 43.140 42.130 67.200 1.00 0.00 H

ATOM 1754 N TRP 180 35.280 42.810 62.320 1.00 0.00 N

ATOM 1755 CA TRP 180 33.920 43.300 62.030 1.00 0.00 C

ATOM 1756 C TRP 180 33.240 42.470 60.920 1.00 0.00 C

ATOM 1757 O TRP 180 32.090 42.080 61.090 1.00 0.00 O

ATOM 1758 CB TRP 180 33.990 44.770 61.610 1.00 0.00 C

ATOM 1759 CG TRP 180 32.590 45.370 61.550 1.00 0.00 C

ATOM 1760 CD1 TRP 180 31.970 45.960 62.560 1.00 0.00 C

ATOM 1761 CD2 TRP 180 31.710 45.320 60.470 1.00 0.00 C

ATOM 1762 CE2 TRP 180 30.550 45.920 60.920 1.00 0.00 C

ATOM 1763 CE3 TRP 180 31.800 44.840 59.180 1.00 0.00 C

ATOM 1764 NE1 TRP 180 30.730 46.280 62.170 1.00 0.00 N

ATOM 1765 CZ2 TRP 180 29.470 46.070 60.050 1.00 0.00 C

ATOM 1766 CZ3 TRP 180 30.720 44.960 58.320 1.00 0.00 C

ATOM 1767 CH2 TRP 180 29.560 45.590 58.750 1.00 0.00 C

ATOM 1768 H TRP 180 36.000 43.500 62.230 1.00 0.00 H

ATOM 1769 HD1 TRP 180 32.420 46.220 63.510 1.00 0.00 H

ATOM 1770 HE1 TRP 180 30.020 46.660 62.760 1.00 0.00 H

ATOM 1771 HE3 TRP 180 32.720 44.360 58.830 1.00 0.00 H

ATOM 1772 HZ2 TRP 180 28.560 46.580 60.380 1.00 0.00 H

ATOM 1773 HZ3 TRP 180 30.770 44.550 57.310 1.00 0.00 H

ATOM 1774 HH2 TRP 180 28.740 45.770 58.050 1.00 0.00 H

ATOM 1775 N VAL 181 33.980 42.160 59.860 1.00 0.00 N

ATOM 1776 CA VAL 181 33.510 41.290 58.760 1.00 0.00 C

ATOM 1777 C VAL 181 33.170 39.890 59.310 1.00 0.00 C

ATOM 1778 O VAL 181 31.990 39.530 59.270 1.00 0.00 O

ATOM 1779 CB VAL 181 34.520 41.270 57.590 1.00 0.00 C

ATOM 1780 CG1 VAL 181 34.040 40.400 56.430 1.00 0.00 C

ATOM 1781 CG2 VAL 181 34.790 42.670 57.040 1.00 0.00 C

ATOM 1782 H VAL 181 34.900 42.560 59.730 1.00 0.00 H

ATOM 1783 N SER 182 34.090 39.270 60.040 1.00 0.00 N

ATOM 1784 CA SER 182 33.860 37.980 60.740 1.00 0.00 C

ATOM 1785 C SER 182 32.580 37.990 61.600 1.00 0.00 C

ATOM 1786 O SER 182 31.780 37.060 61.550 1.00 0.00 O

ATOM 1787 CB SER 182 35.020 37.600 61.660 1.00 0.00 C

ATOM 1788 OG SER 182 36.280 37.580 60.980 1.00 0.00 O

ATOM 1789 H SER 182 35.060 39.560 60.020 1.00 0.00 H

ATOM 1790 HG SER 182 36.760 36.730 61.190 1.00 0.00 H

ATOM 1791 N SER 183 32.420 39.060 62.390 1.00 0.00 N

ATOM 1792 CA SER 183 31.270 39.310 63.280 1.00 0.00 C

ATOM 1793 C SER 183 29.930 39.560 62.560 1.00 0.00 C

ATOM 1794 O SER 183 28.920 38.940 62.920 1.00 0.00 O

ATOM 1795 CB SER 183 31.540 40.520 64.180 1.00 0.00 C

ATOM 1796 OG SER 183 32.580 40.300 65.140 1.00 0.00 O

ATOM 1797 H SER 183 33.160 39.730 62.470 1.00 0.00 H

ATOM 1798 HG SER 183 32.180 39.800 65.920 1.00 0.00 H

ATOM 1799 N ASN 184 29.950 40.420 61.550 1.00 0.00 N

ATOM 1800 CA ASN 184 28.740 40.990 60.920 1.00 0.00 C

ATOM 1801 C ASN 184 28.360 40.560 59.500 1.00 0.00 C

ATOM 1802 O ASN 184 27.200 40.690 59.130 1.00 0.00 O

ATOM 1803 CB ASN 184 28.790 42.520 60.940 1.00 0.00 C

ATOM 1804 CG ASN 184 28.700 43.040 62.370 1.00 0.00 C

ATOM 1805 ND2 ASN 184 29.820 43.410 62.930 1.00 0.00 N

ATOM 1806 OD1 ASN 184 27.630 43.150 62.940 1.00 0.00 O

ATOM 1807 H ASN 184 30.810 40.870 61.270 1.00 0.00 H

ATOM 1808 1HD2 ASN 184 30.660 43.450 62.390 1.00 0.00 H

ATOM 1809 2HD2 ASN 184 29.850 43.500 63.930 1.00 0.00 H

ATOM 1810 N ILE 185 29.310 40.070 58.700 1.00 0.00 N

ATOM 1811 CA ILE 185 29.080 39.830 57.260 1.00 0.00 C

ATOM 1812 C ILE 185 27.900 38.890 56.930 1.00 0.00 C

ATOM 1813 O ILE 185 27.190 39.130 55.950 1.00 0.00 O

ATOM 1814 CB ILE 185 30.380 39.450 56.530 1.00 0.00 C

ATOM 1815 CG1 ILE 185 30.270 39.780 55.040 1.00 0.00 C

ATOM 1816 CG2 ILE 185 30.820 37.990 56.720 1.00 0.00 C

ATOM 1817 CD ILE 185 30.220 41.290 54.780 1.00 0.00 C

ATOM 1818 H ILE 185 30.220 39.810 59.040 1.00 0.00 H

ATOM 1819 N ALA 186 27.610 37.950 57.830 1.00 0.00 N

ATOM 1820 CA ALA 186 26.420 37.080 57.710 1.00 0.00 C

ATOM 1821 C ALA 186 25.100 37.890 57.650 1.00 0.00 C

ATOM 1822 O ALA 186 24.260 37.620 56.800 1.00 0.00 O

ATOM 1823 CB ALA 186 26.400 36.080 58.860 1.00 0.00 C

ATOM 1824 H ALA 186 28.240 37.730 58.590 1.00 0.00 H

ATOM 1825 N ALA 187 25.050 39.020 58.350 1.00 0.00 N

ATOM 1826 CA ALA 187 23.880 39.930 58.340 1.00 0.00 C

ATOM 1827 C ALA 187 23.630 40.550 56.950 1.00 0.00 C

ATOM 1828 O ALA 187 22.480 40.640 56.500 1.00 0.00 O

ATOM 1829 CB ALA 187 24.060 41.020 59.400 1.00 0.00 C

ATOM 1830 H ALA 187 25.840 39.350 58.880 1.00 0.00 H

ATOM 1831 N PHE 188 24.720 40.750 56.210 1.00 0.00 N

ATOM 1832 CA PHE 188 24.760 41.220 54.810 1.00 0.00 C

ATOM 1833 C PHE 188 24.530 40.130 53.750 1.00 0.00 C

ATOM 1834 O PHE 188 24.380 40.440 52.570 1.00 0.00 O

ATOM 1835 CB PHE 188 26.110 41.900 54.560 1.00 0.00 C

ATOM 1836 CG PHE 188 26.250 43.210 55.350 1.00 0.00 C

ATOM 1837 CD1 PHE 188 26.630 43.190 56.680 1.00 0.00 C

ATOM 1838 CD2 PHE 188 25.840 44.410 54.760 1.00 0.00 C

ATOM 1839 CE1 PHE 188 26.560 44.350 57.440 1.00 0.00 C

ATOM 1840 CE2 PHE 188 25.780 45.570 55.520 1.00 0.00 C

ATOM 1841 CZ PHE 188 26.140 45.540 56.860 1.00 0.00 C

ATOM 1842 H PHE 188 25.630 40.560 56.590 1.00 0.00 H

ATOM 1843 HD1 PHE 188 26.990 42.270 57.140 1.00 0.00 H

ATOM 1844 HD2 PHE 188 25.510 44.420 53.720 1.00 0.00 H

ATOM 1845 HE1 PHE 188 26.820 44.320 58.500 1.00 0.00 H

ATOM 1846 HE2 PHE 188 25.360 46.470 55.090 1.00 0.00 H

ATOM 1847 HZ PHE 188 26.050 46.440 57.470 1.00 0.00 H

ATOM 1848 N GLY 189 24.560 38.870 54.190 1.00 0.00 N

ATOM 1849 CA GLY 189 24.470 37.670 53.320 1.00 0.00 C

ATOM 1850 C GLY 189 25.820 37.160 52.780 1.00 0.00 C

ATOM 1851 O GLY 189 25.870 36.410 51.810 1.00 0.00 O

ATOM 1852 H GLY 189 24.520 38.660 55.160 1.00 0.00 H

ATOM 1853 N GLY 190 26.880 37.480 53.520 1.00 0.00 N

ATOM 1854 CA GLY 190 28.260 37.060 53.190 1.00 0.00 C

ATOM 1855 C GLY 190 28.720 35.880 54.060 1.00 0.00 C

ATOM 1856 O GLY 190 28.210 35.650 55.160 1.00 0.00 O

ATOM 1857 H GLY 190 26.800 38.070 54.320 1.00 0.00 H

ATOM 1858 N ASP 191 29.670 35.140 53.500 1.00 0.00 N

ATOM 1859 CA ASP 191 30.320 33.990 54.170 1.00 0.00 C

ATOM 1860 C ASP 191 31.570 34.430 54.940 1.00 0.00 C

ATOM 1861 O ASP 191 32.590 34.760 54.310 1.00 0.00 O

ATOM 1862 CB ASP 191 30.700 32.870 53.190 1.00 0.00 C

ATOM 1863 CG ASP 191 30.820 31.510 53.900 1.00 0.00 C

ATOM 1864 OD1 ASP 191 31.100 31.510 55.110 1.00 0.00 O

ATOM 1865 OD2 ASP 191 30.600 30.470 53.250 1.00 0.00 O

ATOM 1866 H ASP 191 29.940 35.280 52.540 1.00 0.00 H

ATOM 1867 N PRO 192 31.530 34.420 56.280 1.00 0.00 N

ATOM 1868 CA PRO 192 32.730 34.620 57.110 1.00 0.00 C

ATOM 1869 C PRO 192 33.820 33.560 56.850 1.00 0.00 C

ATOM 1870 O PRO 192 35.010 33.870 56.910 1.00 0.00 O

ATOM 1871 CB PRO 192 32.220 34.610 58.550 1.00 0.00 C

ATOM 1872 CG PRO 192 30.960 33.750 58.470 1.00 0.00 C

ATOM 1873 CD PRO 192 30.350 34.120 57.120 1.00 0.00 C

ATOM 1874 N SER 193 33.410 32.360 56.440 1.00 0.00 N

ATOM 1875 CA SER 193 34.310 31.250 56.030 1.00 0.00 C

ATOM 1876 C SER 193 34.970 31.510 54.670 1.00 0.00 C

ATOM 1877 O SER 193 36.150 31.200 54.500 1.00 0.00 O

ATOM 1878 CB SER 193 33.500 29.950 55.980 1.00 0.00 C

ATOM 1879 OG SER 193 34.310 28.780 55.830 1.00 0.00 O

ATOM 1880 H SER 193 32.430 32.160 56.240 1.00 0.00 H

ATOM 1881 HG SER 193 33.910 28.090 56.450 1.00 0.00 H

ATOM 1882 N LYS 194 34.270 32.190 53.750 1.00 0.00 N

ATOM 1883 CA LYS 194 34.820 32.560 52.430 1.00 0.00 C

ATOM 1884 C LYS 194 35.050 34.080 52.300 1.00 0.00 C

ATOM 1885 O LYS 194 34.390 34.800 51.550 1.00 0.00 O

ATOM 1886 CB LYS 194 33.970 32.090 51.240 1.00 0.00 C

ATOM 1887 CG LYS 194 34.020 30.600 50.890 1.00 0.00 C

ATOM 1888 CD LYS 194 33.220 29.790 51.900 1.00 0.00 C

ATOM 1889 CE LYS 194 32.840 28.400 51.420 1.00 0.00 C

ATOM 1890 NZ LYS 194 32.050 27.760 52.480 1.00 0.00 N1+

ATOM 1891 H LYS 194 33.330 32.490 53.930 1.00 0.00 H

ATOM 1892 HZ1 LYS 194 31.920 26.790 52.270 1.00 0.00 H

ATOM 1893 HZ2 LYS 194 32.540 27.860 53.340 1.00 0.00 H

ATOM 1894 HZ3 LYS 194 31.170 28.230 52.550 1.00 0.00 H

ATOM 1895 N VAL 195 36.130 34.510 52.940 1.00 0.00 N

ATOM 1896 CA VAL 195 36.650 35.880 52.780 1.00 0.00 C

ATOM 1897 C VAL 195 38.000 35.800 52.050 1.00 0.00 C

ATOM 1898 O VAL 195 38.830 34.940 52.360 1.00 0.00 O

ATOM 1899 CB VAL 195 36.790 36.580 54.150 1.00 0.00 C

ATOM 1900 CG1 VAL 195 37.310 38.020 54.020 1.00 0.00 C

ATOM 1901 CG2 VAL 195 35.470 36.630 54.920 1.00 0.00 C

ATOM 1902 H VAL 195 36.620 33.930 53.590 1.00 0.00 H

ATOM 1903 N THR 196 38.210 36.690 51.080 1.00 0.00 N

ATOM 1904 CA THR 196 39.520 36.930 50.440 1.00 0.00 C

ATOM 1905 C THR 196 39.940 38.400 50.590 1.00 0.00 C

ATOM 1906 O THR 196 39.150 39.320 50.400 1.00 0.00 O

ATOM 1907 CB THR 196 39.640 36.470 48.980 1.00 0.00 C

ATOM 1908 CG2 THR 196 41.100 36.510 48.510 1.00 0.00 C

ATOM 1909 OG1 THR 196 39.200 35.110 48.860 1.00 0.00 O

ATOM 1910 H THR 196 37.430 37.220 50.720 1.00 0.00 H

ATOM 1911 HG1 THR 196 39.210 34.890 47.880 1.00 0.00 H

ATOM 1912 N LEU 197 41.170 38.560 51.070 1.00 0.00 N

ATOM 1913 CA LEU 197 41.830 39.870 51.190 1.00 0.00 C

ATOM 1914 C LEU 197 42.680 40.110 49.950 1.00 0.00 C

ATOM 1915 O LEU 197 43.440 39.240 49.520 1.00 0.00 O

ATOM 1916 CB LEU 197 42.790 39.940 52.390 1.00 0.00 C

ATOM 1917 CG LEU 197 42.210 39.620 53.770 1.00 0.00 C

ATOM 1918 CD1 LEU 197 43.300 39.860 54.820 1.00 0.00 C

ATOM 1919 CD2 LEU 197 40.970 40.440 54.110 1.00 0.00 C

ATOM 1920 H LEU 197 41.700 37.780 51.420 1.00 0.00 H

ATOM 1921 N PHE 198 42.460 41.270 49.370 1.00 0.00 N

ATOM 1922 CA PHE 198 43.260 41.750 48.240 1.00 0.00 C

ATOM 1923 C PHE 198 43.550 43.240 48.390 1.00 0.00 C

ATOM 1924 O PHE 198 42.810 43.970 49.060 1.00 0.00 O

ATOM 1925 CB PHE 198 42.590 41.400 46.900 1.00 0.00 C

ATOM 1926 CG PHE 198 41.340 42.190 46.530 1.00 0.00 C

ATOM 1927 CD1 PHE 198 40.230 42.240 47.370 1.00 0.00 C

ATOM 1928 CD2 PHE 198 41.370 42.950 45.360 1.00 0.00 C

ATOM 1929 CE1 PHE 198 39.180 43.080 47.060 1.00 0.00 C

ATOM 1930 CE2 PHE 198 40.310 43.780 45.050 1.00 0.00 C

ATOM 1931 CZ PHE 198 39.230 43.870 45.910 1.00 0.00 C

ATOM 1932 H PHE 198 41.800 41.940 49.740 1.00 0.00 H

ATOM 1933 HD1 PHE 198 40.160 41.570 48.220 1.00 0.00 H

ATOM 1934 HD2 PHE 198 42.250 42.920 44.710 1.00 0.00 H

ATOM 1935 HE1 PHE 198 38.320 43.150 47.730 1.00 0.00 H

ATOM 1936 HE2 PHE 198 40.380 44.450 44.190 1.00 0.00 H

ATOM 1937 HZ PHE 198 38.470 44.640 45.740 1.00 0.00 H

ATOM 1938 N GLY 199 44.710 43.590 47.880 1.00 0.00 N

ATOM 1939 CA GLY 199 45.260 44.950 47.990 1.00 0.00 C

ATOM 1940 C GLY 199 46.420 45.120 47.010 1.00 0.00 C

ATOM 1941 O GLY 199 46.980 44.130 46.510 1.00 0.00 O

ATOM 1942 H GLY 199 45.290 42.950 47.380 1.00 0.00 H

ATOM 1943 N GLU 200 46.710 46.380 46.740 1.00 0.00 N

ATOM 1944 CA GLU 200 47.810 46.780 45.850 1.00 0.00 C

ATOM 1945 C GLU 200 48.840 47.650 46.580 1.00 0.00 C

ATOM 1946 O GLU 200 48.530 48.250 47.620 1.00 0.00 O

ATOM 1947 CB GLU 200 47.170 47.430 44.620 1.00 0.00 C

ATOM 1948 CG GLU 200 48.160 47.920 43.550 1.00 0.00 C

ATOM 1949 CD GLU 200 48.850 49.240 43.920 1.00 0.00 C

ATOM 1950 OE1 GLU 200 48.310 50.020 44.740 1.00 0.00 O

ATOM 1951 OE2 GLU 200 49.980 49.410 43.420 1.00 0.00 O

ATOM 1952 H GLU 200 46.160 47.140 47.090 1.00 0.00 H

ATOM 1953 N SER 201 50.100 47.390 46.240 1.00 0.00 N

ATOM 1954 CA SER 201 51.310 48.060 46.770 1.00 0.00 C

ATOM 1955 C SER 201 51.410 47.940 48.300 1.00 0.00 C

ATOM 1956 O SER 201 51.550 46.830 48.820 1.00 0.00 O

ATOM 1957 CB SER 201 51.330 49.500 46.240 1.00 0.00 C

ATOM 1958 OG SER 201 52.620 50.120 46.380 1.00 0.00 O

ATOM 1959 H SER 201 50.270 46.790 45.460 1.00 0.00 H

ATOM 1960 HG SER 201 52.800 50.550 45.470 1.00 0.00 H

ATOM 1961 N ALA 202 51.230 49.040 49.040 1.00 0.00 N

ATOM 1962 CA ALA 202 51.170 49.020 50.520 1.00 0.00 C

ATOM 1963 C ALA 202 50.020 48.160 51.070 1.00 0.00 C

ATOM 1964 O ALA 202 50.190 47.440 52.060 1.00 0.00 O

ATOM 1965 CB ALA 202 51.050 50.440 51.080 1.00 0.00 C

ATOM 1966 H ALA 202 51.240 49.940 48.610 1.00 0.00 H

ATOM 1967 N GLY 203 48.900 48.160 50.360 1.00 0.00 N

ATOM 1968 CA GLY 203 47.730 47.300 50.630 1.00 0.00 C

ATOM 1969 C GLY 203 48.060 45.810 50.440 1.00 0.00 C

ATOM 1970 O GLY 203 47.830 45.010 51.340 1.00 0.00 O

ATOM 1971 H GLY 203 48.810 48.700 49.520 1.00 0.00 H

ATOM 1972 N ALA 204 48.770 45.500 49.350 1.00 0.00 N

ATOM 1973 CA ALA 204 49.270 44.140 49.080 1.00 0.00 C

ATOM 1974 C ALA 204 50.290 43.670 50.130 1.00 0.00 C

ATOM 1975 O ALA 204 50.100 42.630 50.760 1.00 0.00 O

ATOM 1976 CB ALA 204 49.880 44.090 47.690 1.00 0.00 C

ATOM 1977 H ALA 204 48.970 46.180 48.640 1.00 0.00 H

ATOM 1978 N ALA 205 51.220 44.560 50.480 1.00 0.00 N

ATOM 1979 CA ALA 205 52.160 44.350 51.600 1.00 0.00 C

ATOM 1980 C ALA 205 51.400 44.080 52.910 1.00 0.00 C

ATOM 1981 O ALA 205 51.620 43.030 53.530 1.00 0.00 O

ATOM 1982 CB ALA 205 53.080 45.570 51.750 1.00 0.00 C

ATOM 1983 H ALA 205 51.380 45.390 49.920 1.00 0.00 H

ATOM 1984 N SER 206 50.370 44.880 53.180 1.00 0.00 N

ATOM 1985 CA SER 206 49.420 44.700 54.310 1.00 0.00 C

ATOM 1986 C SER 206 48.770 43.310 54.320 1.00 0.00 C

ATOM 1987 O SER 206 48.770 42.640 55.350 1.00 0.00 O

ATOM 1988 CB SER 206 48.300 45.740 54.310 1.00 0.00 C

ATOM 1989 OG SER 206 48.760 47.060 54.630 1.00 0.00 O

ATOM 1990 H SER 206 50.240 45.740 52.670 1.00 0.00 H

ATOM 1991 HG SER 206 48.810 47.620 53.790 1.00 0.00 H

ATOM 1992 N VAL 207 48.300 42.850 53.160 1.00 0.00 N

ATOM 1993 CA VAL 207 47.710 41.500 52.970 1.00 0.00 C

ATOM 1994 C VAL 207 48.730 40.400 53.360 1.00 0.00 C

ATOM 1995 O VAL 207 48.430 39.560 54.210 1.00 0.00 O

ATOM 1996 CB VAL 207 47.200 41.320 51.520 1.00 0.00 C

ATOM 1997 CG1 VAL 207 46.560 39.950 51.290 1.00 0.00 C

ATOM 1998 CG2 VAL 207 46.150 42.370 51.140 1.00 0.00 C

ATOM 1999 H VAL 207 48.280 43.440 52.340 1.00 0.00 H

ATOM 2000 N GLY 208 49.980 40.570 52.900 1.00 0.00 N

ATOM 2001 CA GLY 208 51.130 39.720 53.280 1.00 0.00 C

ATOM 2002 C GLY 208 51.410 39.700 54.800 1.00 0.00 C

ATOM 2003 O GLY 208 51.580 38.630 55.390 1.00 0.00 O

ATOM 2004 H GLY 208 50.170 41.270 52.200 1.00 0.00 H

ATOM 2005 N PHE 209 51.340 40.870 55.430 1.00 0.00 N

ATOM 2006 CA PHE 209 51.410 40.980 56.910 1.00 0.00 C

ATOM 2007 C PHE 209 50.270 40.250 57.640 1.00 0.00 C

ATOM 2008 O PHE 209 50.490 39.600 58.660 1.00 0.00 O

ATOM 2009 CB PHE 209 51.430 42.440 57.370 1.00 0.00 C

ATOM 2010 CG PHE 209 52.750 43.130 57.010 1.00 0.00 C

ATOM 2011 CD1 PHE 209 53.910 42.830 57.700 1.00 0.00 C

ATOM 2012 CD2 PHE 209 52.770 44.050 55.970 1.00 0.00 C

ATOM 2013 CE1 PHE 209 55.100 43.440 57.350 1.00 0.00 C

ATOM 2014 CE2 PHE 209 53.970 44.650 55.600 1.00 0.00 C

ATOM 2015 CZ PHE 209 55.130 44.350 56.290 1.00 0.00 C

ATOM 2016 H PHE 209 51.360 41.740 54.920 1.00 0.00 H

ATOM 2017 HD1 PHE 209 53.880 42.130 58.540 1.00 0.00 H

ATOM 2018 HD2 PHE 209 51.840 44.390 55.530 1.00 0.00 H

ATOM 2019 HE1 PHE 209 56.010 43.230 57.900 1.00 0.00 H

ATOM 2020 HE2 PHE 209 54.010 45.290 54.720 1.00 0.00 H

ATOM 2021 HZ PHE 209 56.070 44.790 55.970 1.00 0.00 H

ATOM 2022 N HIS 210 49.060 40.320 57.080 1.00 0.00 N

ATOM 2023 CA HIS 210 47.920 39.520 57.570 1.00 0.00 C

ATOM 2024 C HIS 210 48.170 38.000 57.420 1.00 0.00 C

ATOM 2025 O HIS 210 47.740 37.230 58.270 1.00 0.00 O

ATOM 2026 CB HIS 210 46.590 39.920 56.930 1.00 0.00 C

ATOM 2027 CG HIS 210 46.120 41.310 57.380 1.00 0.00 C

ATOM 2028 CD2 HIS 210 46.130 42.400 56.620 1.00 0.00 C

ATOM 2029 ND1 HIS 210 45.720 41.670 58.590 1.00 0.00 N

ATOM 2030 CE1 HIS 210 45.520 42.990 58.590 1.00 0.00 C

ATOM 2031 NE2 HIS 210 45.800 43.440 57.370 1.00 0.00 N1+

ATOM 2032 H HIS 210 48.900 40.900 56.270 1.00 0.00 H

ATOM 2033 HD1 HIS 210 45.630 41.060 59.390 1.00 0.00 H

ATOM 2034 HE2 HIS 210 45.890 44.420 57.090 1.00 0.00 H

ATOM 2035 N LEU 211 48.880 37.600 56.370 1.00 0.00 N

ATOM 2036 CA LEU 211 49.380 36.220 56.220 1.00 0.00 C

ATOM 2037 C LEU 211 50.380 35.820 57.320 1.00 0.00 C

ATOM 2038 O LEU 211 50.400 34.670 57.720 1.00 0.00 O

ATOM 2039 CB LEU 211 50.060 35.980 54.870 1.00 0.00 C

ATOM 2040 CG LEU 211 49.130 36.090 53.650 1.00 0.00 C

ATOM 2041 CD1 LEU 211 49.960 35.930 52.380 1.00 0.00 C

ATOM 2042 CD2 LEU 211 48.070 34.990 53.680 1.00 0.00 C

ATOM 2043 H LEU 211 49.040 38.200 55.580 1.00 0.00 H

ATOM 2044 N LEU 212 51.260 36.740 57.700 1.00 0.00 N

ATOM 2045 CA LEU 212 52.270 36.480 58.760 1.00 0.00 C

ATOM 2046 C LEU 212 51.720 36.470 60.190 1.00 0.00 C

ATOM 2047 O LEU 212 51.970 35.540 60.960 1.00 0.00 O

ATOM 2048 CB LEU 212 53.410 37.500 58.650 1.00 0.00 C

ATOM 2049 CG LEU 212 54.130 37.450 57.290 1.00 0.00 C

ATOM 2050 CD1 LEU 212 55.100 38.620 57.230 1.00 0.00 C

ATOM 2051 CD2 LEU 212 54.870 36.130 57.090 1.00 0.00 C

ATOM 2052 H LEU 212 51.420 37.570 57.160 1.00 0.00 H

ATOM 2053 N SER 213 50.920 37.490 60.510 1.00 0.00 N

ATOM 2054 CA SER 213 50.360 37.730 61.850 1.00 0.00 C

ATOM 2055 C SER 213 49.500 36.550 62.370 1.00 0.00 C

ATOM 2056 O SER 213 48.420 36.320 61.820 1.00 0.00 O

ATOM 2057 CB SER 213 49.510 39.000 61.840 1.00 0.00 C

ATOM 2058 OG SER 213 49.030 39.340 63.150 1.00 0.00 O

ATOM 2059 H SER 213 50.700 38.190 59.820 1.00 0.00 H

ATOM 2060 HG SER 213 49.770 39.860 63.600 1.00 0.00 H

ATOM 2061 N PRO 214 49.910 35.940 63.490 1.00 0.00 N

ATOM 2062 CA PRO 214 49.090 34.940 64.200 1.00 0.00 C

ATOM 2063 C PRO 214 47.690 35.480 64.520 1.00 0.00 C

ATOM 2064 O PRO 214 46.710 34.870 64.100 1.00 0.00 O

ATOM 2065 CB PRO 214 49.880 34.620 65.470 1.00 0.00 C

ATOM 2066 CG PRO 214 51.320 34.770 65.000 1.00 0.00 C

ATOM 2067 CD PRO 214 51.260 36.000 64.090 1.00 0.00 C

ATOM 2068 N ALA 215 47.620 36.750 64.910 1.00 0.00 N

ATOM 2069 CA ALA 215 46.350 37.450 65.190 1.00 0.00 C

ATOM 2070 C ALA 215 45.410 37.470 63.970 1.00 0.00 C

ATOM 2071 O ALA 215 44.250 37.090 64.080 1.00 0.00 O

ATOM 2072 CB ALA 215 46.630 38.870 65.670 1.00 0.00 C

ATOM 2073 H ALA 215 48.450 37.320 65.020 1.00 0.00 H

ATOM 2074 N SER 216 45.990 37.730 62.790 1.00 0.00 N

ATOM 2075 CA SER 216 45.250 37.760 61.510 1.00 0.00 C

ATOM 2076 C SER 216 44.720 36.400 61.020 1.00 0.00 C

ATOM 2077 O SER 216 43.690 36.360 60.340 1.00 0.00 O

ATOM 2078 CB SER 216 46.020 38.430 60.370 1.00 0.00 C

ATOM 2079 OG SER 216 46.290 39.830 60.570 1.00 0.00 O

ATOM 2080 H SER 216 46.980 37.820 62.710 1.00 0.00 H

ATOM 2081 HG SER 216 47.220 39.890 60.970 1.00 0.00 H

ATOM 2082 N HIS 217 45.420 35.310 61.330 1.00 0.00 N

ATOM 2083 CA HIS 217 44.920 33.950 61.040 1.00 0.00 C

ATOM 2084 C HIS 217 43.640 33.670 61.850 1.00 0.00 C

ATOM 2085 O HIS 217 43.680 33.640 63.080 1.00 0.00 O

ATOM 2086 CB HIS 217 45.950 32.860 61.360 1.00 0.00 C

ATOM 2087 CG HIS 217 45.440 31.470 60.930 1.00 0.00 C

ATOM 2088 CD2 HIS 217 44.970 30.540 61.750 1.00 0.00 C

ATOM 2089 ND1 HIS 217 45.280 31.040 59.670 1.00 0.00 N

ATOM 2090 CE1 HIS 217 44.710 29.830 59.740 1.00 0.00 C

ATOM 2091 NE2 HIS 217 44.520 29.530 61.020 1.00 0.00 N

ATOM 2092 H HIS 217 46.300 35.340 61.810 1.00 0.00 H

ATOM 2093 HE2 HIS 217 44.050 28.720 61.380 1.00 0.00 H

ATOM 2094 N GLY 218 42.610 33.300 61.110 1.00 0.00 N

ATOM 2095 CA GLY 218 41.240 33.180 61.650 1.00 0.00 C

ATOM 2096 C GLY 218 40.270 34.220 61.080 1.00 0.00 C

ATOM 2097 O GLY 218 39.060 33.990 61.020 1.00 0.00 O

ATOM 2098 H GLY 218 42.760 32.890 60.210 1.00 0.00 H

ATOM 2099 N PHE 219 40.800 35.380 60.670 1.00 0.00 N

ATOM 2100 CA PHE 219 40.020 36.450 60.030 1.00 0.00 C

ATOM 2101 C PHE 219 39.580 36.220 58.580 1.00 0.00 C

ATOM 2102 O PHE 219 38.620 36.830 58.130 1.00 0.00 O

ATOM 2103 CB PHE 219 40.780 37.780 60.090 1.00 0.00 C

ATOM 2104 CG PHE 219 40.740 38.400 61.490 1.00 0.00 C

ATOM 2105 CD1 PHE 219 39.530 38.490 62.170 1.00 0.00 C

ATOM 2106 CD2 PHE 219 41.930 38.620 62.160 1.00 0.00 C

ATOM 2107 CE1 PHE 219 39.530 38.740 63.530 1.00 0.00 C

ATOM 2108 CE2 PHE 219 41.930 38.880 63.520 1.00 0.00 C

ATOM 2109 CZ PHE 219 40.730 38.910 64.210 1.00 0.00 C

ATOM 2110 H PHE 219 41.750 35.610 60.880 1.00 0.00 H

ATOM 2111 HD1 PHE 219 38.590 38.470 61.620 1.00 0.00 H

ATOM 2112 HD2 PHE 219 42.860 38.630 61.600 1.00 0.00 H

ATOM 2113 HE1 PHE 219 38.590 38.870 64.040 1.00 0.00 H

ATOM 2114 HE2 PHE 219 42.870 39.060 64.040 1.00 0.00 H

ATOM 2115 HZ PHE 219 40.730 39.020 65.300 1.00 0.00 H

ATOM 2116 N PHE 220 40.370 35.440 57.850 1.00 0.00 N

ATOM 2117 CA PHE 220 40.260 35.370 56.380 1.00 0.00 C

ATOM 2118 C PHE 220 40.690 34.030 55.760 1.00 0.00 C

ATOM 2119 O PHE 220 41.570 33.350 56.290 1.00 0.00 O

ATOM 2120 CB PHE 220 41.080 36.560 55.870 1.00 0.00 C

ATOM 2121 CG PHE 220 42.610 36.410 55.890 1.00 0.00 C

ATOM 2122 CD1 PHE 220 43.280 36.780 57.050 1.00 0.00 C

ATOM 2123 CD2 PHE 220 43.330 36.080 54.750 1.00 0.00 C

ATOM 2124 CE1 PHE 220 44.670 36.790 57.060 1.00 0.00 C

ATOM 2125 CE2 PHE 220 44.710 36.130 54.740 1.00 0.00 C

ATOM 2126 CZ PHE 220 45.380 36.480 55.910 1.00 0.00 C

ATOM 2127 H PHE 220 41.050 34.830 58.260 1.00 0.00 H

ATOM 2128 HD1 PHE 220 42.720 37.190 57.890 1.00 0.00 H

ATOM 2129 HD2 PHE 220 42.820 35.780 53.830 1.00 0.00 H

ATOM 2130 HE1 PHE 220 45.190 37.070 57.970 1.00 0.00 H

ATOM 2131 HE2 PHE 220 45.250 36.070 53.800 1.00 0.00 H

ATOM 2132 HZ PHE 220 46.470 36.510 55.920 1.00 0.00 H

ATOM 2133 N GLN 221 40.090 33.690 54.620 1.00 0.00 N

ATOM 2134 CA GLN 221 40.390 32.460 53.860 1.00 0.00 C

ATOM 2135 C GLN 221 41.640 32.560 52.960 1.00 0.00 C

ATOM 2136 O GLN 221 42.640 31.940 53.310 1.00 0.00 O

ATOM 2137 CB GLN 221 39.180 31.920 53.080 1.00 0.00 C

ATOM 2138 CG GLN 221 39.500 30.560 52.440 1.00 0.00 C

ATOM 2139 CD GLN 221 38.410 29.910 51.580 1.00 0.00 C

ATOM 2140 NE2 GLN 221 37.610 30.650 50.840 1.00 0.00 N

ATOM 2141 OE1 GLN 221 38.360 28.690 51.460 1.00 0.00 O

ATOM 2142 H GLN 221 39.230 34.130 54.350 1.00 0.00 H

ATOM 2143 1HE2 GLN 221 37.680 31.660 50.780 1.00 0.00 H

ATOM 2144 2HE2 GLN 221 36.890 30.170 50.350 1.00 0.00 H

ATOM 2145 N ARG 222 41.620 33.370 51.900 1.00 0.00 N

ATOM 2146 CA ARG 222 42.720 33.430 50.900 1.00 0.00 C

ATOM 2147 C ARG 222 43.300 34.840 50.690 1.00 0.00 C

ATOM 2148 O ARG 222 42.780 35.810 51.250 1.00 0.00 O

ATOM 2149 CB ARG 222 42.230 32.880 49.560 1.00 0.00 C

ATOM 2150 CG ARG 222 41.930 31.390 49.660 1.00 0.00 C

ATOM 2151 CD ARG 222 41.310 30.900 48.360 1.00 0.00 C

ATOM 2152 NE ARG 222 40.820 29.530 48.570 1.00 0.00 N

ATOM 2153 CZ ARG 222 39.580 29.090 48.350 1.00 0.00 C

ATOM 2154 NH1 ARG 222 38.590 29.890 47.920 1.00 0.00 N

ATOM 2155 NH2 ARG 222 39.310 27.800 48.520 1.00 0.00 N

ATOM 2156 H ARG 222 40.820 33.940 51.680 1.00 0.00 H

ATOM 2157 HE ARG 222 41.520 28.880 48.890 1.00 0.00 H

ATOM 2158 1HH1 ARG 222 38.760 30.860 47.750 1.00 0.00 H

ATOM 2159 2HH1 ARG 222 37.660 29.540 47.830 1.00 0.00 H

ATOM 2160 1HH2 ARG 222 40.010 27.210 48.940 1.00 0.00 H

ATOM 2161 2HH2 ARG 222 38.480 27.390 48.140 1.00 0.00 H

ATOM 2162 N ALA 223 44.410 34.940 49.970 1.00 0.00 N

ATOM 2163 CA ALA 223 45.040 36.250 49.720 1.00 0.00 C

ATOM 2164 C ALA 223 45.380 36.530 48.250 1.00 0.00 C

ATOM 2165 O ALA 223 45.640 35.610 47.470 1.00 0.00 O

ATOM 2166 CB ALA 223 46.290 36.370 50.600 1.00 0.00 C

ATOM 2167 H ALA 223 44.840 34.160 49.500 1.00 0.00 H

ATOM 2168 N VAL 224 45.190 37.790 47.890 1.00 0.00 N

ATOM 2169 CA VAL 224 45.600 38.370 46.590 1.00 0.00 C

ATOM 2170 C VAL 224 46.470 39.620 46.870 1.00 0.00 C

ATOM 2171 O VAL 224 46.080 40.510 47.620 1.00 0.00 O

ATOM 2172 CB VAL 224 44.360 38.690 45.730 1.00 0.00 C

ATOM 2173 CG1 VAL 224 44.680 39.460 44.450 1.00 0.00 C

ATOM 2174 CG2 VAL 224 43.630 37.410 45.320 1.00 0.00 C

ATOM 2175 H VAL 224 44.670 38.430 48.470 1.00 0.00 H

ATOM 2176 N MET 225 47.670 39.580 46.300 1.00 0.00 N

ATOM 2177 CA MET 225 48.640 40.690 46.390 1.00 0.00 C

ATOM 2178 C MET 225 49.040 41.200 45.000 1.00 0.00 C

ATOM 2179 O MET 225 49.460 40.440 44.120 1.00 0.00 O

ATOM 2180 CB MET 225 49.880 40.290 47.190 1.00 0.00 C

ATOM 2181 CG MET 225 49.600 40.140 48.690 1.00 0.00 C

ATOM 2182 SD MET 225 51.120 39.940 49.680 1.00 0.00 S

ATOM 2183 CE MET 225 51.310 38.170 49.630 1.00 0.00 C

ATOM 2184 H MET 225 47.940 38.810 45.720 1.00 0.00 H

ATOM 2185 N GLN 226 48.670 42.450 44.780 1.00 0.00 N

ATOM 2186 CA GLN 226 48.950 43.190 43.530 1.00 0.00 C

ATOM 2187 C GLN 226 50.030 44.230 43.810 1.00 0.00 C

ATOM 2188 O GLN 226 49.920 45.020 44.750 1.00 0.00 O

ATOM 2189 CB GLN 226 47.690 43.920 43.050 1.00 0.00 C

ATOM 2190 CG GLN 226 46.540 42.980 42.660 1.00 0.00 C

ATOM 2191 CD GLN 226 45.210 43.710 42.450 1.00 0.00 C

ATOM 2192 NE2 GLN 226 45.220 44.910 41.910 1.00 0.00 N

ATOM 2193 OE1 GLN 226 44.150 43.190 42.790 1.00 0.00 O

ATOM 2194 H GLN 226 48.160 42.980 45.460 1.00 0.00 H

ATOM 2195 1HE2 GLN 226 46.080 45.360 41.610 1.00 0.00 H

ATOM 2196 2HE2 GLN 226 44.360 45.380 41.770 1.00 0.00 H

ATOM 2197 N SER 227 51.190 44.010 43.200 1.00 0.00 N

ATOM 2198 CA SER 227 52.350 44.920 43.270 1.00 0.00 C

ATOM 2199 C SER 227 52.840 45.330 44.680 1.00 0.00 C

ATOM 2200 O SER 227 53.190 46.480 44.940 1.00 0.00 O

ATOM 2201 CB SER 227 52.080 46.150 42.400 1.00 0.00 C

ATOM 2202 OG SER 227 51.550 45.750 41.130 1.00 0.00 O

ATOM 2203 H SER 227 51.240 43.360 42.440 1.00 0.00 H

ATOM 2204 HG SER 227 51.590 46.590 40.550 1.00 0.00 H

ATOM 2205 N GLY 228 52.860 44.360 45.590 1.00 0.00 N

ATOM 2206 CA GLY 228 53.390 44.550 46.950 1.00 0.00 C

ATOM 2207 C GLY 228 53.410 43.260 47.790 1.00 0.00 C

ATOM 2208 O GLY 228 52.540 42.400 47.700 1.00 0.00 O

ATOM 2209 H GLY 228 52.430 43.470 45.400 1.00 0.00 H

ATOM 2210 N CYS 229 54.470 43.160 48.570 1.00 0.00 N

ATOM 2211 CA CYS 229 54.670 42.060 49.540 1.00 0.00 C

ATOM 2212 C CYS 229 55.480 42.570 50.750 1.00 0.00 C

ATOM 2213 O CYS 229 56.340 43.430 50.570 1.00 0.00 O

ATOM 2214 CB CYS 229 55.390 40.890 48.840 1.00 0.00 C

ATOM 2215 SG CYS 229 57.110 41.230 48.340 1.00 0.00 S

ATOM 2216 H CYS 229 55.260 43.760 48.430 1.00 0.00 H

ATOM 2217 HG CYS 229 57.310 40.070 47.730 1.00 0.00 H

ATOM 2218 N PRO 230 55.310 41.980 51.950 1.00 0.00 N

ATOM 2219 CA PRO 230 55.980 42.470 53.180 1.00 0.00 C

ATOM 2220 C PRO 230 57.510 42.560 53.110 1.00 0.00 C

ATOM 2221 O PRO 230 58.100 43.390 53.790 1.00 0.00 O

ATOM 2222 CB PRO 230 55.500 41.520 54.280 1.00 0.00 C

ATOM 2223 CG PRO 230 55.200 40.220 53.540 1.00 0.00 C

ATOM 2224 CD PRO 230 54.610 40.710 52.220 1.00 0.00 C

ATOM 2225 N ASN 231 58.130 41.710 52.290 1.00 0.00 N

ATOM 2226 CA ASN 231 59.600 41.710 52.100 1.00 0.00 C

ATOM 2227 C ASN 231 60.160 42.740 51.100 1.00 0.00 C

ATOM 2228 O ASN 231 61.360 42.770 50.850 1.00 0.00 O

ATOM 2229 CB ASN 231 60.080 40.310 51.740 1.00 0.00 C

ATOM 2230 CG ASN 231 59.590 39.740 50.410 1.00 0.00 C

ATOM 2231 ND2 ASN 231 60.440 39.660 49.410 1.00 0.00 N

ATOM 2232 OD1 ASN 231 58.440 39.370 50.280 1.00 0.00 O

ATOM 2233 H ASN 231 57.640 41.020 51.760 1.00 0.00 H

ATOM 2234 1HD2 ASN 231 61.380 40.010 49.460 1.00 0.00 H

ATOM 2235 2HD2 ASN 231 60.060 39.380 48.530 1.00 0.00 H

ATOM 2236 N ALA 232 59.270 43.510 50.480 1.00 0.00 N

ATOM 2237 CA ALA 232 59.680 44.670 49.660 1.00 0.00 C

ATOM 2238 C ALA 232 60.480 45.630 50.560 1.00 0.00 C

ATOM 2239 O ALA 232 60.020 45.920 51.660 1.00 0.00 O

ATOM 2240 CB ALA 232 58.430 45.360 49.120 1.00 0.00 C

ATOM 2241 H ALA 232 58.280 43.420 50.600 1.00 0.00 H

ATOM 2242 N PRO 233 61.650 46.090 50.110 1.00 0.00 N

ATOM 2243 CA PRO 233 62.570 46.880 50.970 1.00 0.00 C

ATOM 2244 C PRO 233 61.920 48.090 51.650 1.00 0.00 C

ATOM 2245 O PRO 233 62.120 48.320 52.840 1.00 0.00 O

ATOM 2246 CB PRO 233 63.730 47.260 50.050 1.00 0.00 C

ATOM 2247 CG PRO 233 63.150 47.170 48.640 1.00 0.00 C

ATOM 2248 CD PRO 233 62.130 46.040 48.720 1.00 0.00 C

ATOM 2249 N TRP 234 60.990 48.710 50.930 1.00 0.00 N

ATOM 2250 CA TRP 234 60.150 49.820 51.420 1.00 0.00 C

ATOM 2251 C TRP 234 59.100 49.450 52.490 1.00 0.00 C

ATOM 2252 O TRP 234 58.780 50.280 53.340 1.00 0.00 O

ATOM 2253 CB TRP 234 59.490 50.470 50.200 1.00 0.00 C

ATOM 2254 CG TRP 234 58.660 49.530 49.320 1.00 0.00 C

ATOM 2255 CD1 TRP 234 59.040 49.060 48.130 1.00 0.00 C

ATOM 2256 CD2 TRP 234 57.340 49.120 49.500 1.00 0.00 C

ATOM 2257 CE2 TRP 234 57.000 48.400 48.370 1.00 0.00 C

ATOM 2258 CE3 TRP 234 56.430 49.290 50.520 1.00 0.00 C

ATOM 2259 NE1 TRP 234 58.040 48.390 47.560 1.00 0.00 N

ATOM 2260 CZ2 TRP 234 55.750 47.790 48.280 1.00 0.00 C

ATOM 2261 CZ3 TRP 234 55.180 48.690 50.440 1.00 0.00 C

ATOM 2262 CH2 TRP 234 54.850 47.930 49.320 1.00 0.00 C

ATOM 2263 H TRP 234 60.810 48.470 49.980 1.00 0.00 H

ATOM 2264 HD1 TRP 234 60.000 49.270 47.650 1.00 0.00 H

ATOM 2265 HE1 TRP 234 58.080 47.940 46.670 1.00 0.00 H

ATOM 2266 HE3 TRP 234 56.650 49.930 51.370 1.00 0.00 H

ATOM 2267 HZ2 TRP 234 55.480 47.220 47.400 1.00 0.00 H

ATOM 2268 HZ3 TRP 234 54.440 48.850 51.220 1.00 0.00 H

ATOM 2269 HH2 TRP 234 53.890 47.410 49.280 1.00 0.00 H

ATOM 2270 N ALA 235 58.650 48.190 52.510 1.00 0.00 N

ATOM 2271 CA ALA 235 57.490 47.730 53.300 1.00 0.00 C

ATOM 2272 C ALA 235 57.640 47.750 54.830 1.00 0.00 C

ATOM 2273 O ALA 235 56.680 48.060 55.520 1.00 0.00 O

ATOM 2274 CB ALA 235 57.050 46.350 52.830 1.00 0.00 C

ATOM 2275 H ALA 235 59.150 47.460 52.040 1.00 0.00 H

ATOM 2276 N THR 236 58.840 47.490 55.340 1.00 0.00 N

ATOM 2277 CA THR 236 59.110 47.500 56.790 1.00 0.00 C

ATOM 2278 C THR 236 60.200 48.510 57.180 1.00 0.00 C

ATOM 2279 O THR 236 61.140 48.760 56.420 1.00 0.00 O

ATOM 2280 CB THR 236 59.540 46.130 57.330 1.00 0.00 C

ATOM 2281 CG2 THR 236 58.370 45.150 57.440 1.00 0.00 C

ATOM 2282 OG1 THR 236 60.600 45.590 56.540 1.00 0.00 O

ATOM 2283 H THR 236 59.640 47.280 54.770 1.00 0.00 H

ATOM 2284 HG1 THR 236 61.040 44.830 57.050 1.00 0.00 H

ATOM 2285 N VAL 237 60.030 49.090 58.360 1.00 0.00 N

ATOM 2286 CA VAL 237 61.070 49.960 58.960 1.00 0.00 C

ATOM 2287 C VAL 237 61.670 49.290 60.200 1.00 0.00 C

ATOM 2288 O VAL 237 60.970 48.670 61.000 1.00 0.00 O

ATOM 2289 CB VAL 237 60.590 51.400 59.250 1.00 0.00 C

ATOM 2290 CG1 VAL 237 60.190 52.120 57.960 1.00 0.00 C

ATOM 2291 CG2 VAL 237 59.440 51.490 60.250 1.00 0.00 C

ATOM 2292 H VAL 237 59.220 48.920 58.930 1.00 0.00 H

ATOM 2293 N ASN 238 62.990 49.340 60.260 1.00 0.00 N

ATOM 2294 CA ASN 238 63.780 48.760 61.370 1.00 0.00 C

ATOM 2295 C ASN 238 63.530 49.470 62.710 1.00 0.00 C

ATOM 2296 O ASN 238 63.110 50.620 62.730 1.00 0.00 O

ATOM 2297 CB ASN 238 65.270 48.800 61.030 1.00 0.00 C

ATOM 2298 CG ASN 238 65.550 47.950 59.790 1.00 0.00 C

ATOM 2299 ND2 ASN 238 66.230 48.540 58.840 1.00 0.00 N

ATOM 2300 OD1 ASN 238 65.080 46.830 59.640 1.00 0.00 O

ATOM 2301 H ASN 238 63.530 49.760 59.520 1.00 0.00 H

ATOM 2302 1HD2 ASN 238 66.570 49.470 58.940 1.00 0.00 H

ATOM 2303 2HD2 ASN 238 66.390 47.990 58.000 1.00 0.00 H

ATOM 2304 N GLN 239 63.840 48.780 63.810 1.00 0.00 N

ATOM 2305 CA GLN 239 63.740 49.400 65.150 1.00 0.00 C

ATOM 2306 C GLN 239 64.600 50.660 65.310 1.00 0.00 C

ATOM 2307 O GLN 239 64.110 51.680 65.780 1.00 0.00 O

ATOM 2308 CB GLN 239 63.990 48.420 66.320 1.00 0.00 C

ATOM 2309 CG GLN 239 65.410 47.880 66.470 1.00 0.00 C

ATOM 2310 CD GLN 239 65.870 47.090 65.250 1.00 0.00 C

ATOM 2311 NE2 GLN 239 65.240 45.960 65.000 1.00 0.00 N

ATOM 2312 OE1 GLN 239 66.710 47.530 64.490 1.00 0.00 O

ATOM 2313 H GLN 239 64.160 47.830 63.770 1.00 0.00 H

ATOM 2314 1HE2 GLN 239 64.540 45.620 65.630 1.00 0.00 H

ATOM 2315 2HE2 GLN 239 65.490 45.430 64.190 1.00 0.00 H

ATOM 2316 N LYS 240 65.790 50.610 64.710 1.00 0.00 N

ATOM 2317 CA LYS 240 66.720 51.760 64.660 1.00 0.00 C

ATOM 2318 C LYS 240 66.090 52.940 63.900 1.00 0.00 C

ATOM 2319 O LYS 240 65.790 53.960 64.530 1.00 0.00 O

ATOM 2320 CB LYS 240 68.030 51.280 64.040 1.00 0.00 C

ATOM 2321 CG LYS 240 69.140 52.330 64.130 1.00 0.00 C

ATOM 2322 CD LYS 240 70.520 51.710 64.350 1.00 0.00 C

ATOM 2323 CE LYS 240 70.990 50.830 63.190 1.00 0.00 C

ATOM 2324 NZ LYS 240 72.230 50.120 63.530 1.00 0.00 N1+

ATOM 2325 H LYS 240 66.160 49.760 64.340 1.00 0.00 H

ATOM 2326 HZ1 LYS 240 72.360 49.360 62.910 1.00 0.00 H

ATOM 2327 HZ2 LYS 240 73.020 50.730 63.450 1.00 0.00 H

ATOM 2328 HZ3 LYS 240 72.180 49.770 64.470 1.00 0.00 H

ATOM 2329 N ASP 241 65.660 52.670 62.670 1.00 0.00 N

ATOM 2330 CA ASP 241 64.910 53.630 61.830 1.00 0.00 C

ATOM 2331 C ASP 241 63.720 54.220 62.590 1.00 0.00 C

ATOM 2332 O ASP 241 63.730 55.410 62.890 1.00 0.00 O

ATOM 2333 CB ASP 241 64.390 52.970 60.540 1.00 0.00 C

ATOM 2334 CG ASP 241 65.450 52.570 59.500 1.00 0.00 C

ATOM 2335 OD1 ASP 241 66.650 52.660 59.790 1.00 0.00 O

ATOM 2336 OD2 ASP 241 65.040 52.050 58.450 1.00 0.00 O

ATOM 2337 H ASP 241 65.990 51.880 62.160 1.00 0.00 H

ATOM 2338 N LEU 242 62.840 53.360 63.100 1.00 0.00 N

ATOM 2339 CA LEU 242 61.620 53.800 63.810 1.00 0.00 C

ATOM 2340 C LEU 242 61.900 54.730 64.990 1.00 0.00 C

ATOM 2341 O LEU 242 61.260 55.780 65.100 1.00 0.00 O

ATOM 2342 CB LEU 242 60.700 52.610 64.180 1.00 0.00 C

ATOM 2343 CG LEU 242 61.110 51.740 65.380 1.00 0.00 C

ATOM 2344 CD1 LEU 242 60.710 52.360 66.710 1.00 0.00 C

ATOM 2345 CD2 LEU 242 60.490 50.350 65.280 1.00 0.00 C

ATOM 2346 H LEU 242 62.970 52.360 63.020 1.00 0.00 H

ATOM 2347 N TRP 243 62.930 54.400 65.770 1.00 0.00 N

ATOM 2348 CA TRP 243 63.280 55.160 66.990 1.00 0.00 C

ATOM 2349 C TRP 243 63.880 56.530 66.660 1.00 0.00 C

ATOM 2350 O TRP 243 63.360 57.550 67.110 1.00 0.00 O

ATOM 2351 CB TRP 243 64.210 54.320 67.870 1.00 0.00 C

ATOM 2352 CG TRP 243 64.510 54.960 69.230 1.00 0.00 C

ATOM 2353 CD1 TRP 243 63.870 55.980 69.810 1.00 0.00 C

ATOM 2354 CD2 TRP 243 65.570 54.610 70.050 1.00 0.00 C

ATOM 2355 CE2 TRP 243 65.510 55.460 71.140 1.00 0.00 C

ATOM 2356 CE3 TRP 243 66.550 53.640 69.950 1.00 0.00 C

ATOM 2357 NE1 TRP 243 64.490 56.290 70.950 1.00 0.00 N

ATOM 2358 CZ2 TRP 243 66.430 55.320 72.170 1.00 0.00 C

ATOM 2359 CZ3 TRP 243 67.470 53.490 70.980 1.00 0.00 C

ATOM 2360 CH2 TRP 243 67.400 54.330 72.080 1.00 0.00 C

ATOM 2361 H TRP 243 63.510 53.610 65.580 1.00 0.00 H

ATOM 2362 HD1 TRP 243 63.040 56.540 69.380 1.00 0.00 H

ATOM 2363 HE1 TRP 243 64.340 57.140 71.460 1.00 0.00 H

ATOM 2364 HE3 TRP 243 66.690 53.090 69.020 1.00 0.00 H

ATOM 2365 HZ2 TRP 243 66.470 56.060 72.970 1.00 0.00 H

ATOM 2366 HZ3 TRP 243 68.200 52.690 70.940 1.00 0.00 H

ATOM 2367 HH2 TRP 243 68.160 54.250 72.850 1.00 0.00 H

ATOM 2368 N ASP 244 64.840 56.530 65.730 1.00 0.00 N

ATOM 2369 CA ASP 244 65.400 57.770 65.170 1.00 0.00 C

ATOM 2370 C ASP 244 64.270 58.660 64.600 1.00 0.00 C

ATOM 2371 O ASP 244 64.050 59.760 65.110 1.00 0.00 O

ATOM 2372 CB ASP 244 66.450 57.390 64.110 1.00 0.00 C

ATOM 2373 CG ASP 244 67.780 56.860 64.670 1.00 0.00 C

ATOM 2374 OD1 ASP 244 67.880 56.550 65.880 1.00 0.00 O

ATOM 2375 OD2 ASP 244 68.770 56.880 63.920 1.00 0.00 O

ATOM 2376 H ASP 244 65.310 55.700 65.440 1.00 0.00 H

ATOM 2377 N ARG 245 63.350 58.000 63.900 1.00 0.00 N

ATOM 2378 CA ARG 245 62.150 58.600 63.280 1.00 0.00 C

ATOM 2379 C ARG 245 61.150 59.250 64.260 1.00 0.00 C

ATOM 2380 O ARG 245 60.800 60.420 64.090 1.00 0.00 O

ATOM 2381 CB ARG 245 61.490 57.590 62.330 1.00 0.00 C

ATOM 2382 CG ARG 245 62.410 57.360 61.130 1.00 0.00 C

ATOM 2383 CD ARG 245 61.860 56.400 60.080 1.00 0.00 C

ATOM 2384 NE ARG 245 62.900 56.100 59.080 1.00 0.00 N

ATOM 2385 CZ ARG 245 62.700 55.410 57.930 1.00 0.00 C

ATOM 2386 NH1 ARG 245 61.480 55.020 57.560 1.00 0.00 N

ATOM 2387 NH2 ARG 245 63.720 55.080 57.140 1.00 0.00 N

ATOM 2388 H ARG 245 63.520 57.040 63.650 1.00 0.00 H

ATOM 2389 HE ARG 245 63.800 56.480 59.280 1.00 0.00 H

ATOM 2390 1HH1 ARG 245 60.680 55.260 58.110 1.00 0.00 H

ATOM 2391 2HH1 ARG 245 61.360 54.480 56.720 1.00 0.00 H

ATOM 2392 1HH2 ARG 245 64.670 55.320 57.380 1.00 0.00 H

ATOM 2393 2HH2 ARG 245 63.550 54.570 56.300 1.00 0.00 H

ATOM 2394 N SER 246 60.780 58.530 65.310 1.00 0.00 N

ATOM 2395 CA SER 246 59.910 59.080 66.390 1.00 0.00 C

ATOM 2396 C SER 246 60.570 60.240 67.140 1.00 0.00 C

ATOM 2397 O SER 246 59.970 61.310 67.300 1.00 0.00 O

ATOM 2398 CB SER 246 59.440 57.990 67.370 1.00 0.00 C

ATOM 2399 OG SER 246 60.520 57.370 68.070 1.00 0.00 O

ATOM 2400 H SER 246 61.080 57.580 65.440 1.00 0.00 H

ATOM 2401 HG SER 246 60.180 56.490 68.420 1.00 0.00 H

ATOM 2402 N MET 247 61.870 60.090 67.390 1.00 0.00 N

ATOM 2403 CA MET 247 62.720 61.130 68.000 1.00 0.00 C

ATOM 2404 C MET 247 62.940 62.390 67.140 1.00 0.00 C

ATOM 2405 O MET 247 63.090 63.480 67.690 1.00 0.00 O

ATOM 2406 CB MET 247 64.060 60.560 68.450 1.00 0.00 C

ATOM 2407 CG MET 247 63.930 59.690 69.710 1.00 0.00 C

ATOM 2408 SD MET 247 63.310 60.610 71.170 1.00 0.00 S

ATOM 2409 CE MET 247 63.530 59.340 72.410 1.00 0.00 C

ATOM 2410 H MET 247 62.320 59.200 67.240 1.00 0.00 H

ATOM 2411 N MET 248 63.050 62.220 65.820 1.00 0.00 N

ATOM 2412 CA MET 248 63.110 63.360 64.890 1.00 0.00 C

ATOM 2413 C MET 248 61.770 64.130 64.850 1.00 0.00 C

ATOM 2414 O MET 248 61.780 65.330 65.120 1.00 0.00 O

ATOM 2415 CB MET 248 63.560 62.920 63.490 1.00 0.00 C

ATOM 2416 CG MET 248 63.750 64.090 62.520 1.00 0.00 C

ATOM 2417 SD MET 248 64.930 65.380 63.060 1.00 0.00 S

ATOM 2418 CE MET 248 64.570 66.640 61.870 1.00 0.00 C

ATOM 2419 H MET 248 63.260 61.320 65.420 1.00 0.00 H

ATOM 2420 N LEU 249 60.660 63.400 64.790 1.00 0.00 N

ATOM 2421 CA LEU 249 59.300 64.000 64.850 1.00 0.00 C

ATOM 2422 C LEU 249 59.100 64.820 66.140 1.00 0.00 C

ATOM 2423 O LEU 249 58.730 65.990 66.090 1.00 0.00 O

ATOM 2424 CB LEU 249 58.230 62.890 64.750 1.00 0.00 C

ATOM 2425 CG LEU 249 56.810 63.440 64.610 1.00 0.00 C

ATOM 2426 CD1 LEU 249 56.570 64.080 63.230 1.00 0.00 C

ATOM 2427 CD2 LEU 249 55.790 62.330 64.860 1.00 0.00 C

ATOM 2428 H LEU 249 60.690 62.420 64.590 1.00 0.00 H

ATOM 2429 N SER 250 59.560 64.230 67.230 1.00 0.00 N

ATOM 2430 CA SER 250 59.720 64.840 68.570 1.00 0.00 C

ATOM 2431 C SER 250 60.400 66.210 68.520 1.00 0.00 C

ATOM 2432 O SER 250 59.780 67.240 68.780 1.00 0.00 O

ATOM 2433 CB SER 250 60.560 63.820 69.360 1.00 0.00 C

ATOM 2434 OG SER 250 61.430 64.370 70.350 1.00 0.00 O

ATOM 2435 H SER 250 59.810 63.250 67.190 1.00 0.00 H

ATOM 2436 HG SER 250 61.970 63.620 70.740 1.00 0.00 H

ATOM 2437 N LYS 251 61.620 66.190 67.990 1.00 0.00 N

ATOM 2438 CA LYS 251 62.510 67.360 67.880 1.00 0.00 C

ATOM 2439 C LYS 251 61.910 68.460 66.980 1.00 0.00 C

ATOM 2440 O LYS 251 61.640 69.560 67.460 1.00 0.00 O

ATOM 2441 CB LYS 251 63.850 66.820 67.370 1.00 0.00 C

ATOM 2442 CG LYS 251 64.970 67.840 67.490 1.00 0.00 C

ATOM 2443 CD LYS 251 65.220 68.240 68.950 1.00 0.00 C

ATOM 2444 CE LYS 251 65.890 69.600 69.020 1.00 0.00 C

ATOM 2445 NZ LYS 251 67.230 69.590 68.410 1.00 0.00 N1+

ATOM 2446 H LYS 251 62.010 65.330 67.650 1.00 0.00 H

ATOM 2447 HZ1 LYS 251 67.650 70.490 68.530 1.00 0.00 H

ATOM 2448 HZ2 LYS 251 67.800 68.920 68.900 1.00 0.00 H

ATOM 2449 HZ3 LYS 251 67.160 69.360 67.440 1.00 0.00 H

ATOM 2450 N ALA 252 61.440 68.010 65.820 1.00 0.00 N

ATOM 2451 CA ALA 252 60.780 68.850 64.800 1.00 0.00 C

ATOM 2452 C ALA 252 59.510 69.550 65.320 1.00 0.00 C

ATOM 2453 O ALA 252 59.300 70.740 65.080 1.00 0.00 O

ATOM 2454 CB ALA 252 60.510 67.900 63.630 1.00 0.00 C

ATOM 2455 H ALA 252 61.560 67.050 65.550 1.00 0.00 H

ATOM 2456 N LEU 253 58.710 68.820 66.100 1.00 0.00 N

ATOM 2457 CA LEU 253 57.510 69.360 66.770 1.00 0.00 C

ATOM 2458 C LEU 253 57.770 70.190 68.050 1.00 0.00 C

ATOM 2459 O LEU 253 56.890 70.910 68.510 1.00 0.00 O

ATOM 2460 CB LEU 253 56.510 68.230 67.090 1.00 0.00 C

ATOM 2461 CG LEU 253 55.920 67.520 65.860 1.00 0.00 C

ATOM 2462 CD1 LEU 253 54.900 66.480 66.310 1.00 0.00 C

ATOM 2463 CD2 LEU 253 55.180 68.460 64.920 1.00 0.00 C

ATOM 2464 H LEU 253 58.850 67.830 66.210 1.00 0.00 H

ATOM 2465 N GLY 254 59.010 70.140 68.540 1.00 0.00 N

ATOM 2466 CA GLY 254 59.400 70.770 69.820 1.00 0.00 C

ATOM 2467 C GLY 254 58.840 70.030 71.060 1.00 0.00 C

ATOM 2468 O GLY 254 58.620 70.650 72.100 1.00 0.00 O

ATOM 2469 H GLY 254 59.760 69.710 68.050 1.00 0.00 H

ATOM 2470 N CYS 255 58.770 68.710 70.910 1.00 0.00 N

ATOM 2471 CA CYS 255 58.400 67.750 71.960 1.00 0.00 C

ATOM 2472 C CYS 255 59.510 66.810 72.510 1.00 0.00 C

ATOM 2473 O CYS 255 59.150 65.800 73.120 1.00 0.00 O

ATOM 2474 CB CYS 255 57.260 66.900 71.370 1.00 0.00 C

ATOM 2475 SG CYS 255 55.650 67.740 71.220 1.00 0.00 S

ATOM 2476 H CYS 255 59.010 68.280 70.040 1.00 0.00 H

ATOM 2477 N PRO 256 60.830 67.100 72.410 1.00 0.00 N

ATOM 2478 CA PRO 256 61.870 66.170 72.910 1.00 0.00 C

ATOM 2479 C PRO 256 61.840 66.110 74.440 1.00 0.00 C

ATOM 2480 O PRO 256 61.910 67.140 75.120 1.00 0.00 O

ATOM 2481 CB PRO 256 63.190 66.730 72.370 1.00 0.00 C

ATOM 2482 CG PRO 256 62.960 68.230 72.310 1.00 0.00 C

ATOM 2483 CD PRO 256 61.480 68.330 71.920 1.00 0.00 C

ATOM 2484 N MET 257 61.700 64.890 74.950 1.00 0.00 N

ATOM 2485 CA MET 257 61.490 64.640 76.390 1.00 0.00 C

ATOM 2486 C MET 257 62.250 63.410 76.890 1.00 0.00 C

ATOM 2487 O MET 257 62.260 62.370 76.220 1.00 0.00 O

ATOM 2488 CB MET 257 60.000 64.480 76.710 1.00 0.00 C

ATOM 2489 CG MET 257 59.190 65.760 76.500 1.00 0.00 C

ATOM 2490 SD MET 257 59.750 67.160 77.530 1.00 0.00 S

ATOM 2491 CE MET 257 59.130 68.520 76.570 1.00 0.00 C

ATOM 2492 H MET 257 61.880 64.080 74.410 1.00 0.00 H

ATOM 2493 N SER 258 62.700 63.500 78.150 1.00 0.00 N

ATOM 2494 CA SER 258 63.410 62.400 78.850 1.00 0.00 C

ATOM 2495 C SER 258 62.570 61.120 78.940 1.00 0.00 C

ATOM 2496 O SER 258 63.070 60.000 78.950 1.00 0.00 O

ATOM 2497 CB SER 258 63.840 62.780 80.280 1.00 0.00 C

ATOM 2498 OG SER 258 62.760 62.920 81.210 1.00 0.00 O

ATOM 2499 H SER 258 62.690 64.380 78.620 1.00 0.00 H

ATOM 2500 HG SER 258 62.900 63.800 81.700 1.00 0.00 H

ATOM 2501 N ASN 259 61.270 61.330 79.080 1.00 0.00 N

ATOM 2502 CA ASN 259 60.300 60.250 79.150 1.00 0.00 C

ATOM 2503 C ASN 259 59.600 60.100 77.790 1.00 0.00 C

ATOM 2504 O ASN 259 58.960 61.020 77.280 1.00 0.00 O

ATOM 2505 CB ASN 259 59.330 60.600 80.280 1.00 0.00 C

ATOM 2506 CG ASN 259 58.360 59.470 80.630 1.00 0.00 C

ATOM 2507 ND2 ASN 259 58.140 59.300 81.900 1.00 0.00 N

ATOM 2508 OD1 ASN 259 57.810 58.760 79.800 1.00 0.00 O

ATOM 2509 H ASN 259 60.920 62.260 79.220 1.00 0.00 H

ATOM 2510 1HD2 ASN 259 58.620 59.870 82.570 1.00 0.00 H

ATOM 2511 2HD2 ASN 259 57.420 58.700 82.230 1.00 0.00 H

ATOM 2512 N ARG 260 59.680 58.870 77.310 1.00 0.00 N

ATOM 2513 CA ARG 260 59.050 58.430 76.040 1.00 0.00 C

ATOM 2514 C ARG 260 57.520 58.640 76.010 1.00 0.00 C

ATOM 2515 O ARG 260 56.960 59.070 75.010 1.00 0.00 O

ATOM 2516 CB ARG 260 59.420 56.960 75.830 1.00 0.00 C

ATOM 2517 CG ARG 260 60.920 56.730 75.560 1.00 0.00 C

ATOM 2518 CD ARG 260 61.220 55.250 75.810 1.00 0.00 C

ATOM 2519 NE ARG 260 62.540 54.780 75.370 1.00 0.00 N

ATOM 2520 CZ ARG 260 62.950 53.500 75.420 1.00 0.00 C

ATOM 2521 NH1 ARG 260 62.170 52.540 75.940 1.00 0.00 N

ATOM 2522 NH2 ARG 260 64.110 53.140 74.860 1.00 0.00 N

ATOM 2523 H ARG 260 60.270 58.190 77.740 1.00 0.00 H

ATOM 2524 HE ARG 260 63.220 55.510 75.170 1.00 0.00 H

ATOM 2525 1HH1 ARG 260 61.240 52.740 76.250 1.00 0.00 H

ATOM 2526 2HH1 ARG 260 62.560 51.640 76.170 1.00 0.00 H

ATOM 2527 1HH2 ARG 260 64.690 53.820 74.420 1.00 0.00 H

ATOM 2528 2HH2 ARG 260 64.400 52.190 74.900 1.00 0.00 H

ATOM 2529 N GLY 261 56.900 58.470 77.180 1.00 0.00 N

ATOM 2530 CA GLY 261 55.490 58.790 77.440 1.00 0.00 C

ATOM 2531 C GLY 261 55.210 60.300 77.400 1.00 0.00 C

ATOM 2532 O GLY 261 54.290 60.760 76.730 1.00 0.00 O

ATOM 2533 H GLY 261 57.420 58.200 78.000 1.00 0.00 H

ATOM 2534 N LEU 262 56.100 61.100 77.980 1.00 0.00 N

ATOM 2535 CA LEU 262 56.010 62.570 77.880 1.00 0.00 C

ATOM 2536 C LEU 262 56.160 63.110 76.450 1.00 0.00 C

ATOM 2537 O LEU 262 55.730 64.220 76.150 1.00 0.00 O

ATOM 2538 CB LEU 262 56.990 63.290 78.820 1.00 0.00 C

ATOM 2539 CG LEU 262 56.480 63.480 80.260 1.00 0.00 C

ATOM 2540 CD1 LEU 262 55.200 64.310 80.330 1.00 0.00 C

ATOM 2541 CD2 LEU 262 56.280 62.150 80.970 1.00 0.00 C

ATOM 2542 H LEU 262 56.860 60.740 78.520 1.00 0.00 H

ATOM 2543 N LEU 263 56.880 62.360 75.620 1.00 0.00 N

ATOM 2544 CA LEU 263 56.880 62.600 74.170 1.00 0.00 C

ATOM 2545 C LEU 263 55.490 62.360 73.550 1.00 0.00 C

ATOM 2546 O LEU 263 54.940 63.280 72.930 1.00 0.00 O

ATOM 2547 CB LEU 263 57.980 61.770 73.500 1.00 0.00 C

ATOM 2548 CG LEU 263 58.020 61.840 71.970 1.00 0.00 C

ATOM 2549 CD1 LEU 263 57.980 63.290 71.470 1.00 0.00 C

ATOM 2550 CD2 LEU 263 59.280 61.160 71.450 1.00 0.00 C

ATOM 2551 H LEU 263 57.560 61.700 75.950 1.00 0.00 H

ATOM 2552 N GLU 264 54.880 61.210 73.840 1.00 0.00 N

ATOM 2553 CA GLU 264 53.530 60.880 73.330 1.00 0.00 C

ATOM 2554 C GLU 264 52.450 61.900 73.750 1.00 0.00 C

ATOM 2555 O GLU 264 51.820 62.490 72.880 1.00 0.00 O

ATOM 2556 CB GLU 264 53.140 59.430 73.660 1.00 0.00 C

ATOM 2557 CG GLU 264 52.660 59.060 75.070 1.00 0.00 C

ATOM 2558 CD GLU 264 51.200 59.400 75.410 1.00 0.00 C

ATOM 2559 OE1 GLU 264 50.390 59.480 74.470 1.00 0.00 O

ATOM 2560 OE2 GLU 264 50.870 59.520 76.620 1.00 0.00 O

ATOM 2561 H GLU 264 55.320 60.500 74.380 1.00 0.00 H

ATOM 2562 N THR 265 52.530 62.330 75.010 1.00 0.00 N

ATOM 2563 CA THR 265 51.620 63.350 75.580 1.00 0.00 C

ATOM 2564 C THR 265 51.690 64.680 74.800 1.00 0.00 C

ATOM 2565 O THR 265 50.670 65.180 74.330 1.00 0.00 O

ATOM 2566 CB THR 265 51.840 63.640 77.080 1.00 0.00 C

ATOM 2567 CG2 THR 265 51.650 62.390 77.940 1.00 0.00 C

ATOM 2568 OG1 THR 265 53.130 64.210 77.330 1.00 0.00 O

ATOM 2569 H THR 265 53.100 61.850 75.680 1.00 0.00 H

ATOM 2570 HG1 THR 265 53.060 64.910 78.060 1.00 0.00 H

ATOM 2571 N CYS 266 52.920 65.120 74.550 1.00 0.00 N

ATOM 2572 CA CYS 266 53.230 66.330 73.760 1.00 0.00 C

ATOM 2573 C CYS 266 52.820 66.200 72.280 1.00 0.00 C

ATOM 2574 O CYS 266 52.180 67.100 71.730 1.00 0.00 O

ATOM 2575 CB CYS 266 54.720 66.640 73.910 1.00 0.00 C

ATOM 2576 SG CYS 266 55.260 68.220 73.170 1.00 0.00 S

ATOM 2577 H CYS 266 53.710 64.690 74.990 1.00 0.00 H

ATOM 2578 N LEU 267 53.090 65.040 71.680 1.00 0.00 N

ATOM 2579 CA LEU 267 52.660 64.730 70.300 1.00 0.00 C

ATOM 2580 C LEU 267 51.130 64.720 70.150 1.00 0.00 C

ATOM 2581 O LEU 267 50.610 65.290 69.190 1.00 0.00 O

ATOM 2582 CB LEU 267 53.200 63.380 69.810 1.00 0.00 C

ATOM 2583 CG LEU 267 54.730 63.250 69.820 1.00 0.00 C

ATOM 2584 CD1 LEU 267 55.110 61.850 69.340 1.00 0.00 C

ATOM 2585 CD2 LEU 267 55.420 64.290 68.940 1.00 0.00 C

ATOM 2586 H LEU 267 53.660 64.340 72.130 1.00 0.00 H

ATOM 2587 N GLN 268 50.440 64.160 71.130 1.00 0.00 N

ATOM 2588 CA GLN 268 48.960 64.160 71.190 1.00 0.00 C

ATOM 2589 C GLN 268 48.340 65.570 71.230 1.00 0.00 C

ATOM 2590 O GLN 268 47.370 65.790 70.520 1.00 0.00 O

ATOM 2591 CB GLN 268 48.440 63.360 72.390 1.00 0.00 C

ATOM 2592 CG GLN 268 48.560 61.850 72.210 1.00 0.00 C

ATOM 2593 CD GLN 268 47.920 61.090 73.380 1.00 0.00 C

ATOM 2594 NE2 GLN 268 48.210 61.520 74.590 1.00 0.00 N

ATOM 2595 OE1 GLN 268 47.050 60.240 73.220 1.00 0.00 O

ATOM 2596 H GLN 268 50.890 63.620 71.850 1.00 0.00 H

ATOM 2597 1HE2 GLN 268 48.780 62.320 74.720 1.00 0.00 H

ATOM 2598 2HE2 GLN 268 48.210 60.820 75.320 1.00 0.00 H

ATOM 2599 N GLN 269 49.030 66.500 71.880 1.00 0.00 N

ATOM 2600 CA GLN 269 48.660 67.940 71.930 1.00 0.00 C

ATOM 2601 C GLN 269 48.650 68.620 70.550 1.00 0.00 C

ATOM 2602 O GLN 269 47.950 69.610 70.340 1.00 0.00 O

ATOM 2603 CB GLN 269 49.620 68.720 72.840 1.00 0.00 C

ATOM 2604 CG GLN 269 49.520 68.400 74.330 1.00 0.00 C

ATOM 2605 CD GLN 269 48.150 68.740 74.930 1.00 0.00 C

ATOM 2606 NE2 GLN 269 47.900 70.000 75.170 1.00 0.00 N

ATOM 2607 OE1 GLN 269 47.280 67.890 75.060 1.00 0.00 O

ATOM 2608 H GLN 269 49.840 66.270 72.420 1.00 0.00 H

ATOM 2609 1HE2 GLN 269 48.540 70.710 74.900 1.00 0.00 H

ATOM 2610 2HE2 GLN 269 47.020 70.220 75.590 1.00 0.00 H

ATOM 2611 N ALA 270 49.590 68.220 69.690 1.00 0.00 N

ATOM 2612 CA ALA 270 49.780 68.800 68.350 1.00 0.00 C

ATOM 2613 C ALA 270 48.560 68.590 67.440 1.00 0.00 C

ATOM 2614 O ALA 270 48.060 67.480 67.320 1.00 0.00 O

ATOM 2615 CB ALA 270 50.990 68.160 67.670 1.00 0.00 C

ATOM 2616 H ALA 270 50.210 67.460 69.920 1.00 0.00 H

ATOM 2617 N ASP 271 48.210 69.640 66.700 1.00 0.00 N

ATOM 2618 CA ASP 271 47.230 69.520 65.610 1.00 0.00 C

ATOM 2619 C ASP 271 47.770 68.560 64.530 1.00 0.00 C

ATOM 2620 O ASP 271 48.950 68.650 64.160 1.00 0.00 O

ATOM 2621 CB ASP 271 46.950 70.860 64.940 1.00 0.00 C

ATOM 2622 CG ASP 271 46.380 72.020 65.770 1.00 0.00 C

ATOM 2623 OD1 ASP 271 45.840 71.810 66.870 1.00 0.00 O

ATOM 2624 OD2 ASP 271 46.430 73.140 65.240 1.00 0.00 O

ATOM 2625 H ASP 271 48.410 70.580 66.970 1.00 0.00 H

ATOM 2626 N ALA 272 46.860 67.810 63.920 1.00 0.00 N

ATOM 2627 CA ALA 272 47.200 66.810 62.880 1.00 0.00 C

ATOM 2628 C ALA 272 48.100 67.350 61.750 1.00 0.00 C

ATOM 2629 O ALA 272 49.190 66.820 61.540 1.00 0.00 O

ATOM 2630 CB ALA 272 45.910 66.240 62.300 1.00 0.00 C

ATOM 2631 H ALA 272 45.910 67.790 64.230 1.00 0.00 H

ATOM 2632 N LYS 273 47.800 68.570 61.290 1.00 0.00 N

ATOM 2633 CA LYS 273 48.600 69.280 60.270 1.00 0.00 C

ATOM 2634 C LYS 273 50.080 69.450 60.670 1.00 0.00 C

ATOM 2635 O LYS 273 50.960 69.300 59.820 1.00 0.00 O

ATOM 2636 CB LYS 273 47.950 70.630 59.930 1.00 0.00 C

ATOM 2637 CG LYS 273 48.720 71.450 58.890 1.00 0.00 C

ATOM 2638 CD LYS 273 47.970 72.720 58.470 1.00 0.00 C

ATOM 2639 CE LYS 273 48.670 73.420 57.300 1.00 0.00 C

ATOM 2640 NZ LYS 273 49.870 74.190 57.670 1.00 0.00 N1+

ATOM 2641 H LYS 273 46.950 69.020 61.570 1.00 0.00 H

ATOM 2642 HZ1 LYS 273 50.260 74.600 56.840 1.00 0.00 H

ATOM 2643 HZ2 LYS 273 49.640 74.900 58.330 1.00 0.00 H

ATOM 2644 HZ3 LYS 273 50.560 73.590 58.070 1.00 0.00 H

ATOM 2645 N LYS 274 50.350 69.690 61.960 1.00 0.00 N

ATOM 2646 CA LYS 274 51.730 69.900 62.450 1.00 0.00 C

ATOM 2647 C LYS 274 52.570 68.620 62.350 1.00 0.00 C

ATOM 2648 O LYS 274 53.660 68.660 61.780 1.00 0.00 O

ATOM 2649 CB LYS 274 51.750 70.450 63.880 1.00 0.00 C

ATOM 2650 CG LYS 274 51.520 71.960 64.010 1.00 0.00 C

ATOM 2651 CD LYS 274 50.060 72.430 63.870 1.00 0.00 C

ATOM 2652 CE LYS 274 49.650 72.810 62.440 1.00 0.00 C

ATOM 2653 NZ LYS 274 48.200 73.040 62.340 1.00 0.00 N1+

ATOM 2654 H LYS 274 49.680 69.460 62.670 1.00 0.00 H

ATOM 2655 HZ1 LYS 274 47.920 73.210 61.400 1.00 0.00 H

ATOM 2656 HZ2 LYS 274 47.670 72.270 62.690 1.00 0.00 H

ATOM 2657 HZ3 LYS 274 47.930 73.850 62.880 1.00 0.00 H

ATOM 2658 N ILE 275 51.950 67.490 62.710 1.00 0.00 N

ATOM 2659 CA ILE 275 52.540 66.140 62.570 1.00 0.00 C

ATOM 2660 C ILE 275 52.810 65.840 61.080 1.00 0.00 C

ATOM 2661 O ILE 275 53.970 65.670 60.710 1.00 0.00 O

ATOM 2662 CB ILE 275 51.610 65.080 63.210 1.00 0.00 C

ATOM 2663 CG1 ILE 275 51.400 65.390 64.700 1.00 0.00 C

ATOM 2664 CG2 ILE 275 52.190 63.670 63.040 1.00 0.00 C

ATOM 2665 CD ILE 275 50.220 64.640 65.330 1.00 0.00 C

ATOM 2666 H ILE 275 51.040 67.500 63.110 1.00 0.00 H

ATOM 2667 N THR 276 51.780 65.970 60.240 1.00 0.00 N

ATOM 2668 CA THR 276 51.880 65.700 58.780 1.00 0.00 C

ATOM 2669 C THR 276 53.020 66.500 58.150 1.00 0.00 C

ATOM 2670 O THR 276 53.920 65.930 57.540 1.00 0.00 O

ATOM 2671 CB THR 276 50.600 66.060 58.020 1.00 0.00 C

ATOM 2672 CG2 THR 276 50.660 65.460 56.610 1.00 0.00 C

ATOM 2673 OG1 THR 276 49.440 65.580 58.710 1.00 0.00 O

ATOM 2674 H THR 276 50.840 66.140 60.560 1.00 0.00 H

ATOM 2675 HG1 THR 276 48.610 65.790 58.160 1.00 0.00 H

ATOM 2676 N ALA 277 53.030 67.810 58.430 1.00 0.00 N

ATOM 2677 CA ALA 277 54.070 68.730 57.950 1.00 0.00 C

ATOM 2678 C ALA 277 55.480 68.190 58.250 1.00 0.00 C

ATOM 2679 O ALA 277 56.290 68.050 57.340 1.00 0.00 O

ATOM 2680 CB ALA 277 53.880 70.080 58.650 1.00 0.00 C

ATOM 2681 H ALA 277 52.300 68.230 58.970 1.00 0.00 H

ATOM 2682 N LYS 278 55.690 67.760 59.490 1.00 0.00 N

ATOM 2683 CA LYS 278 56.990 67.250 59.970 1.00 0.00 C

ATOM 2684 C LYS 278 57.400 65.850 59.470 1.00 0.00 C

ATOM 2685 O LYS 278 58.560 65.640 59.170 1.00 0.00 O

ATOM 2686 CB LYS 278 57.050 67.390 61.490 1.00 0.00 C

ATOM 2687 CG LYS 278 57.030 68.860 61.950 1.00 0.00 C

ATOM 2688 CD LYS 278 58.350 69.580 61.640 1.00 0.00 C

ATOM 2689 CE LYS 278 58.430 71.050 62.080 1.00 0.00 C

ATOM 2690 NZ LYS 278 57.580 71.920 61.280 1.00 0.00 N1+

ATOM 2691 H LYS 278 54.940 67.690 60.160 1.00 0.00 H

ATOM 2692 HZ1 LYS 278 57.720 72.880 61.560 1.00 0.00 H

ATOM 2693 HZ2 LYS 278 57.820 71.820 60.310 1.00 0.00 H

ATOM 2694 HZ3 LYS 278 56.620 71.670 61.430 1.00 0.00 H

ATOM 2695 N GLN 279 56.420 65.040 59.050 1.00 0.00 N

ATOM 2696 CA GLN 279 56.660 63.640 58.630 1.00 0.00 C

ATOM 2697 C GLN 279 57.740 63.430 57.550 1.00 0.00 C

ATOM 2698 O GLN 279 58.470 62.440 57.600 1.00 0.00 O

ATOM 2699 CB GLN 279 55.360 62.990 58.140 1.00 0.00 C

ATOM 2700 CG GLN 279 54.280 62.790 59.210 1.00 0.00 C

ATOM 2701 CD GLN 279 53.020 62.130 58.640 1.00 0.00 C

ATOM 2702 NE2 GLN 279 52.090 61.790 59.500 1.00 0.00 N

ATOM 2703 OE1 GLN 279 52.830 61.940 57.450 1.00 0.00 O

ATOM 2704 H GLN 279 55.470 65.350 58.980 1.00 0.00 H

ATOM 2705 1HE2 GLN 279 52.190 61.970 60.470 1.00 0.00 H

ATOM 2706 2HE2 GLN 279 51.230 61.500 59.090 1.00 0.00 H

ATOM 2707 N TYR 280 57.840 64.360 56.590 1.00 0.00 N

ATOM 2708 CA TYR 280 58.910 64.340 55.580 1.00 0.00 C

ATOM 2709 C TYR 280 60.340 64.670 56.030 1.00 0.00 C

ATOM 2710 O TYR 280 61.280 64.160 55.440 1.00 0.00 O

ATOM 2711 CB TYR 280 58.570 65.180 54.350 1.00 0.00 C

ATOM 2712 CG TYR 280 57.690 64.360 53.420 1.00 0.00 C

ATOM 2713 CD1 TYR 280 58.250 63.380 52.610 1.00 0.00 C

ATOM 2714 CD2 TYR 280 56.310 64.450 53.540 1.00 0.00 C

ATOM 2715 CE1 TYR 280 57.440 62.480 51.930 1.00 0.00 C

ATOM 2716 CE2 TYR 280 55.510 63.560 52.860 1.00 0.00 C

ATOM 2717 CZ TYR 280 56.060 62.580 52.040 1.00 0.00 C

ATOM 2718 OH TYR 280 55.290 61.750 51.300 1.00 0.00 O

ATOM 2719 H TYR 280 57.130 65.050 56.470 1.00 0.00 H

ATOM 2720 HD1 TYR 280 59.330 63.290 52.520 1.00 0.00 H

ATOM 2721 HD2 TYR 280 55.860 65.160 54.230 1.00 0.00 H

ATOM 2722 HE1 TYR 280 57.870 61.620 51.440 1.00 0.00 H

ATOM 2723 HE2 TYR 280 54.490 63.460 53.210 1.00 0.00 H

ATOM 2724 HH TYR 280 54.350 62.100 51.260 1.00 0.00 H

ATOM 2725 N ASP 281 60.480 65.500 57.060 1.00 0.00 N

ATOM 2726 CA ASP 281 61.830 65.900 57.520 1.00 0.00 C

ATOM 2727 C ASP 281 62.630 64.830 58.280 1.00 0.00 C

ATOM 2728 O ASP 281 63.860 64.830 58.260 1.00 0.00 O

ATOM 2729 CB ASP 281 61.820 67.220 58.280 1.00 0.00 C

ATOM 2730 CG ASP 281 60.860 67.390 59.460 1.00 0.00 C

ATOM 2731 OD1 ASP 281 61.000 66.640 60.440 1.00 0.00 O

ATOM 2732 OD2 ASP 281 60.080 68.370 59.390 1.00 0.00 O

ATOM 2733 H ASP 281 59.740 65.740 57.690 1.00 0.00 H

ATOM 2734 N ILE 282 61.900 63.790 58.640 1.00 0.00 N

ATOM 2735 CA ILE 282 62.340 62.660 59.470 1.00 0.00 C

ATOM 2736 C ILE 282 63.540 61.850 58.940 1.00 0.00 C

ATOM 2737 O ILE 282 64.460 61.540 59.700 1.00 0.00 O

ATOM 2738 CB ILE 282 61.080 61.790 59.710 1.00 0.00 C

ATOM 2739 CG1 ILE 282 59.860 62.550 60.280 1.00 0.00 C

ATOM 2740 CG2 ILE 282 61.370 60.590 60.600 1.00 0.00 C

ATOM 2741 CD ILE 282 60.110 63.460 61.490 1.00 0.00 C

ATOM 2742 H ILE 282 60.910 63.800 58.470 1.00 0.00 H

ATOM 2743 N ILE 283 63.550 61.520 57.650 1.00 0.00 N

ATOM 2744 CA ILE 283 64.470 60.500 57.100 1.00 0.00 C

ATOM 2745 C ILE 283 65.930 60.870 56.780 1.00 0.00 C

ATOM 2746 O ILE 283 66.720 59.970 56.470 1.00 0.00 O

ATOM 2747 CB ILE 283 63.880 59.660 55.940 1.00 0.00 C

ATOM 2748 CG1 ILE 283 63.320 60.450 54.750 1.00 0.00 C

ATOM 2749 CG2 ILE 283 62.950 58.580 56.490 1.00 0.00 C

ATOM 2750 CD ILE 283 61.970 61.160 54.930 1.00 0.00 C

ATOM 2751 H ILE 283 63.010 62.030 56.970 1.00 0.00 H

ATOM 2752 N THR 284 66.190 62.170 56.590 1.00 0.00 N

ATOM 2753 CA THR 284 67.500 62.780 56.240 1.00 0.00 C

ATOM 2754 C THR 284 67.500 62.980 54.720 1.00 0.00 C

ATOM 2755 O THR 284 67.150 64.040 54.220 1.00 0.00 O

ATOM 2756 CB THR 284 68.740 62.000 56.750 1.00 0.00 C

ATOM 2757 CG2 THR 284 70.070 62.630 56.340 1.00 0.00 C

ATOM 2758 OG1 THR 284 68.700 61.940 58.170 1.00 0.00 O

ATOM 2759 H THR 284 65.420 62.810 56.470 1.00 0.00 H

ATOM 2760 HG1 THR 284 69.000 61.020 58.480 1.00 0.00 H

ATOM 2761 N LYS 285 67.630 61.840 54.040 1.00 0.00 N

ATOM 2762 CA LYS 285 67.530 61.740 52.580 1.00 0.00 C

ATOM 2763 C LYS 285 66.230 60.980 52.270 1.00 0.00 C

ATOM 2764 O LYS 285 66.110 59.830 52.690 1.00 0.00 O

ATOM 2765 CB LYS 285 68.700 60.920 52.010 1.00 0.00 C

ATOM 2766 CG LYS 285 70.080 61.510 52.300 1.00 0.00 C

ATOM 2767 CD LYS 285 71.150 60.450 52.030 1.00 0.00 C

ATOM 2768 CE LYS 285 72.530 60.960 52.430 1.00 0.00 C

ATOM 2769 NZ LYS 285 73.470 59.850 52.630 1.00 0.00 N1+

ATOM 2770 H LYS 285 67.730 60.970 54.530 1.00 0.00 H

ATOM 2771 HZ1 LYS 285 74.320 60.230 53.010 1.00 0.00 H

ATOM 2772 HZ2 LYS 285 73.080 59.190 53.270 1.00 0.00 H

ATOM 2773 HZ3 LYS 285 73.670 59.410 51.760 1.00 0.00 H

ATOM 2774 N PRO 286 65.190 61.690 51.840 1.00 0.00 N

ATOM 2775 CA PRO 286 64.060 61.030 51.160 1.00 0.00 C

ATOM 2776 C PRO 286 64.520 60.500 49.790 1.00 0.00 C

ATOM 2777 O PRO 286 65.430 61.080 49.190 1.00 0.00 O

ATOM 2778 CB PRO 286 63.020 62.140 50.960 1.00 0.00 C

ATOM 2779 CG PRO 286 63.290 63.090 52.120 1.00 0.00 C

ATOM 2780 CD PRO 286 64.810 63.040 52.290 1.00 0.00 C

ATOM 2781 N PRO 287 64.260 59.220 49.550 1.00 0.00 N

ATOM 2782 CA PRO 287 64.030 58.770 48.160 1.00 0.00 C

ATOM 2783 C PRO 287 62.530 58.850 47.870 1.00 0.00 C

ATOM 2784 O PRO 287 61.700 58.850 48.790 1.00 0.00 O

ATOM 2785 CB PRO 287 64.470 57.310 48.140 1.00 0.00 C

ATOM 2786 CG PRO 287 65.440 57.190 49.310 1.00 0.00 C

ATOM 2787 CD PRO 287 64.830 58.120 50.350 1.00 0.00 C

ATOM 2788 N LEU 288 62.200 58.980 46.600 1.00 0.00 N

ATOM 2789 CA LEU 288 60.910 58.440 46.120 1.00 0.00 C

ATOM 2790 C LEU 288 60.950 56.910 46.320 1.00 0.00 C

ATOM 2791 O LEU 288 62.010 56.300 46.210 1.00 0.00 O

ATOM 2792 CB LEU 288 60.520 58.870 44.690 1.00 0.00 C

ATOM 2793 CG LEU 288 61.500 58.790 43.510 1.00 0.00 C

ATOM 2794 CD1 LEU 288 62.680 59.750 43.610 1.00 0.00 C

ATOM 2795 CD2 LEU 288 61.970 57.360 43.270 1.00 0.00 C

ATOM 2796 H LEU 288 62.710 59.530 45.940 1.00 0.00 H

ATOM 2797 N LEU 289 59.850 56.410 46.870 1.00 0.00 N

ATOM 2798 CA LEU 289 59.740 55.060 47.480 1.00 0.00 C

ATOM 2799 C LEU 289 60.410 54.970 48.860 1.00 0.00 C

ATOM 2800 O LEU 289 61.060 53.990 49.240 1.00 0.00 O

ATOM 2801 CB LEU 289 60.220 53.950 46.550 1.00 0.00 C

ATOM 2802 CG LEU 289 59.230 53.730 45.420 1.00 0.00 C

ATOM 2803 CD1 LEU 289 59.960 53.200 44.200 1.00 0.00 C

ATOM 2804 CD2 LEU 289 58.170 52.730 45.870 1.00 0.00 C

ATOM 2805 H LEU 289 59.030 57.000 46.970 1.00 0.00 H

ATOM 2806 N ASN 290 60.260 56.060 49.610 1.00 0.00 N

ATOM 2807 CA ASN 290 60.590 56.090 51.050 1.00 0.00 C

ATOM 2808 C ASN 290 59.290 56.310 51.830 1.00 0.00 C

ATOM 2809 O ASN 290 58.550 57.260 51.600 1.00 0.00 O

ATOM 2810 CB ASN 290 61.560 57.220 51.360 1.00 0.00 C

ATOM 2811 CG ASN 290 62.040 57.240 52.820 1.00 0.00 C

ATOM 2812 ND2 ASN 290 63.280 56.830 53.000 1.00 0.00 N

ATOM 2813 OD1 ASN 290 61.320 57.520 53.760 1.00 0.00 O

ATOM 2814 H ASN 290 59.980 56.930 49.210 1.00 0.00 H

ATOM 2815 1HD2 ASN 290 63.800 56.500 52.220 1.00 0.00 H

ATOM 2816 2HD2 ASN 290 63.730 56.940 53.890 1.00 0.00 H

ATOM 2817 N PHE 291 59.160 55.480 52.850 1.00 0.00 N

ATOM 2818 CA PHE 291 58.020 55.540 53.770 1.00 0.00 C

ATOM 2819 C PHE 291 58.530 55.850 55.180 1.00 0.00 C

ATOM 2820 O PHE 291 59.150 54.990 55.810 1.00 0.00 O

ATOM 2821 CB PHE 291 57.230 54.230 53.640 1.00 0.00 C

ATOM 2822 CG PHE 291 56.800 53.990 52.180 1.00 0.00 C

ATOM 2823 CD1 PHE 291 56.210 55.010 51.430 1.00 0.00 C

ATOM 2824 CD2 PHE 291 57.180 52.820 51.540 1.00 0.00 C

ATOM 2825 CE1 PHE 291 56.000 54.860 50.060 1.00 0.00 C

ATOM 2826 CE2 PHE 291 56.960 52.670 50.180 1.00 0.00 C

ATOM 2827 CZ PHE 291 56.380 53.680 49.430 1.00 0.00 C

ATOM 2828 H PHE 291 59.740 54.670 52.950 1.00 0.00 H

ATOM 2829 HD1 PHE 291 55.850 55.900 51.930 1.00 0.00 H

ATOM 2830 HD2 PHE 291 57.720 52.050 52.100 1.00 0.00 H

ATOM 2831 HE1 PHE 291 55.330 55.530 49.530 1.00 0.00 H

ATOM 2832 HE2 PHE 291 57.160 51.720 49.690 1.00 0.00 H

ATOM 2833 HZ PHE 291 56.190 53.540 48.370 1.00 0.00 H

ATOM 2834 N PRO 292 58.450 57.120 55.600 1.00 0.00 N

ATOM 2835 CA PRO 292 58.920 57.570 56.930 1.00 0.00 C

ATOM 2836 C PRO 292 58.320 56.750 58.080 1.00 0.00 C

ATOM 2837 O PRO 292 59.070 56.210 58.900 1.00 0.00 O

ATOM 2838 CB PRO 292 58.500 59.040 57.010 1.00 0.00 C

ATOM 2839 CG PRO 292 58.580 59.500 55.560 1.00 0.00 C

ATOM 2840 CD PRO 292 58.050 58.290 54.780 1.00 0.00 C

ATOM 2841 N PHE 293 57.020 56.490 58.000 1.00 0.00 N

ATOM 2842 CA PHE 293 56.310 55.680 59.020 1.00 0.00 C

ATOM 2843 C PHE 293 55.590 54.490 58.380 1.00 0.00 C

ATOM 2844 O PHE 293 54.600 54.670 57.660 1.00 0.00 O

ATOM 2845 CB PHE 293 55.330 56.550 59.820 1.00 0.00 C

ATOM 2846 CG PHE 293 56.060 57.630 60.600 1.00 0.00 C

ATOM 2847 CD1 PHE 293 56.740 57.310 61.770 1.00 0.00 C

ATOM 2848 CD2 PHE 293 56.090 58.930 60.110 1.00 0.00 C

ATOM 2849 CE1 PHE 293 57.460 58.290 62.440 1.00 0.00 C

ATOM 2850 CE2 PHE 293 56.800 59.920 60.800 1.00 0.00 C

ATOM 2851 CZ PHE 293 57.480 59.590 61.960 1.00 0.00 C

ATOM 2852 H PHE 293 56.450 56.770 57.220 1.00 0.00 H

ATOM 2853 HD1 PHE 293 56.700 56.290 62.160 1.00 0.00 H

ATOM 2854 HD2 PHE 293 55.590 59.170 59.170 1.00 0.00 H

ATOM 2855 HE1 PHE 293 58.040 58.030 63.320 1.00 0.00 H

ATOM 2856 HE2 PHE 293 56.840 60.930 60.400 1.00 0.00 H

ATOM 2857 HZ PHE 293 58.020 60.360 62.510 1.00 0.00 H

ATOM 2858 N GLY 294 56.120 53.320 58.690 1.00 0.00 N

ATOM 2859 CA GLY 294 55.620 52.050 58.130 1.00 0.00 C

ATOM 2860 C GLY 294 55.530 50.910 59.170 1.00 0.00 C

ATOM 2861 O GLY 294 55.710 51.150 60.370 1.00 0.00 O

ATOM 2862 H GLY 294 56.760 53.220 59.460 1.00 0.00 H

ATOM 2863 N PRO 295 55.190 49.690 58.730 1.00 0.00 N

ATOM 2864 CA PRO 295 55.180 48.500 59.610 1.00 0.00 C

ATOM 2865 C PRO 295 56.540 48.250 60.270 1.00 0.00 C

ATOM 2866 O PRO 295 57.600 48.460 59.680 1.00 0.00 O

ATOM 2867 CB PRO 295 54.800 47.340 58.700 1.00 0.00 C

ATOM 2868 CG PRO 295 53.850 48.010 57.710 1.00 0.00 C

ATOM 2869 CD PRO 295 54.500 49.370 57.470 1.00 0.00 C

ATOM 2870 N THR 296 56.460 47.890 61.550 1.00 0.00 N

ATOM 2871 CA THR 296 57.620 47.590 62.410 1.00 0.00 C

ATOM 2872 C THR 296 57.520 46.180 63.010 1.00 0.00 C

ATOM 2873 O THR 296 56.450 45.560 62.970 1.00 0.00 O

ATOM 2874 CB THR 296 57.750 48.600 63.570 1.00 0.00 C

ATOM 2875 CG2 THR 296 57.790 50.050 63.080 1.00 0.00 C

ATOM 2876 OG1 THR 296 56.690 48.430 64.510 1.00 0.00 O

ATOM 2877 H THR 296 55.570 47.880 62.020 1.00 0.00 H

ATOM 2878 HG1 THR 296 56.380 49.320 64.850 1.00 0.00 H

ATOM 2879 N VAL 297 58.640 45.670 63.510 1.00 0.00 N

ATOM 2880 CA VAL 297 58.630 44.470 64.370 1.00 0.00 C

ATOM 2881 C VAL 297 58.340 44.950 65.810 1.00 0.00 C

ATOM 2882 O VAL 297 59.200 45.540 66.470 1.00 0.00 O

ATOM 2883 CB VAL 297 59.940 43.660 64.260 1.00 0.00 C

ATOM 2884 CG1 VAL 297 59.870 42.370 65.100 1.00 0.00 C

ATOM 2885 CG2 VAL 297 60.250 43.270 62.810 1.00 0.00 C

ATOM 2886 H VAL 297 59.530 46.100 63.370 1.00 0.00 H

ATOM 2887 N ASP 298 57.040 45.020 66.050 1.00 0.00 N

ATOM 2888 CA ASP 298 56.410 45.510 67.290 1.00 0.00 C

ATOM 2889 C ASP 298 56.390 44.500 68.460 1.00 0.00 C

ATOM 2890 O ASP 298 56.080 44.890 69.590 1.00 0.00 O

ATOM 2891 CB ASP 298 54.980 45.980 66.970 1.00 0.00 C

ATOM 2892 CG ASP 298 54.020 44.920 66.420 1.00 0.00 C

ATOM 2893 OD1 ASP 298 54.450 43.840 65.980 1.00 0.00 O

ATOM 2894 OD2 ASP 298 52.820 45.220 66.290 1.00 0.00 O

ATOM 2895 H ASP 298 56.370 44.690 65.380 1.00 0.00 H

ATOM 2896 N GLY 299 56.400 43.220 68.110 1.00 0.00 N

ATOM 2897 CA GLY 299 56.310 42.110 69.080 1.00 0.00 C

ATOM 2898 C GLY 299 54.890 41.550 69.210 1.00 0.00 C

ATOM 2899 O GLY 299 54.710 40.440 69.730 1.00 0.00 O

ATOM 2900 H GLY 299 56.430 42.940 67.150 1.00 0.00 H

ATOM 2901 N VAL 300 53.890 42.370 68.890 1.00 0.00 N

ATOM 2902 CA VAL 300 52.470 41.950 68.990 1.00 0.00 C

ATOM 2903 C VAL 300 51.870 41.490 67.650 1.00 0.00 C

ATOM 2904 O VAL 300 51.430 40.350 67.560 1.00 0.00 O

ATOM 2905 CB VAL 300 51.560 42.940 69.750 1.00 0.00 C

ATOM 2906 CG1 VAL 300 51.910 42.980 71.240 1.00 0.00 C

ATOM 2907 CG2 VAL 300 51.610 44.360 69.190 1.00 0.00 C

ATOM 2908 H VAL 300 54.070 43.280 68.530 1.00 0.00 H

ATOM 2909 N PHE 301 51.900 42.340 66.620 1.00 0.00 N

ATOM 2910 CA PHE 301 51.410 41.970 65.270 1.00 0.00 C

ATOM 2911 C PHE 301 52.460 41.140 64.520 1.00 0.00 C

ATOM 2912 O PHE 301 52.110 40.110 63.920 1.00 0.00 O

ATOM 2913 CB PHE 301 51.060 43.230 64.470 1.00 0.00 C

ATOM 2914 CG PHE 301 50.260 42.900 63.210 1.00 0.00 C

ATOM 2915 CD1 PHE 301 50.880 42.360 62.100 1.00 0.00 C

ATOM 2916 CD2 PHE 301 48.900 43.180 63.170 1.00 0.00 C

ATOM 2917 CE1 PHE 301 50.160 42.080 60.950 1.00 0.00 C

ATOM 2918 CE2 PHE 301 48.170 42.900 62.010 1.00 0.00 C

ATOM 2919 CZ PHE 301 48.790 42.340 60.910 1.00 0.00 C

ATOM 2920 H PHE 301 52.350 43.240 66.670 1.00 0.00 H

ATOM 2921 HD1 PHE 301 51.960 42.160 62.110 1.00 0.00 H

ATOM 2922 HD2 PHE 301 48.420 43.660 64.020 1.00 0.00 H

ATOM 2923 HE1 PHE 301 50.670 41.670 60.080 1.00 0.00 H

ATOM 2924 HE2 PHE 301 47.160 43.270 61.920 1.00 0.00 H

ATOM 2925 HZ PHE 301 48.220 42.090 60.020 1.00 0.00 H

ATOM 2926 N LEU 302 53.680 41.670 64.490 1.00 0.00 N

ATOM 2927 CA LEU 302 54.900 41.040 63.960 1.00 0.00 C

ATOM 2928 C LEU 302 55.830 40.700 65.120 1.00 0.00 C

ATOM 2929 O LEU 302 56.460 41.540 65.750 1.00 0.00 O

ATOM 2930 CB LEU 302 55.690 41.930 63.000 1.00 0.00 C

ATOM 2931 CG LEU 302 55.150 42.070 61.580 1.00 0.00 C

ATOM 2932 CD1 LEU 302 56.120 42.940 60.800 1.00 0.00 C

ATOM 2933 CD2 LEU 302 54.970 40.720 60.880 1.00 0.00 C

ATOM 2934 H LEU 302 53.870 42.540 64.980 1.00 0.00 H

ATOM 2935 N THR 303 55.810 39.410 65.400 1.00 0.00 N

ATOM 2936 CA THR 303 56.590 38.760 66.470 1.00 0.00 C

ATOM 2937 C THR 303 58.120 38.870 66.250 1.00 0.00 C

ATOM 2938 O THR 303 58.870 39.110 67.200 1.00 0.00 O

ATOM 2939 CB THR 303 56.190 37.280 66.480 1.00 0.00 C

ATOM 2940 CG2 THR 303 54.730 37.070 66.910 1.00 0.00 C

ATOM 2941 OG1 THR 303 56.440 36.750 65.170 1.00 0.00 O

ATOM 2942 H THR 303 55.280 38.770 64.850 1.00 0.00 H

ATOM 2943 HG1 THR 303 56.060 35.780 65.070 1.00 0.00 H

ATOM 2944 N ALA 304 58.540 38.660 65.010 1.00 0.00 N

ATOM 2945 CA ALA 304 59.960 38.620 64.580 1.00 0.00 C

ATOM 2946 C ALA 304 60.140 39.170 63.150 1.00 0.00 C

ATOM 2947 O ALA 304 59.170 39.590 62.510 1.00 0.00 O

ATOM 2948 CB ALA 304 60.420 37.160 64.650 1.00 0.00 C

ATOM 2949 H ALA 304 57.880 38.440 64.290 1.00 0.00 H

ATOM 2950 N GLU 305 61.380 39.110 62.650 1.00 0.00 N

ATOM 2951 CA GLU 305 61.740 39.510 61.270 1.00 0.00 C

ATOM 2952 C GLU 305 60.840 38.790 60.250 1.00 0.00 C

ATOM 2953 O GLU 305 60.720 37.560 60.250 1.00 0.00 O

ATOM 2954 CB GLU 305 63.180 39.130 60.900 1.00 0.00 C

ATOM 2955 CG GLU 305 64.290 39.380 61.930 1.00 0.00 C

ATOM 2956 CD GLU 305 64.320 38.310 63.040 1.00 0.00 C

ATOM 2957 OE1 GLU 305 64.730 37.170 62.780 1.00 0.00 O

ATOM 2958 OE2 GLU 305 63.900 38.650 64.160 1.00 0.00 O

ATOM 2959 H GLU 305 62.170 38.840 63.220 1.00 0.00 H

ATOM 2960 N VAL 306 60.360 39.570 59.270 1.00 0.00 N

ATOM 2961 CA VAL 306 59.540 39.060 58.150 1.00 0.00 C

ATOM 2962 C VAL 306 60.210 37.860 57.450 1.00 0.00 C

ATOM 2963 O VAL 306 59.600 36.800 57.300 1.00 0.00 O

ATOM 2964 CB VAL 306 59.170 40.210 57.170 1.00 0.00 C

ATOM 2965 CG1 VAL 306 60.370 40.990 56.620 1.00 0.00 C

ATOM 2966 CG2 VAL 306 58.320 39.710 55.990 1.00 0.00 C

ATOM 2967 H VAL 306 60.470 40.560 59.310 1.00 0.00 H

ATOM 2968 N LYS 307 61.500 38.000 57.200 1.00 0.00 N

ATOM 2969 CA LYS 307 62.290 36.950 56.530 1.00 0.00 C

ATOM 2970 C LYS 307 62.480 35.640 57.300 1.00 0.00 C

ATOM 2971 O LYS 307 62.190 34.600 56.730 1.00 0.00 O

ATOM 2972 CB LYS 307 63.600 37.500 55.980 1.00 0.00 C

ATOM 2973 CG LYS 307 63.320 38.260 54.670 1.00 0.00 C

ATOM 2974 CD LYS 307 62.800 37.300 53.600 1.00 0.00 C

ATOM 2975 CE LYS 307 62.470 38.080 52.330 1.00 0.00 C

ATOM 2976 NZ LYS 307 61.890 37.220 51.290 1.00 0.00 N1+

ATOM 2977 H LYS 307 62.000 38.840 57.400 1.00 0.00 H

ATOM 2978 HZ1 LYS 307 61.710 37.760 50.470 1.00 0.00 H

ATOM 2979 HZ2 LYS 307 62.570 36.530 51.010 1.00 0.00 H

ATOM 2980 HZ3 LYS 307 61.050 36.780 51.600 1.00 0.00 H

ATOM 2981 N LYS 308 62.690 35.720 58.620 1.00 0.00 N

ATOM 2982 CA LYS 308 62.700 34.510 59.460 1.00 0.00 C

ATOM 2983 C LYS 308 61.330 33.820 59.410 1.00 0.00 C

ATOM 2984 O LYS 308 61.250 32.610 59.180 1.00 0.00 O

ATOM 2985 CB LYS 308 63.100 34.820 60.920 1.00 0.00 C

ATOM 2986 CG LYS 308 63.700 33.600 61.630 1.00 0.00 C

ATOM 2987 CD LYS 308 62.720 32.440 61.870 1.00 0.00 C

ATOM 2988 CE LYS 308 63.430 31.120 62.130 1.00 0.00 C

ATOM 2989 NZ LYS 308 62.510 29.970 62.040 1.00 0.00 N1+

ATOM 2990 H LYS 308 62.860 36.590 59.090 1.00 0.00 H

ATOM 2991 HZ1 LYS 308 62.950 29.150 62.420 1.00 0.00 H

ATOM 2992 HZ2 LYS 308 61.660 30.160 62.540 1.00 0.00 H

ATOM 2993 HZ3 LYS 308 62.260 29.820 61.090 1.00 0.00 H

ATOM 2994 N LEU 309 60.250 34.590 59.520 1.00 0.00 N

ATOM 2995 CA LEU 309 58.880 34.050 59.430 1.00 0.00 C

ATOM 2996 C LEU 309 58.630 33.320 58.100 1.00 0.00 C

ATOM 2997 O LEU 309 58.220 32.150 58.100 1.00 0.00 O

ATOM 2998 CB LEU 309 57.820 35.140 59.650 1.00 0.00 C

ATOM 2999 CG LEU 309 57.940 35.880 60.990 1.00 0.00 C

ATOM 3000 CD1 LEU 309 56.860 36.960 61.070 1.00 0.00 C

ATOM 3001 CD2 LEU 309 57.840 34.940 62.200 1.00 0.00 C

ATOM 3002 H LEU 309 60.310 35.580 59.720 1.00 0.00 H

ATOM 3003 N LEU 310 59.100 33.940 57.010 1.00 0.00 N

ATOM 3004 CA LEU 310 59.070 33.360 55.660 1.00 0.00 C

ATOM 3005 C LEU 310 59.900 32.070 55.530 1.00 0.00 C

ATOM 3006 O LEU 310 59.330 31.010 55.280 1.00 0.00 O

ATOM 3007 CB LEU 310 59.510 34.390 54.600 1.00 0.00 C

ATOM 3008 CG LEU 310 58.600 35.620 54.530 1.00 0.00 C

ATOM 3009 CD1 LEU 310 59.080 36.570 53.430 1.00 0.00 C

ATOM 3010 CD2 LEU 310 57.150 35.220 54.260 1.00 0.00 C

ATOM 3011 H LEU 310 59.430 34.890 57.070 1.00 0.00 H

ATOM 3012 N THR 311 61.180 32.160 55.890 1.00 0.00 N

ATOM 3013 CA THR 311 62.100 31.000 55.870 1.00 0.00 C

ATOM 3014 C THR 311 61.990 30.210 57.180 1.00 0.00 C

ATOM 3015 O THR 311 62.640 30.450 58.210 1.00 0.00 O

ATOM 3016 CB THR 311 63.530 31.390 55.460 1.00 0.00 C

ATOM 3017 CG2 THR 311 64.280 32.290 56.460 1.00 0.00 C

ATOM 3018 OG1 THR 311 64.280 30.200 55.200 1.00 0.00 O

ATOM 3019 H THR 311 61.570 33.010 56.260 1.00 0.00 H

ATOM 3020 HG1 THR 311 65.250 30.440 55.170 1.00 0.00 H

ATOM 3021 N GLY 312 61.070 29.260 57.100 1.00 0.00 N

ATOM 3022 CA GLY 312 60.560 28.480 58.240 1.00 0.00 C

ATOM 3023 C GLY 312 59.020 28.340 58.270 1.00 0.00 C

ATOM 3024 O GLY 312 58.480 27.950 59.310 1.00 0.00 O

ATOM 3025 H GLY 312 60.710 28.980 56.200 1.00 0.00 H

ATOM 3026 N GLY 313 58.360 28.950 57.290 1.00 0.00 N

ATOM 3027 CA GLY 313 56.930 28.750 56.990 1.00 0.00 C

ATOM 3028 C GLY 313 55.970 29.150 58.120 1.00 0.00 C

ATOM 3029 O GLY 313 54.840 28.650 58.190 1.00 0.00 O

ATOM 3030 H GLY 313 58.810 29.620 56.690 1.00 0.00 H

ATOM 3031 N ASN 314 56.350 30.190 58.850 1.00 0.00 N

ATOM 3032 CA ASN 314 55.500 30.690 59.940 1.00 0.00 C

ATOM 3033 C ASN 314 54.560 31.770 59.410 1.00 0.00 C

ATOM 3034 O ASN 314 54.850 32.960 59.360 1.00 0.00 O

ATOM 3035 CB ASN 314 56.300 31.160 61.160 1.00 0.00 C

ATOM 3036 CG ASN 314 55.350 31.350 62.350 1.00 0.00 C

ATOM 3037 ND2 ASN 314 55.860 31.950 63.380 1.00 0.00 N

ATOM 3038 OD1 ASN 314 54.200 30.920 62.370 1.00 0.00 O

ATOM 3039 H ASN 314 57.140 30.760 58.620 1.00 0.00 H

ATOM 3040 1HD2 ASN 314 56.810 32.260 63.380 1.00 0.00 H

ATOM 3041 2HD2 ASN 314 55.390 31.890 64.260 1.00 0.00 H

ATOM 3042 N LEU 315 53.550 31.180 58.790 1.00 0.00 N

ATOM 3043 CA LEU 315 52.410 31.840 58.140 1.00 0.00 C

ATOM 3044 C LEU 315 51.260 30.830 57.940 1.00 0.00 C

ATOM 3045 O LEU 315 51.520 29.620 57.890 1.00 0.00 O

ATOM 3046 CB LEU 315 52.850 32.460 56.800 1.00 0.00 C

ATOM 3047 CG LEU 315 53.620 31.520 55.850 1.00 0.00 C

ATOM 3048 CD1 LEU 315 52.680 30.630 55.030 1.00 0.00 C

ATOM 3049 CD2 LEU 315 54.550 32.320 54.950 1.00 0.00 C

ATOM 3050 H LEU 315 53.620 30.200 58.590 1.00 0.00 H

ATOM 3051 N PRO 316 50.030 31.310 58.100 1.00 0.00 N

ATOM 3052 CA PRO 316 48.810 30.690 57.570 1.00 0.00 C

ATOM 3053 C PRO 316 48.980 30.100 56.160 1.00 0.00 C

ATOM 3054 O PRO 316 49.400 30.790 55.230 1.00 0.00 O

ATOM 3055 CB PRO 316 47.830 31.860 57.560 1.00 0.00 C

ATOM 3056 CG PRO 316 48.180 32.610 58.840 1.00 0.00 C

ATOM 3057 CD PRO 316 49.640 32.290 59.140 1.00 0.00 C

ATOM 3058 N LYS 317 48.650 28.820 56.090 1.00 0.00 N

ATOM 3059 CA LYS 317 48.680 28.030 54.850 1.00 0.00 C

ATOM 3060 C LYS 317 47.310 28.130 54.170 1.00 0.00 C

ATOM 3061 O LYS 317 46.270 27.960 54.810 1.00 0.00 O

ATOM 3062 CB LYS 317 48.980 26.550 55.100 1.00 0.00 C

ATOM 3063 CG LYS 317 50.150 26.250 56.050 1.00 0.00 C

ATOM 3064 CD LYS 317 51.470 26.910 55.650 1.00 0.00 C

ATOM 3065 CE LYS 317 52.600 26.490 56.590 1.00 0.00 C

ATOM 3066 NZ LYS 317 52.340 26.800 58.000 1.00 0.00 N1+

ATOM 3067 H LYS 317 48.300 28.330 56.910 1.00 0.00 H

ATOM 3068 HZ1 LYS 317 53.100 26.470 58.560 1.00 0.00 H

ATOM 3069 HZ2 LYS 317 52.240 27.790 58.120 1.00 0.00 H

ATOM 3070 HZ3 LYS 317 51.510 26.340 58.320 1.00 0.00 H

ATOM 3071 N LYS 318 47.400 28.640 52.950 1.00 0.00 N

ATOM 3072 CA LYS 318 46.260 28.970 52.060 1.00 0.00 C

ATOM 3073 C LYS 318 46.790 29.330 50.670 1.00 0.00 C

ATOM 3074 O LYS 318 47.950 29.700 50.510 1.00 0.00 O

ATOM 3075 CB LYS 318 45.470 30.160 52.620 1.00 0.00 C

ATOM 3076 CG LYS 318 46.310 31.440 52.770 1.00 0.00 C

ATOM 3077 CD LYS 318 45.530 32.590 53.390 1.00 0.00 C

ATOM 3078 CE LYS 318 45.280 32.360 54.880 1.00 0.00 C

ATOM 3079 NZ LYS 318 44.220 33.270 55.300 1.00 0.00 N1+

ATOM 3080 H LYS 318 48.290 28.800 52.540 1.00 0.00 H

ATOM 3081 HZ1 LYS 318 43.960 33.110 56.260 1.00 0.00 H

ATOM 3082 HZ2 LYS 318 44.500 34.230 55.180 1.00 0.00 H

ATOM 3083 HZ3 LYS 318 43.420 33.090 54.730 1.00 0.00 H

ATOM 3084 N GLU 319 45.890 29.310 49.690 1.00 0.00 N

ATOM 3085 CA GLU 319 46.180 29.740 48.320 1.00 0.00 C

ATOM 3086 C GLU 319 46.440 31.260 48.240 1.00 0.00 C

ATOM 3087 O GLU 319 45.800 32.060 48.930 1.00 0.00 O

ATOM 3088 CB GLU 319 45.020 29.360 47.380 1.00 0.00 C

ATOM 3089 CG GLU 319 44.690 27.860 47.320 1.00 0.00 C

ATOM 3090 CD GLU 319 44.030 27.280 48.580 1.00 0.00 C

ATOM 3091 OE1 GLU 319 43.380 28.040 49.320 1.00 0.00 O

ATOM 3092 OE2 GLU 319 44.160 26.050 48.800 1.00 0.00 O

ATOM 3093 H GLU 319 44.990 28.870 49.800 1.00 0.00 H

ATOM 3094 N LEU 320 47.360 31.600 47.350 1.00 0.00 N

ATOM 3095 CA LEU 320 47.710 33.000 47.020 1.00 0.00 C

ATOM 3096 C LEU 320 47.640 33.270 45.510 1.00 0.00 C

ATOM 3097 O LEU 320 48.020 32.430 44.690 1.00 0.00 O

ATOM 3098 CB LEU 320 49.110 33.300 47.570 1.00 0.00 C

ATOM 3099 CG LEU 320 49.640 34.720 47.290 1.00 0.00 C

ATOM 3100 CD1 LEU 320 48.880 35.790 48.060 1.00 0.00 C

ATOM 3101 CD2 LEU 320 51.130 34.790 47.600 1.00 0.00 C

ATOM 3102 H LEU 320 47.870 30.900 46.840 1.00 0.00 H

ATOM 3103 N MET 321 47.120 34.440 45.180 1.00 0.00 N

ATOM 3104 CA MET 321 47.260 35.040 43.840 1.00 0.00 C

ATOM 3105 C MET 321 48.080 36.330 44.010 1.00 0.00 C

ATOM 3106 O MET 321 47.860 37.110 44.940 1.00 0.00 O

ATOM 3107 CB MET 321 45.900 35.370 43.220 1.00 0.00 C

ATOM 3108 CG MET 321 46.030 35.830 41.760 1.00 0.00 C

ATOM 3109 SD MET 321 44.610 36.800 41.140 1.00 0.00 S

ATOM 3110 CE MET 321 43.400 35.530 40.870 1.00 0.00 C

ATOM 3111 H MET 321 46.630 35.010 45.850 1.00 0.00 H

ATOM 3112 N ILE 322 49.170 36.390 43.250 1.00 0.00 N

ATOM 3113 CA ILE 322 50.130 37.510 43.310 1.00 0.00 C

ATOM 3114 C ILE 322 50.630 37.890 41.910 1.00 0.00 C

ATOM 3115 O ILE 322 50.600 37.080 40.990 1.00 0.00 O

ATOM 3116 CB ILE 322 51.220 37.120 44.330 1.00 0.00 C

ATOM 3117 CG1 ILE 322 51.940 38.370 44.850 1.00 0.00 C

ATOM 3118 CG2 ILE 322 52.180 36.040 43.800 1.00 0.00 C

ATOM 3119 CD ILE 322 52.740 38.150 46.140 1.00 0.00 C

ATOM 3120 H ILE 322 49.390 35.670 42.580 1.00 0.00 H

ATOM 3121 N GLY 323 50.970 39.170 41.750 1.00 0.00 N

ATOM 3122 CA GLY 323 51.530 39.680 40.490 1.00 0.00 C

ATOM 3123 C GLY 323 51.960 41.150 40.500 1.00 0.00 C

ATOM 3124 O GLY 323 52.010 41.780 41.560 1.00 0.00 O

ATOM 3125 H GLY 323 50.790 39.860 42.460 1.00 0.00 H

ATOM 3126 N LEU 324 52.530 41.490 39.350 1.00 0.00 N

ATOM 3127 CA LEU 324 53.090 42.820 39.030 1.00 0.00 C

ATOM 3128 C LEU 324 52.640 43.280 37.640 1.00 0.00 C

ATOM 3129 O LEU 324 52.410 42.460 36.750 1.00 0.00 O

ATOM 3130 CB LEU 324 54.630 42.840 38.970 1.00 0.00 C

ATOM 3131 CG LEU 324 55.440 42.700 40.260 1.00 0.00 C

ATOM 3132 CD1 LEU 324 54.980 43.620 41.380 1.00 0.00 C

ATOM 3133 CD2 LEU 324 55.510 41.230 40.670 1.00 0.00 C

ATOM 3134 H LEU 324 52.490 40.870 38.570 1.00 0.00 H

ATOM 3135 N ASN 325 52.870 44.570 37.440 1.00 0.00 N

ATOM 3136 CA ASN 325 52.790 45.240 36.130 1.00 0.00 C

ATOM 3137 C ASN 325 54.200 45.340 35.520 1.00 0.00 C

ATOM 3138 O ASN 325 55.220 45.030 36.140 1.00 0.00 O

ATOM 3139 CB ASN 325 52.160 46.630 36.300 1.00 0.00 C

ATOM 3140 CG ASN 325 50.750 46.580 36.910 1.00 0.00 C

ATOM 3141 ND2 ASN 325 50.310 47.710 37.410 1.00 0.00 N

ATOM 3142 OD1 ASN 325 50.060 45.570 36.970 1.00 0.00 O

ATOM 3143 H ASN 325 53.160 45.180 38.200 1.00 0.00 H

ATOM 3144 1HD2 ASN 325 50.840 48.550 37.340 1.00 0.00 H

ATOM 3145 2HD2 ASN 325 49.500 47.660 38.030 1.00 0.00 H

ATOM 3146 N LYS 326 54.200 45.530 34.210 1.00 0.00 N

ATOM 3147 CA LYS 326 55.440 45.620 33.420 1.00 0.00 C

ATOM 3148 C LYS 326 56.410 46.730 33.860 1.00 0.00 C

ATOM 3149 O LYS 326 57.590 46.440 34.080 1.00 0.00 O

ATOM 3150 CB LYS 326 55.060 45.730 31.940 1.00 0.00 C

ATOM 3151 CG LYS 326 56.280 45.670 31.030 1.00 0.00 C

ATOM 3152 CD LYS 326 55.960 45.100 29.640 1.00 0.00 C

ATOM 3153 CE LYS 326 55.110 46.020 28.770 1.00 0.00 C

ATOM 3154 NZ LYS 326 55.080 45.470 27.410 1.00 0.00 N1+

ATOM 3155 H LYS 326 53.340 45.470 33.690 1.00 0.00 H

ATOM 3156 HZ1 LYS 326 54.670 46.120 26.760 1.00 0.00 H

ATOM 3157 HZ2 LYS 326 56.000 45.260 27.110 1.00 0.00 H

ATOM 3158 HZ3 LYS 326 54.510 44.650 27.380 1.00 0.00 H

ATOM 3159 N ASP 327 55.850 47.900 34.170 1.00 0.00 N

ATOM 3160 CA ASP 327 56.650 49.120 34.390 1.00 0.00 C

ATOM 3161 C ASP 327 56.320 49.790 35.740 1.00 0.00 C

ATOM 3162 O ASP 327 56.000 50.980 35.840 1.00 0.00 O

ATOM 3163 CB ASP 327 56.440 50.020 33.160 1.00 0.00 C

ATOM 3164 CG ASP 327 56.770 49.320 31.820 1.00 0.00 C

ATOM 3165 OD1 ASP 327 57.810 48.630 31.750 1.00 0.00 O

ATOM 3166 OD2 ASP 327 55.890 49.360 30.930 1.00 0.00 O

ATOM 3167 H ASP 327 54.860 48.050 34.170 1.00 0.00 H

ATOM 3168 N GLU 328 56.530 49.000 36.790 1.00 0.00 N

ATOM 3169 CA GLU 328 56.370 49.380 38.210 1.00 0.00 C

ATOM 3170 C GLU 328 57.160 50.630 38.620 1.00 0.00 C

ATOM 3171 O GLU 328 56.610 51.590 39.150 1.00 0.00 O

ATOM 3172 CB GLU 328 56.880 48.230 39.100 1.00 0.00 C

ATOM 3173 CG GLU 328 56.040 46.960 39.070 1.00 0.00 C

ATOM 3174 CD GLU 328 54.640 47.130 39.670 1.00 0.00 C

ATOM 3175 OE1 GLU 328 54.410 48.080 40.440 1.00 0.00 O

ATOM 3176 OE2 GLU 328 53.800 46.280 39.340 1.00 0.00 O

ATOM 3177 H GLU 328 56.780 48.040 36.650 1.00 0.00 H

ATOM 3178 N GLY 329 58.410 50.670 38.170 1.00 0.00 N

ATOM 3179 CA GLY 329 59.400 51.700 38.540 1.00 0.00 C

ATOM 3180 C GLY 329 59.140 53.120 38.020 1.00 0.00 C

ATOM 3181 O GLY 329 59.390 54.110 38.720 1.00 0.00 O

ATOM 3182 H GLY 329 58.750 49.950 37.540 1.00 0.00 H

ATOM 3183 N THR 330 58.530 53.190 36.850 1.00 0.00 N

ATOM 3184 CA THR 330 58.410 54.420 36.050 1.00 0.00 C

ATOM 3185 C THR 330 57.790 55.640 36.760 1.00 0.00 C

ATOM 3186 O THR 330 58.410 56.700 36.750 1.00 0.00 O

ATOM 3187 CB THR 330 57.650 54.150 34.750 1.00 0.00 C

ATOM 3188 CG2 THR 330 58.190 55.110 33.700 1.00 0.00 C

ATOM 3189 OG1 THR 330 57.930 52.830 34.280 1.00 0.00 O

ATOM 3190 H THR 330 58.190 52.380 36.360 1.00 0.00 H

ATOM 3191 HG1 THR 330 57.600 52.730 33.330 1.00 0.00 H

ATOM 3192 N CYS 331 56.680 55.440 37.470 1.00 0.00 N

ATOM 3193 CA CYS 331 55.960 56.540 38.140 1.00 0.00 C

ATOM 3194 C CYS 331 56.870 57.360 39.060 1.00 0.00 C

ATOM 3195 O CYS 331 57.080 58.540 38.810 1.00 0.00 O

ATOM 3196 CB CYS 331 54.770 56.000 38.940 1.00 0.00 C

ATOM 3197 SG CYS 331 53.660 57.360 39.470 1.00 0.00 S

ATOM 3198 H CYS 331 56.200 54.540 37.490 1.00 0.00 H

ATOM 3199 HG CYS 331 52.900 56.610 40.270 1.00 0.00 H

ATOM 3200 N PHE 332 57.690 56.620 39.800 1.00 0.00 N

ATOM 3201 CA PHE 332 58.610 57.180 40.810 1.00 0.00 C

ATOM 3202 C PHE 332 59.710 58.090 40.240 1.00 0.00 C

ATOM 3203 O PHE 332 60.000 59.140 40.800 1.00 0.00 O

ATOM 3204 CB PHE 332 59.180 56.010 41.610 1.00 0.00 C

ATOM 3205 CG PHE 332 58.040 55.300 42.340 1.00 0.00 C

ATOM 3206 CD1 PHE 332 57.510 55.870 43.500 1.00 0.00 C

ATOM 3207 CD2 PHE 332 57.460 54.160 41.800 1.00 0.00 C

ATOM 3208 CE1 PHE 332 56.400 55.290 44.100 1.00 0.00 C

ATOM 3209 CE2 PHE 332 56.340 53.590 42.400 1.00 0.00 C

ATOM 3210 CZ PHE 332 55.810 54.160 43.550 1.00 0.00 C

ATOM 3211 H PHE 332 57.770 55.640 39.660 1.00 0.00 H

ATOM 3212 HD1 PHE 332 57.950 56.780 43.910 1.00 0.00 H

ATOM 3213 HD2 PHE 332 57.900 53.690 40.920 1.00 0.00 H

ATOM 3214 HE1 PHE 332 55.960 55.740 44.990 1.00 0.00 H

ATOM 3215 HE2 PHE 332 55.890 52.700 41.970 1.00 0.00 H

ATOM 3216 HZ PHE 332 54.950 53.700 44.030 1.00 0.00 H

ATOM 3217 N LEU 333 60.110 57.810 39.000 1.00 0.00 N

ATOM 3218 CA LEU 333 61.180 58.570 38.320 1.00 0.00 C

ATOM 3219 C LEU 333 60.850 60.050 38.080 1.00 0.00 C

ATOM 3220 O LEU 333 61.660 60.900 38.430 1.00 0.00 O

ATOM 3221 CB LEU 333 61.550 57.890 36.990 1.00 0.00 C

ATOM 3222 CG LEU 333 61.910 56.420 37.170 1.00 0.00 C

ATOM 3223 CD1 LEU 333 62.340 55.860 35.820 1.00 0.00 C

ATOM 3224 CD2 LEU 333 63.000 56.210 38.230 1.00 0.00 C

ATOM 3225 H LEU 333 59.680 57.090 38.460 1.00 0.00 H

ATOM 3226 N VAL 334 59.590 60.310 37.740 1.00 0.00 N

ATOM 3227 CA VAL 334 59.100 61.680 37.490 1.00 0.00 C

ATOM 3228 C VAL 334 59.140 62.590 38.740 1.00 0.00 C

ATOM 3229 O VAL 334 59.250 63.810 38.610 1.00 0.00 O

ATOM 3230 CB VAL 334 57.720 61.720 36.800 1.00 0.00 C

ATOM 3231 CG1 VAL 334 57.740 60.950 35.480 1.00 0.00 C

ATOM 3232 CG2 VAL 334 56.540 61.270 37.670 1.00 0.00 C

ATOM 3233 H VAL 334 58.900 59.580 37.670 1.00 0.00 H

ATOM 3234 N TYR 335 59.110 61.960 39.910 1.00 0.00 N

ATOM 3235 CA TYR 335 59.110 62.620 41.230 1.00 0.00 C

ATOM 3236 C TYR 335 60.420 63.340 41.590 1.00 0.00 C

ATOM 3237 O TYR 335 60.390 64.550 41.810 1.00 0.00 O

ATOM 3238 CB TYR 335 58.700 61.610 42.310 1.00 0.00 C

ATOM 3239 CG TYR 335 57.200 61.310 42.240 1.00 0.00 C

ATOM 3240 CD1 TYR 335 56.680 60.480 41.260 1.00 0.00 C

ATOM 3241 CD2 TYR 335 56.340 62.070 43.010 1.00 0.00 C

ATOM 3242 CE1 TYR 335 55.310 60.450 41.020 1.00 0.00 C

ATOM 3243 CE2 TYR 335 54.970 62.050 42.780 1.00 0.00 C

ATOM 3244 CZ TYR 335 54.450 61.250 41.770 1.00 0.00 C

ATOM 3245 OH TYR 335 53.160 61.400 41.410 1.00 0.00 O

ATOM 3246 H TYR 335 59.100 60.960 39.960 1.00 0.00 H

ATOM 3247 HD1 TYR 335 57.340 59.790 40.740 1.00 0.00 H

ATOM 3248 HD2 TYR 335 56.750 62.660 43.820 1.00 0.00 H

ATOM 3249 HE1 TYR 335 54.910 59.780 40.260 1.00 0.00 H

ATOM 3250 HE2 TYR 335 54.310 62.660 43.410 1.00 0.00 H

ATOM 3251 HH TYR 335 52.640 61.840 42.180 1.00 0.00 H

ATOM 3252 N GLY 336 61.530 62.610 41.470 1.00 0.00 N

ATOM 3253 CA GLY 336 62.860 63.090 41.910 1.00 0.00 C

ATOM 3254 C GLY 336 64.020 63.010 40.890 1.00 0.00 C

ATOM 3255 O GLY 336 64.930 63.830 40.940 1.00 0.00 O

ATOM 3256 H GLY 336 61.510 61.710 41.030 1.00 0.00 H

ATOM 3257 N SER 337 63.950 62.060 39.960 1.00 0.00 N

ATOM 3258 CA SER 337 65.110 61.690 39.130 1.00 0.00 C

ATOM 3259 C SER 337 65.400 62.750 38.040 1.00 0.00 C

ATOM 3260 O SER 337 64.520 63.060 37.240 1.00 0.00 O

ATOM 3261 CB SER 337 64.910 60.320 38.490 1.00 0.00 C

ATOM 3262 OG SER 337 64.920 59.260 39.460 1.00 0.00 O

ATOM 3263 H SER 337 63.070 61.810 39.550 1.00 0.00 H

ATOM 3264 HG SER 337 64.660 58.410 38.990 1.00 0.00 H

ATOM 3265 N PRO 338 66.650 63.220 37.940 1.00 0.00 N

ATOM 3266 CA PRO 338 67.020 64.300 37.000 1.00 0.00 C

ATOM 3267 C PRO 338 66.840 63.870 35.540 1.00 0.00 C

ATOM 3268 O PRO 338 66.960 62.690 35.210 1.00 0.00 O

ATOM 3269 CB PRO 338 68.490 64.610 37.320 1.00 0.00 C

ATOM 3270 CG PRO 338 69.030 63.260 37.790 1.00 0.00 C

ATOM 3271 CD PRO 338 67.860 62.670 38.580 1.00 0.00 C

ATOM 3272 N GLY 339 66.330 64.820 34.770 1.00 0.00 N

ATOM 3273 CA GLY 339 66.060 64.660 33.320 1.00 0.00 C

ATOM 3274 C GLY 339 64.760 63.910 32.990 1.00 0.00 C

ATOM 3275 O GLY 339 64.460 63.700 31.820 1.00 0.00 O

ATOM 3276 H GLY 339 66.040 65.700 35.150 1.00 0.00 H

ATOM 3277 N PHE 340 63.980 63.570 34.020 1.00 0.00 N

ATOM 3278 CA PHE 340 62.670 62.930 33.840 1.00 0.00 C

ATOM 3279 C PHE 340 61.520 63.940 33.900 1.00 0.00 C

ATOM 3280 O PHE 340 61.440 64.800 34.770 1.00 0.00 O

ATOM 3281 CB PHE 340 62.410 61.830 34.880 1.00 0.00 C

ATOM 3282 CG PHE 340 63.260 60.590 34.600 1.00 0.00 C

ATOM 3283 CD1 PHE 340 64.500 60.450 35.200 1.00 0.00 C

ATOM 3284 CD2 PHE 340 62.770 59.590 33.770 1.00 0.00 C

ATOM 3285 CE1 PHE 340 65.230 59.290 35.020 1.00 0.00 C

ATOM 3286 CE2 PHE 340 63.510 58.430 33.580 1.00 0.00 C

ATOM 3287 CZ PHE 340 64.740 58.270 34.210 1.00 0.00 C

ATOM 3288 H PHE 340 64.260 63.690 34.970 1.00 0.00 H

ATOM 3289 HD1 PHE 340 64.950 61.300 35.710 1.00 0.00 H

ATOM 3290 HD2 PHE 340 61.870 59.770 33.190 1.00 0.00 H

ATOM 3291 HE1 PHE 340 66.240 59.220 35.440 1.00 0.00 H

ATOM 3292 HE2 PHE 340 63.150 57.670 32.880 1.00 0.00 H

ATOM 3293 HZ PHE 340 65.350 57.390 34.000 1.00 0.00 H

ATOM 3294 N SER 341 60.690 63.830 32.870 1.00 0.00 N

ATOM 3295 CA SER 341 59.450 64.610 32.750 1.00 0.00 C

ATOM 3296 C SER 341 58.300 63.820 32.090 1.00 0.00 C

ATOM 3297 O SER 341 58.490 62.930 31.270 1.00 0.00 O

ATOM 3298 CB SER 341 59.710 65.930 32.020 1.00 0.00 C

ATOM 3299 OG SER 341 59.960 65.790 30.620 1.00 0.00 O

ATOM 3300 H SER 341 60.890 63.210 32.110 1.00 0.00 H

ATOM 3301 HG SER 341 60.270 66.700 30.290 1.00 0.00 H

ATOM 3302 N ILE 342 57.110 64.100 32.610 1.00 0.00 N

ATOM 3303 CA ILE 342 55.800 63.630 32.110 1.00 0.00 C

ATOM 3304 C ILE 342 55.560 64.220 30.700 1.00 0.00 C

ATOM 3305 O ILE 342 55.040 63.590 29.780 1.00 0.00 O

ATOM 3306 CB ILE 342 54.700 64.110 33.080 1.00 0.00 C

ATOM 3307 CG1 ILE 342 54.880 63.560 34.500 1.00 0.00 C

ATOM 3308 CG2 ILE 342 53.270 63.780 32.650 1.00 0.00 C

ATOM 3309 CD ILE 342 55.900 64.360 35.310 1.00 0.00 C

ATOM 3310 H ILE 342 57.050 64.600 33.470 1.00 0.00 H

ATOM 3311 N THR 343 55.940 65.480 30.610 1.00 0.00 N

ATOM 3312 CA THR 343 55.810 66.360 29.430 1.00 0.00 C

ATOM 3313 C THR 343 56.680 65.970 28.220 1.00 0.00 C

ATOM 3314 O THR 343 56.380 66.420 27.110 1.00 0.00 O

ATOM 3315 CB THR 343 56.090 67.810 29.840 1.00 0.00 C

ATOM 3316 CG2 THR 343 55.050 68.280 30.860 1.00 0.00 C

ATOM 3317 OG1 THR 343 57.410 67.920 30.400 1.00 0.00 O

ATOM 3318 H THR 343 56.270 65.960 31.420 1.00 0.00 H

ATOM 3319 HG1 THR 343 57.730 68.870 30.280 1.00 0.00 H

ATOM 3320 N GLY 344 57.800 65.290 28.450 1.00 0.00 N

ATOM 3321 CA GLY 344 58.720 64.880 27.380 1.00 0.00 C

ATOM 3322 C GLY 344 59.060 63.380 27.370 1.00 0.00 C

ATOM 3323 O GLY 344 58.630 62.600 28.220 1.00 0.00 O

ATOM 3324 H GLY 344 58.070 64.990 29.370 1.00 0.00 H

ATOM 3325 N GLN 345 59.950 63.050 26.430 1.00 0.00 N

ATOM 3326 CA GLN 345 60.470 61.690 26.220 1.00 0.00 C

ATOM 3327 C GLN 345 61.270 61.110 27.400 1.00 0.00 C

ATOM 3328 O GLN 345 61.370 59.890 27.520 1.00 0.00 O

ATOM 3329 CB GLN 345 61.380 61.640 24.980 1.00 0.00 C

ATOM 3330 CG GLN 345 60.670 61.970 23.660 1.00 0.00 C

ATOM 3331 CD GLN 345 61.630 61.800 22.470 1.00 0.00 C

ATOM 3332 NE2 GLN 345 61.110 61.410 21.330 1.00 0.00 N

ATOM 3333 OE1 GLN 345 62.820 62.100 22.520 1.00 0.00 O

ATOM 3334 H GLN 345 60.300 63.740 25.800 1.00 0.00 H

ATOM 3335 1HE2 GLN 345 60.110 61.340 21.210 1.00 0.00 H

ATOM 3336 2HE2 GLN 345 61.760 61.120 20.640 1.00 0.00 H

ATOM 3337 N SER 346 61.900 61.990 28.180 1.00 0.00 N

ATOM 3338 CA SER 346 62.820 61.660 29.290 1.00 0.00 C

ATOM 3339 C SER 346 64.070 60.880 28.850 1.00 0.00 C

ATOM 3340 O SER 346 64.450 59.860 29.420 1.00 0.00 O

ATOM 3341 CB SER 346 62.110 60.970 30.460 1.00 0.00 C

ATOM 3342 OG SER 346 61.110 61.820 31.020 1.00 0.00 O

ATOM 3343 H SER 346 61.690 62.980 28.130 1.00 0.00 H

ATOM 3344 HG SER 346 60.270 61.280 31.170 1.00 0.00 H

ATOM 3345 N LEU 347 64.620 61.280 27.710 1.00 0.00 N

ATOM 3346 CA LEU 347 65.900 60.740 27.210 1.00 0.00 C

ATOM 3347 C LEU 347 67.060 61.080 28.170 1.00 0.00 C

ATOM 3348 O LEU 347 67.510 62.230 28.260 1.00 0.00 O

ATOM 3349 CB LEU 347 66.190 61.250 25.790 1.00 0.00 C

ATOM 3350 CG LEU 347 65.260 60.730 24.680 1.00 0.00 C

ATOM 3351 CD1 LEU 347 65.810 61.230 23.350 1.00 0.00 C

ATOM 3352 CD2 LEU 347 65.120 59.200 24.650 1.00 0.00 C

ATOM 3353 H LEU 347 64.180 61.950 27.120 1.00 0.00 H

ATOM 3354 N ILE 348 67.520 60.050 28.870 1.00 0.00 N

ATOM 3355 CA ILE 348 68.450 60.200 30.010 1.00 0.00 C

ATOM 3356 C ILE 348 69.920 60.040 29.570 1.00 0.00 C

ATOM 3357 O ILE 348 70.270 59.070 28.910 1.00 0.00 O

ATOM 3358 CB ILE 348 68.030 59.200 31.110 1.00 0.00 C

ATOM 3359 CG1 ILE 348 66.620 59.500 31.650 1.00 0.00 C

ATOM 3360 CG2 ILE 348 69.020 59.040 32.280 1.00 0.00 C

ATOM 3361 CD ILE 348 66.370 60.930 32.150 1.00 0.00 C

ATOM 3362 H ILE 348 67.200 59.120 28.700 1.00 0.00 H

ATOM 3363 N SER 349 70.750 60.980 30.030 1.00 0.00 N

ATOM 3364 CA SER 349 72.210 60.920 29.800 1.00 0.00 C

ATOM 3365 C SER 349 72.850 59.890 30.740 1.00 0.00 C

ATOM 3366 O SER 349 72.250 59.490 31.750 1.00 0.00 O

ATOM 3367 CB SER 349 72.910 62.290 29.960 1.00 0.00 C

ATOM 3368 OG SER 349 73.240 62.600 31.320 1.00 0.00 O

ATOM 3369 H SER 349 70.450 61.710 30.650 1.00 0.00 H

ATOM 3370 HG SER 349 72.580 63.330 31.640 1.00 0.00 H

ATOM 3371 N ARG 350 74.060 59.460 30.410 1.00 0.00 N

ATOM 3372 CA ARG 350 74.860 58.600 31.310 1.00 0.00 C

ATOM 3373 C ARG 350 75.010 59.220 32.710 1.00 0.00 C

ATOM 3374 O ARG 350 74.820 58.540 33.720 1.00 0.00 O

ATOM 3375 CB ARG 350 76.250 58.360 30.680 1.00 0.00 C

ATOM 3376 CG ARG 350 76.800 56.970 30.980 1.00 0.00 C

ATOM 3377 CD ARG 350 77.160 56.790 32.460 1.00 0.00 C

ATOM 3378 NE ARG 350 77.430 55.390 32.790 1.00 0.00 N

ATOM 3379 CZ ARG 350 76.530 54.400 32.940 1.00 0.00 C

ATOM 3380 NH1 ARG 350 75.220 54.610 32.770 1.00 0.00 N

ATOM 3381 NH2 ARG 350 76.960 53.180 33.250 1.00 0.00 N

ATOM 3382 H ARG 350 74.410 59.520 29.470 1.00 0.00 H

ATOM 3383 HE ARG 350 78.420 55.180 32.920 1.00 0.00 H

ATOM 3384 1HH1 ARG 350 74.880 55.520 32.540 1.00 0.00 H

ATOM 3385 2HH1 ARG 350 74.560 53.860 32.870 1.00 0.00 H

ATOM 3386 1HH2 ARG 350 77.940 53.000 33.370 1.00 0.00 H

ATOM 3387 2HH2 ARG 350 76.320 52.420 33.380 1.00 0.00 H

ATOM 3388 N ARG 351 75.200 60.540 32.730 1.00 0.00 N

ATOM 3389 CA ARG 351 75.380 61.310 33.980 1.00 0.00 C

ATOM 3390 C ARG 351 74.100 61.330 34.830 1.00 0.00 C

ATOM 3391 O ARG 351 74.150 60.960 36.010 1.00 0.00 O

ATOM 3392 CB ARG 351 75.840 62.740 33.650 1.00 0.00 C

ATOM 3393 CG ARG 351 77.100 62.770 32.780 1.00 0.00 C

ATOM 3394 CD ARG 351 77.770 64.150 32.760 1.00 0.00 C

ATOM 3395 NE ARG 351 78.910 64.210 33.700 1.00 0.00 N

ATOM 3396 CZ ARG 351 78.870 64.290 35.050 1.00 0.00 C

ATOM 3397 NH1 ARG 351 77.720 64.350 35.730 1.00 0.00 N

ATOM 3398 NH2 ARG 351 80.010 64.310 35.740 1.00 0.00 N

ATOM 3399 H ARG 351 75.150 61.090 31.900 1.00 0.00 H

ATOM 3400 HE ARG 351 79.800 64.210 33.260 1.00 0.00 H

ATOM 3401 1HH1 ARG 351 76.850 64.400 35.250 1.00 0.00 H

ATOM 3402 2HH1 ARG 351 77.720 64.310 36.730 1.00 0.00 H

ATOM 3403 1HH2 ARG 351 80.890 64.240 35.280 1.00 0.00 H

ATOM 3404 2HH2 ARG 351 80.000 64.470 36.730 1.00 0.00 H

ATOM 3405 N ASP 352 72.970 61.570 34.170 1.00 0.00 N

ATOM 3406 CA ASP 352 71.630 61.530 34.800 1.00 0.00 C

ATOM 3407 C ASP 352 71.240 60.130 35.290 1.00 0.00 C

ATOM 3408 O ASP 352 70.770 59.990 36.420 1.00 0.00 O

ATOM 3409 CB ASP 352 70.560 62.070 33.850 1.00 0.00 C

ATOM 3410 CG ASP 352 70.870 63.500 33.410 1.00 0.00 C

ATOM 3411 OD1 ASP 352 71.280 64.300 34.280 1.00 0.00 O

ATOM 3412 OD2 ASP 352 70.950 63.690 32.180 1.00 0.00 O

ATOM 3413 H ASP 352 72.960 61.950 33.240 1.00 0.00 H

ATOM 3414 N PHE 353 71.630 59.100 34.540 1.00 0.00 N

ATOM 3415 CA PHE 353 71.500 57.680 34.950 1.00 0.00 C

ATOM 3416 C PHE 353 72.160 57.440 36.320 1.00 0.00 C

ATOM 3417 O PHE 353 71.480 57.060 37.280 1.00 0.00 O

ATOM 3418 CB PHE 353 72.140 56.800 33.860 1.00 0.00 C

ATOM 3419 CG PHE 353 72.120 55.300 34.200 1.00 0.00 C

ATOM 3420 CD1 PHE 353 71.030 54.530 33.830 1.00 0.00 C

ATOM 3421 CD2 PHE 353 73.100 54.760 35.010 1.00 0.00 C

ATOM 3422 CE1 PHE 353 70.910 53.230 34.300 1.00 0.00 C

ATOM 3423 CE2 PHE 353 73.000 53.450 35.460 1.00 0.00 C

ATOM 3424 CZ PHE 353 71.900 52.680 35.090 1.00 0.00 C

ATOM 3425 H PHE 353 71.920 59.240 33.580 1.00 0.00 H

ATOM 3426 HD1 PHE 353 70.230 54.960 33.230 1.00 0.00 H

ATOM 3427 HD2 PHE 353 73.930 55.380 35.350 1.00 0.00 H

ATOM 3428 HE1 PHE 353 69.940 52.740 34.240 1.00 0.00 H

ATOM 3429 HE2 PHE 353 73.720 53.040 36.160 1.00 0.00 H

ATOM 3430 HZ PHE 353 71.860 51.630 35.360 1.00 0.00 H

ATOM 3431 N LEU 354 73.420 57.860 36.440 1.00 0.00 N

ATOM 3432 CA LEU 354 74.210 57.720 37.680 1.00 0.00 C

ATOM 3433 C LEU 354 73.600 58.530 38.840 1.00 0.00 C

ATOM 3434 O LEU 354 73.390 57.990 39.930 1.00 0.00 O

ATOM 3435 CB LEU 354 75.660 58.140 37.430 1.00 0.00 C

ATOM 3436 CG LEU 354 76.330 57.380 36.270 1.00 0.00 C

ATOM 3437 CD1 LEU 354 77.750 57.890 36.060 1.00 0.00 C

ATOM 3438 CD2 LEU 354 76.430 55.880 36.530 1.00 0.00 C

ATOM 3439 H LEU 354 73.890 58.290 35.660 1.00 0.00 H

ATOM 3440 N ALA 355 73.160 59.740 38.530 1.00 0.00 N

ATOM 3441 CA ALA 355 72.450 60.630 39.480 1.00 0.00 C

ATOM 3442 C ALA 355 71.110 60.030 39.960 1.00 0.00 C

ATOM 3443 O ALA 355 70.890 59.940 41.170 1.00 0.00 O

ATOM 3444 CB ALA 355 72.250 61.990 38.810 1.00 0.00 C

ATOM 3445 H ALA 355 73.260 60.110 37.600 1.00 0.00 H

ATOM 3446 N GLY 356 70.360 59.440 39.030 1.00 0.00 N

ATOM 3447 CA GLY 356 69.120 58.690 39.270 1.00 0.00 C

ATOM 3448 C GLY 356 69.320 57.620 40.350 1.00 0.00 C

ATOM 3449 O GLY 356 68.870 57.830 41.480 1.00 0.00 O

ATOM 3450 H GLY 356 70.570 59.560 38.050 1.00 0.00 H

ATOM 3451 N VAL 357 70.320 56.770 40.110 1.00 0.00 N

ATOM 3452 CA VAL 357 70.740 55.670 41.020 1.00 0.00 C

ATOM 3453 C VAL 357 71.020 56.200 42.440 1.00 0.00 C

ATOM 3454 O VAL 357 70.510 55.660 43.420 1.00 0.00 O

ATOM 3455 CB VAL 357 71.980 54.940 40.470 1.00 0.00 C

ATOM 3456 CG1 VAL 357 72.450 53.800 41.370 1.00 0.00 C

ATOM 3457 CG2 VAL 357 71.730 54.370 39.070 1.00 0.00 C

ATOM 3458 H VAL 357 70.840 56.820 39.250 1.00 0.00 H

ATOM 3459 N ASN 358 71.760 57.300 42.520 1.00 0.00 N

ATOM 3460 CA ASN 358 72.130 57.940 43.800 1.00 0.00 C

ATOM 3461 C ASN 358 70.930 58.420 44.630 1.00 0.00 C

ATOM 3462 O ASN 358 70.960 58.310 45.860 1.00 0.00 O

ATOM 3463 CB ASN 358 73.080 59.110 43.540 1.00 0.00 C

ATOM 3464 CG ASN 358 74.420 58.670 42.940 1.00 0.00 C

ATOM 3465 ND2 ASN 358 74.990 59.520 42.130 1.00 0.00 N

ATOM 3466 OD1 ASN 358 74.990 57.620 43.230 1.00 0.00 O

ATOM 3467 H ASN 358 72.120 57.740 41.690 1.00 0.00 H

ATOM 3468 1HD2 ASN 358 74.530 60.380 41.910 1.00 0.00 H

ATOM 3469 2HD2 ASN 358 75.800 59.220 41.630 1.00 0.00 H

ATOM 3470 N LEU 359 69.910 58.950 43.960 1.00 0.00 N

ATOM 3471 CA LEU 359 68.630 59.360 44.590 1.00 0.00 C

ATOM 3472 C LEU 359 67.780 58.170 45.080 1.00 0.00 C

ATOM 3473 O LEU 359 67.100 58.260 46.100 1.00 0.00 O

ATOM 3474 CB LEU 359 67.790 60.180 43.600 1.00 0.00 C

ATOM 3475 CG LEU 359 68.450 61.490 43.160 1.00 0.00 C

ATOM 3476 CD1 LEU 359 67.540 62.190 42.160 1.00 0.00 C

ATOM 3477 CD2 LEU 359 68.700 62.410 44.350 1.00 0.00 C

ATOM 3478 H LEU 359 69.970 59.120 42.970 1.00 0.00 H

ATOM 3479 N THR 360 67.790 57.130 44.250 1.00 0.00 N

ATOM 3480 CA THR 360 67.130 55.830 44.440 1.00 0.00 C

ATOM 3481 C THR 360 67.740 55.050 45.620 1.00 0.00 C

ATOM 3482 O THR 360 67.030 54.530 46.480 1.00 0.00 O

ATOM 3483 CB THR 360 67.300 55.060 43.110 1.00 0.00 C

ATOM 3484 CG2 THR 360 66.430 53.810 43.000 1.00 0.00 C

ATOM 3485 OG1 THR 360 66.990 55.940 42.030 1.00 0.00 O

ATOM 3486 H THR 360 68.140 57.240 43.310 1.00 0.00 H

ATOM 3487 HG1 THR 360 67.030 55.430 41.130 1.00 0.00 H

ATOM 3488 N LEU 361 69.080 55.010 45.650 1.00 0.00 N

ATOM 3489 CA LEU 361 69.880 54.330 46.690 1.00 0.00 C

ATOM 3490 C LEU 361 70.860 55.320 47.370 1.00 0.00 C

ATOM 3491 O LEU 361 72.080 55.280 47.210 1.00 0.00 O

ATOM 3492 CB LEU 361 70.650 53.140 46.080 1.00 0.00 C

ATOM 3493 CG LEU 361 69.810 52.030 45.430 1.00 0.00 C

ATOM 3494 CD1 LEU 361 69.650 52.260 43.930 1.00 0.00 C

ATOM 3495 CD2 LEU 361 70.480 50.670 45.670 1.00 0.00 C

ATOM 3496 H LEU 361 69.610 55.360 44.880 1.00 0.00 H

ATOM 3497 N THR 362 70.280 56.200 48.180 1.00 0.00 N

ATOM 3498 CA THR 362 71.030 57.250 48.910 1.00 0.00 C

ATOM 3499 C THR 362 71.930 56.730 50.040 1.00 0.00 C

ATOM 3500 O THR 362 72.860 57.430 50.460 1.00 0.00 O

ATOM 3501 CB THR 362 70.100 58.320 49.500 1.00 0.00 C

ATOM 3502 CG2 THR 362 69.300 59.080 48.440 1.00 0.00 C

ATOM 3503 OG1 THR 362 69.210 57.700 50.440 1.00 0.00 O

ATOM 3504 H THR 362 69.280 56.270 48.240 1.00 0.00 H

ATOM 3505 HG1 THR 362 69.220 58.230 51.300 1.00 0.00 H

ATOM 3506 N ASN 363 71.410 55.710 50.720 1.00 0.00 N

ATOM 3507 CA ASN 363 72.110 54.980 51.800 1.00 0.00 C

ATOM 3508 C ASN 363 73.250 54.080 51.310 1.00 0.00 C

ATOM 3509 O ASN 363 74.280 53.980 51.960 1.00 0.00 O

ATOM 3510 CB ASN 363 71.100 54.160 52.630 1.00 0.00 C

ATOM 3511 CG ASN 363 70.050 55.110 53.200 1.00 0.00 C

ATOM 3512 ND2 ASN 363 68.870 55.080 52.630 1.00 0.00 N

ATOM 3513 OD1 ASN 363 70.270 55.830 54.170 1.00 0.00 O

ATOM 3514 H ASN 363 70.440 55.510 50.640 1.00 0.00 H

ATOM 3515 1HD2 ASN 363 68.600 54.290 52.060 1.00 0.00 H

ATOM 3516 2HD2 ASN 363 68.270 55.880 52.730 1.00 0.00 H

ATOM 3517 N ALA 364 73.090 53.600 50.070 1.00 0.00 N

ATOM 3518 CA ALA 364 74.060 52.700 49.420 1.00 0.00 C

ATOM 3519 C ALA 364 75.480 53.270 49.340 1.00 0.00 C

ATOM 3520 O ALA 364 75.700 54.430 48.970 1.00 0.00 O

ATOM 3521 CB ALA 364 73.560 52.390 48.010 1.00 0.00 C

ATOM 3522 H ALA 364 72.300 53.810 49.510 1.00 0.00 H

ATOM 3523 N HIS 365 76.410 52.410 49.710 1.00 0.00 N

ATOM 3524 CA HIS 365 77.850 52.610 49.460 1.00 0.00 C

ATOM 3525 C HIS 365 78.100 52.660 47.950 1.00 0.00 C

ATOM 3526 O HIS 365 77.320 52.140 47.150 1.00 0.00 O

ATOM 3527 CB HIS 365 78.710 51.490 50.040 1.00 0.00 C

ATOM 3528 CG HIS 365 78.690 51.500 51.570 1.00 0.00 C

ATOM 3529 CD2 HIS 365 78.050 50.610 52.320 1.00 0.00 C

ATOM 3530 ND1 HIS 365 79.220 52.440 52.350 1.00 0.00 N

ATOM 3531 CE1 HIS 365 78.880 52.130 53.600 1.00 0.00 C

ATOM 3532 NE2 HIS 365 78.160 51.020 53.580 1.00 0.00 N

ATOM 3533 H HIS 365 76.170 51.540 50.150 1.00 0.00 H

ATOM 3534 HE2 HIS 365 77.680 50.610 54.370 1.00 0.00 H

ATOM 3535 N SER 366 79.200 53.310 47.600 1.00 0.00 N

ATOM 3536 CA SER 366 79.580 53.480 46.180 1.00 0.00 C

ATOM 3537 C SER 366 79.670 52.150 45.410 1.00 0.00 C

ATOM 3538 O SER 366 79.010 52.040 44.380 1.00 0.00 O

ATOM 3539 CB SER 366 80.870 54.280 46.090 1.00 0.00 C

ATOM 3540 OG SER 366 80.700 55.550 46.730 1.00 0.00 O

ATOM 3541 H SER 366 79.800 53.750 48.260 1.00 0.00 H

ATOM 3542 HG SER 366 81.530 56.090 46.530 1.00 0.00 H

ATOM 3543 N ILE 367 80.130 51.110 46.100 1.00 0.00 N

ATOM 3544 CA ILE 367 80.220 49.730 45.570 1.00 0.00 C

ATOM 3545 C ILE 367 78.860 49.150 45.130 1.00 0.00 C

ATOM 3546 O ILE 367 78.700 48.700 44.000 1.00 0.00 O

ATOM 3547 CB ILE 367 80.990 48.820 46.550 1.00 0.00 C

ATOM 3548 CG1 ILE 367 81.310 47.470 45.890 1.00 0.00 C

ATOM 3549 CG2 ILE 367 80.300 48.660 47.920 1.00 0.00 C

ATOM 3550 CD ILE 367 82.440 46.700 46.580 1.00 0.00 C

ATOM 3551 H ILE 367 80.590 51.240 46.980 1.00 0.00 H

ATOM 3552 N ILE 368 77.850 49.360 45.980 1.00 0.00 N

ATOM 3553 CA ILE 368 76.440 48.980 45.730 1.00 0.00 C

ATOM 3554 C ILE 368 75.950 49.670 44.450 1.00 0.00 C

ATOM 3555 O ILE 368 75.570 49.020 43.480 1.00 0.00 O

ATOM 3556 CB ILE 368 75.550 49.380 46.910 1.00 0.00 C

ATOM 3557 CG1 ILE 368 76.100 48.840 48.230 1.00 0.00 C

ATOM 3558 CG2 ILE 368 74.110 48.900 46.670 1.00 0.00 C

ATOM 3559 CD ILE 368 75.440 49.430 49.480 1.00 0.00 C

ATOM 3560 H ILE 368 78.010 49.760 46.880 1.00 0.00 H

ATOM 3561 N LYS 369 76.110 50.990 44.450 1.00 0.00 N

ATOM 3562 CA LYS 369 75.720 51.850 43.320 1.00 0.00 C

ATOM 3563 C LYS 369 76.380 51.420 42.010 1.00 0.00 C

ATOM 3564 O LYS 369 75.660 51.050 41.090 1.00 0.00 O

ATOM 3565 CB LYS 369 76.010 53.320 43.660 1.00 0.00 C

ATOM 3566 CG LYS 369 75.080 53.790 44.780 1.00 0.00 C

ATOM 3567 CD LYS 369 75.410 55.210 45.260 1.00 0.00 C

ATOM 3568 CE LYS 369 76.670 55.280 46.130 1.00 0.00 C

ATOM 3569 NZ LYS 369 77.080 56.670 46.380 1.00 0.00 N1+

ATOM 3570 H LYS 369 76.530 51.460 45.230 1.00 0.00 H

ATOM 3571 HZ1 LYS 369 77.890 56.700 46.970 1.00 0.00 H

ATOM 3572 HZ2 LYS 369 77.330 57.110 45.510 1.00 0.00 H

ATOM 3573 HZ3 LYS 369 76.320 57.180 46.790 1.00 0.00 H

ATOM 3574 N GLU 370 77.690 51.150 42.060 1.00 0.00 N

ATOM 3575 CA GLU 370 78.460 50.620 40.910 1.00 0.00 C

ATOM 3576 C GLU 370 77.930 49.280 40.390 1.00 0.00 C

ATOM 3577 O GLU 370 77.670 49.150 39.190 1.00 0.00 O

ATOM 3578 CB GLU 370 79.920 50.370 41.280 1.00 0.00 C

ATOM 3579 CG GLU 370 80.700 51.610 41.710 1.00 0.00 C

ATOM 3580 CD GLU 370 82.120 51.190 42.060 1.00 0.00 C

ATOM 3581 OE1 GLU 370 82.310 50.670 43.180 1.00 0.00 O

ATOM 3582 OE2 GLU 370 83.010 51.490 41.230 1.00 0.00 O

ATOM 3583 H GLU 370 78.230 51.340 42.880 1.00 0.00 H

ATOM 3584 N ALA 371 77.720 48.340 41.310 1.00 0.00 N

ATOM 3585 CA ALA 371 77.180 47.000 41.020 1.00 0.00 C

ATOM 3586 C ALA 371 75.800 47.070 40.340 1.00 0.00 C

ATOM 3587 O ALA 371 75.670 46.680 39.180 1.00 0.00 O

ATOM 3588 CB ALA 371 77.120 46.210 42.330 1.00 0.00 C

ATOM 3589 H ALA 371 77.970 48.500 42.270 1.00 0.00 H

ATOM 3590 N VAL 372 74.940 47.900 40.930 1.00 0.00 N

ATOM 3591 CA VAL 372 73.620 48.290 40.400 1.00 0.00 C

ATOM 3592 C VAL 372 73.730 48.860 38.970 1.00 0.00 C

ATOM 3593 O VAL 372 73.210 48.270 38.020 1.00 0.00 O

ATOM 3594 CB VAL 372 73.040 49.260 41.460 1.00 0.00 C

ATOM 3595 CG1 VAL 372 71.930 50.190 40.980 1.00 0.00 C

ATOM 3596 CG2 VAL 372 72.460 48.450 42.630 1.00 0.00 C

ATOM 3597 H VAL 372 75.170 48.310 41.820 1.00 0.00 H

ATOM 3598 N ILE 373 74.610 49.850 38.810 1.00 0.00 N

ATOM 3599 CA ILE 373 74.910 50.510 37.520 1.00 0.00 C

ATOM 3600 C ILE 373 75.390 49.500 36.450 1.00 0.00 C

ATOM 3601 O ILE 373 74.900 49.510 35.330 1.00 0.00 O

ATOM 3602 CB ILE 373 75.910 51.670 37.780 1.00 0.00 C

ATOM 3603 CG1 ILE 373 75.190 52.770 38.570 1.00 0.00 C

ATOM 3604 CG2 ILE 373 76.510 52.240 36.490 1.00 0.00 C

ATOM 3605 CD ILE 373 76.110 53.690 39.370 1.00 0.00 C

ATOM 3606 H ILE 373 75.070 50.260 39.610 1.00 0.00 H

ATOM 3607 N PHE 374 76.280 48.600 36.860 1.00 0.00 N

ATOM 3608 CA PHE 374 76.880 47.590 35.970 1.00 0.00 C

ATOM 3609 C PHE 374 75.830 46.630 35.410 1.00 0.00 C

ATOM 3610 O PHE 374 75.590 46.630 34.200 1.00 0.00 O

ATOM 3611 CB PHE 374 77.940 46.780 36.720 1.00 0.00 C

ATOM 3612 CG PHE 374 78.890 46.160 35.700 1.00 0.00 C

ATOM 3613 CD1 PHE 374 78.650 44.910 35.130 1.00 0.00 C

ATOM 3614 CD2 PHE 374 79.930 46.940 35.240 1.00 0.00 C

ATOM 3615 CE1 PHE 374 79.470 44.470 34.100 1.00 0.00 C

ATOM 3616 CE2 PHE 374 80.760 46.480 34.240 1.00 0.00 C

ATOM 3617 CZ PHE 374 80.520 45.250 33.650 1.00 0.00 C

ATOM 3618 H PHE 374 76.570 48.540 37.820 1.00 0.00 H

ATOM 3619 HD1 PHE 374 77.830 44.290 35.490 1.00 0.00 H

ATOM 3620 HD2 PHE 374 80.010 47.970 35.560 1.00 0.00 H

ATOM 3621 HE1 PHE 374 79.280 43.500 33.640 1.00 0.00 H

ATOM 3622 HE2 PHE 374 81.670 47.040 34.020 1.00 0.00 H

ATOM 3623 HZ PHE 374 81.160 44.910 32.830 1.00 0.00 H

ATOM 3624 N GLU 375 75.080 46.020 36.330 1.00 0.00 N

ATOM 3625 CA GLU 375 74.030 45.040 36.010 1.00 0.00 C

ATOM 3626 C GLU 375 72.940 45.600 35.080 1.00 0.00 C

ATOM 3627 O GLU 375 72.410 44.900 34.220 1.00 0.00 O

ATOM 3628 CB GLU 375 73.380 44.520 37.300 1.00 0.00 C

ATOM 3629 CG GLU 375 74.390 43.890 38.270 1.00 0.00 C

ATOM 3630 CD GLU 375 75.210 42.720 37.680 1.00 0.00 C

ATOM 3631 OE1 GLU 375 74.630 41.880 36.950 1.00 0.00 O

ATOM 3632 OE2 GLU 375 76.420 42.640 38.020 1.00 0.00 O

ATOM 3633 H GLU 375 75.270 46.130 37.310 1.00 0.00 H

ATOM 3634 N TYR 376 72.670 46.900 35.240 1.00 0.00 N

ATOM 3635 CA TYR 376 71.680 47.620 34.430 1.00 0.00 C

ATOM 3636 C TYR 376 72.140 48.290 33.130 1.00 0.00 C

ATOM 3637 O TYR 376 71.290 48.770 32.370 1.00 0.00 O

ATOM 3638 CB TYR 376 70.910 48.600 35.330 1.00 0.00 C

ATOM 3639 CG TYR 376 69.830 47.830 36.070 1.00 0.00 C

ATOM 3640 CD1 TYR 376 70.150 47.180 37.250 1.00 0.00 C

ATOM 3641 CD2 TYR 376 68.560 47.690 35.530 1.00 0.00 C

ATOM 3642 CE1 TYR 376 69.210 46.390 37.890 1.00 0.00 C

ATOM 3643 CE2 TYR 376 67.610 46.900 36.160 1.00 0.00 C

ATOM 3644 CZ TYR 376 67.940 46.250 37.340 1.00 0.00 C

ATOM 3645 OH TYR 376 67.080 45.400 37.940 1.00 0.00 O

ATOM 3646 H TYR 376 73.070 47.420 36.000 1.00 0.00 H

ATOM 3647 HD1 TYR 376 71.140 47.280 37.680 1.00 0.00 H

ATOM 3648 HD2 TYR 376 68.310 48.230 34.610 1.00 0.00 H

ATOM 3649 HE1 TYR 376 69.470 45.900 38.830 1.00 0.00 H

ATOM 3650 HE2 TYR 376 66.620 46.770 35.740 1.00 0.00 H

ATOM 3651 HH TYR 376 66.180 45.300 37.430 1.00 0.00 H

ATOM 3652 N THR 377 73.450 48.390 32.880 1.00 0.00 N

ATOM 3653 CA THR 377 73.910 49.030 31.630 1.00 0.00 C

ATOM 3654 C THR 377 74.660 48.130 30.630 1.00 0.00 C

ATOM 3655 O THR 377 75.530 48.610 29.890 1.00 0.00 O

ATOM 3656 CB THR 377 74.620 50.380 31.820 1.00 0.00 C

ATOM 3657 CG2 THR 377 73.750 51.400 32.560 1.00 0.00 C

ATOM 3658 OG1 THR 377 75.900 50.250 32.450 1.00 0.00 O

ATOM 3659 H THR 377 74.160 48.120 33.540 1.00 0.00 H

ATOM 3660 HG1 THR 377 76.580 50.270 31.710 1.00 0.00 H

ATOM 3661 N ASP 378 74.270 46.860 30.560 1.00 0.00 N

ATOM 3662 CA ASP 378 74.610 46.000 29.410 1.00 0.00 C

ATOM 3663 C ASP 378 73.750 46.480 28.230 1.00 0.00 C

ATOM 3664 O ASP 378 72.540 46.740 28.340 1.00 0.00 O

ATOM 3665 CB ASP 378 74.390 44.510 29.720 1.00 0.00 C

ATOM 3666 CG ASP 378 72.950 44.020 29.540 1.00 0.00 C

ATOM 3667 OD1 ASP 378 72.080 44.440 30.340 1.00 0.00 O

ATOM 3668 OD2 ASP 378 72.700 43.340 28.520 1.00 0.00 O

ATOM 3669 H ASP 378 73.660 46.420 31.230 1.00 0.00 H

ATOM 3670 N TRP 379 74.430 46.830 27.160 1.00 0.00 N

ATOM 3671 CA TRP 379 73.750 47.440 26.010 1.00 0.00 C

ATOM 3672 C TRP 379 73.850 46.530 24.790 1.00 0.00 C

ATOM 3673 O TRP 379 74.950 46.180 24.370 1.00 0.00 O

ATOM 3674 CB TRP 379 74.320 48.820 25.660 1.00 0.00 C

ATOM 3675 CG TRP 379 74.500 49.820 26.820 1.00 0.00 C

ATOM 3676 CD1 TRP 379 75.580 50.580 26.920 1.00 0.00 C

ATOM 3677 CD2 TRP 379 73.560 50.360 27.670 1.00 0.00 C

ATOM 3678 CE2 TRP 379 74.120 51.500 28.220 1.00 0.00 C

ATOM 3679 CE3 TRP 379 72.280 49.980 28.070 1.00 0.00 C

ATOM 3680 NE1 TRP 379 75.350 51.600 27.740 1.00 0.00 N

ATOM 3681 CZ2 TRP 379 73.410 52.280 29.120 1.00 0.00 C

ATOM 3682 CZ3 TRP 379 71.560 50.750 28.970 1.00 0.00 C

ATOM 3683 CH2 TRP 379 72.120 51.910 29.490 1.00 0.00 C

ATOM 3684 H TRP 379 75.410 46.640 27.050 1.00 0.00 H

ATOM 3685 HD1 TRP 379 76.530 50.380 26.430 1.00 0.00 H

ATOM 3686 HE1 TRP 379 76.010 52.310 27.980 1.00 0.00 H

ATOM 3687 HE3 TRP 379 71.870 49.030 27.730 1.00 0.00 H

ATOM 3688 HZ2 TRP 379 73.870 53.150 29.580 1.00 0.00 H

ATOM 3689 HZ3 TRP 379 70.530 50.490 29.220 1.00 0.00 H

ATOM 3690 HH2 TRP 379 71.570 52.510 30.210 1.00 0.00 H

ATOM 3691 N THR 380 72.740 45.840 24.550 1.00 0.00 N

ATOM 3692 CA THR 380 72.500 45.220 23.220 1.00 0.00 C

ATOM 3693 C THR 380 72.560 46.320 22.130 1.00 0.00 C

ATOM 3694 O THR 380 73.470 46.330 21.310 1.00 0.00 O

ATOM 3695 CB THR 380 71.160 44.460 23.200 1.00 0.00 C

ATOM 3696 CG2 THR 380 71.110 43.570 21.970 1.00 0.00 C

ATOM 3697 OG1 THR 380 70.990 43.670 24.390 1.00 0.00 O

ATOM 3698 H THR 380 72.240 45.380 25.280 1.00 0.00 H

ATOM 3699 HG1 THR 380 70.110 43.150 24.340 1.00 0.00 H

ATOM 3700 N ASP 381 71.590 47.240 22.170 1.00 0.00 N

ATOM 3701 CA ASP 381 71.590 48.480 21.350 1.00 0.00 C

ATOM 3702 C ASP 381 72.390 49.570 22.080 1.00 0.00 C

ATOM 3703 O ASP 381 71.900 50.250 22.980 1.00 0.00 O

ATOM 3704 CB ASP 381 70.130 48.910 21.140 1.00 0.00 C

ATOM 3705 CG ASP 381 69.940 49.930 20.010 1.00 0.00 C

ATOM 3706 OD1 ASP 381 70.530 49.700 18.940 1.00 0.00 O

ATOM 3707 OD2 ASP 381 69.160 50.890 20.200 1.00 0.00 O

ATOM 3708 H ASP 381 70.710 47.050 22.590 1.00 0.00 H

ATOM 3709 N LYS 382 73.680 49.640 21.760 1.00 0.00 N

ATOM 3710 CA LYS 382 74.670 50.460 22.500 1.00 0.00 C

ATOM 3711 C LYS 382 74.330 51.960 22.650 1.00 0.00 C

ATOM 3712 O LYS 382 74.340 52.480 23.770 1.00 0.00 O

ATOM 3713 CB LYS 382 76.030 50.230 21.830 1.00 0.00 C

ATOM 3714 CG LYS 382 77.200 51.050 22.360 1.00 0.00 C

ATOM 3715 CD LYS 382 77.580 50.740 23.810 1.00 0.00 C

ATOM 3716 CE LYS 382 78.930 51.370 24.170 1.00 0.00 C

ATOM 3717 NZ LYS 382 80.010 50.720 23.420 1.00 0.00 N1+

ATOM 3718 H LYS 382 74.050 49.120 21.000 1.00 0.00 H

ATOM 3719 HZ1 LYS 382 80.900 51.090 23.690 1.00 0.00 H

ATOM 3720 HZ2 LYS 382 80.010 49.730 23.600 1.00 0.00 H

ATOM 3721 HZ3 LYS 382 79.910 50.870 22.440 1.00 0.00 H

ATOM 3722 N GLU 383 73.870 52.580 21.580 1.00 0.00 N

ATOM 3723 CA GLU 383 73.750 54.050 21.510 1.00 0.00 C

ATOM 3724 C GLU 383 72.320 54.560 21.760 1.00 0.00 C

ATOM 3725 O GLU 383 71.600 54.980 20.860 1.00 0.00 O

ATOM 3726 CB GLU 383 74.350 54.600 20.210 1.00 0.00 C

ATOM 3727 CG GLU 383 73.730 54.130 18.890 1.00 0.00 C

ATOM 3728 CD GLU 383 73.940 52.650 18.570 1.00 0.00 C

ATOM 3729 OE1 GLU 383 75.020 52.120 18.900 1.00 0.00 O

ATOM 3730 OE2 GLU 383 73.120 52.080 17.830 1.00 0.00 O

ATOM 3731 H GLU 383 73.660 52.100 20.720 1.00 0.00 H

ATOM 3732 N SER 384 71.960 54.600 23.040 1.00 0.00 N

ATOM 3733 CA SER 384 70.630 55.120 23.410 1.00 0.00 C

ATOM 3734 C SER 384 70.370 55.690 24.810 1.00 0.00 C

ATOM 3735 O SER 384 70.520 55.080 25.860 1.00 0.00 O

ATOM 3736 CB SER 384 69.520 54.130 23.040 1.00 0.00 C

ATOM 3737 OG SER 384 69.660 52.900 23.750 1.00 0.00 O

ATOM 3738 H SER 384 72.460 54.070 23.740 1.00 0.00 H

ATOM 3739 HG SER 384 69.810 52.160 23.080 1.00 0.00 H

ATOM 3740 N ARG 385 69.860 56.910 24.720 1.00 0.00 N

ATOM 3741 CA ARG 385 69.210 57.630 25.840 1.00 0.00 C

ATOM 3742 C ARG 385 67.930 56.920 26.330 1.00 0.00 C

ATOM 3743 O ARG 385 67.580 57.030 27.510 1.00 0.00 O

ATOM 3744 CB ARG 385 68.870 59.080 25.460 1.00 0.00 C

ATOM 3745 CG ARG 385 70.050 60.060 25.490 1.00 0.00 C

ATOM 3746 CD ARG 385 70.930 60.050 24.240 1.00 0.00 C

ATOM 3747 NE ARG 385 70.390 60.890 23.160 1.00 0.00 N

ATOM 3748 CZ ARG 385 69.500 60.540 22.210 1.00 0.00 C

ATOM 3749 NH1 ARG 385 68.900 59.340 22.190 1.00 0.00 N

ATOM 3750 NH2 ARG 385 69.120 61.430 21.300 1.00 0.00 N

ATOM 3751 H ARG 385 70.040 57.440 23.900 1.00 0.00 H

ATOM 3752 HE ARG 385 70.750 61.830 23.150 1.00 0.00 H

ATOM 3753 1HH1 ARG 385 69.080 58.680 22.900 1.00 0.00 H

ATOM 3754 2HH1 ARG 385 68.290 59.100 21.430 1.00 0.00 H

ATOM 3755 1HH2 ARG 385 69.430 62.390 21.360 1.00 0.00 H

ATOM 3756 2HH2 ARG 385 68.570 61.140 20.520 1.00 0.00 H

ATOM 3757 N MET 386 67.250 56.220 25.410 1.00 0.00 N

ATOM 3758 CA MET 386 66.100 55.350 25.740 1.00 0.00 C

ATOM 3759 C MET 386 66.500 54.220 26.710 1.00 0.00 C

ATOM 3760 O MET 386 66.030 54.240 27.840 1.00 0.00 O

ATOM 3761 CB MET 386 65.380 54.830 24.490 1.00 0.00 C

ATOM 3762 CG MET 386 66.170 53.820 23.650 1.00 0.00 C

ATOM 3763 SD MET 386 65.350 53.200 22.140 1.00 0.00 S

ATOM 3764 CE MET 386 66.500 51.890 21.770 1.00 0.00 C

ATOM 3765 H MET 386 67.470 56.250 24.440 1.00 0.00 H

ATOM 3766 N LYS 387 67.650 53.590 26.430 1.00 0.00 N

ATOM 3767 CA LYS 387 68.200 52.530 27.280 1.00 0.00 C

ATOM 3768 C LYS 387 68.560 52.970 28.710 1.00 0.00 C

ATOM 3769 O LYS 387 68.240 52.220 29.630 1.00 0.00 O

ATOM 3770 CB LYS 387 69.410 51.860 26.640 1.00 0.00 C

ATOM 3771 CG LYS 387 68.990 50.530 26.000 1.00 0.00 C

ATOM 3772 CD LYS 387 70.220 49.860 25.390 1.00 0.00 C

ATOM 3773 CE LYS 387 70.010 48.380 25.090 1.00 0.00 C

ATOM 3774 NZ LYS 387 69.920 47.590 26.330 1.00 0.00 N1+

ATOM 3775 H LYS 387 68.140 53.720 25.560 1.00 0.00 H

ATOM 3776 HZ1 LYS 387 69.760 46.630 26.130 1.00 0.00 H

ATOM 3777 HZ2 LYS 387 70.760 47.670 26.880 1.00 0.00 H

ATOM 3778 HZ3 LYS 387 69.140 47.920 26.880 1.00 0.00 H

ATOM 3779 N ASN 388 69.170 54.140 28.890 1.00 0.00 N

ATOM 3780 CA ASN 388 69.380 54.700 30.240 1.00 0.00 C

ATOM 3781 C ASN 388 68.070 54.970 31.000 1.00 0.00 C

ATOM 3782 O ASN 388 67.920 54.490 32.120 1.00 0.00 O

ATOM 3783 CB ASN 388 70.230 55.980 30.250 1.00 0.00 C

ATOM 3784 CG ASN 388 71.740 55.720 30.240 1.00 0.00 C

ATOM 3785 ND2 ASN 388 72.460 56.680 29.720 1.00 0.00 N

ATOM 3786 OD1 ASN 388 72.290 54.790 30.810 1.00 0.00 O

ATOM 3787 H ASN 388 69.600 54.630 28.130 1.00 0.00 H

ATOM 3788 1HD2 ASN 388 72.030 57.520 29.420 1.00 0.00 H

ATOM 3789 2HD2 ASN 388 73.420 56.490 29.500 1.00 0.00 H

ATOM 3790 N ARG 389 67.090 55.550 30.310 1.00 0.00 N

ATOM 3791 CA ARG 389 65.730 55.780 30.850 1.00 0.00 C

ATOM 3792 C ARG 389 65.090 54.460 31.310 1.00 0.00 C

ATOM 3793 O ARG 389 64.980 54.230 32.520 1.00 0.00 O

ATOM 3794 CB ARG 389 64.890 56.550 29.830 1.00 0.00 C

ATOM 3795 CG ARG 389 63.390 56.570 30.140 1.00 0.00 C

ATOM 3796 CD ARG 389 62.550 57.500 29.260 1.00 0.00 C

ATOM 3797 NE ARG 389 62.860 57.360 27.820 1.00 0.00 N

ATOM 3798 CZ ARG 389 62.410 56.400 26.990 1.00 0.00 C

ATOM 3799 NH1 ARG 389 61.560 55.460 27.380 1.00 0.00 N

ATOM 3800 NH2 ARG 389 62.810 56.410 25.720 1.00 0.00 N

ATOM 3801 H ARG 389 67.190 55.780 29.340 1.00 0.00 H

ATOM 3802 HE ARG 389 63.410 58.100 27.450 1.00 0.00 H

ATOM 3803 1HH1 ARG 389 61.190 55.470 28.310 1.00 0.00 H

ATOM 3804 2HH1 ARG 389 61.440 54.630 26.840 1.00 0.00 H

ATOM 3805 1HH2 ARG 389 63.410 57.120 25.360 1.00 0.00 H

ATOM 3806 2HH2 ARG 389 62.470 55.710 25.090 1.00 0.00 H

ATOM 3807 N ASP 390 64.920 53.540 30.360 1.00 0.00 N

ATOM 3808 CA ASP 390 64.310 52.220 30.600 1.00 0.00 C

ATOM 3809 C ASP 390 65.040 51.440 31.700 1.00 0.00 C

ATOM 3810 O ASP 390 64.420 51.060 32.690 1.00 0.00 O

ATOM 3811 CB ASP 390 64.260 51.400 29.300 1.00 0.00 C

ATOM 3812 CG ASP 390 63.460 52.080 28.180 1.00 0.00 C

ATOM 3813 OD1 ASP 390 62.450 52.750 28.480 1.00 0.00 O

ATOM 3814 OD2 ASP 390 63.920 51.960 27.030 1.00 0.00 O

ATOM 3815 H ASP 390 65.060 53.740 29.380 1.00 0.00 H

ATOM 3816 N SER 391 66.380 51.500 31.670 1.00 0.00 N

ATOM 3817 CA SER 391 67.250 50.870 32.680 1.00 0.00 C

ATOM 3818 C SER 391 66.960 51.360 34.110 1.00 0.00 C

ATOM 3819 O SER 391 66.680 50.530 34.980 1.00 0.00 O

ATOM 3820 CB SER 391 68.720 51.100 32.330 1.00 0.00 C

ATOM 3821 OG SER 391 69.570 50.520 33.310 1.00 0.00 O

ATOM 3822 H SER 391 66.850 52.000 30.940 1.00 0.00 H

ATOM 3823 HG SER 391 70.310 49.980 32.860 1.00 0.00 H

ATOM 3824 N ILE 392 66.800 52.680 34.270 1.00 0.00 N

ATOM 3825 CA ILE 392 66.410 53.320 35.540 1.00 0.00 C

ATOM 3826 C ILE 392 65.000 52.860 36.000 1.00 0.00 C

ATOM 3827 O ILE 392 64.870 52.420 37.140 1.00 0.00 O

ATOM 3828 CB ILE 392 66.570 54.860 35.410 1.00 0.00 C

ATOM 3829 CG1 ILE 392 68.050 55.280 35.270 1.00 0.00 C

ATOM 3830 CG2 ILE 392 65.930 55.670 36.540 1.00 0.00 C

ATOM 3831 CD ILE 392 68.950 54.970 36.480 1.00 0.00 C

ATOM 3832 H ILE 392 66.910 53.310 33.490 1.00 0.00 H

ATOM 3833 N GLY 393 64.040 52.810 35.070 1.00 0.00 N

ATOM 3834 CA GLY 393 62.670 52.310 35.330 1.00 0.00 C

ATOM 3835 C GLY 393 62.640 50.850 35.800 1.00 0.00 C

ATOM 3836 O GLY 393 62.120 50.540 36.880 1.00 0.00 O

ATOM 3837 H GLY 393 64.200 53.130 34.130 1.00 0.00 H

ATOM 3838 N GLN 394 63.370 50.020 35.060 1.00 0.00 N

ATOM 3839 CA GLN 394 63.580 48.590 35.380 1.00 0.00 C

ATOM 3840 C GLN 394 64.190 48.420 36.780 1.00 0.00 C

ATOM 3841 O GLN 394 63.560 47.830 37.640 1.00 0.00 O

ATOM 3842 CB GLN 394 64.550 47.920 34.390 1.00 0.00 C

ATOM 3843 CG GLN 394 64.250 48.110 32.900 1.00 0.00 C

ATOM 3844 CD GLN 394 65.420 47.660 32.000 1.00 0.00 C

ATOM 3845 NE2 GLN 394 66.630 47.710 32.510 1.00 0.00 N

ATOM 3846 OE1 GLN 394 65.250 47.280 30.850 1.00 0.00 O

ATOM 3847 H GLN 394 63.730 50.320 34.170 1.00 0.00 H

ATOM 3848 1HE2 GLN 394 66.770 48.000 33.450 1.00 0.00 H

ATOM 3849 2HE2 GLN 394 67.430 47.600 31.880 1.00 0.00 H

ATOM 3850 N LEU 395 65.250 49.190 37.050 1.00 0.00 N

ATOM 3851 CA LEU 395 65.990 49.200 38.330 1.00 0.00 C

ATOM 3852 C LEU 395 65.050 49.340 39.540 1.00 0.00 C

ATOM 3853 O LEU 395 64.950 48.450 40.380 1.00 0.00 O

ATOM 3854 CB LEU 395 66.980 50.380 38.300 1.00 0.00 C

ATOM 3855 CG LEU 395 67.860 50.480 39.550 1.00 0.00 C

ATOM 3856 CD1 LEU 395 68.930 49.410 39.510 1.00 0.00 C

ATOM 3857 CD2 LEU 395 68.500 51.860 39.670 1.00 0.00 C

ATOM 3858 H LEU 395 65.630 49.810 36.350 1.00 0.00 H

ATOM 3859 N VAL 396 64.240 50.390 39.460 1.00 0.00 N

ATOM 3860 CA VAL 396 63.260 50.780 40.500 1.00 0.00 C

ATOM 3861 C VAL 396 62.210 49.670 40.690 1.00 0.00 C

ATOM 3862 O VAL 396 62.180 49.040 41.750 1.00 0.00 O

ATOM 3863 CB VAL 396 62.650 52.140 40.110 1.00 0.00 C

ATOM 3864 CG1 VAL 396 61.680 52.670 41.160 1.00 0.00 C

ATOM 3865 CG2 VAL 396 63.750 53.190 39.930 1.00 0.00 C

ATOM 3866 H VAL 396 64.270 51.000 38.670 1.00 0.00 H

ATOM 3867 N GLY 397 61.580 49.280 39.590 1.00 0.00 N

ATOM 3868 CA GLY 397 60.540 48.230 39.560 1.00 0.00 C

ATOM 3869 C GLY 397 61.020 46.860 40.070 1.00 0.00 C

ATOM 3870 O GLY 397 60.420 46.300 40.990 1.00 0.00 O

ATOM 3871 H GLY 397 61.780 49.700 38.690 1.00 0.00 H

ATOM 3872 N ASP 398 62.180 46.440 39.560 1.00 0.00 N

ATOM 3873 CA ASP 398 62.800 45.150 39.900 1.00 0.00 C

ATOM 3874 C ASP 398 63.170 45.070 41.390 1.00 0.00 C

ATOM 3875 O ASP 398 62.650 44.180 42.080 1.00 0.00 O

ATOM 3876 CB ASP 398 64.030 44.860 39.000 1.00 0.00 C

ATOM 3877 CG ASP 398 63.820 44.850 37.470 1.00 0.00 C

ATOM 3878 OD1 ASP 398 62.670 44.840 36.990 1.00 0.00 O

ATOM 3879 OD2 ASP 398 64.840 44.830 36.740 1.00 0.00 O

ATOM 3880 H ASP 398 62.650 46.940 38.820 1.00 0.00 H

ATOM 3881 N GLN 399 63.860 46.090 41.920 1.00 0.00 N

ATOM 3882 CA GLN 399 64.250 46.180 43.340 1.00 0.00 C

ATOM 3883 C GLN 399 63.060 46.310 44.310 1.00 0.00 C

ATOM 3884 O GLN 399 63.030 45.660 45.350 1.00 0.00 O

ATOM 3885 CB GLN 399 65.220 47.360 43.600 1.00 0.00 C

ATOM 3886 CG GLN 399 65.780 47.350 45.030 1.00 0.00 C

ATOM 3887 CD GLN 399 66.660 48.550 45.400 1.00 0.00 C

ATOM 3888 NE2 GLN 399 66.640 48.920 46.670 1.00 0.00 N

ATOM 3889 OE1 GLN 399 67.390 49.140 44.620 1.00 0.00 O

ATOM 3890 H GLN 399 64.190 46.850 41.340 1.00 0.00 H

ATOM 3891 1HE2 GLN 399 66.070 48.430 47.330 1.00 0.00 H

ATOM 3892 2HE2 GLN 399 67.170 49.730 46.910 1.00 0.00 H

ATOM 3893 N MET 400 62.200 47.290 44.030 1.00 0.00 N

ATOM 3894 CA MET 400 61.080 47.660 44.920 1.00 0.00 C

ATOM 3895 C MET 400 59.920 46.660 44.940 1.00 0.00 C

ATOM 3896 O MET 400 59.460 46.250 46.010 1.00 0.00 O

ATOM 3897 CB MET 400 60.550 49.040 44.520 1.00 0.00 C

ATOM 3898 CG MET 400 61.580 50.160 44.650 1.00 0.00 C

ATOM 3899 SD MET 400 61.870 50.720 46.370 1.00 0.00 S

ATOM 3900 CE MET 400 63.430 49.920 46.670 1.00 0.00 C

ATOM 3901 H MET 400 62.300 47.870 43.220 1.00 0.00 H

ATOM 3902 N PHE 401 59.510 46.230 43.750 1.00 0.00 N

ATOM 3903 CA PHE 401 58.270 45.440 43.590 1.00 0.00 C

ATOM 3904 C PHE 401 58.510 44.000 43.100 1.00 0.00 C

ATOM 3905 O PHE 401 58.440 43.070 43.910 1.00 0.00 O

ATOM 3906 CB PHE 401 57.310 46.190 42.650 1.00 0.00 C

ATOM 3907 CG PHE 401 56.950 47.570 43.190 1.00 0.00 C

ATOM 3908 CD1 PHE 401 57.650 48.690 42.750 1.00 0.00 C

ATOM 3909 CD2 PHE 401 56.040 47.690 44.220 1.00 0.00 C

ATOM 3910 CE1 PHE 401 57.440 49.920 43.350 1.00 0.00 C

ATOM 3911 CE2 PHE 401 55.820 48.920 44.820 1.00 0.00 C

ATOM 3912 CZ PHE 401 56.510 50.040 44.390 1.00 0.00 C

ATOM 3913 H PHE 401 59.950 46.520 42.900 1.00 0.00 H

ATOM 3914 HD1 PHE 401 58.370 48.600 41.930 1.00 0.00 H

ATOM 3915 HD2 PHE 401 55.440 46.830 44.500 1.00 0.00 H

ATOM 3916 HE1 PHE 401 58.010 50.790 43.040 1.00 0.00 H

ATOM 3917 HE2 PHE 401 55.020 49.020 45.560 1.00 0.00 H

ATOM 3918 HZ PHE 401 56.240 51.020 44.770 1.00 0.00 H

ATOM 3919 N VAL 402 59.000 43.870 41.870 1.00 0.00 N

ATOM 3920 CA VAL 402 59.040 42.590 41.130 1.00 0.00 C

ATOM 3921 C VAL 402 59.800 41.490 41.890 1.00 0.00 C

ATOM 3922 O VAL 402 59.180 40.530 42.360 1.00 0.00 O

ATOM 3923 CB VAL 402 59.590 42.760 39.700 1.00 0.00 C

ATOM 3924 CG1 VAL 402 59.430 41.460 38.910 1.00 0.00 C

ATOM 3925 CG2 VAL 402 58.900 43.900 38.940 1.00 0.00 C

ATOM 3926 H VAL 402 59.360 44.670 41.370 1.00 0.00 H

ATOM 3927 N CYS 403 61.100 41.670 42.060 1.00 0.00 N

ATOM 3928 CA CYS 403 61.970 40.660 42.690 1.00 0.00 C

ATOM 3929 C CYS 403 61.590 40.290 44.150 1.00 0.00 C

ATOM 3930 O CYS 403 61.480 39.090 44.400 1.00 0.00 O

ATOM 3931 CB CYS 403 63.440 41.030 42.530 1.00 0.00 C

ATOM 3932 SG CYS 403 63.910 41.130 40.760 1.00 0.00 S

ATOM 3933 H CYS 403 61.540 42.560 41.880 1.00 0.00 H

ATOM 3934 N PRO 404 61.170 41.250 44.990 1.00 0.00 N

ATOM 3935 CA PRO 404 60.560 40.940 46.300 1.00 0.00 C

ATOM 3936 C PRO 404 59.330 40.040 46.180 1.00 0.00 C

ATOM 3937 O PRO 404 59.250 39.030 46.880 1.00 0.00 O

ATOM 3938 CB PRO 404 60.160 42.290 46.890 1.00 0.00 C

ATOM 3939 CG PRO 404 61.220 43.230 46.340 1.00 0.00 C

ATOM 3940 CD PRO 404 61.470 42.700 44.930 1.00 0.00 C

ATOM 3941 N VAL 405 58.430 40.350 45.240 1.00 0.00 N

ATOM 3942 CA VAL 405 57.200 39.560 45.000 1.00 0.00 C

ATOM 3943 C VAL 405 57.540 38.120 44.570 1.00 0.00 C

ATOM 3944 O VAL 405 57.090 37.170 45.220 1.00 0.00 O

ATOM 3945 CB VAL 405 56.300 40.250 43.960 1.00 0.00 C

ATOM 3946 CG1 VAL 405 55.050 39.420 43.630 1.00 0.00 C

ATOM 3947 CG2 VAL 405 55.810 41.600 44.480 1.00 0.00 C

ATOM 3948 H VAL 405 58.530 41.160 44.660 1.00 0.00 H

ATOM 3949 N VAL 406 58.410 38.000 43.570 1.00 0.00 N

ATOM 3950 CA VAL 406 58.890 36.690 43.060 1.00 0.00 C

ATOM 3951 C VAL 406 59.450 35.850 44.230 1.00 0.00 C

ATOM 3952 O VAL 406 58.960 34.740 44.480 1.00 0.00 O

ATOM 3953 CB VAL 406 59.940 36.860 41.940 1.00 0.00 C

ATOM 3954 CG1 VAL 406 60.400 35.520 41.360 1.00 0.00 C

ATOM 3955 CG2 VAL 406 59.390 37.710 40.780 1.00 0.00 C

ATOM 3956 H VAL 406 58.720 38.800 43.050 1.00 0.00 H

ATOM 3957 N ASP 407 60.310 36.450 45.040 1.00 0.00 N

ATOM 3958 CA ASP 407 60.880 35.770 46.220 1.00 0.00 C

ATOM 3959 C ASP 407 59.820 35.350 47.260 1.00 0.00 C

ATOM 3960 O ASP 407 59.730 34.170 47.580 1.00 0.00 O

ATOM 3961 CB ASP 407 61.980 36.620 46.860 1.00 0.00 C

ATOM 3962 CG ASP 407 62.690 35.880 47.990 1.00 0.00 C

ATOM 3963 OD1 ASP 407 62.620 34.630 48.060 1.00 0.00 O

ATOM 3964 OD2 ASP 407 63.310 36.570 48.810 1.00 0.00 O

ATOM 3965 H ASP 407 60.630 37.390 44.890 1.00 0.00 H

ATOM 3966 N PHE 408 58.890 36.260 47.570 1.00 0.00 N

ATOM 3967 CA PHE 408 57.770 35.970 48.480 1.00 0.00 C

ATOM 3968 C PHE 408 56.940 34.770 47.980 1.00 0.00 C

ATOM 3969 O PHE 408 56.840 33.770 48.700 1.00 0.00 O

ATOM 3970 CB PHE 408 56.850 37.190 48.650 1.00 0.00 C

ATOM 3971 CG PHE 408 55.830 36.960 49.770 1.00 0.00 C

ATOM 3972 CD1 PHE 408 56.170 37.270 51.080 1.00 0.00 C

ATOM 3973 CD2 PHE 408 54.630 36.310 49.510 1.00 0.00 C

ATOM 3974 CE1 PHE 408 55.320 36.930 52.120 1.00 0.00 C

ATOM 3975 CE2 PHE 408 53.790 35.940 50.560 1.00 0.00 C

ATOM 3976 CZ PHE 408 54.140 36.240 51.870 1.00 0.00 C

ATOM 3977 H PHE 408 58.910 37.190 47.190 1.00 0.00 H

ATOM 3978 HD1 PHE 408 57.150 37.690 51.310 1.00 0.00 H

ATOM 3979 HD2 PHE 408 54.320 36.130 48.480 1.00 0.00 H

ATOM 3980 HE1 PHE 408 55.550 37.250 53.140 1.00 0.00 H

ATOM 3981 HE2 PHE 408 52.860 35.410 50.350 1.00 0.00 H

ATOM 3982 HZ PHE 408 53.510 35.910 52.700 1.00 0.00 H

ATOM 3983 N ALA 409 56.580 34.800 46.700 1.00 0.00 N

ATOM 3984 CA ALA 409 55.850 33.720 46.010 1.00 0.00 C

ATOM 3985 C ALA 409 56.590 32.370 46.110 1.00 0.00 C

ATOM 3986 O ALA 409 56.060 31.440 46.710 1.00 0.00 O

ATOM 3987 CB ALA 409 55.610 34.100 44.550 1.00 0.00 C

ATOM 3988 H ALA 409 56.770 35.610 46.130 1.00 0.00 H

ATOM 3989 N ASN 410 57.880 32.370 45.780 1.00 0.00 N

ATOM 3990 CA ASN 410 58.760 31.190 45.900 1.00 0.00 C

ATOM 3991 C ASN 410 58.820 30.620 47.330 1.00 0.00 C

ATOM 3992 O ASN 410 58.490 29.460 47.550 1.00 0.00 O

ATOM 3993 CB ASN 410 60.180 31.500 45.410 1.00 0.00 C

ATOM 3994 CG ASN 410 60.290 31.480 43.890 1.00 0.00 C

ATOM 3995 ND2 ASN 410 60.570 32.610 43.290 1.00 0.00 N

ATOM 3996 OD1 ASN 410 60.150 30.460 43.240 1.00 0.00 O

ATOM 3997 H ASN 410 58.320 33.190 45.390 1.00 0.00 H

ATOM 3998 1HD2 ASN 410 60.660 33.460 43.810 1.00 0.00 H

ATOM 3999 2HD2 ASN 410 60.700 32.580 42.300 1.00 0.00 H

ATOM 4000 N LYS 411 59.110 31.500 48.290 1.00 0.00 N

ATOM 4001 CA LYS 411 59.170 31.130 49.720 1.00 0.00 C

ATOM 4002 C LYS 411 57.840 30.580 50.280 1.00 0.00 C

ATOM 4003 O LYS 411 57.820 29.530 50.910 1.00 0.00 O

ATOM 4004 CB LYS 411 59.670 32.290 50.580 1.00 0.00 C

ATOM 4005 CG LYS 411 61.180 32.520 50.440 1.00 0.00 C

ATOM 4006 CD LYS 411 61.960 31.230 50.710 1.00 0.00 C

ATOM 4007 CE LYS 411 63.460 31.450 50.790 1.00 0.00 C

ATOM 4008 NZ LYS 411 64.190 30.170 50.730 1.00 0.00 N1+

ATOM 4009 H LYS 411 59.330 32.460 48.080 1.00 0.00 H

ATOM 4010 HZ1 LYS 411 65.180 30.380 50.750 1.00 0.00 H

ATOM 4011 HZ2 LYS 411 63.940 29.590 51.500 1.00 0.00 H

ATOM 4012 HZ3 LYS 411 63.980 29.710 49.870 1.00 0.00 H

ATOM 4013 N TYR 412 56.760 31.260 49.920 1.00 0.00 N

ATOM 4014 CA TYR 412 55.390 30.880 50.300 1.00 0.00 C

ATOM 4015 C TYR 412 54.960 29.530 49.690 1.00 0.00 C

ATOM 4016 O TYR 412 54.400 28.680 50.380 1.00 0.00 O

ATOM 4017 CB TYR 412 54.450 32.010 49.850 1.00 0.00 C

ATOM 4018 CG TYR 412 53.050 31.840 50.430 1.00 0.00 C

ATOM 4019 CD1 TYR 412 52.750 32.420 51.650 1.00 0.00 C

ATOM 4020 CD2 TYR 412 52.130 31.020 49.780 1.00 0.00 C

ATOM 4021 CE1 TYR 412 51.510 32.180 52.230 1.00 0.00 C

ATOM 4022 CE2 TYR 412 50.900 30.780 50.360 1.00 0.00 C

ATOM 4023 CZ TYR 412 50.590 31.370 51.590 1.00 0.00 C

ATOM 4024 OH TYR 412 49.390 31.130 52.150 1.00 0.00 O

ATOM 4025 H TYR 412 56.820 32.120 49.400 1.00 0.00 H

ATOM 4026 HD1 TYR 412 53.480 33.050 52.150 1.00 0.00 H

ATOM 4027 HD2 TYR 412 52.370 30.580 48.820 1.00 0.00 H

ATOM 4028 HE1 TYR 412 51.270 32.620 53.210 1.00 0.00 H

ATOM 4029 HE2 TYR 412 50.190 30.140 49.850 1.00 0.00 H

ATOM 4030 HH TYR 412 48.820 30.650 51.460 1.00 0.00 H

ATOM 4031 N SER 413 55.250 29.360 48.400 1.00 0.00 N

ATOM 4032 CA SER 413 54.960 28.120 47.650 1.00 0.00 C

ATOM 4033 C SER 413 55.660 26.870 48.210 1.00 0.00 C

ATOM 4034 O SER 413 55.030 25.820 48.300 1.00 0.00 O

ATOM 4035 CB SER 413 55.310 28.280 46.170 1.00 0.00 C

ATOM 4036 OG SER 413 54.410 29.210 45.570 1.00 0.00 O

ATOM 4037 H SER 413 55.590 30.120 47.830 1.00 0.00 H

ATOM 4038 HG SER 413 54.610 29.270 44.590 1.00 0.00 H

ATOM 4039 N GLN 414 56.880 27.040 48.710 1.00 0.00 N

ATOM 4040 CA GLN 414 57.660 25.950 49.360 1.00 0.00 C

ATOM 4041 C GLN 414 56.950 25.270 50.540 1.00 0.00 C

ATOM 4042 O GLN 414 57.120 24.080 50.800 1.00 0.00 O

ATOM 4043 CB GLN 414 59.030 26.470 49.810 1.00 0.00 C

ATOM 4044 CG GLN 414 59.910 26.690 48.580 1.00 0.00 C

ATOM 4045 CD GLN 414 61.290 27.270 48.900 1.00 0.00 C

ATOM 4046 NE2 GLN 414 61.790 28.020 47.950 1.00 0.00 N

ATOM 4047 OE1 GLN 414 61.900 27.070 49.940 1.00 0.00 O

ATOM 4048 H GLN 414 57.390 27.900 48.600 1.00 0.00 H

ATOM 4049 1HE2 GLN 414 61.260 28.170 47.120 1.00 0.00 H

ATOM 4050 2HE2 GLN 414 62.730 28.360 48.030 1.00 0.00 H

ATOM 4051 N ASN 415 56.090 26.040 51.200 1.00 0.00 N

ATOM 4052 CA ASN 415 55.200 25.540 52.270 1.00 0.00 C

ATOM 4053 C ASN 415 54.170 24.480 51.820 1.00 0.00 C

ATOM 4054 O ASN 415 53.520 23.890 52.680 1.00 0.00 O

ATOM 4055 CB ASN 415 54.480 26.700 52.950 1.00 0.00 C

ATOM 4056 CG ASN 415 55.440 27.700 53.610 1.00 0.00 C

ATOM 4057 ND2 ASN 415 55.030 28.940 53.640 1.00 0.00 N

ATOM 4058 OD1 ASN 415 56.500 27.370 54.110 1.00 0.00 O

ATOM 4059 H ASN 415 56.060 27.030 51.050 1.00 0.00 H

ATOM 4060 1HD2 ASN 415 54.150 29.190 53.240 1.00 0.00 H

ATOM 4061 2HD2 ASN 415 55.640 29.620 54.030 1.00 0.00 H

ATOM 4062 N GLY 416 53.980 24.310 50.510 1.00 0.00 N

ATOM 4063 CA GLY 416 53.080 23.270 49.960 1.00 0.00 C

ATOM 4064 C GLY 416 51.830 23.810 49.240 1.00 0.00 C

ATOM 4065 O GLY 416 51.240 23.110 48.420 1.00 0.00 O

ATOM 4066 H GLY 416 54.490 24.830 49.820 1.00 0.00 H

ATOM 4067 N GLY 417 51.440 25.020 49.620 1.00 0.00 N

ATOM 4068 CA GLY 417 50.270 25.740 49.100 1.00 0.00 C

ATOM 4069 C GLY 417 50.320 26.160 47.620 1.00 0.00 C

ATOM 4070 O GLY 417 51.390 26.360 47.030 1.00 0.00 O

ATOM 4071 H GLY 417 51.900 25.500 50.370 1.00 0.00 H

ATOM 4072 N LYS 418 49.120 26.370 47.110 1.00 0.00 N

ATOM 4073 CA LYS 418 48.900 26.770 45.700 1.00 0.00 C

ATOM 4074 C LYS 418 49.020 28.290 45.530 1.00 0.00 C

ATOM 4075 O LYS 418 48.310 29.070 46.150 1.00 0.00 O

ATOM 4076 CB LYS 418 47.540 26.290 45.170 1.00 0.00 C

ATOM 4077 CG LYS 418 47.260 24.790 45.370 1.00 0.00 C

ATOM 4078 CD LYS 418 48.210 23.840 44.630 1.00 0.00 C

ATOM 4079 CE LYS 418 47.890 23.720 43.140 1.00 0.00 C

ATOM 4080 NZ LYS 418 48.710 22.680 42.520 1.00 0.00 N1+

ATOM 4081 H LYS 418 48.300 26.360 47.700 1.00 0.00 H

ATOM 4082 HZ1 LYS 418 48.470 22.590 41.550 1.00 0.00 H

ATOM 4083 HZ2 LYS 418 49.680 22.900 42.590 1.00 0.00 H

ATOM 4084 HZ3 LYS 418 48.530 21.800 42.960 1.00 0.00 H

ATOM 4085 N THR 419 49.980 28.670 44.700 1.00 0.00 N

ATOM 4086 CA THR 419 50.220 30.090 44.350 1.00 0.00 C

ATOM 4087 C THR 419 50.010 30.270 42.840 1.00 0.00 C

ATOM 4088 O THR 419 50.350 29.390 42.050 1.00 0.00 O

ATOM 4089 CB THR 419 51.650 30.530 44.740 1.00 0.00 C

ATOM 4090 CG2 THR 419 51.880 32.040 44.540 1.00 0.00 C

ATOM 4091 OG1 THR 419 51.920 30.210 46.100 1.00 0.00 O

ATOM 4092 H THR 419 50.620 28.010 44.300 1.00 0.00 H

ATOM 4093 HG1 THR 419 52.900 29.960 46.170 1.00 0.00 H

ATOM 4094 N PHE 420 49.410 31.390 42.480 1.00 0.00 N

ATOM 4095 CA PHE 420 49.270 31.820 41.080 1.00 0.00 C

ATOM 4096 C PHE 420 49.890 33.210 40.900 1.00 0.00 C

ATOM 4097 O PHE 420 49.680 34.110 41.720 1.00 0.00 O

ATOM 4098 CB PHE 420 47.800 31.850 40.650 1.00 0.00 C

ATOM 4099 CG PHE 420 47.140 30.480 40.820 1.00 0.00 C

ATOM 4100 CD1 PHE 420 47.250 29.500 39.830 1.00 0.00 C

ATOM 4101 CD2 PHE 420 46.480 30.200 42.010 1.00 0.00 C

ATOM 4102 CE1 PHE 420 46.690 28.250 40.040 1.00 0.00 C

ATOM 4103 CE2 PHE 420 45.920 28.940 42.220 1.00 0.00 C

ATOM 4104 CZ PHE 420 46.020 27.970 41.230 1.00 0.00 C

ATOM 4105 H PHE 420 48.950 31.970 43.160 1.00 0.00 H

ATOM 4106 HD1 PHE 420 47.790 29.710 38.910 1.00 0.00 H

ATOM 4107 HD2 PHE 420 46.410 30.970 42.780 1.00 0.00 H

ATOM 4108 HE1 PHE 420 46.780 27.480 39.270 1.00 0.00 H

ATOM 4109 HE2 PHE 420 45.400 28.740 43.150 1.00 0.00 H

ATOM 4110 HZ PHE 420 45.560 27.000 41.380 1.00 0.00 H

ATOM 4111 N PHE 421 50.730 33.310 39.880 1.00 0.00 N

ATOM 4112 CA PHE 421 51.460 34.540 39.560 1.00 0.00 C

ATOM 4113 C PHE 421 51.080 35.060 38.170 1.00 0.00 C

ATOM 4114 O PHE 421 51.010 34.300 37.200 1.00 0.00 O

ATOM 4115 CB PHE 421 52.960 34.260 39.620 1.00 0.00 C

ATOM 4116 CG PHE 421 53.770 35.550 39.800 1.00 0.00 C

ATOM 4117 CD1 PHE 421 54.000 36.410 38.730 1.00 0.00 C

ATOM 4118 CD2 PHE 421 54.370 35.790 41.030 1.00 0.00 C

ATOM 4119 CE1 PHE 421 54.820 37.520 38.910 1.00 0.00 C

ATOM 4120 CE2 PHE 421 55.200 36.880 41.200 1.00 0.00 C

ATOM 4121 CZ PHE 421 55.430 37.750 40.130 1.00 0.00 C

ATOM 4122 H PHE 421 50.950 32.500 39.310 1.00 0.00 H

ATOM 4123 HD1 PHE 421 53.570 36.210 37.750 1.00 0.00 H

ATOM 4124 HD2 PHE 421 54.180 35.110 41.860 1.00 0.00 H

ATOM 4125 HE1 PHE 421 54.940 38.240 38.100 1.00 0.00 H

ATOM 4126 HE2 PHE 421 55.660 37.070 42.160 1.00 0.00 H

ATOM 4127 HZ PHE 421 56.110 38.580 40.250 1.00 0.00 H

ATOM 4128 N TYR 422 50.850 36.370 38.110 1.00 0.00 N

ATOM 4129 CA TYR 422 50.580 37.050 36.830 1.00 0.00 C

ATOM 4130 C TYR 422 51.510 38.250 36.580 1.00 0.00 C

ATOM 4131 O TYR 422 52.120 38.820 37.480 1.00 0.00 O

ATOM 4132 CB TYR 422 49.100 37.450 36.700 1.00 0.00 C

ATOM 4133 CG TYR 422 48.720 38.720 37.460 1.00 0.00 C

ATOM 4134 CD1 TYR 422 48.570 38.690 38.840 1.00 0.00 C

ATOM 4135 CD2 TYR 422 48.580 39.930 36.780 1.00 0.00 C

ATOM 4136 CE1 TYR 422 48.330 39.870 39.530 1.00 0.00 C

ATOM 4137 CE2 TYR 422 48.280 41.090 37.470 1.00 0.00 C

ATOM 4138 CZ TYR 422 48.150 41.070 38.850 1.00 0.00 C

ATOM 4139 OH TYR 422 47.650 42.130 39.520 1.00 0.00 O

ATOM 4140 H TYR 422 50.880 36.950 38.930 1.00 0.00 H

ATOM 4141 HD1 TYR 422 48.630 37.750 39.380 1.00 0.00 H

ATOM 4142 HD2 TYR 422 48.740 39.960 35.700 1.00 0.00 H

ATOM 4143 HE1 TYR 422 48.540 39.900 40.590 1.00 0.00 H

ATOM 4144 HE2 TYR 422 48.060 42.010 36.920 1.00 0.00 H

ATOM 4145 HH TYR 422 48.250 42.940 39.370 1.00 0.00 H

ATOM 4146 N PHE 423 51.660 38.510 35.290 1.00 0.00 N

ATOM 4147 CA PHE 423 52.400 39.650 34.750 1.00 0.00 C

ATOM 4148 C PHE 423 51.460 40.490 33.880 1.00 0.00 C

ATOM 4149 O PHE 423 50.980 40.040 32.850 1.00 0.00 O

ATOM 4150 CB PHE 423 53.590 39.100 33.950 1.00 0.00 C

ATOM 4151 CG PHE 423 54.440 40.220 33.350 1.00 0.00 C

ATOM 4152 CD1 PHE 423 55.200 41.030 34.180 1.00 0.00 C

ATOM 4153 CD2 PHE 423 54.400 40.450 31.980 1.00 0.00 C

ATOM 4154 CE1 PHE 423 55.930 42.080 33.640 1.00 0.00 C

ATOM 4155 CE2 PHE 423 55.170 41.470 31.430 1.00 0.00 C

ATOM 4156 CZ PHE 423 55.950 42.260 32.260 1.00 0.00 C

ATOM 4157 H PHE 423 51.290 37.880 34.600 1.00 0.00 H

ATOM 4158 HD1 PHE 423 55.200 40.860 35.250 1.00 0.00 H

ATOM 4159 HD2 PHE 423 53.640 39.950 31.370 1.00 0.00 H

ATOM 4160 HE1 PHE 423 56.190 42.930 34.270 1.00 0.00 H

ATOM 4161 HE2 PHE 423 55.030 41.740 30.390 1.00 0.00 H

ATOM 4162 HZ PHE 423 56.620 43.010 31.830 1.00 0.00 H

ATOM 4163 N PHE 424 51.060 41.630 34.440 1.00 0.00 N

ATOM 4164 CA PHE 424 50.120 42.560 33.790 1.00 0.00 C

ATOM 4165 C PHE 424 50.860 43.500 32.830 1.00 0.00 C

ATOM 4166 O PHE 424 51.690 44.330 33.230 1.00 0.00 O

ATOM 4167 CB PHE 424 49.340 43.320 34.860 1.00 0.00 C

ATOM 4168 CG PHE 424 48.220 44.160 34.250 1.00 0.00 C

ATOM 4169 CD1 PHE 424 47.020 43.570 33.880 1.00 0.00 C

ATOM 4170 CD2 PHE 424 48.420 45.520 34.060 1.00 0.00 C

ATOM 4171 CE1 PHE 424 46.020 44.350 33.310 1.00 0.00 C

ATOM 4172 CE2 PHE 424 47.430 46.290 33.480 1.00 0.00 C

ATOM 4173 CZ PHE 424 46.230 45.710 33.100 1.00 0.00 C

ATOM 4174 H PHE 424 51.460 41.960 35.310 1.00 0.00 H

ATOM 4175 HD1 PHE 424 46.860 42.510 34.050 1.00 0.00 H

ATOM 4176 HD2 PHE 424 49.320 45.990 34.450 1.00 0.00 H

ATOM 4177 HE1 PHE 424 45.060 43.900 33.080 1.00 0.00 H

ATOM 4178 HE2 PHE 424 47.580 47.360 33.330 1.00 0.00 H

ATOM 4179 HZ PHE 424 45.460 46.320 32.630 1.00 0.00 H

ATOM 4180 N ASP 425 50.650 43.230 31.550 1.00 0.00 N

ATOM 4181 CA ASP 425 51.360 43.930 30.470 1.00 0.00 C

ATOM 4182 C ASP 425 50.490 44.780 29.520 1.00 0.00 C

ATOM 4183 O ASP 425 50.970 45.250 28.480 1.00 0.00 O

ATOM 4184 CB ASP 425 52.230 42.900 29.740 1.00 0.00 C

ATOM 4185 CG ASP 425 51.470 41.830 28.950 1.00 0.00 C

ATOM 4186 OD1 ASP 425 51.090 40.780 29.500 1.00 0.00 O

ATOM 4187 OD2 ASP 425 51.470 41.980 27.710 1.00 0.00 O

ATOM 4188 H ASP 425 50.110 42.430 31.260 1.00 0.00 H

ATOM 4189 N HIS 426 49.300 45.160 29.980 1.00 0.00 N

ATOM 4190 CA HIS 426 48.430 46.080 29.220 1.00 0.00 C

ATOM 4191 C HIS 426 48.470 47.490 29.810 1.00 0.00 C

ATOM 4192 O HIS 426 48.330 47.680 31.020 1.00 0.00 O

ATOM 4193 CB HIS 426 46.990 45.560 29.180 1.00 0.00 C

ATOM 4194 CG HIS 426 46.080 46.450 28.310 1.00 0.00 C

ATOM 4195 CD2 HIS 426 45.210 47.350 28.750 1.00 0.00 C

ATOM 4196 ND1 HIS 426 46.120 46.510 26.980 1.00 0.00 N

ATOM 4197 CE1 HIS 426 45.260 47.470 26.620 1.00 0.00 C

ATOM 4198 NE2 HIS 426 44.700 47.980 27.710 1.00 0.00 N

ATOM 4199 H HIS 426 48.930 44.860 30.860 1.00 0.00 H

ATOM 4200 HE2 HIS 426 44.030 48.740 27.730 1.00 0.00 H

ATOM 4201 N ARG 427 48.610 48.460 28.910 1.00 0.00 N

ATOM 4202 CA ARG 427 48.550 49.890 29.230 1.00 0.00 C

ATOM 4203 C ARG 427 47.190 50.410 28.740 1.00 0.00 C

ATOM 4204 O ARG 427 46.850 50.270 27.560 1.00 0.00 O

ATOM 4205 CB ARG 427 49.700 50.590 28.500 1.00 0.00 C

ATOM 4206 CG ARG 427 49.840 52.050 28.920 1.00 0.00 C

ATOM 4207 CD ARG 427 50.950 52.710 28.130 1.00 0.00 C

ATOM 4208 NE ARG 427 51.060 54.110 28.570 1.00 0.00 N

ATOM 4209 CZ ARG 427 50.760 55.180 27.820 1.00 0.00 C

ATOM 4210 NH1 ARG 427 50.450 55.070 26.530 1.00 0.00 N

ATOM 4211 NH2 ARG 427 50.790 56.390 28.340 1.00 0.00 N

ATOM 4212 H ARG 427 48.740 48.250 27.930 1.00 0.00 H

ATOM 4213 HE ARG 427 51.450 54.220 29.490 1.00 0.00 H

ATOM 4214 1HH1 ARG 427 50.570 54.200 26.050 1.00 0.00 H

ATOM 4215 2HH1 ARG 427 50.130 55.850 25.990 1.00 0.00 H

ATOM 4216 1HH2 ARG 427 51.080 56.550 29.280 1.00 0.00 H

ATOM 4217 2HH2 ARG 427 50.490 57.170 27.800 1.00 0.00 H

ATOM 4218 N SER 428 46.410 50.920 29.680 1.00 0.00 N

ATOM 4219 CA SER 428 45.070 51.490 29.420 1.00 0.00 C

ATOM 4220 C SER 428 45.140 52.580 28.340 1.00 0.00 C

ATOM 4221 O SER 428 45.970 53.490 28.390 1.00 0.00 O

ATOM 4222 CB SER 428 44.400 52.060 30.680 1.00 0.00 C

ATOM 4223 OG SER 428 44.990 53.290 31.130 1.00 0.00 O

ATOM 4224 H SER 428 46.630 50.790 30.660 1.00 0.00 H

ATOM 4225 HG SER 428 44.410 53.710 31.860 1.00 0.00 H

ATOM 4226 N SER 429 44.240 52.460 27.380 1.00 0.00 N

ATOM 4227 CA SER 429 44.060 53.430 26.290 1.00 0.00 C

ATOM 4228 C SER 429 43.860 54.870 26.810 1.00 0.00 C

ATOM 4229 O SER 429 44.370 55.830 26.240 1.00 0.00 O

ATOM 4230 CB SER 429 42.860 53.040 25.410 1.00 0.00 C

ATOM 4231 OG SER 429 41.650 53.000 26.180 1.00 0.00 O

ATOM 4232 H SER 429 43.660 51.620 27.330 1.00 0.00 H

ATOM 4233 HG SER 429 41.410 52.010 26.410 1.00 0.00 H

ATOM 4234 N LEU 430 43.290 54.950 28.010 1.00 0.00 N

ATOM 4235 CA LEU 430 42.990 56.210 28.720 1.00 0.00 C

ATOM 4236 C LEU 430 44.180 56.940 29.360 1.00 0.00 C

ATOM 4237 O LEU 430 44.110 58.150 29.560 1.00 0.00 O

ATOM 4238 CB LEU 430 41.910 55.910 29.770 1.00 0.00 C

ATOM 4239 CG LEU 430 41.280 57.160 30.390 1.00 0.00 C

ATOM 4240 CD1 LEU 430 40.480 57.960 29.360 1.00 0.00 C

ATOM 4241 CD2 LEU 430 40.390 56.750 31.560 1.00 0.00 C

ATOM 4242 H LEU 430 43.020 54.120 28.500 1.00 0.00 H

ATOM 4243 N MET 431 45.220 56.190 29.740 1.00 0.00 N

ATOM 4244 CA MET 431 46.350 56.700 30.560 1.00 0.00 C

ATOM 4245 C MET 431 46.830 58.120 30.220 1.00 0.00 C

ATOM 4246 O MET 431 47.390 58.390 29.150 1.00 0.00 O

ATOM 4247 CB MET 431 47.520 55.710 30.500 1.00 0.00 C

ATOM 4248 CG MET 431 48.430 55.930 31.710 1.00 0.00 C

ATOM 4249 SD MET 431 50.030 55.060 31.610 1.00 0.00 S

ATOM 4250 CE MET 431 50.470 55.230 33.320 1.00 0.00 C

ATOM 4251 H MET 431 45.260 55.210 29.550 1.00 0.00 H

ATOM 4252 N SER 432 46.590 59.010 31.180 1.00 0.00 N

ATOM 4253 CA SER 432 47.010 60.430 31.110 1.00 0.00 C

ATOM 4254 C SER 432 48.530 60.640 31.050 1.00 0.00 C

ATOM 4255 O SER 432 49.040 61.560 30.410 1.00 0.00 O

ATOM 4256 CB SER 432 46.430 61.190 32.320 1.00 0.00 C

ATOM 4257 OG SER 432 46.570 62.600 32.120 1.00 0.00 O

ATOM 4258 H SER 432 45.950 58.800 31.920 1.00 0.00 H

ATOM 4259 HG SER 432 46.120 63.080 32.880 1.00 0.00 H

ATOM 4260 N TRP 433 49.260 59.750 31.710 1.00 0.00 N

ATOM 4261 CA TRP 433 50.740 59.740 31.750 1.00 0.00 C

ATOM 4262 C TRP 433 51.350 59.450 30.360 1.00 0.00 C

ATOM 4263 O TRP 433 50.650 58.890 29.510 1.00 0.00 O

ATOM 4264 CB TRP 433 51.200 58.700 32.780 1.00 0.00 C

ATOM 4265 CG TRP 433 50.740 59.020 34.200 1.00 0.00 C

ATOM 4266 CD1 TRP 433 49.710 58.450 34.830 1.00 0.00 C

ATOM 4267 CD2 TRP 433 51.390 59.810 35.130 1.00 0.00 C

ATOM 4268 CE2 TRP 433 50.700 59.670 36.320 1.00 0.00 C

ATOM 4269 CE3 TRP 433 52.500 60.640 35.070 1.00 0.00 C

ATOM 4270 NE1 TRP 433 49.700 58.830 36.110 1.00 0.00 N

ATOM 4271 CZ2 TRP 433 51.100 60.370 37.460 1.00 0.00 C

ATOM 4272 CZ3 TRP 433 52.920 61.310 36.220 1.00 0.00 C

ATOM 4273 CH2 TRP 433 52.210 61.190 37.400 1.00 0.00 C

ATOM 4274 H TRP 433 48.830 58.970 32.180 1.00 0.00 H

ATOM 4275 HD1 TRP 433 49.000 57.760 34.380 1.00 0.00 H

ATOM 4276 HE1 TRP 433 49.080 58.450 36.810 1.00 0.00 H

ATOM 4277 HE3 TRP 433 53.030 60.790 34.130 1.00 0.00 H

ATOM 4278 HZ2 TRP 433 50.530 60.280 38.390 1.00 0.00 H

ATOM 4279 HZ3 TRP 433 53.900 61.790 36.240 1.00 0.00 H

ATOM 4280 HH2 TRP 433 52.510 61.770 38.280 1.00 0.00 H

ATOM 4281 N PRO 434 52.590 59.890 30.080 1.00 0.00 N

ATOM 4282 CA PRO 434 53.260 59.650 28.790 1.00 0.00 C

ATOM 4283 C PRO 434 53.510 58.170 28.470 1.00 0.00 C

ATOM 4284 O PRO 434 53.580 57.330 29.370 1.00 0.00 O

ATOM 4285 CB PRO 434 54.570 60.430 28.880 1.00 0.00 C

ATOM 4286 CG PRO 434 54.860 60.470 30.370 1.00 0.00 C

ATOM 4287 CD PRO 434 53.470 60.650 30.980 1.00 0.00 C

ATOM 4288 N GLU 435 53.640 57.870 27.180 1.00 0.00 N

ATOM 4289 CA GLU 435 53.890 56.490 26.680 1.00 0.00 C

ATOM 4290 C GLU 435 55.140 55.840 27.280 1.00 0.00 C

ATOM 4291 O GLU 435 55.040 54.710 27.760 1.00 0.00 O

ATOM 4292 CB GLU 435 53.970 56.440 25.150 1.00 0.00 C

ATOM 4293 CG GLU 435 54.170 55.020 24.590 1.00 0.00 C

ATOM 4294 CD GLU 435 52.960 54.100 24.770 1.00 0.00 C

ATOM 4295 OE1 GLU 435 51.940 54.430 24.130 1.00 0.00 O

ATOM 4296 OE2 GLU 435 53.040 53.140 25.580 1.00 0.00 O

ATOM 4297 H GLU 435 53.510 58.570 26.470 1.00 0.00 H

ATOM 4298 N TRP 436 56.230 56.600 27.440 1.00 0.00 N

ATOM 4299 CA TRP 436 57.490 56.080 28.010 1.00 0.00 C

ATOM 4300 C TRP 436 57.320 55.430 29.400 1.00 0.00 C

ATOM 4301 O TRP 436 58.130 54.600 29.800 1.00 0.00 O

ATOM 4302 CB TRP 436 58.610 57.130 28.050 1.00 0.00 C

ATOM 4303 CG TRP 436 58.460 58.250 29.090 1.00 0.00 C

ATOM 4304 CD1 TRP 436 57.960 59.470 28.910 1.00 0.00 C

ATOM 4305 CD2 TRP 436 58.930 58.190 30.380 1.00 0.00 C

ATOM 4306 CE2 TRP 436 58.660 59.420 30.960 1.00 0.00 C

ATOM 4307 CE3 TRP 436 59.590 57.210 31.110 1.00 0.00 C

ATOM 4308 NE1 TRP 436 58.090 60.180 30.030 1.00 0.00 N

ATOM 4309 CZ2 TRP 436 58.990 59.650 32.290 1.00 0.00 C

ATOM 4310 CZ3 TRP 436 59.950 57.450 32.420 1.00 0.00 C

ATOM 4311 CH2 TRP 436 59.630 58.660 33.020 1.00 0.00 C

ATOM 4312 H TRP 436 56.250 57.550 27.130 1.00 0.00 H

ATOM 4313 HD1 TRP 436 57.500 59.850 27.990 1.00 0.00 H

ATOM 4314 HE1 TRP 436 57.860 61.150 30.130 1.00 0.00 H

ATOM 4315 HE3 TRP 436 59.790 56.230 30.670 1.00 0.00 H

ATOM 4316 HZ2 TRP 436 58.720 60.590 32.770 1.00 0.00 H

ATOM 4317 HZ3 TRP 436 60.430 56.670 33.010 1.00 0.00 H

ATOM 4318 HH2 TRP 436 59.810 58.790 34.090 1.00 0.00 H

ATOM 4319 N MET 437 56.290 55.890 30.110 1.00 0.00 N

ATOM 4320 CA MET 437 55.870 55.340 31.400 1.00 0.00 C

ATOM 4321 C MET 437 55.370 53.890 31.390 1.00 0.00 C

ATOM 4322 O MET 437 55.510 53.190 32.400 1.00 0.00 O

ATOM 4323 CB MET 437 54.870 56.270 32.090 1.00 0.00 C

ATOM 4324 CG MET 437 55.590 57.550 32.500 1.00 0.00 C

ATOM 4325 SD MET 437 54.670 58.610 33.670 1.00 0.00 S

ATOM 4326 CE MET 437 54.720 57.580 35.130 1.00 0.00 C

ATOM 4327 H MET 437 55.720 56.630 29.760 1.00 0.00 H

ATOM 4328 N GLY 438 54.820 53.480 30.250 1.00 0.00 N

ATOM 4329 CA GLY 438 54.320 52.110 30.030 1.00 0.00 C

ATOM 4330 C GLY 438 53.150 51.710 30.940 1.00 0.00 C

ATOM 4331 O GLY 438 52.260 52.500 31.260 1.00 0.00 O

ATOM 4332 H GLY 438 54.880 54.020 29.410 1.00 0.00 H

ATOM 4333 N VAL 439 53.270 50.470 31.390 1.00 0.00 N

ATOM 4334 CA VAL 439 52.270 49.790 32.250 1.00 0.00 C

ATOM 4335 C VAL 439 52.700 50.040 33.710 1.00 0.00 C

ATOM 4336 O VAL 439 53.360 49.230 34.360 1.00 0.00 O

ATOM 4337 CB VAL 439 52.180 48.280 31.930 1.00 0.00 C

ATOM 4338 CG1 VAL 439 51.040 47.630 32.720 1.00 0.00 C

ATOM 4339 CG2 VAL 439 51.990 48.000 30.440 1.00 0.00 C

ATOM 4340 H VAL 439 54.140 49.970 31.250 1.00 0.00 H

ATOM 4341 N ALA 440 52.310 51.230 34.150 1.00 0.00 N

ATOM 4342 CA ALA 440 52.710 51.800 35.450 1.00 0.00 C

ATOM 4343 C ALA 440 52.010 51.170 36.660 1.00 0.00 C

ATOM 4344 O ALA 440 50.910 50.630 36.580 1.00 0.00 O

ATOM 4345 CB ALA 440 52.490 53.310 35.430 1.00 0.00 C

ATOM 4346 H ALA 440 51.730 51.810 33.590 1.00 0.00 H

ATOM 4347 N HIS 441 52.650 51.400 37.800 1.00 0.00 N

ATOM 4348 CA HIS 441 52.180 51.040 39.150 1.00 0.00 C

ATOM 4349 C HIS 441 50.780 51.640 39.400 1.00 0.00 C

ATOM 4350 O HIS 441 50.520 52.790 39.050 1.00 0.00 O

ATOM 4351 CB HIS 441 53.190 51.630 40.130 1.00 0.00 C

ATOM 4352 CG HIS 441 52.950 51.210 41.580 1.00 0.00 C

ATOM 4353 CD2 HIS 441 52.800 52.030 42.630 1.00 0.00 C

ATOM 4354 ND1 HIS 441 53.170 49.990 42.050 1.00 0.00 N

ATOM 4355 CE1 HIS 441 53.170 50.060 43.370 1.00 0.00 C

ATOM 4356 NE2 HIS 441 52.970 51.330 43.750 1.00 0.00 N

ATOM 4357 H HIS 441 53.520 51.900 37.800 1.00 0.00 H

ATOM 4358 HD1 HIS 441 53.460 49.190 41.500 1.00 0.00 H

ATOM 4359 N GLY 442 49.870 50.760 39.820 1.00 0.00 N

ATOM 4360 CA GLY 442 48.480 51.120 40.160 1.00 0.00 C

ATOM 4361 C GLY 442 47.450 50.860 39.060 1.00 0.00 C

ATOM 4362 O GLY 442 46.250 50.800 39.340 1.00 0.00 O

ATOM 4363 H GLY 442 50.130 49.820 40.090 1.00 0.00 H

ATOM 4364 N TYR 443 47.890 50.800 37.810 1.00 0.00 N

ATOM 4365 CA TYR 443 46.990 50.800 36.630 1.00 0.00 C

ATOM 4366 C TYR 443 46.190 49.540 36.270 1.00 0.00 C

ATOM 4367 O TYR 443 45.310 49.600 35.410 1.00 0.00 O

ATOM 4368 CB TYR 443 47.710 51.390 35.420 1.00 0.00 C

ATOM 4369 CG TYR 443 47.830 52.910 35.600 1.00 0.00 C

ATOM 4370 CD1 TYR 443 48.900 53.420 36.300 1.00 0.00 C

ATOM 4371 CD2 TYR 443 46.750 53.740 35.280 1.00 0.00 C

ATOM 4372 CE1 TYR 443 48.900 54.740 36.710 1.00 0.00 C

ATOM 4373 CE2 TYR 443 46.750 55.060 35.700 1.00 0.00 C

ATOM 4374 CZ TYR 443 47.820 55.560 36.430 1.00 0.00 C

ATOM 4375 OH TYR 443 47.770 56.820 36.930 1.00 0.00 O

ATOM 4376 H TYR 443 48.870 50.680 37.610 1.00 0.00 H

ATOM 4377 HD1 TYR 443 49.680 52.750 36.670 1.00 0.00 H

ATOM 4378 HD2 TYR 443 45.880 53.330 34.760 1.00 0.00 H

ATOM 4379 HE1 TYR 443 49.770 55.120 37.240 1.00 0.00 H

ATOM 4380 HE2 TYR 443 45.870 55.670 35.480 1.00 0.00 H

ATOM 4381 HH TYR 443 46.840 57.180 36.800 1.00 0.00 H

ATOM 4382 N GLU 444 46.520 48.420 36.890 1.00 0.00 N

ATOM 4383 CA GLU 444 45.750 47.170 36.800 1.00 0.00 C

ATOM 4384 C GLU 444 44.450 47.220 37.640 1.00 0.00 C

ATOM 4385 O GLU 444 43.460 46.570 37.290 1.00 0.00 O

ATOM 4386 CB GLU 444 46.630 45.960 37.160 1.00 0.00 C

ATOM 4387 CG GLU 444 46.860 45.620 38.650 1.00 0.00 C

ATOM 4388 CD GLU 444 47.460 46.700 39.560 1.00 0.00 C

ATOM 4389 OE1 GLU 444 48.040 47.680 39.080 1.00 0.00 O

ATOM 4390 OE2 GLU 444 47.310 46.520 40.780 1.00 0.00 O

ATOM 4391 H GLU 444 47.280 48.360 37.570 1.00 0.00 H

ATOM 4392 N ILE 445 44.430 48.070 38.660 1.00 0.00 N

ATOM 4393 CA ILE 445 43.300 48.210 39.610 1.00 0.00 C

ATOM 4394 C ILE 445 41.960 48.500 38.880 1.00 0.00 C

ATOM 4395 O ILE 445 41.000 47.770 39.090 1.00 0.00 O

ATOM 4396 CB ILE 445 43.630 49.240 40.710 1.00 0.00 C

ATOM 4397 CG1 ILE 445 44.910 48.840 41.470 1.00 0.00 C

ATOM 4398 CG2 ILE 445 42.470 49.370 41.710 1.00 0.00 C

ATOM 4399 CD ILE 445 45.490 49.950 42.340 1.00 0.00 C

ATOM 4400 H ILE 445 45.250 48.620 38.900 1.00 0.00 H

ATOM 4401 N GLU 446 41.960 49.450 37.950 1.00 0.00 N

ATOM 4402 CA GLU 446 40.820 49.770 37.050 1.00 0.00 C

ATOM 4403 C GLU 446 40.140 48.510 36.470 1.00 0.00 C

ATOM 4404 O GLU 446 38.920 48.320 36.550 1.00 0.00 O

ATOM 4405 CB GLU 446 41.340 50.600 35.870 1.00 0.00 C

ATOM 4406 CG GLU 446 41.380 52.110 36.070 1.00 0.00 C

ATOM 4407 CD GLU 446 42.290 52.860 35.070 1.00 0.00 C

ATOM 4408 OE1 GLU 446 42.290 52.550 33.860 1.00 0.00 O

ATOM 4409 OE2 GLU 446 42.950 53.820 35.530 1.00 0.00 O

ATOM 4410 H GLU 446 42.720 50.120 37.900 1.00 0.00 H

ATOM 4411 N PHE 447 41.000 47.610 35.980 1.00 0.00 N

ATOM 4412 CA PHE 447 40.640 46.300 35.410 1.00 0.00 C

ATOM 4413 C PHE 447 40.130 45.290 36.450 1.00 0.00 C

ATOM 4414 O PHE 447 39.070 44.690 36.260 1.00 0.00 O

ATOM 4415 CB PHE 447 41.850 45.730 34.660 1.00 0.00 C

ATOM 4416 CG PHE 447 42.180 46.560 33.410 1.00 0.00 C

ATOM 4417 CD1 PHE 447 43.090 47.610 33.450 1.00 0.00 C

ATOM 4418 CD2 PHE 447 41.500 46.280 32.230 1.00 0.00 C

ATOM 4419 CE1 PHE 447 43.310 48.380 32.320 1.00 0.00 C

ATOM 4420 CE2 PHE 447 41.700 47.070 31.110 1.00 0.00 C

ATOM 4421 CZ PHE 447 42.610 48.120 31.140 1.00 0.00 C

ATOM 4422 H PHE 447 41.980 47.810 35.970 1.00 0.00 H

ATOM 4423 HD1 PHE 447 43.600 47.870 34.380 1.00 0.00 H

ATOM 4424 HD2 PHE 447 40.920 45.360 32.160 1.00 0.00 H

ATOM 4425 HE1 PHE 447 43.920 49.280 32.380 1.00 0.00 H

ATOM 4426 HE2 PHE 447 41.140 46.860 30.190 1.00 0.00 H

ATOM 4427 HZ PHE 447 42.700 48.790 30.290 1.00 0.00 H

ATOM 4428 N VAL 448 40.780 45.230 37.610 1.00 0.00 N

ATOM 4429 CA VAL 448 40.360 44.380 38.750 1.00 0.00 C

ATOM 4430 C VAL 448 38.940 44.750 39.230 1.00 0.00 C

ATOM 4431 O VAL 448 38.040 43.910 39.230 1.00 0.00 O

ATOM 4432 CB VAL 448 41.400 44.440 39.890 1.00 0.00 C

ATOM 4433 CG1 VAL 448 41.030 43.550 41.080 1.00 0.00 C

ATOM 4434 CG2 VAL 448 42.800 44.010 39.410 1.00 0.00 C

ATOM 4435 H VAL 448 41.640 45.730 37.750 1.00 0.00 H

ATOM 4436 N PHE 449 38.710 46.050 39.410 1.00 0.00 N

ATOM 4437 CA PHE 449 37.400 46.610 39.840 1.00 0.00 C

ATOM 4438 C PHE 449 36.260 46.590 38.820 1.00 0.00 C

ATOM 4439 O PHE 449 35.090 46.700 39.190 1.00 0.00 O

ATOM 4440 CB PHE 449 37.600 48.010 40.410 1.00 0.00 C

ATOM 4441 CG PHE 449 38.140 47.920 41.830 1.00 0.00 C

ATOM 4442 CD1 PHE 449 39.500 47.760 42.050 1.00 0.00 C

ATOM 4443 CD2 PHE 449 37.270 47.880 42.910 1.00 0.00 C

ATOM 4444 CE1 PHE 449 40.000 47.550 43.330 1.00 0.00 C

ATOM 4445 CE2 PHE 449 37.760 47.670 44.190 1.00 0.00 C

ATOM 4446 CZ PHE 449 39.120 47.500 44.400 1.00 0.00 C

ATOM 4447 H PHE 449 39.450 46.720 39.330 1.00 0.00 H

ATOM 4448 HD1 PHE 449 40.190 47.830 41.210 1.00 0.00 H

ATOM 4449 HD2 PHE 449 36.200 48.000 42.740 1.00 0.00 H

ATOM 4450 HE1 PHE 449 41.070 47.420 43.490 1.00 0.00 H

ATOM 4451 HE2 PHE 449 37.070 47.670 45.030 1.00 0.00 H

ATOM 4452 HZ PHE 449 39.510 47.370 45.410 1.00 0.00 H

ATOM 4453 N GLY 450 36.620 46.580 37.540 1.00 0.00 N

ATOM 4454 CA GLY 450 35.650 46.520 36.440 1.00 0.00 C

ATOM 4455 C GLY 450 35.270 47.840 35.760 1.00 0.00 C

ATOM 4456 O GLY 450 34.350 47.840 34.950 1.00 0.00 O

ATOM 4457 H GLY 450 37.580 46.450 37.280 1.00 0.00 H

ATOM 4458 N MET 451 36.130 48.850 35.870 1.00 0.00 N

ATOM 4459 CA MET 451 35.880 50.180 35.270 1.00 0.00 C

ATOM 4460 C MET 451 35.590 50.170 33.750 1.00 0.00 C

ATOM 4461 O MET 451 34.530 50.680 33.380 1.00 0.00 O

ATOM 4462 CB MET 451 36.990 51.190 35.640 1.00 0.00 C

ATOM 4463 CG MET 451 37.050 51.500 37.130 1.00 0.00 C

ATOM 4464 SD MET 451 35.540 52.330 37.760 1.00 0.00 S

ATOM 4465 CE MET 451 34.860 51.030 38.760 1.00 0.00 C

ATOM 4466 H MET 451 37.010 48.750 36.340 1.00 0.00 H

ATOM 4467 N PRO 452 36.350 49.420 32.920 1.00 0.00 N

ATOM 4468 CA PRO 452 36.040 49.240 31.490 1.00 0.00 C

ATOM 4469 C PRO 452 34.680 48.600 31.150 1.00 0.00 C

ATOM 4470 O PRO 452 34.230 48.720 30.010 1.00 0.00 O

ATOM 4471 CB PRO 452 37.170 48.390 30.920 1.00 0.00 C

ATOM 4472 CG PRO 452 38.350 48.820 31.780 1.00 0.00 C

ATOM 4473 CD PRO 452 37.730 48.950 33.170 1.00 0.00 C

ATOM 4474 N LEU 453 34.040 47.910 32.090 1.00 0.00 N

ATOM 4475 CA LEU 453 32.670 47.390 31.880 1.00 0.00 C

ATOM 4476 C LEU 453 31.630 48.470 31.580 1.00 0.00 C

ATOM 4477 O LEU 453 30.600 48.200 30.970 1.00 0.00 O

ATOM 4478 CB LEU 453 32.170 46.550 33.050 1.00 0.00 C

ATOM 4479 CG LEU 453 32.440 45.060 32.840 1.00 0.00 C

ATOM 4480 CD1 LEU 453 33.910 44.710 33.080 1.00 0.00 C

ATOM 4481 CD2 LEU 453 31.490 44.250 33.710 1.00 0.00 C

ATOM 4482 H LEU 453 34.400 47.780 33.020 1.00 0.00 H

ATOM 4483 N ASN 454 31.890 49.660 32.120 1.00 0.00 N

ATOM 4484 CA ASN 454 31.080 50.850 31.830 1.00 0.00 C

ATOM 4485 C ASN 454 31.370 51.380 30.420 1.00 0.00 C

ATOM 4486 O ASN 454 32.300 52.140 30.200 1.00 0.00 O

ATOM 4487 CB ASN 454 31.340 51.940 32.880 1.00 0.00 C

ATOM 4488 CG ASN 454 30.400 53.140 32.710 1.00 0.00 C

ATOM 4489 ND2 ASN 454 30.560 54.110 33.570 1.00 0.00 N

ATOM 4490 OD1 ASN 454 29.490 53.180 31.890 1.00 0.00 O

ATOM 4491 H ASN 454 32.710 49.830 32.680 1.00 0.00 H

ATOM 4492 1HD2 ASN 454 31.240 54.020 34.290 1.00 0.00 H

ATOM 4493 2HD2 ASN 454 29.870 54.830 33.600 1.00 0.00 H

ATOM 4494 N THR 455 30.430 51.030 29.540 1.00 0.00 N

ATOM 4495 CA THR 455 30.390 51.480 28.120 1.00 0.00 C

ATOM 4496 C THR 455 30.470 53.010 27.940 1.00 0.00 C

ATOM 4497 O THR 455 31.170 53.500 27.060 1.00 0.00 O

ATOM 4498 CB THR 455 29.140 50.880 27.440 1.00 0.00 C

ATOM 4499 CG2 THR 455 27.810 51.330 28.060 1.00 0.00 C

ATOM 4500 OG1 THR 455 29.160 51.100 26.030 1.00 0.00 O

ATOM 4501 H THR 455 29.760 50.340 29.780 1.00 0.00 H

ATOM 4502 HG1 THR 455 29.070 50.190 25.580 1.00 0.00 H

ATOM 4503 N SER 456 29.890 53.730 28.910 1.00 0.00 N

ATOM 4504 CA SER 456 29.950 55.210 28.970 1.00 0.00 C

ATOM 4505 C SER 456 31.400 55.710 29.030 1.00 0.00 C

ATOM 4506 O SER 456 31.770 56.560 28.220 1.00 0.00 O

ATOM 4507 CB SER 456 29.190 55.750 30.170 1.00 0.00 C

ATOM 4508 OG SER 456 27.780 55.620 29.960 1.00 0.00 O

ATOM 4509 H SER 456 29.430 53.300 29.680 1.00 0.00 H

ATOM 4510 HG SER 456 27.280 55.620 30.840 1.00 0.00 H

ATOM 4511 N LEU 457 32.210 55.040 29.850 1.00 0.00 N

ATOM 4512 CA LEU 457 33.670 55.250 29.890 1.00 0.00 C

ATOM 4513 C LEU 457 34.260 54.590 28.640 1.00 0.00 C

ATOM 4514 O LEU 457 34.010 53.410 28.370 1.00 0.00 O

ATOM 4515 CB LEU 457 34.300 54.610 31.130 1.00 0.00 C

ATOM 4516 CG LEU 457 33.750 55.060 32.490 1.00 0.00 C

ATOM 4517 CD1 LEU 457 34.460 54.280 33.590 1.00 0.00 C

ATOM 4518 CD2 LEU 457 33.900 56.570 32.730 1.00 0.00 C

ATOM 4519 H LEU 457 31.920 54.180 30.280 1.00 0.00 H

ATOM 4520 N GLY 458 34.830 55.430 27.790 1.00 0.00 N

ATOM 4521 CA GLY 458 35.470 55.030 26.520 1.00 0.00 C

ATOM 4522 C GLY 458 36.770 54.290 26.790 1.00 0.00 C

ATOM 4523 O GLY 458 37.780 54.930 27.090 1.00 0.00 O

ATOM 4524 H GLY 458 34.830 56.430 27.950 1.00 0.00 H

ATOM 4525 N TYR 459 36.600 52.990 26.980 1.00 0.00 N

ATOM 4526 CA TYR 459 37.670 51.980 26.970 1.00 0.00 C

ATOM 4527 C TYR 459 37.410 51.080 25.760 1.00 0.00 C

ATOM 4528 O TYR 459 36.260 50.840 25.370 1.00 0.00 O

ATOM 4529 CB TYR 459 37.620 51.090 28.220 1.00 0.00 C

ATOM 4530 CG TYR 459 38.060 51.800 29.500 1.00 0.00 C

ATOM 4531 CD1 TYR 459 39.400 51.800 29.880 1.00 0.00 C

ATOM 4532 CD2 TYR 459 37.100 52.320 30.360 1.00 0.00 C

ATOM 4533 CE1 TYR 459 39.770 52.310 31.120 1.00 0.00 C

ATOM 4534 CE2 TYR 459 37.470 52.820 31.600 1.00 0.00 C

ATOM 4535 CZ TYR 459 38.800 52.800 31.990 1.00 0.00 C

ATOM 4536 OH TYR 459 39.080 52.970 33.300 1.00 0.00 O

ATOM 4537 H TYR 459 35.670 52.610 27.070 1.00 0.00 H

ATOM 4538 HD1 TYR 459 40.150 51.320 29.240 1.00 0.00 H

ATOM 4539 HD2 TYR 459 36.040 52.260 30.080 1.00 0.00 H

ATOM 4540 HE1 TYR 459 40.820 52.310 31.410 1.00 0.00 H

ATOM 4541 HE2 TYR 459 36.710 53.190 32.280 1.00 0.00 H

ATOM 4542 HH TYR 459 38.730 53.880 33.610 1.00 0.00 H

ATOM 4543 N THR 460 38.470 50.530 25.190 1.00 0.00 N

ATOM 4544 CA THR 460 38.330 49.690 23.980 1.00 0.00 C

ATOM 4545 C THR 460 37.520 48.440 24.320 1.00 0.00 C

ATOM 4546 O THR 460 37.490 47.950 25.450 1.00 0.00 O

ATOM 4547 CB THR 460 39.650 49.280 23.300 1.00 0.00 C

ATOM 4548 CG2 THR 460 40.590 50.470 23.090 1.00 0.00 C

ATOM 4549 OG1 THR 460 40.270 48.190 23.970 1.00 0.00 O

ATOM 4550 H THR 460 39.370 50.530 25.660 1.00 0.00 H

ATOM 4551 HG1 THR 460 41.290 48.220 23.800 1.00 0.00 H

ATOM 4552 N LYS 461 36.900 47.890 23.280 1.00 0.00 N

ATOM 4553 CA LYS 461 36.120 46.640 23.380 1.00 0.00 C

ATOM 4554 C LYS 461 36.960 45.500 24.010 1.00 0.00 C

ATOM 4555 O LYS 461 36.570 44.940 25.030 1.00 0.00 O

ATOM 4556 CB LYS 461 35.610 46.250 21.990 1.00 0.00 C

ATOM 4557 CG LYS 461 34.810 47.360 21.320 1.00 0.00 C

ATOM 4558 CD LYS 461 34.100 46.880 20.050 1.00 0.00 C

ATOM 4559 CE LYS 461 32.800 46.130 20.360 1.00 0.00 C

ATOM 4560 NZ LYS 461 31.770 47.040 20.910 1.00 0.00 N1+

ATOM 4561 H LYS 461 36.910 48.340 22.380 1.00 0.00 H

ATOM 4562 HZ1 LYS 461 30.900 46.550 20.970 1.00 0.00 H

ATOM 4563 HZ2 LYS 461 32.030 47.380 21.820 1.00 0.00 H

ATOM 4564 HZ3 LYS 461 31.650 47.810 20.290 1.00 0.00 H

ATOM 4565 N ASN 462 38.240 45.490 23.600 1.00 0.00 N

ATOM 4566 CA ASN 462 39.300 44.600 24.090 1.00 0.00 C

ATOM 4567 C ASN 462 39.600 44.780 25.590 1.00 0.00 C

ATOM 4568 O ASN 462 39.710 43.800 26.320 1.00 0.00 O

ATOM 4569 CB ASN 462 40.580 44.830 23.290 1.00 0.00 C

ATOM 4570 CG ASN 462 40.390 44.450 21.820 1.00 0.00 C

ATOM 4571 ND2 ASN 462 40.910 45.240 20.910 1.00 0.00 N

ATOM 4572 OD1 ASN 462 39.870 43.390 21.490 1.00 0.00 O

ATOM 4573 H ASN 462 38.540 46.150 22.910 1.00 0.00 H

ATOM 4574 1HD2 ASN 462 41.470 46.040 21.120 1.00 0.00 H

ATOM 4575 2HD2 ASN 462 40.630 45.110 19.960 1.00 0.00 H

ATOM 4576 N GLU 463 39.660 46.040 26.030 1.00 0.00 N

ATOM 4577 CA GLU 463 39.850 46.390 27.450 1.00 0.00 C

ATOM 4578 C GLU 463 38.740 45.830 28.350 1.00 0.00 C

ATOM 4579 O GLU 463 39.030 44.950 29.150 1.00 0.00 O

ATOM 4580 CB GLU 463 40.020 47.900 27.620 1.00 0.00 C

ATOM 4581 CG GLU 463 41.380 48.290 27.050 1.00 0.00 C

ATOM 4582 CD GLU 463 41.680 49.780 27.080 1.00 0.00 C

ATOM 4583 OE1 GLU 463 40.820 50.580 26.670 1.00 0.00 O

ATOM 4584 OE2 GLU 463 42.850 50.100 27.310 1.00 0.00 O

ATOM 4585 H GLU 463 39.610 46.830 25.410 1.00 0.00 H

ATOM 4586 N VAL 464 37.490 46.030 27.910 1.00 0.00 N

ATOM 4587 CA VAL 464 36.290 45.500 28.600 1.00 0.00 C

ATOM 4588 C VAL 464 36.370 43.970 28.750 1.00 0.00 C

ATOM 4589 O VAL 464 36.080 43.420 29.810 1.00 0.00 O

ATOM 4590 CB VAL 464 34.980 45.860 27.860 1.00 0.00 C

ATOM 4591 CG1 VAL 464 33.750 45.620 28.740 1.00 0.00 C

ATOM 4592 CG2 VAL 464 34.960 47.280 27.300 1.00 0.00 C

ATOM 4593 H VAL 464 37.320 46.610 27.110 1.00 0.00 H

ATOM 4594 N ASN 465 36.790 43.310 27.670 1.00 0.00 N

ATOM 4595 CA ASN 465 37.030 41.850 27.650 1.00 0.00 C

ATOM 4596 C ASN 465 38.090 41.400 28.660 1.00 0.00 C

ATOM 4597 O ASN 465 37.780 40.620 29.560 1.00 0.00 O

ATOM 4598 CB ASN 465 37.370 41.370 26.240 1.00 0.00 C

ATOM 4599 CG ASN 465 36.110 41.440 25.380 1.00 0.00 C

ATOM 4600 ND2 ASN 465 35.190 40.540 25.650 1.00 0.00 N

ATOM 4601 OD1 ASN 465 35.960 42.270 24.500 1.00 0.00 O

ATOM 4602 H ASN 465 36.950 43.780 26.790 1.00 0.00 H

ATOM 4603 1HD2 ASN 465 35.390 39.830 26.340 1.00 0.00 H

ATOM 4604 2HD2 ASN 465 34.360 40.540 25.100 1.00 0.00 H

ATOM 4605 N MET 466 39.250 42.050 28.620 1.00 0.00 N

ATOM 4606 CA MET 466 40.360 41.780 29.560 1.00 0.00 C

ATOM 4607 C MET 466 39.930 42.000 31.020 1.00 0.00 C

ATOM 4608 O MET 466 40.080 41.080 31.830 1.00 0.00 O

ATOM 4609 CB MET 466 41.560 42.660 29.200 1.00 0.00 C

ATOM 4610 CG MET 466 42.820 42.140 29.910 1.00 0.00 C

ATOM 4611 SD MET 466 44.320 43.140 29.670 1.00 0.00 S

ATOM 4612 CE MET 466 43.840 44.570 30.610 1.00 0.00 C

ATOM 4613 H MET 466 39.430 42.770 27.930 1.00 0.00 H

ATOM 4614 N THR 467 39.190 43.080 31.270 1.00 0.00 N

ATOM 4615 CA THR 467 38.590 43.420 32.580 1.00 0.00 C

ATOM 4616 C THR 467 37.670 42.310 33.100 1.00 0.00 C

ATOM 4617 O THR 467 37.900 41.760 34.170 1.00 0.00 O

ATOM 4618 CB THR 467 37.780 44.720 32.440 1.00 0.00 C

ATOM 4619 CG2 THR 467 37.420 45.300 33.800 1.00 0.00 C

ATOM 4620 OG1 THR 467 38.550 45.680 31.740 1.00 0.00 O

ATOM 4621 H THR 467 39.130 43.830 30.600 1.00 0.00 H

ATOM 4622 HG1 THR 467 38.600 46.550 32.250 1.00 0.00 H

ATOM 4623 N LYS 468 36.680 41.920 32.280 1.00 0.00 N

ATOM 4624 CA LYS 468 35.740 40.840 32.650 1.00 0.00 C

ATOM 4625 C LYS 468 36.440 39.480 32.880 1.00 0.00 C

ATOM 4626 O LYS 468 36.050 38.700 33.740 1.00 0.00 O

ATOM 4627 CB LYS 468 34.600 40.760 31.620 1.00 0.00 C

ATOM 4628 CG LYS 468 33.520 39.770 32.080 1.00 0.00 C

ATOM 4629 CD LYS 468 32.200 39.890 31.320 1.00 0.00 C

ATOM 4630 CE LYS 468 31.320 41.040 31.830 1.00 0.00 C

ATOM 4631 NZ LYS 468 30.020 41.040 31.140 1.00 0.00 N1+

ATOM 4632 H LYS 468 36.520 42.370 31.400 1.00 0.00 H

ATOM 4633 HZ1 LYS 468 29.430 41.760 31.520 1.00 0.00 H

ATOM 4634 HZ2 LYS 468 30.160 41.200 30.160 1.00 0.00 H

ATOM 4635 HZ3 LYS 468 29.580 40.150 31.260 1.00 0.00 H

ATOM 4636 N LYS 469 37.530 39.240 32.150 1.00 0.00 N

ATOM 4637 CA LYS 469 38.410 38.080 32.420 1.00 0.00 C

ATOM 4638 C LYS 469 39.040 38.190 33.810 1.00 0.00 C

ATOM 4639 O LYS 469 38.790 37.330 34.660 1.00 0.00 O

ATOM 4640 CB LYS 469 39.520 37.950 31.380 1.00 0.00 C

ATOM 4641 CG LYS 469 38.950 37.650 30.010 1.00 0.00 C

ATOM 4642 CD LYS 469 40.010 37.980 28.980 1.00 0.00 C

ATOM 4643 CE LYS 469 39.450 37.960 27.570 1.00 0.00 C

ATOM 4644 NZ LYS 469 39.420 36.620 26.980 1.00 0.00 N1+

ATOM 4645 H LYS 469 37.790 39.820 31.370 1.00 0.00 H

ATOM 4646 HZ1 LYS 469 39.260 36.720 26.000 1.00 0.00 H

ATOM 4647 HZ2 LYS 469 40.300 36.150 27.120 1.00 0.00 H

ATOM 4648 HZ3 LYS 469 38.680 36.060 27.370 1.00 0.00 H

ATOM 4649 N ILE 470 39.700 39.320 34.070 1.00 0.00 N

ATOM 4650 CA ILE 470 40.400 39.610 35.340 1.00 0.00 C

ATOM 4651 C ILE 470 39.450 39.480 36.550 1.00 0.00 C

ATOM 4652 O ILE 470 39.660 38.590 37.370 1.00 0.00 O

ATOM 4653 CB ILE 470 41.120 40.970 35.240 1.00 0.00 C

ATOM 4654 CG1 ILE 470 42.260 40.850 34.210 1.00 0.00 C

ATOM 4655 CG2 ILE 470 41.670 41.460 36.600 1.00 0.00 C

ATOM 4656 CD ILE 470 42.820 42.200 33.730 1.00 0.00 C

ATOM 4657 H ILE 470 39.750 40.060 33.390 1.00 0.00 H

ATOM 4658 N MET 471 38.310 40.160 36.480 1.00 0.00 N

ATOM 4659 CA MET 471 37.290 40.080 37.560 1.00 0.00 C

ATOM 4660 C MET 471 36.850 38.630 37.850 1.00 0.00 C

ATOM 4661 O MET 471 36.900 38.200 39.000 1.00 0.00 O

ATOM 4662 CB MET 471 36.040 40.890 37.230 1.00 0.00 C

ATOM 4663 CG MET 471 36.270 42.390 37.060 1.00 0.00 C

ATOM 4664 SD MET 471 34.710 43.330 37.190 1.00 0.00 S

ATOM 4665 CE MET 471 33.730 42.550 35.930 1.00 0.00 C

ATOM 4666 H MET 471 38.130 40.830 35.760 1.00 0.00 H

ATOM 4667 N LYS 472 36.580 37.870 36.780 1.00 0.00 N

ATOM 4668 CA LYS 472 36.300 36.430 36.900 1.00 0.00 C

ATOM 4669 C LYS 472 37.430 35.640 37.560 1.00 0.00 C

ATOM 4670 O LYS 472 37.170 35.060 38.610 1.00 0.00 O

ATOM 4671 CB LYS 472 35.900 35.790 35.570 1.00 0.00 C

ATOM 4672 CG LYS 472 34.410 36.020 35.270 1.00 0.00 C

ATOM 4673 CD LYS 472 33.490 35.280 36.230 1.00 0.00 C

ATOM 4674 CE LYS 472 32.030 35.660 35.950 1.00 0.00 C

ATOM 4675 NZ LYS 472 31.180 35.300 37.090 1.00 0.00 N1+

ATOM 4676 H LYS 472 36.510 38.250 35.850 1.00 0.00 H

ATOM 4677 HZ1 LYS 472 30.230 35.520 36.880 1.00 0.00 H

ATOM 4678 HZ2 LYS 472 31.270 34.320 37.290 1.00 0.00 H

ATOM 4679 HZ3 LYS 472 31.500 35.840 37.880 1.00 0.00 H

ATOM 4680 N HIS 473 38.670 35.830 37.120 1.00 0.00 N

ATOM 4681 CA HIS 473 39.850 35.180 37.740 1.00 0.00 C

ATOM 4682 C HIS 473 39.950 35.440 39.250 1.00 0.00 C

ATOM 4683 O HIS 473 39.930 34.490 40.040 1.00 0.00 O

ATOM 4684 CB HIS 473 41.160 35.620 37.080 1.00 0.00 C

ATOM 4685 CG HIS 473 41.400 35.020 35.700 1.00 0.00 C

ATOM 4686 CD2 HIS 473 41.250 35.630 34.530 1.00 0.00 C

ATOM 4687 ND1 HIS 473 41.840 33.790 35.460 1.00 0.00 N

ATOM 4688 CE1 HIS 473 41.930 33.630 34.150 1.00 0.00 C

ATOM 4689 NE2 HIS 473 41.570 34.770 33.560 1.00 0.00 N

ATOM 4690 H HIS 473 38.870 36.470 36.380 1.00 0.00 H

ATOM 4691 HE2 HIS 473 41.670 34.980 32.590 1.00 0.00 H

ATOM 4692 N TRP 474 39.780 36.710 39.630 1.00 0.00 N

ATOM 4693 CA TRP 474 39.830 37.170 41.030 1.00 0.00 C

ATOM 4694 C TRP 474 38.700 36.570 41.880 1.00 0.00 C

ATOM 4695 O TRP 474 38.980 35.810 42.810 1.00 0.00 O

ATOM 4696 CB TRP 474 39.840 38.710 41.080 1.00 0.00 C

ATOM 4697 CG TRP 474 41.250 39.320 41.040 1.00 0.00 C

ATOM 4698 CD1 TRP 474 41.730 40.170 41.960 1.00 0.00 C

ATOM 4699 CD2 TRP 474 42.220 39.260 40.030 1.00 0.00 C

ATOM 4700 CE2 TRP 474 43.240 40.110 40.420 1.00 0.00 C

ATOM 4701 CE3 TRP 474 42.320 38.570 38.850 1.00 0.00 C

ATOM 4702 NE1 TRP 474 42.920 40.640 41.580 1.00 0.00 N

ATOM 4703 CZ2 TRP 474 44.370 40.250 39.620 1.00 0.00 C

ATOM 4704 CZ3 TRP 474 43.440 38.690 38.040 1.00 0.00 C

ATOM 4705 CH2 TRP 474 44.470 39.530 38.440 1.00 0.00 C

ATOM 4706 H TRP 474 39.630 37.440 38.960 1.00 0.00 H

ATOM 4707 HD1 TRP 474 41.200 40.490 42.850 1.00 0.00 H

ATOM 4708 HE1 TRP 474 43.440 41.350 42.070 1.00 0.00 H

ATOM 4709 HE3 TRP 474 41.510 37.920 38.530 1.00 0.00 H

ATOM 4710 HZ2 TRP 474 45.140 40.980 39.870 1.00 0.00 H

ATOM 4711 HZ3 TRP 474 43.480 38.180 37.080 1.00 0.00 H

ATOM 4712 HH2 TRP 474 45.370 39.630 37.820 1.00 0.00 H

ATOM 4713 N THR 475 37.460 36.660 41.400 1.00 0.00 N

ATOM 4714 CA THR 475 36.270 36.050 42.040 1.00 0.00 C

ATOM 4715 C THR 475 36.360 34.510 42.090 1.00 0.00 C

ATOM 4716 O THR 475 35.990 33.900 43.090 1.00 0.00 O

ATOM 4717 CB THR 475 35.000 36.460 41.300 1.00 0.00 C

ATOM 4718 CG2 THR 475 33.710 35.900 41.920 1.00 0.00 C

ATOM 4719 OG1 THR 475 34.930 37.880 41.300 1.00 0.00 O

ATOM 4720 H THR 475 37.240 37.260 40.610 1.00 0.00 H

ATOM 4721 HG1 THR 475 34.350 38.130 40.510 1.00 0.00 H

ATOM 4722 N ASN 476 36.750 33.900 40.980 1.00 0.00 N

ATOM 4723 CA ASN 476 36.940 32.440 40.890 1.00 0.00 C

ATOM 4724 C ASN 476 37.940 31.940 41.930 1.00 0.00 C

ATOM 4725 O ASN 476 37.550 31.190 42.810 1.00 0.00 O

ATOM 4726 CB ASN 476 37.340 32.010 39.470 1.00 0.00 C

ATOM 4727 CG ASN 476 36.220 32.230 38.450 1.00 0.00 C

ATOM 4728 ND2 ASN 476 36.600 32.360 37.200 1.00 0.00 N

ATOM 4729 OD1 ASN 476 35.040 32.300 38.770 1.00 0.00 O

ATOM 4730 H ASN 476 36.930 34.410 40.120 1.00 0.00 H

ATOM 4731 1HD2 ASN 476 37.580 32.320 36.970 1.00 0.00 H

ATOM 4732 2HD2 ASN 476 35.900 32.510 36.510 1.00 0.00 H

ATOM 4733 N PHE 477 39.060 32.650 42.040 1.00 0.00 N

ATOM 4734 CA PHE 477 40.060 32.420 43.100 1.00 0.00 C

ATOM 4735 C PHE 477 39.470 32.660 44.510 1.00 0.00 C

ATOM 4736 O PHE 477 39.580 31.800 45.380 1.00 0.00 O

ATOM 4737 CB PHE 477 41.270 33.320 42.850 1.00 0.00 C

ATOM 4738 CG PHE 477 42.360 33.100 43.910 1.00 0.00 C

ATOM 4739 CD1 PHE 477 43.250 32.050 43.770 1.00 0.00 C

ATOM 4740 CD2 PHE 477 42.380 33.890 45.050 1.00 0.00 C

ATOM 4741 CE1 PHE 477 44.160 31.780 44.770 1.00 0.00 C

ATOM 4742 CE2 PHE 477 43.300 33.640 46.050 1.00 0.00 C

ATOM 4743 CZ PHE 477 44.190 32.580 45.910 1.00 0.00 C

ATOM 4744 H PHE 477 39.310 33.360 41.360 1.00 0.00 H

ATOM 4745 HD1 PHE 477 43.170 31.380 42.920 1.00 0.00 H

ATOM 4746 HD2 PHE 477 41.610 34.650 45.190 1.00 0.00 H

ATOM 4747 HE1 PHE 477 44.850 30.940 44.680 1.00 0.00 H

ATOM 4748 HE2 PHE 477 43.360 34.290 46.920 1.00 0.00 H

ATOM 4749 HZ PHE 477 44.910 32.390 46.690 1.00 0.00 H

ATOM 4750 N ALA 478 38.760 33.780 44.670 1.00 0.00 N

ATOM 4751 CA ALA 478 38.050 34.110 45.920 1.00 0.00 C

ATOM 4752 C ALA 478 37.090 32.990 46.380 1.00 0.00 C

ATOM 4753 O ALA 478 37.240 32.470 47.480 1.00 0.00 O

ATOM 4754 CB ALA 478 37.280 35.410 45.720 1.00 0.00 C

ATOM 4755 H ALA 478 38.800 34.530 44.000 1.00 0.00 H

ATOM 4756 N ARG 479 36.260 32.510 45.460 1.00 0.00 N

ATOM 4757 CA ARG 479 35.280 31.430 45.710 1.00 0.00 C

ATOM 4758 C ARG 479 35.930 30.050 45.880 1.00 0.00 C

ATOM 4759 O ARG 479 35.830 29.460 46.960 1.00 0.00 O

ATOM 4760 CB ARG 479 34.230 31.360 44.590 1.00 0.00 C

ATOM 4761 CG ARG 479 33.460 32.670 44.390 1.00 0.00 C

ATOM 4762 CD ARG 479 32.340 32.590 43.360 1.00 0.00 C

ATOM 4763 NE ARG 479 32.880 32.150 42.060 1.00 0.00 N

ATOM 4764 CZ ARG 479 32.450 31.120 41.330 1.00 0.00 C

ATOM 4765 NH1 ARG 479 31.350 30.410 41.630 1.00 0.00 N

ATOM 4766 NH2 ARG 479 33.140 30.800 40.240 1.00 0.00 N

ATOM 4767 H ARG 479 36.210 32.940 44.550 1.00 0.00 H

ATOM 4768 HE ARG 479 33.680 32.670 41.740 1.00 0.00 H

ATOM 4769 1HH1 ARG 479 30.750 30.680 42.380 1.00 0.00 H

ATOM 4770 2HH1 ARG 479 31.130 29.580 41.100 1.00 0.00 H

ATOM 4771 1HH2 ARG 479 33.910 31.360 39.910 1.00 0.00 H

ATOM 4772 2HH2 ARG 479 33.020 29.890 39.810 1.00 0.00 H

ATOM 4773 N THR 480 36.760 29.650 44.920 1.00 0.00 N

ATOM 4774 CA THR 480 37.250 28.260 44.800 1.00 0.00 C

ATOM 4775 C THR 480 38.690 28.010 45.270 1.00 0.00 C

ATOM 4776 O THR 480 38.970 26.930 45.780 1.00 0.00 O

ATOM 4777 CB THR 480 37.170 27.720 43.370 1.00 0.00 C

ATOM 4778 CG2 THR 480 35.750 27.790 42.800 1.00 0.00 C

ATOM 4779 OG1 THR 480 38.110 28.410 42.540 1.00 0.00 O

ATOM 4780 H THR 480 37.010 30.250 44.150 1.00 0.00 H

ATOM 4781 HG1 THR 480 37.730 28.500 41.580 1.00 0.00 H

ATOM 4782 N GLY 481 39.570 29.010 45.100 1.00 0.00 N

ATOM 4783 CA GLY 481 41.030 28.860 45.280 1.00 0.00 C

ATOM 4784 C GLY 481 41.800 28.800 43.950 1.00 0.00 C

ATOM 4785 O GLY 481 43.030 28.660 43.960 1.00 0.00 O

ATOM 4786 H GLY 481 39.270 29.950 44.900 1.00 0.00 H

ATOM 4787 N GLN 482 41.060 28.710 42.860 1.00 0.00 N

ATOM 4788 CA GLN 482 41.590 28.570 41.490 1.00 0.00 C

ATOM 4789 C GLN 482 41.030 29.690 40.590 1.00 0.00 C

ATOM 4790 O GLN 482 39.810 29.850 40.520 1.00 0.00 O

ATOM 4791 CB GLN 482 41.200 27.210 40.910 1.00 0.00 C

ATOM 4792 CG GLN 482 41.740 26.020 41.730 1.00 0.00 C

ATOM 4793 CD GLN 482 41.300 24.680 41.140 1.00 0.00 C

ATOM 4794 NE2 GLN 482 40.110 24.240 41.500 1.00 0.00 N

ATOM 4795 OE1 GLN 482 42.060 23.970 40.490 1.00 0.00 O

ATOM 4796 H GLN 482 40.050 28.700 42.900 1.00 0.00 H

ATOM 4797 1HE2 GLN 482 39.550 24.720 42.160 1.00 0.00 H

ATOM 4798 2HE2 GLN 482 39.760 23.460 40.990 1.00 0.00 H

ATOM 4799 N PRO 483 41.900 30.500 39.980 1.00 0.00 N

ATOM 4800 CA PRO 483 41.490 31.530 39.000 1.00 0.00 C

ATOM 4801 C PRO 483 40.690 31.010 37.790 1.00 0.00 C

ATOM 4802 O PRO 483 39.720 31.660 37.390 1.00 0.00 O

ATOM 4803 CB PRO 483 42.800 32.150 38.530 1.00 0.00 C

ATOM 4804 CG PRO 483 43.720 32.020 39.750 1.00 0.00 C

ATOM 4805 CD PRO 483 43.320 30.670 40.340 1.00 0.00 C

ATOM 4806 N GLY 484 40.970 29.770 37.370 1.00 0.00 N

ATOM 4807 CA GLY 484 40.500 29.240 36.080 1.00 0.00 C

ATOM 4808 C GLY 484 39.380 28.180 36.120 1.00 0.00 C

ATOM 4809 O GLY 484 38.900 27.750 37.170 1.00 0.00 O

ATOM 4810 H GLY 484 41.560 29.140 37.880 1.00 0.00 H

ATOM 4811 N ILE 485 38.860 28.010 34.910 1.00 0.00 N

ATOM 4812 CA ILE 485 37.790 27.060 34.510 1.00 0.00 C

ATOM 4813 C ILE 485 37.980 26.690 33.030 1.00 0.00 C

ATOM 4814 O ILE 485 38.500 25.610 32.730 1.00 0.00 O

ATOM 4815 CB ILE 485 36.360 27.580 34.800 1.00 0.00 C

ATOM 4816 CG1 ILE 485 36.220 29.100 34.610 1.00 0.00 C

ATOM 4817 CG2 ILE 485 35.890 27.080 36.170 1.00 0.00 C

ATOM 4818 CD ILE 485 34.790 29.560 34.300 1.00 0.00 C

ATOM 4819 H ILE 485 39.250 28.520 34.140 1.00 0.00 H

ATOM 4820 N ASP 486 37.410 27.500 32.150 1.00 0.00 N

ATOM 4821 CA ASP 486 37.750 27.580 30.720 1.00 0.00 C

ATOM 4822 C ASP 486 38.790 28.710 30.510 1.00 0.00 C

ATOM 4823 O ASP 486 39.030 29.530 31.400 1.00 0.00 O

ATOM 4824 CB ASP 486 36.500 27.920 29.900 1.00 0.00 C

ATOM 4825 CG ASP 486 35.210 27.110 30.110 1.00 0.00 C

ATOM 4826 OD1 ASP 486 35.260 25.950 30.570 1.00 0.00 O

ATOM 4827 OD2 ASP 486 34.150 27.680 29.800 1.00 0.00 O

ATOM 4828 H ASP 486 36.530 27.930 32.360 1.00 0.00 H

ATOM 4829 N GLY 487 39.420 28.680 29.340 1.00 0.00 N

ATOM 4830 CA GLY 487 40.380 29.700 28.870 1.00 0.00 C

ATOM 4831 C GLY 487 41.820 29.150 28.810 1.00 0.00 C

ATOM 4832 O GLY 487 42.050 27.950 28.710 1.00 0.00 O

ATOM 4833 H GLY 487 39.360 27.860 28.760 1.00 0.00 H

ATOM 4834 N ALA 488 42.770 30.060 28.670 1.00 0.00 N

ATOM 4835 CA ALA 488 44.190 29.710 28.870 1.00 0.00 C

ATOM 4836 C ALA 488 44.350 29.400 30.360 1.00 0.00 C

ATOM 4837 O ALA 488 44.140 30.300 31.180 1.00 0.00 O

ATOM 4838 CB ALA 488 45.060 30.900 28.430 1.00 0.00 C

ATOM 4839 H ALA 488 42.590 31.010 28.410 1.00 0.00 H

ATOM 4840 N ALA 489 44.450 28.110 30.710 1.00 0.00 N

ATOM 4841 CA ALA 489 44.510 27.710 32.130 1.00 0.00 C

ATOM 4842 C ALA 489 45.710 28.340 32.840 1.00 0.00 C

ATOM 4843 O ALA 489 46.750 28.570 32.220 1.00 0.00 O

ATOM 4844 CB ALA 489 44.560 26.180 32.280 1.00 0.00 C

ATOM 4845 H ALA 489 44.450 27.350 30.050 1.00 0.00 H

ATOM 4846 N TRP 490 45.510 28.640 34.120 1.00 0.00 N

ATOM 4847 CA TRP 490 46.540 29.270 34.960 1.00 0.00 C

ATOM 4848 C TRP 490 47.260 28.190 35.780 1.00 0.00 C

ATOM 4849 O TRP 490 46.660 27.620 36.700 1.00 0.00 O

ATOM 4850 CB TRP 490 45.900 30.320 35.880 1.00 0.00 C

ATOM 4851 CG TRP 490 46.880 31.390 36.380 1.00 0.00 C

ATOM 4852 CD1 TRP 490 48.180 31.290 36.640 1.00 0.00 C

ATOM 4853 CD2 TRP 490 46.490 32.660 36.790 1.00 0.00 C

ATOM 4854 CE2 TRP 490 47.610 33.250 37.360 1.00 0.00 C

ATOM 4855 CE3 TRP 490 45.300 33.350 36.690 1.00 0.00 C

ATOM 4856 NE1 TRP 490 48.620 32.400 37.230 1.00 0.00 N

ATOM 4857 CZ2 TRP 490 47.520 34.540 37.880 1.00 0.00 C

ATOM 4858 CZ3 TRP 490 45.210 34.650 37.210 1.00 0.00 C

ATOM 4859 CH2 TRP 490 46.320 35.230 37.800 1.00 0.00 C

ATOM 4860 H TRP 490 44.630 28.460 34.560 1.00 0.00 H

ATOM 4861 HD1 TRP 490 48.830 30.470 36.320 1.00 0.00 H

ATOM 4862 HE1 TRP 490 49.580 32.600 37.440 1.00 0.00 H

ATOM 4863 HE3 TRP 490 44.430 32.910 36.220 1.00 0.00 H

ATOM 4864 HZ2 TRP 490 48.380 35.000 38.350 1.00 0.00 H

ATOM 4865 HZ3 TRP 490 44.250 35.170 37.190 1.00 0.00 H

ATOM 4866 HH2 TRP 490 46.240 36.240 38.220 1.00 0.00 H

ATOM 4867 N PRO 491 48.470 27.800 35.350 1.00 0.00 N

ATOM 4868 CA PRO 491 49.280 26.820 36.100 1.00 0.00 C

ATOM 4869 C PRO 491 49.690 27.400 37.450 1.00 0.00 C

ATOM 4870 O PRO 491 49.870 28.610 37.620 1.00 0.00 O

ATOM 4871 CB PRO 491 50.520 26.600 35.240 1.00 0.00 C

ATOM 4872 CG PRO 491 50.020 26.890 33.820 1.00 0.00 C

ATOM 4873 CD PRO 491 49.100 28.090 34.050 1.00 0.00 C

ATOM 4874 N HIS 492 49.790 26.500 38.420 1.00 0.00 N

ATOM 4875 CA HIS 492 50.280 26.850 39.770 1.00 0.00 C

ATOM 4876 C HIS 492 51.790 27.120 39.740 1.00 0.00 C

ATOM 4877 O HIS 492 52.560 26.390 39.120 1.00 0.00 O

ATOM 4878 CB HIS 492 49.930 25.760 40.790 1.00 0.00 C

ATOM 4879 CG HIS 492 50.360 24.350 40.380 1.00 0.00 C

ATOM 4880 CD2 HIS 492 51.500 23.740 40.700 1.00 0.00 C

ATOM 4881 ND1 HIS 492 49.650 23.520 39.610 1.00 0.00 N

ATOM 4882 CE1 HIS 492 50.390 22.420 39.420 1.00 0.00 C

ATOM 4883 NE2 HIS 492 51.530 22.550 40.100 1.00 0.00 N

ATOM 4884 H HIS 492 49.640 25.530 38.250 1.00 0.00 H

ATOM 4885 HE2 HIS 492 52.290 21.900 40.120 1.00 0.00 H

ATOM 4886 N PHE 493 52.150 28.220 40.390 1.00 0.00 N

ATOM 4887 CA PHE 493 53.530 28.690 40.550 1.00 0.00 C

ATOM 4888 C PHE 493 54.270 27.840 41.610 1.00 0.00 C

ATOM 4889 O PHE 493 53.920 27.860 42.790 1.00 0.00 O

ATOM 4890 CB PHE 493 53.520 30.170 40.950 1.00 0.00 C

ATOM 4891 CG PHE 493 54.930 30.740 41.110 1.00 0.00 C

ATOM 4892 CD1 PHE 493 55.630 30.530 42.290 1.00 0.00 C

ATOM 4893 CD2 PHE 493 55.530 31.410 40.050 1.00 0.00 C

ATOM 4894 CE1 PHE 493 56.940 30.970 42.420 1.00 0.00 C

ATOM 4895 CE2 PHE 493 56.840 31.850 40.190 1.00 0.00 C

ATOM 4896 CZ PHE 493 57.540 31.640 41.370 1.00 0.00 C

ATOM 4897 H PHE 493 51.460 28.780 40.870 1.00 0.00 H

ATOM 4898 HD1 PHE 493 55.170 29.980 43.100 1.00 0.00 H

ATOM 4899 HD2 PHE 493 55.000 31.550 39.110 1.00 0.00 H

ATOM 4900 HE1 PHE 493 57.550 30.630 43.260 1.00 0.00 H

ATOM 4901 HE2 PHE 493 57.360 32.260 39.320 1.00 0.00 H

ATOM 4902 HZ PHE 493 58.580 31.970 41.450 1.00 0.00 H

ATOM 4903 N THR 494 55.250 27.080 41.130 1.00 0.00 N

ATOM 4904 CA THR 494 56.160 26.310 42.010 1.00 0.00 C

ATOM 4905 C THR 494 57.580 26.920 41.900 1.00 0.00 C

ATOM 4906 O THR 494 57.740 27.990 41.300 1.00 0.00 O

ATOM 4907 CB THR 494 56.190 24.820 41.610 1.00 0.00 C

ATOM 4908 CG2 THR 494 54.800 24.190 41.620 1.00 0.00 C

ATOM 4909 OG1 THR 494 56.800 24.640 40.330 1.00 0.00 O

ATOM 4910 H THR 494 55.420 27.000 40.140 1.00 0.00 H

ATOM 4911 HG1 THR 494 56.820 23.650 40.140 1.00 0.00 H

ATOM 4912 N ALA 495 58.600 26.200 42.360 1.00 0.00 N

ATOM 4913 CA ALA 495 60.010 26.590 42.120 1.00 0.00 C

ATOM 4914 C ALA 495 60.400 26.570 40.620 1.00 0.00 C

ATOM 4915 O ALA 495 61.410 27.140 40.210 1.00 0.00 O

ATOM 4916 CB ALA 495 60.950 25.710 42.940 1.00 0.00 C

ATOM 4917 H ALA 495 58.480 25.370 42.900 1.00 0.00 H

ATOM 4918 N GLN 496 59.600 25.860 39.810 1.00 0.00 N

ATOM 4919 CA GLN 496 59.640 25.890 38.330 1.00 0.00 C

ATOM 4920 C GLN 496 59.310 27.280 37.750 1.00 0.00 C

ATOM 4921 O GLN 496 59.770 27.640 36.670 1.00 0.00 O

ATOM 4922 CB GLN 496 58.680 24.830 37.810 1.00 0.00 C

ATOM 4923 CG GLN 496 58.570 24.710 36.290 1.00 0.00 C

ATOM 4924 CD GLN 496 57.870 23.410 35.880 1.00 0.00 C

ATOM 4925 NE2 GLN 496 56.810 23.060 36.560 1.00 0.00 N

ATOM 4926 OE1 GLN 496 58.340 22.650 35.040 1.00 0.00 O

ATOM 4927 H GLN 496 58.880 25.280 40.200 1.00 0.00 H

ATOM 4928 1HE2 GLN 496 56.450 23.630 37.290 1.00 0.00 H

ATOM 4929 2HE2 GLN 496 56.160 22.430 36.070 1.00 0.00 H

ATOM 4930 N GLN 497 58.460 28.020 38.480 1.00 0.00 N

ATOM 4931 CA GLN 497 58.140 29.440 38.240 1.00 0.00 C

ATOM 4932 C GLN 497 57.350 29.760 36.950 1.00 0.00 C

ATOM 4933 O GLN 497 57.670 30.680 36.200 1.00 0.00 O

ATOM 4934 CB GLN 497 59.410 30.300 38.380 1.00 0.00 C

ATOM 4935 CG GLN 497 60.090 30.150 39.740 1.00 0.00 C

ATOM 4936 CD GLN 497 61.340 31.010 39.870 1.00 0.00 C

ATOM 4937 NE2 GLN 497 62.450 30.380 40.190 1.00 0.00 N

ATOM 4938 OE1 GLN 497 61.310 32.230 39.770 1.00 0.00 O

ATOM 4939 H GLN 497 58.080 27.660 39.340 1.00 0.00 H

ATOM 4940 1HE2 GLN 497 62.440 29.390 40.340 1.00 0.00 H

ATOM 4941 2HE2 GLN 497 63.310 30.890 40.260 1.00 0.00 H

ATOM 4942 N GLU 498 56.260 29.010 36.770 1.00 0.00 N

ATOM 4943 CA GLU 498 55.250 29.280 35.710 1.00 0.00 C

ATOM 4944 C GLU 498 54.330 30.430 36.150 1.00 0.00 C

ATOM 4945 O GLU 498 53.760 30.430 37.250 1.00 0.00 O

ATOM 4946 CB GLU 498 54.370 28.070 35.410 1.00 0.00 C

ATOM 4947 CG GLU 498 55.110 26.850 34.850 1.00 0.00 C

ATOM 4948 CD GLU 498 54.210 25.610 34.730 1.00 0.00 C

ATOM 4949 OE1 GLU 498 53.170 25.680 34.060 1.00 0.00 O

ATOM 4950 OE2 GLU 498 54.520 24.600 35.400 1.00 0.00 O

ATOM 4951 H GLU 498 56.050 28.230 37.360 1.00 0.00 H

ATOM 4952 N TYR 499 54.220 31.420 35.270 1.00 0.00 N

ATOM 4953 CA TYR 499 53.330 32.580 35.430 1.00 0.00 C

ATOM 4954 C TYR 499 52.580 32.880 34.130 1.00 0.00 C

ATOM 4955 O TYR 499 53.030 32.520 33.040 1.00 0.00 O

ATOM 4956 CB TYR 499 54.120 33.800 35.910 1.00 0.00 C

ATOM 4957 CG TYR 499 55.050 34.410 34.860 1.00 0.00 C

ATOM 4958 CD1 TYR 499 56.320 33.870 34.660 1.00 0.00 C

ATOM 4959 CD2 TYR 499 54.610 35.470 34.080 1.00 0.00 C

ATOM 4960 CE1 TYR 499 57.140 34.410 33.680 1.00 0.00 C

ATOM 4961 CE2 TYR 499 55.440 36.010 33.100 1.00 0.00 C

ATOM 4962 CZ TYR 499 56.700 35.470 32.910 1.00 0.00 C

ATOM 4963 OH TYR 499 57.500 35.900 31.900 1.00 0.00 O

ATOM 4964 H TYR 499 54.750 31.400 34.410 1.00 0.00 H

ATOM 4965 HD1 TYR 499 56.650 33.000 35.220 1.00 0.00 H

ATOM 4966 HD2 TYR 499 53.620 35.890 34.240 1.00 0.00 H

ATOM 4967 HE1 TYR 499 58.110 33.960 33.480 1.00 0.00 H

ATOM 4968 HE2 TYR 499 55.070 36.840 32.490 1.00 0.00 H

ATOM 4969 HH TYR 499 57.090 36.730 31.480 1.00 0.00 H

ATOM 4970 N VAL 500 51.480 33.610 34.260 1.00 0.00 N

ATOM 4971 CA VAL 500 50.680 34.020 33.070 1.00 0.00 C

ATOM 4972 C VAL 500 50.750 35.530 32.820 1.00 0.00 C

ATOM 4973 O VAL 500 50.890 36.330 33.740 1.00 0.00 O

ATOM 4974 CB VAL 500 49.220 33.530 33.100 1.00 0.00 C

ATOM 4975 CG1 VAL 500 49.160 32.000 33.170 1.00 0.00 C

ATOM 4976 CG2 VAL 500 48.360 34.190 34.180 1.00 0.00 C

ATOM 4977 H VAL 500 51.170 33.970 35.140 1.00 0.00 H

ATOM 4978 N THR 501 50.690 35.870 31.540 1.00 0.00 N

ATOM 4979 CA THR 501 50.600 37.270 31.090 1.00 0.00 C

ATOM 4980 C THR 501 49.130 37.700 30.920 1.00 0.00 C

ATOM 4981 O THR 501 48.290 36.930 30.440 1.00 0.00 O

ATOM 4982 CB THR 501 51.400 37.530 29.810 1.00 0.00 C

ATOM 4983 CG2 THR 501 52.900 37.350 30.060 1.00 0.00 C

ATOM 4984 OG1 THR 501 50.980 36.680 28.730 1.00 0.00 O

ATOM 4985 H THR 501 50.780 35.190 30.810 1.00 0.00 H

ATOM 4986 HG1 THR 501 51.570 36.880 27.940 1.00 0.00 H

ATOM 4987 N LEU 502 48.850 38.890 31.430 1.00 0.00 N

ATOM 4988 CA LEU 502 47.510 39.510 31.360 1.00 0.00 C

ATOM 4989 C LEU 502 47.520 40.770 30.480 1.00 0.00 C

ATOM 4990 O LEU 502 47.990 41.840 30.850 1.00 0.00 O

ATOM 4991 CB LEU 502 46.900 39.830 32.740 1.00 0.00 C

ATOM 4992 CG LEU 502 46.540 38.610 33.610 1.00 0.00 C

ATOM 4993 CD1 LEU 502 45.840 39.110 34.870 1.00 0.00 C

ATOM 4994 CD2 LEU 502 45.630 37.610 32.900 1.00 0.00 C

ATOM 4995 H LEU 502 49.570 39.480 31.810 1.00 0.00 H

ATOM 4996 N ASN 503 46.990 40.530 29.290 1.00 0.00 N

ATOM 4997 CA ASN 503 46.980 41.480 28.160 1.00 0.00 C

ATOM 4998 C ASN 503 45.750 41.320 27.260 1.00 0.00 C

ATOM 4999 O ASN 503 45.050 40.300 27.310 1.00 0.00 O

ATOM 5000 CB ASN 503 48.250 41.270 27.320 1.00 0.00 C

ATOM 5001 CG ASN 503 48.420 39.840 26.810 1.00 0.00 C

ATOM 5002 ND2 ASN 503 49.530 39.250 27.170 1.00 0.00 N

ATOM 5003 OD1 ASN 503 47.540 39.240 26.200 1.00 0.00 O

ATOM 5004 H ASN 503 46.550 39.640 29.110 1.00 0.00 H

ATOM 5005 1HD2 ASN 503 50.170 39.750 27.780 1.00 0.00 H

ATOM 5006 2HD2 ASN 503 49.710 38.330 26.830 1.00 0.00 H

ATOM 5007 N TYR 504 45.540 42.290 26.370 1.00 0.00 N

ATOM 5008 CA TYR 504 44.420 42.230 25.410 1.00 0.00 C

ATOM 5009 C TYR 504 44.630 41.130 24.350 1.00 0.00 C

ATOM 5010 O TYR 504 43.770 40.270 24.210 1.00 0.00 O

ATOM 5011 CB TYR 504 44.100 43.600 24.790 1.00 0.00 C

ATOM 5012 CG TYR 504 44.750 43.910 23.430 1.00 0.00 C

ATOM 5013 CD1 TYR 504 45.980 44.550 23.360 1.00 0.00 C

ATOM 5014 CD2 TYR 504 44.230 43.320 22.280 1.00 0.00 C

ATOM 5015 CE1 TYR 504 46.720 44.530 22.190 1.00 0.00 C

ATOM 5016 CE2 TYR 504 44.950 43.320 21.100 1.00 0.00 C

ATOM 5017 CZ TYR 504 46.210 43.910 21.060 1.00 0.00 C

ATOM 5018 OH TYR 504 47.000 43.720 19.970 1.00 0.00 O

ATOM 5019 H TYR 504 46.190 43.050 26.270 1.00 0.00 H

ATOM 5020 HD1 TYR 504 46.290 45.200 24.180 1.00 0.00 H

ATOM 5021 HD2 TYR 504 43.360 42.680 22.380 1.00 0.00 H

ATOM 5022 HE1 TYR 504 47.700 45.000 22.160 1.00 0.00 H

ATOM 5023 HE2 TYR 504 44.560 42.800 20.220 1.00 0.00 H

ATOM 5024 HH TYR 504 46.550 44.170 19.170 1.00 0.00 H

ATOM 5025 N ASN 505 45.840 41.060 23.770 1.00 0.00 N

ATOM 5026 CA ASN 505 46.110 40.240 22.570 1.00 0.00 C

ATOM 5027 C ASN 505 46.010 38.730 22.820 1.00 0.00 C

ATOM 5028 O ASN 505 45.010 38.110 22.460 1.00 0.00 O

ATOM 5029 CB ASN 505 47.410 40.650 21.850 1.00 0.00 C

ATOM 5030 CG ASN 505 48.710 40.450 22.640 1.00 0.00 C

ATOM 5031 ND2 ASN 505 49.770 40.120 21.940 1.00 0.00 N

ATOM 5032 OD1 ASN 505 48.740 40.400 23.860 1.00 0.00 O

ATOM 5033 H ASN 505 46.650 41.480 24.190 1.00 0.00 H

ATOM 5034 1HD2 ASN 505 49.770 40.020 20.950 1.00 0.00 H

ATOM 5035 2HD2 ASN 505 50.570 39.790 22.460 1.00 0.00 H

ATOM 5036 N HIS 506 46.990 38.160 23.500 1.00 0.00 N

ATOM 5037 CA HIS 506 47.030 36.720 23.800 1.00 0.00 C

ATOM 5038 C HIS 506 47.720 36.460 25.150 1.00 0.00 C

ATOM 5039 O HIS 506 48.890 36.800 25.320 1.00 0.00 O

ATOM 5040 CB HIS 506 47.750 35.930 22.710 1.00 0.00 C

ATOM 5041 CG HIS 506 47.220 34.490 22.660 1.00 0.00 C

ATOM 5042 CD2 HIS 506 46.470 34.000 21.680 1.00 0.00 C

ATOM 5043 ND1 HIS 506 47.340 33.550 23.590 1.00 0.00 N

ATOM 5044 CE1 HIS 506 46.640 32.480 23.200 1.00 0.00 C

ATOM 5045 NE2 HIS 506 46.120 32.760 22.000 1.00 0.00 N

ATOM 5046 H HIS 506 47.670 38.720 24.000 1.00 0.00 H

ATOM 5047 HD1 HIS 506 47.850 33.620 24.460 1.00 0.00 H

ATOM 5048 N PRO 507 46.980 35.880 26.100 1.00 0.00 N

ATOM 5049 CA PRO 507 47.570 35.390 27.360 1.00 0.00 C

ATOM 5050 C PRO 507 48.530 34.220 27.090 1.00 0.00 C

ATOM 5051 O PRO 507 48.230 33.320 26.310 1.00 0.00 O

ATOM 5052 CB PRO 507 46.370 34.980 28.200 1.00 0.00 C

ATOM 5053 CG PRO 507 45.300 34.620 27.180 1.00 0.00 C

ATOM 5054 CD PRO 507 45.530 35.650 26.070 1.00 0.00 C

ATOM 5055 N GLU 508 49.700 34.330 27.700 1.00 0.00 N

ATOM 5056 CA GLU 508 50.800 33.350 27.540 1.00 0.00 C

ATOM 5057 C GLU 508 51.200 32.740 28.890 1.00 0.00 C

ATOM 5058 O GLU 508 51.010 33.360 29.940 1.00 0.00 O

ATOM 5059 CB GLU 508 52.040 34.050 26.990 1.00 0.00 C

ATOM 5060 CG GLU 508 51.890 34.640 25.580 1.00 0.00 C

ATOM 5061 CD GLU 508 53.190 35.390 25.250 1.00 0.00 C

ATOM 5062 OE1 GLU 508 53.240 36.610 25.530 1.00 0.00 O

ATOM 5063 OE2 GLU 508 54.160 34.710 24.860 1.00 0.00 O

ATOM 5064 H GLU 508 49.940 35.130 28.250 1.00 0.00 H

ATOM 5065 N LYS 509 51.770 31.550 28.820 1.00 0.00 N

ATOM 5066 CA LYS 509 52.410 30.900 29.980 1.00 0.00 C

ATOM 5067 C LYS 509 53.930 30.940 29.780 1.00 0.00 C

ATOM 5068 O LYS 509 54.470 30.470 28.780 1.00 0.00 O

ATOM 5069 CB LYS 509 52.020 29.440 30.210 1.00 0.00 C

ATOM 5070 CG LYS 509 50.550 29.260 30.560 1.00 0.00 C

ATOM 5071 CD LYS 509 49.750 29.100 29.280 1.00 0.00 C

ATOM 5072 CE LYS 509 48.270 29.160 29.590 1.00 0.00 C

ATOM 5073 NZ LYS 509 47.520 28.740 28.410 1.00 0.00 N1+

ATOM 5074 H LYS 509 51.880 31.070 27.950 1.00 0.00 H

ATOM 5075 HZ1 LYS 509 46.560 28.650 28.630 1.00 0.00 H

ATOM 5076 HZ2 LYS 509 47.640 29.380 27.650 1.00 0.00 H

ATOM 5077 HZ3 LYS 509 47.860 27.840 28.100 1.00 0.00 H

ATOM 5078 N LYS 510 54.530 31.690 30.690 1.00 0.00 N

ATOM 5079 CA LYS 510 55.980 31.930 30.680 1.00 0.00 C

ATOM 5080 C LYS 510 56.610 31.550 32.030 1.00 0.00 C

ATOM 5081 O LYS 510 55.920 31.390 33.030 1.00 0.00 O

ATOM 5082 CB LYS 510 56.240 33.400 30.330 1.00 0.00 C

ATOM 5083 CG LYS 510 55.940 33.720 28.860 1.00 0.00 C

ATOM 5084 CD LYS 510 56.260 35.180 28.570 1.00 0.00 C

ATOM 5085 CE LYS 510 56.060 35.490 27.090 1.00 0.00 C

ATOM 5086 NZ LYS 510 56.320 36.900 26.780 1.00 0.00 N1+

ATOM 5087 H LYS 510 54.050 32.020 31.500 1.00 0.00 H

ATOM 5088 HZ1 LYS 510 56.180 37.040 25.800 1.00 0.00 H

ATOM 5089 HZ2 LYS 510 55.690 37.480 27.280 1.00 0.00 H

ATOM 5090 HZ3 LYS 510 57.270 37.120 27.020 1.00 0.00 H

ATOM 5091 N MET 511 57.930 31.380 32.000 1.00 0.00 N

ATOM 5092 CA MET 511 58.690 30.890 33.170 1.00 0.00 C

ATOM 5093 C MET 511 59.830 31.810 33.590 1.00 0.00 C

ATOM 5094 O MET 511 60.490 32.440 32.770 1.00 0.00 O

ATOM 5095 CB MET 511 59.240 29.480 32.920 1.00 0.00 C

ATOM 5096 CG MET 511 58.110 28.460 32.850 1.00 0.00 C

ATOM 5097 SD MET 511 58.700 26.780 32.460 1.00 0.00 S

ATOM 5098 CE MET 511 57.120 26.110 31.990 1.00 0.00 C

ATOM 5099 H MET 511 58.490 31.680 31.230 1.00 0.00 H

ATOM 5100 N MET 512 59.980 31.880 34.910 1.00 0.00 N

ATOM 5101 CA MET 512 61.020 32.650 35.620 1.00 0.00 C

ATOM 5102 C MET 512 61.110 34.100 35.110 1.00 0.00 C

ATOM 5103 O MET 512 61.860 34.430 34.190 1.00 0.00 O

ATOM 5104 CB MET 512 62.380 31.940 35.560 1.00 0.00 C

ATOM 5105 CG MET 512 62.330 30.550 36.210 1.00 0.00 C

ATOM 5106 SD MET 512 63.930 29.670 36.230 1.00 0.00 S

ATOM 5107 CE MET 512 63.410 28.170 37.020 1.00 0.00 C

ATOM 5108 H MET 512 59.300 31.440 35.510 1.00 0.00 H

ATOM 5109 N ILE 513 60.180 34.900 35.610 1.00 0.00 N

ATOM 5110 CA ILE 513 60.110 36.360 35.340 1.00 0.00 C

ATOM 5111 C ILE 513 61.280 37.100 36.020 1.00 0.00 C

ATOM 5112 O ILE 513 61.660 36.820 37.160 1.00 0.00 O

ATOM 5113 CB ILE 513 58.720 36.960 35.660 1.00 0.00 C

ATOM 5114 CG1 ILE 513 58.590 38.410 35.190 1.00 0.00 C

ATOM 5115 CG2 ILE 513 58.360 36.890 37.150 1.00 0.00 C

ATOM 5116 CD ILE 513 58.610 38.570 33.660 1.00 0.00 C

ATOM 5117 H ILE 513 59.450 34.530 36.210 1.00 0.00 H

ATOM 5118 N LYS 514 61.980 37.850 35.170 1.00 0.00 N

ATOM 5119 CA LYS 514 63.190 38.630 35.480 1.00 0.00 C

ATOM 5120 C LYS 514 64.290 37.810 36.210 1.00 0.00 C

ATOM 5121 O LYS 514 64.960 38.340 37.100 1.00 0.00 O

ATOM 5122 CB LYS 514 62.830 39.900 36.240 1.00 0.00 C

ATOM 5123 CG LYS 514 61.990 40.830 35.370 1.00 0.00 C

ATOM 5124 CD LYS 514 61.640 42.070 36.180 1.00 0.00 C

ATOM 5125 CE LYS 514 60.620 42.950 35.470 1.00 0.00 C

ATOM 5126 NZ LYS 514 61.170 43.630 34.290 1.00 0.00 N1+

ATOM 5127 H LYS 514 61.740 37.830 34.190 1.00 0.00 H

ATOM 5128 HZ1 LYS 514 60.520 44.290 33.940 1.00 0.00 H

ATOM 5129 HZ2 LYS 514 61.990 44.130 34.570 1.00 0.00 H

ATOM 5130 HZ3 LYS 514 61.400 42.980 33.570 1.00 0.00 H

ATOM 5131 N PRO 515 64.590 36.590 35.730 1.00 0.00 N

ATOM 5132 CA PRO 515 65.420 35.630 36.470 1.00 0.00 C

ATOM 5133 C PRO 515 66.800 36.200 36.820 1.00 0.00 C

ATOM 5134 O PRO 515 67.050 36.430 38.000 1.00 0.00 O

ATOM 5135 CB PRO 515 65.450 34.390 35.580 1.00 0.00 C

ATOM 5136 CG PRO 515 65.320 34.930 34.160 1.00 0.00 C

ATOM 5137 CD PRO 515 64.400 36.130 34.340 1.00 0.00 C

ATOM 5138 N LYS 516 67.470 36.740 35.810 1.00 0.00 N

ATOM 5139 CA LYS 516 68.800 37.370 35.930 1.00 0.00 C

ATOM 5140 C LYS 516 68.800 38.560 36.900 1.00 0.00 C

ATOM 5141 O LYS 516 69.400 38.480 37.970 1.00 0.00 O

ATOM 5142 CB LYS 516 69.280 37.840 34.550 1.00 0.00 C

ATOM 5143 CG LYS 516 69.290 36.690 33.550 1.00 0.00 C

ATOM 5144 CD LYS 516 69.610 37.110 32.120 1.00 0.00 C

ATOM 5145 CE LYS 516 69.780 35.870 31.220 1.00 0.00 C

ATOM 5146 NZ LYS 516 70.900 35.020 31.640 1.00 0.00 N1+

ATOM 5147 H LYS 516 67.120 36.700 34.870 1.00 0.00 H

ATOM 5148 HZ1 LYS 516 70.970 34.230 31.030 1.00 0.00 H

ATOM 5149 HZ2 LYS 516 71.760 35.540 31.570 1.00 0.00 H

ATOM 5150 HZ3 LYS 516 70.780 34.720 32.590 1.00 0.00 H

ATOM 5151 N LYS 517 67.840 39.450 36.690 1.00 0.00 N

ATOM 5152 CA LYS 517 67.690 40.670 37.510 1.00 0.00 C

ATOM 5153 C LYS 517 67.420 40.380 39.000 1.00 0.00 C

ATOM 5154 O LYS 517 68.170 40.820 39.870 1.00 0.00 O

ATOM 5155 CB LYS 517 66.620 41.560 36.890 1.00 0.00 C

ATOM 5156 CG LYS 517 67.270 42.720 36.140 1.00 0.00 C

ATOM 5157 CD LYS 517 68.210 42.290 35.010 1.00 0.00 C

ATOM 5158 CE LYS 517 69.400 43.240 34.820 1.00 0.00 C

ATOM 5159 NZ LYS 517 68.960 44.620 34.640 1.00 0.00 N1+

ATOM 5160 H LYS 517 67.110 39.290 36.030 1.00 0.00 H

ATOM 5161 HZ1 LYS 517 69.680 45.150 34.190 1.00 0.00 H

ATOM 5162 HZ2 LYS 517 68.150 44.630 34.040 1.00 0.00 H

ATOM 5163 HZ3 LYS 517 68.730 45.020 35.530 1.00 0.00 H

ATOM 5164 N CYS 518 66.500 39.450 39.220 1.00 0.00 N

ATOM 5165 CA CYS 518 66.160 38.960 40.560 1.00 0.00 C

ATOM 5166 C CYS 518 67.280 38.160 41.250 1.00 0.00 C

ATOM 5167 O CYS 518 67.420 38.230 42.470 1.00 0.00 O

ATOM 5168 CB CYS 518 64.830 38.210 40.520 1.00 0.00 C

ATOM 5169 SG CYS 518 63.430 39.290 40.020 1.00 0.00 S

ATOM 5170 H CYS 518 65.970 39.050 38.470 1.00 0.00 H

ATOM 5171 N GLN 519 68.130 37.480 40.470 1.00 0.00 N

ATOM 5172 CA GLN 519 69.360 36.840 40.990 1.00 0.00 C

ATOM 5173 C GLN 519 70.240 37.870 41.720 1.00 0.00 C

ATOM 5174 O GLN 519 70.470 37.730 42.920 1.00 0.00 O

ATOM 5175 CB GLN 519 70.210 36.230 39.860 1.00 0.00 C

ATOM 5176 CG GLN 519 69.560 35.050 39.150 1.00 0.00 C

ATOM 5177 CD GLN 519 70.410 34.600 37.960 1.00 0.00 C

ATOM 5178 NE2 GLN 519 69.790 34.430 36.820 1.00 0.00 N

ATOM 5179 OE1 GLN 519 71.590 34.290 38.080 1.00 0.00 O

ATOM 5180 H GLN 519 67.960 37.350 39.490 1.00 0.00 H

ATOM 5181 1HE2 GLN 519 68.800 34.500 36.760 1.00 0.00 H

ATOM 5182 2HE2 GLN 519 70.360 34.300 36.000 1.00 0.00 H

ATOM 5183 N LEU 520 70.460 39.000 41.050 1.00 0.00 N

ATOM 5184 CA LEU 520 71.230 40.140 41.570 1.00 0.00 C

ATOM 5185 C LEU 520 70.590 40.780 42.820 1.00 0.00 C

ATOM 5186 O LEU 520 71.170 40.690 43.900 1.00 0.00 O

ATOM 5187 CB LEU 520 71.440 41.180 40.460 1.00 0.00 C

ATOM 5188 CG LEU 520 72.530 40.890 39.410 1.00 0.00 C

ATOM 5189 CD1 LEU 520 73.890 40.650 40.070 1.00 0.00 C

ATOM 5190 CD2 LEU 520 72.170 39.760 38.440 1.00 0.00 C

ATOM 5191 H LEU 520 70.120 39.120 40.110 1.00 0.00 H

ATOM 5192 N TRP 521 69.310 41.140 42.690 1.00 0.00 N

ATOM 5193 CA TRP 521 68.530 41.750 43.790 1.00 0.00 C

ATOM 5194 C TRP 521 68.390 40.890 45.050 1.00 0.00 C

ATOM 5195 O TRP 521 68.870 41.290 46.100 1.00 0.00 O

ATOM 5196 CB TRP 521 67.120 42.140 43.310 1.00 0.00 C

ATOM 5197 CG TRP 521 67.180 43.300 42.330 1.00 0.00 C

ATOM 5198 CD1 TRP 521 66.780 43.260 41.060 1.00 0.00 C

ATOM 5199 CD2 TRP 521 67.690 44.560 42.570 1.00 0.00 C

ATOM 5200 CE2 TRP 521 67.560 45.260 41.390 1.00 0.00 C

ATOM 5201 CE3 TRP 521 68.260 45.170 43.680 1.00 0.00 C

ATOM 5202 NE1 TRP 521 67.020 44.440 40.500 1.00 0.00 N

ATOM 5203 CZ2 TRP 521 67.980 46.580 41.300 1.00 0.00 C

ATOM 5204 CZ3 TRP 521 68.700 46.490 43.590 1.00 0.00 C

ATOM 5205 CH2 TRP 521 68.560 47.190 42.400 1.00 0.00 C

ATOM 5206 H TRP 521 68.860 41.160 41.800 1.00 0.00 H

ATOM 5207 HD1 TRP 521 66.340 42.410 40.560 1.00 0.00 H

ATOM 5208 HE1 TRP 521 66.890 44.670 39.520 1.00 0.00 H

ATOM 5209 HE3 TRP 521 68.320 44.640 44.630 1.00 0.00 H

ATOM 5210 HZ2 TRP 521 67.720 47.150 40.410 1.00 0.00 H

ATOM 5211 HZ3 TRP 521 68.980 47.040 44.480 1.00 0.00 H

ATOM 5212 HH2 TRP 521 68.810 48.250 42.370 1.00 0.00 H

ATOM 5213 N ASN 522 67.900 39.670 44.870 1.00 0.00 N

ATOM 5214 CA ASN 522 67.630 38.740 45.980 1.00 0.00 C

ATOM 5215 C ASN 522 68.910 38.170 46.610 1.00 0.00 C

ATOM 5216 O ASN 522 69.100 38.210 47.820 1.00 0.00 O

ATOM 5217 CB ASN 522 66.710 37.610 45.480 1.00 0.00 C

ATOM 5218 CG ASN 522 65.330 38.070 45.000 1.00 0.00 C

ATOM 5219 ND2 ASN 522 64.910 37.580 43.860 1.00 0.00 N

ATOM 5220 OD1 ASN 522 64.640 38.900 45.580 1.00 0.00 O

ATOM 5221 H ASN 522 67.680 39.330 43.950 1.00 0.00 H

ATOM 5222 1HD2 ASN 522 65.500 36.970 43.340 1.00 0.00 H

ATOM 5223 2HD2 ASN 522 64.120 38.050 43.470 1.00 0.00 H

ATOM 5224 N SER 523 69.800 37.650 45.760 1.00 0.00 N

ATOM 5225 CA SER 523 71.060 37.010 46.210 1.00 0.00 C

ATOM 5226 C SER 523 72.120 38.070 46.550 1.00 0.00 C

ATOM 5227 O SER 523 72.240 38.450 47.710 1.00 0.00 O

ATOM 5228 CB SER 523 71.610 36.000 45.200 1.00 0.00 C

ATOM 5229 OG SER 523 70.590 35.040 44.880 1.00 0.00 O

ATOM 5230 H SER 523 69.690 37.700 44.770 1.00 0.00 H

ATOM 5231 HG SER 523 70.930 34.390 44.180 1.00 0.00 H

ATOM 5232 N ILE 524 72.700 38.690 45.530 1.00 0.00 N

ATOM 5233 CA ILE 524 73.890 39.560 45.700 1.00 0.00 C

ATOM 5234 C ILE 524 73.620 40.820 46.530 1.00 0.00 C

ATOM 5235 O ILE 524 74.250 40.980 47.570 1.00 0.00 O

ATOM 5236 CB ILE 524 74.590 39.860 44.350 1.00 0.00 C

ATOM 5237 CG1 ILE 524 75.100 38.540 43.750 1.00 0.00 C

ATOM 5238 CG2 ILE 524 75.750 40.860 44.510 1.00 0.00 C

ATOM 5239 CD ILE 524 75.710 38.650 42.350 1.00 0.00 C

ATOM 5240 H ILE 524 72.260 38.750 44.630 1.00 0.00 H

ATOM 5241 N LEU 525 72.670 41.640 46.100 1.00 0.00 N

ATOM 5242 CA LEU 525 72.500 42.990 46.670 1.00 0.00 C

ATOM 5243 C LEU 525 72.420 43.070 48.210 1.00 0.00 C

ATOM 5244 O LEU 525 73.260 43.790 48.740 1.00 0.00 O

ATOM 5245 CB LEU 525 71.360 43.760 46.020 1.00 0.00 C

ATOM 5246 CG LEU 525 71.480 45.240 46.410 1.00 0.00 C

ATOM 5247 CD1 LEU 525 72.370 45.990 45.410 1.00 0.00 C

ATOM 5248 CD2 LEU 525 70.110 45.870 46.640 1.00 0.00 C

ATOM 5249 H LEU 525 72.010 41.380 45.380 1.00 0.00 H

ATOM 5250 N PRO 526 71.600 42.280 48.930 1.00 0.00 N

ATOM 5251 CA PRO 526 71.430 42.400 50.390 1.00 0.00 C

ATOM 5252 C PRO 526 72.720 42.330 51.220 1.00 0.00 C

ATOM 5253 O PRO 526 72.670 42.630 52.410 1.00 0.00 O

ATOM 5254 CB PRO 526 70.470 41.280 50.780 1.00 0.00 C

ATOM 5255 CG PRO 526 69.620 41.110 49.530 1.00 0.00 C

ATOM 5256 CD PRO 526 70.670 41.250 48.430 1.00 0.00 C

ATOM 5257 N GLN 527 73.790 41.710 50.700 1.00 0.00 N

ATOM 5258 CA GLN 527 75.100 41.760 51.380 1.00 0.00 C

ATOM 5259 C GLN 527 75.650 43.190 51.470 1.00 0.00 C

ATOM 5260 O GLN 527 75.890 43.660 52.580 1.00 0.00 O

ATOM 5261 CB GLN 527 76.110 40.830 50.700 1.00 0.00 C

ATOM 5262 CG GLN 527 77.340 40.590 51.580 1.00 0.00 C

ATOM 5263 CD GLN 527 78.150 39.390 51.100 1.00 0.00 C

ATOM 5264 NE2 GLN 527 78.510 39.350 49.840 1.00 0.00 N

ATOM 5265 OE1 GLN 527 78.400 38.450 51.840 1.00 0.00 O

ATOM 5266 H GLN 527 73.760 41.160 49.870 1.00 0.00 H

ATOM 5267 1HE2 GLN 527 78.290 40.110 49.220 1.00 0.00 H

ATOM 5268 2HE2 GLN 527 78.800 38.460 49.510 1.00 0.00 H

ATOM 5269 N ILE 528 75.730 43.870 50.340 1.00 0.00 N

ATOM 5270 CA ILE 528 76.260 45.250 50.300 1.00 0.00 C

ATOM 5271 C ILE 528 75.140 46.270 50.590 1.00 0.00 C

ATOM 5272 CB ILE 528 77.010 45.530 48.980 1.00 0.00 C

ATOM 5273 CG1 ILE 528 76.250 45.050 47.720 1.00 0.00 C

ATOM 5274 CG2 ILE 528 78.430 44.980 49.100 1.00 0.00 C

ATOM 5275 CD ILE 528 76.880 45.450 46.380 1.00 0.00 C

ATOM 5276 O1 ILE 528 74.010 46.060 50.100 1.00 0.00 O

ATOM 5277 O2 ILE 528 75.360 47.170 51.430 1.00 0.00 O

ATOM 5278 H ILE 528 75.180 43.640 49.540 1.00 0.00 H

HETATM 5279 OW SOL 529 51.260 30.990 38.390 1.00 0.00 O

HETATM 5280 HW1 SOL 529 52.180 30.800 38.020 1.00 0.00 H

HETATM 5281 HW2 SOL 529 50.690 30.180 38.180 1.00 0.00 H

HETATM 5282 OW SOL 530 55.180 53.060 37.420 1.00 0.00 O

HETATM 5283 HW1 SOL 530 55.590 52.460 38.130 1.00 0.00 H

HETATM 5284 HW2 SOL 530 55.290 52.570 36.540 1.00 0.00 H

HETATM 5285 OW SOL 531 44.770 54.340 48.200 1.00 0.00 O

HETATM 5286 HW1 SOL 531 45.670 54.490 48.620 1.00 0.00 H

HETATM 5287 HW2 SOL 531 44.920 53.590 47.530 1.00 0.00 H

HETATM 5288 OW SOL 532 54.660 50.960 63.060 1.00 0.00 O

HETATM 5289 HW1 SOL 532 54.770 51.820 63.580 1.00 0.00 H

HETATM 5290 HW2 SOL 532 55.120 51.110 62.170 1.00 0.00 H

HETATM 5291 OW SOL 533 41.000 49.750 51.580 1.00 0.00 O

HETATM 5292 HW1 SOL 533 40.630 50.280 52.350 1.00 0.00 H

HETATM 5293 HW2 SOL 533 40.650 48.800 51.590 1.00 0.00 H

HETATM 5294 OW SOL 534 45.980 50.480 46.130 1.00 0.00 O

HETATM 5295 HW1 SOL 534 45.500 51.190 45.600 1.00 0.00 H

HETATM 5296 HW2 SOL 534 46.830 50.240 45.580 1.00 0.00 H

HETATM 5297 OW SOL 535 48.910 52.820 52.740 1.00 0.00 O

HETATM 5298 HW1 SOL 535 49.190 53.630 53.250 1.00 0.00 H

HETATM 5299 HW2 SOL 535 48.220 53.140 52.080 1.00 0.00 H

HETATM 5300 OW SOL 536 49.850 44.090 39.630 1.00 0.00 O

HETATM 5301 HW1 SOL 536 50.480 44.580 40.280 1.00 0.00 H

HETATM 5302 HW2 SOL 536 49.620 44.810 38.960 1.00 0.00 H

HETATM 5303 OW SOL 537 47.200 61.990 49.820 1.00 0.00 O

HETATM 5304 HW1 SOL 537 48.120 62.080 50.220 1.00 0.00 H

HETATM 5305 HW2 SOL 537 47.170 61.130 49.290 1.00 0.00 H

HETATM 5306 OW SOL 538 55.130 26.710 38.220 1.00 0.00 O

HETATM 5307 HW1 SOL 538 54.160 26.610 38.520 1.00 0.00 H

HETATM 5308 HW2 SOL 538 55.120 26.440 37.240 1.00 0.00 H

HETATM 5309 OW SOL 539 44.510 59.520 65.450 1.00 0.00 O

HETATM 5310 HW1 SOL 539 43.940 60.250 65.880 1.00 0.00 H

HETATM 5311 HW2 SOL 539 44.000 59.270 64.580 1.00 0.00 H

HETATM 5312 OW SOL 540 41.840 57.160 54.460 1.00 0.00 O

HETATM 5313 HW1 SOL 540 42.200 57.490 53.580 1.00 0.00 H

HETATM 5314 HW2 SOL 540 41.870 56.160 54.390 1.00 0.00 H

HETATM 5315 OW SOL 541 50.250 48.260 40.860 1.00 0.00 O

HETATM 5316 HW1 SOL 541 50.180 48.590 41.830 1.00 0.00 H

HETATM 5317 HW2 SOL 541 49.340 47.830 40.650 1.00 0.00 H

HETATM 5318 OW SOL 542 36.720 38.830 57.890 1.00 0.00 O

HETATM 5319 HW1 SOL 542 37.360 38.100 58.160 1.00 0.00 H

HETATM 5320 HW2 SOL 542 37.260 39.540 57.400 1.00 0.00 H

HETATM 5321 OW SOL 543 56.800 43.790 46.570 1.00 0.00 O

HETATM 5322 HW1 SOL 543 57.730 44.190 46.590 1.00 0.00 H

HETATM 5323 HW2 SOL 543 56.630 43.500 45.630 1.00 0.00 H

HETATM 5324 OW SOL 544 46.790 50.220 32.330 1.00 0.00 O

HETATM 5325 HW1 SOL 544 46.330 49.960 33.190 1.00 0.00 H

HETATM 5326 HW2 SOL 544 47.530 50.880 32.530 1.00 0.00 H

HETATM 5327 OW SOL 545 36.230 50.500 48.100 1.00 0.00 O

HETATM 5328 HW1 SOL 545 35.430 50.930 47.660 1.00 0.00 H

HETATM 5329 HW2 SOL 545 36.840 50.270 47.330 1.00 0.00 H

HETATM 5330 OW SOL 546 34.980 55.350 63.420 1.00 0.00 O

HETATM 5331 HW1 SOL 546 34.190 55.980 63.340 1.00 0.00 H

HETATM 5332 HW2 SOL 546 34.940 54.670 62.670 1.00 0.00 H

HETATM 5333 OW SOL 547 57.010 53.420 61.710 1.00 0.00 O

HETATM 5334 HW1 SOL 547 57.910 53.550 62.150 1.00 0.00 H

HETATM 5335 HW2 SOL 547 56.300 53.530 62.420 1.00 0.00 H

HETATM 5336 OW SOL 548 60.870 46.300 35.510 1.00 0.00 O

HETATM 5337 HW1 SOL 548 61.530 45.920 36.210 1.00 0.00 H

HETATM 5338 HW2 SOL 548 61.340 47.050 35.030 1.00 0.00 H

HETATM 5339 OW SOL 549 46.360 58.730 59.390 1.00 0.00 O

HETATM 5340 HW1 SOL 549 47.370 58.840 59.480 1.00 0.00 H

HETATM 5341 HW2 SOL 549 46.130 59.080 58.470 1.00 0.00 H

HETATM 5342 OW SOL 550 40.220 57.220 41.740 1.00 0.00 O

HETATM 5343 HW1 SOL 550 39.840 58.010 42.270 1.00 0.00 H

HETATM 5344 HW2 SOL 550 40.030 57.430 40.750 1.00 0.00 H

HETATM 5345 OW SOL 551 33.890 46.520 41.610 1.00 0.00 O

HETATM 5346 HW1 SOL 551 33.400 47.320 41.960 1.00 0.00 H

HETATM 5347 HW2 SOL 551 34.420 46.780 40.790 1.00 0.00 H

HETATM 5348 OW SOL 552 49.210 60.790 43.230 1.00 0.00 O

HETATM 5349 HW1 SOL 552 49.970 61.450 43.230 1.00 0.00 H

HETATM 5350 HW2 SOL 552 49.480 59.970 42.730 1.00 0.00 H

HETATM 5351 OW SOL 553 58.610 33.560 37.440 1.00 0.00 O

HETATM 5352 HW1 SOL 553 59.130 33.190 38.220 1.00 0.00 H

HETATM 5353 HW2 SOL 553 57.720 33.920 37.820 1.00 0.00 H

HETATM 5354 OW SOL 554 51.960 26.440 44.070 1.00 0.00 O

HETATM 5355 HW1 SOL 554 52.180 26.400 45.050 1.00 0.00 H

HETATM 5356 HW2 SOL 554 52.700 26.980 43.610 1.00 0.00 H

HETATM 5357 OW SOL 555 42.840 47.950 23.550 1.00 0.00 O

HETATM 5358 HW1 SOL 555 43.400 48.400 24.260 1.00 0.00 H

HETATM 5359 HW2 SOL 555 43.340 47.140 23.220 1.00 0.00 H

HETATM 5360 OW SOL 556 67.030 43.780 47.100 1.00 0.00 O

HETATM 5361 HW1 SOL 556 66.680 44.040 48.040 1.00 0.00 H

HETATM 5362 HW2 SOL 556 67.750 44.420 46.880 1.00 0.00 H

HETATM 5363 OW SOL 557 43.750 51.570 38.060 1.00 0.00 O

HETATM 5364 HW1 SOL 557 43.260 52.410 38.320 1.00 0.00 H

HETATM 5365 HW2 SOL 557 44.220 51.790 37.190 1.00 0.00 H

HETATM 5366 OW SOL 558 33.270 60.230 58.290 1.00 0.00 O

HETATM 5367 HW1 SOL 558 33.280 61.240 58.350 1.00 0.00 H

HETATM 5368 HW2 SOL 558 32.420 59.970 57.840 1.00 0.00 H

HETATM 5369 OW SOL 559 43.320 58.900 63.150 1.00 0.00 O

HETATM 5370 HW1 SOL 559 43.670 59.530 62.420 1.00 0.00 H

HETATM 5371 HW2 SOL 559 42.300 58.890 63.060 1.00 0.00 H

HETATM 5372 OW SOL 560 64.470 43.380 46.430 1.00 0.00 O

HETATM 5373 HW1 SOL 560 65.490 43.440 46.590 1.00 0.00 H

HETATM 5374 HW2 SOL 560 64.190 44.230 45.960 1.00 0.00 H

HETATM 5375 OW SOL 561 42.680 30.520 33.480 1.00 0.00 O

HETATM 5376 HW1 SOL 561 43.440 31.020 33.890 1.00 0.00 H

HETATM 5377 HW2 SOL 561 42.860 30.410 32.500 1.00 0.00 H

HETATM 5378 OW SOL 562 51.580 47.390 68.320 1.00 0.00 O

HETATM 5379 HW1 SOL 562 50.950 46.750 67.850 1.00 0.00 H

HETATM 5380 HW2 SOL 562 51.920 48.000 67.590 1.00 0.00 H

HETATM 5381 OW SOL 563 37.150 56.940 63.390 1.00 0.00 O

HETATM 5382 HW1 SOL 563 36.610 57.810 63.490 1.00 0.00 H

HETATM 5383 HW2 SOL 563 36.430 56.220 63.510 1.00 0.00 H

HETATM 5384 OW SOL 564 40.250 53.610 68.560 1.00 0.00 O

HETATM 5385 HW1 SOL 564 39.810 54.510 68.650 1.00 0.00 H

HETATM 5386 HW2 SOL 564 40.020 53.310 67.630 1.00 0.00 H

HETATM 5387 OW SOL 565 37.530 33.390 50.280 1.00 0.00 O

HETATM 5388 HW1 SOL 565 38.240 34.000 49.880 1.00 0.00 H

HETATM 5389 HW2 SOL 565 36.680 33.920 50.140 1.00 0.00 H

HETATM 5390 OW SOL 566 62.890 34.790 38.530 1.00 0.00 O

HETATM 5391 HW1 SOL 566 63.740 35.300 38.660 1.00 0.00 H

HETATM 5392 HW2 SOL 566 62.220 35.430 38.090 1.00 0.00 H

HETATM 5393 OW SOL 567 60.450 34.900 31.200 1.00 0.00 O

HETATM 5394 HW1 SOL 567 60.870 34.410 31.970 1.00 0.00 H

HETATM 5395 HW2 SOL 567 59.740 34.280 30.830 1.00 0.00 H

HETATM 5396 OW SOL 568 46.250 57.680 65.910 1.00 0.00 O

HETATM 5397 HW1 SOL 568 45.510 58.420 65.810 1.00 0.00 H

HETATM 5398 HW2 SOL 568 46.900 57.850 65.160 1.00 0.00 H

HETATM 5399 OW SOL 569 45.910 56.690 46.650 1.00 0.00 O

HETATM 5400 HW1 SOL 569 45.010 56.880 47.060 1.00 0.00 H

HETATM 5401 HW2 SOL 569 45.920 55.730 46.300 1.00 0.00 H

HETATM 5402 OW SOL 570 49.630 66.380 41.990 1.00 0.00 O

HETATM 5403 HW1 SOL 570 48.780 66.520 41.470 1.00 0.00 H

HETATM 5404 HW2 SOL 570 49.690 65.400 42.250 1.00 0.00 H

HETATM 5405 OW SOL 571 42.970 64.120 68.780 1.00 0.00 O

HETATM 5406 HW1 SOL 571 43.930 64.050 69.130 1.00 0.00 H

HETATM 5407 HW2 SOL 571 42.720 65.090 68.800 1.00 0.00 H

HETATM 5408 OW SOL 572 64.840 52.320 45.260 1.00 0.00 O

HETATM 5409 HW1 SOL 572 65.830 52.190 45.370 1.00 0.00 H

HETATM 5410 HW2 SOL 572 64.680 52.810 44.400 1.00 0.00 H

HETATM 5411 OW SOL 573 20.780 37.120 52.860 1.00 0.00 O

HETATM 5412 HW1 SOL 573 21.170 36.820 53.730 1.00 0.00 H

HETATM 5413 HW2 SOL 573 21.190 36.570 52.120 1.00 0.00 H

HETATM 5414 OW SOL 574 49.960 68.150 51.540 1.00 0.00 O

HETATM 5415 HW1 SOL 574 50.040 68.130 52.540 1.00 0.00 H

HETATM 5416 HW2 SOL 574 50.890 68.360 51.200 1.00 0.00 H

HETATM 5417 OW SOL 575 23.830 34.650 56.480 1.00 0.00 O

HETATM 5418 HW1 SOL 575 22.840 34.860 56.510 1.00 0.00 H

HETATM 5419 HW2 SOL 575 23.950 34.480 55.490 1.00 0.00 H

HETATM 5420 OW SOL 576 45.430 38.080 28.900 1.00 0.00 O

HETATM 5421 HW1 SOL 576 45.260 38.600 28.040 1.00 0.00 H

HETATM 5422 HW2 SOL 576 45.910 37.230 28.660 1.00 0.00 H

HETATM 5423 OW SOL 577 44.730 66.000 33.710 1.00 0.00 O

HETATM 5424 HW1 SOL 577 45.230 66.830 33.400 1.00 0.00 H

HETATM 5425 HW2 SOL 577 43.790 66.320 33.870 1.00 0.00 H

HETATM 5426 OW SOL 578 60.230 29.630 52.860 1.00 0.00 O

HETATM 5427 HW1 SOL 578 60.030 29.310 53.800 1.00 0.00 H

HETATM 5428 HW2 SOL 578 59.360 29.930 52.460 1.00 0.00 H

HETATM 5429 OW SOL 579 59.570 48.730 36.460 1.00 0.00 O

HETATM 5430 HW1 SOL 579 60.460 49.150 36.240 1.00 0.00 H

HETATM 5431 HW2 SOL 579 59.520 47.860 35.930 1.00 0.00 H

HETATM 5432 OW SOL 580 41.060 30.370 56.890 1.00 0.00 O

HETATM 5433 HW1 SOL 580 40.040 30.330 56.980 1.00 0.00 H

HETATM 5434 HW2 SOL 580 41.180 31.220 56.370 1.00 0.00 H

HETATM 5435 OW SOL 581 38.610 59.110 42.970 1.00 0.00 O

HETATM 5436 HW1 SOL 581 38.000 59.580 43.630 1.00 0.00 H

HETATM 5437 HW2 SOL 581 38.290 59.430 42.060 1.00 0.00 H

HETATM 5438 OW SOL 582 58.070 55.440 64.260 1.00 0.00 O

HETATM 5439 HW1 SOL 582 58.270 56.420 64.170 1.00 0.00 H

HETATM 5440 HW2 SOL 582 58.590 55.020 63.490 1.00 0.00 H

HETATM 5441 OW SOL 583 48.810 58.420 47.480 1.00 0.00 O

HETATM 5442 HW1 SOL 583 49.350 58.000 46.740 1.00 0.00 H

HETATM 5443 HW2 SOL 583 47.920 57.940 47.460 1.00 0.00 H

HETATM 5444 OW SOL 584 21.180 43.990 48.560 1.00 0.00 O

HETATM 5445 HW1 SOL 584 21.870 43.270 48.420 1.00 0.00 H

HETATM 5446 HW2 SOL 584 20.860 44.230 47.630 1.00 0.00 H

HETATM 5447 OW SOL 585 46.040 63.150 75.470 1.00 0.00 O

HETATM 5448 HW1 SOL 585 46.630 63.970 75.570 1.00 0.00 H

HETATM 5449 HW2 SOL 585 46.580 62.490 74.930 1.00 0.00 H

HETATM 5450 OW SOL 586 62.050 64.300 38.680 1.00 0.00 O

HETATM 5451 HW1 SOL 586 61.040 64.240 38.720 1.00 0.00 H

HETATM 5452 HW2 SOL 586 62.290 64.890 37.900 1.00 0.00 H

HETATM 5453 OW SOL 587 53.790 64.710 45.730 1.00 0.00 O

HETATM 5454 HW1 SOL 587 53.320 64.080 46.380 1.00 0.00 H

HETATM 5455 HW2 SOL 587 53.460 64.380 44.820 1.00 0.00 H

HETATM 5456 OW SOL 588 57.180 37.980 30.390 1.00 0.00 O

HETATM 5457 HW1 SOL 588 57.250 38.970 30.290 1.00 0.00 H

HETATM 5458 HW2 SOL 588 58.060 37.570 30.100 1.00 0.00 H

HETATM 5459 OW SOL 589 55.590 47.350 70.550 1.00 0.00 O

HETATM 5460 HW1 SOL 589 56.080 46.500 70.280 1.00 0.00 H

HETATM 5461 HW2 SOL 589 54.710 47.300 70.050 1.00 0.00 H

HETATM 5462 OW SOL 590 30.720 43.920 65.530 1.00 0.00 O

HETATM 5463 HW1 SOL 590 31.690 43.640 65.730 1.00 0.00 H

HETATM 5464 HW2 SOL 590 30.710 44.900 65.400 1.00 0.00 H

HETATM 5465 OW SOL 591 36.780 59.090 70.620 1.00 0.00 O

HETATM 5466 HW1 SOL 591 37.670 59.120 70.170 1.00 0.00 H

HETATM 5467 HW2 SOL 591 36.320 58.240 70.280 1.00 0.00 H

HETATM 5468 OW SOL 592 43.020 46.930 69.000 1.00 0.00 O

HETATM 5469 HW1 SOL 592 42.460 47.710 69.360 1.00 0.00 H

HETATM 5470 HW2 SOL 592 43.620 47.310 68.290 1.00 0.00 H

HETATM 5471 OW SOL 593 42.950 45.460 66.150 1.00 0.00 O

HETATM 5472 HW1 SOL 593 42.220 45.840 66.730 1.00 0.00 H

HETATM 5473 HW2 SOL 593 42.790 45.730 65.190 1.00 0.00 H

HETATM 5474 OW SOL 594 29.680 59.470 49.020 1.00 0.00 O

HETATM 5475 HW1 SOL 594 29.780 60.440 48.760 1.00 0.00 H

HETATM 5476 HW2 SOL 594 29.540 58.950 48.150 1.00 0.00 H

HETATM 5477 OW SOL 595 34.120 63.200 54.540 1.00 0.00 O

HETATM 5478 HW1 SOL 595 34.660 63.130 55.380 1.00 0.00 H

HETATM 5479 HW2 SOL 595 33.470 63.970 54.660 1.00 0.00 H

HETATM 5480 OW SOL 596 44.520 60.290 60.960 1.00 0.00 O

HETATM 5481 HW1 SOL 596 45.100 59.500 60.740 1.00 0.00 H

HETATM 5482 HW2 SOL 596 44.940 61.070 60.500 1.00 0.00 H

HETATM 5483 OW SOL 597 40.480 64.880 63.000 1.00 0.00 O

HETATM 5484 HW1 SOL 597 41.210 65.500 63.350 1.00 0.00 H

HETATM 5485 HW2 SOL 597 40.010 65.350 62.250 1.00 0.00 H

HETATM 5486 OW SOL 598 50.700 61.620 55.190 1.00 0.00 O

HETATM 5487 HW1 SOL 598 50.160 60.780 55.340 1.00 0.00 H

HETATM 5488 HW2 SOL 598 51.390 61.390 54.510 1.00 0.00 H

HETATM 5489 OW SOL 599 39.550 57.680 39.210 1.00 0.00 O

HETATM 5490 HW1 SOL 599 38.770 58.290 38.960 1.00 0.00 H

HETATM 5491 HW2 SOL 599 40.130 57.670 38.370 1.00 0.00 H

HETATM 5492 OW SOL 600 41.800 65.790 65.630 1.00 0.00 O

HETATM 5493 HW1 SOL 600 42.210 66.440 64.970 1.00 0.00 H

HETATM 5494 HW2 SOL 600 42.320 64.920 65.550 1.00 0.00 H

HETATM 5495 OW SOL 601 32.550 28.810 47.900 1.00 0.00 O

HETATM 5496 HW1 SOL 601 33.340 29.380 48.160 1.00 0.00 H

HETATM 5497 HW2 SOL 601 31.700 29.350 48.020 1.00 0.00 H

HETATM 5498 OW SOL 602 28.980 32.640 42.100 1.00 0.00 O

HETATM 5499 HW1 SOL 602 28.580 32.550 43.030 1.00 0.00 H

HETATM 5500 HW2 SOL 602 28.540 33.490 41.760 1.00 0.00 H

HETATM 5501 OW SOL 603 29.420 37.050 59.900 1.00 0.00 O

HETATM 5502 HW1 SOL 603 30.050 37.240 60.670 1.00 0.00 H

HETATM 5503 HW2 SOL 603 29.460 36.050 59.760 1.00 0.00 H

HETATM 5504 OW SOL 604 47.670 27.890 58.540 1.00 0.00 O

HETATM 5505 HW1 SOL 604 47.660 27.060 59.140 1.00 0.00 H

HETATM 5506 HW2 SOL 604 47.290 28.640 59.080 1.00 0.00 H

HETATM 5507 OW SOL 605 45.440 64.820 73.100 1.00 0.00 O

HETATM 5508 HW1 SOL 605 44.960 64.890 73.980 1.00 0.00 H

HETATM 5509 HW2 SOL 605 45.170 63.970 72.660 1.00 0.00 H

HETATM 5510 OW SOL 606 58.430 65.890 36.690 1.00 0.00 O

HETATM 5511 HW1 SOL 606 59.040 66.630 37.000 1.00 0.00 H

HETATM 5512 HW2 SOL 606 58.610 65.100 37.300 1.00 0.00 H

HETATM 5513 OW SOL 607 48.930 47.480 26.320 1.00 0.00 O

HETATM 5514 HW1 SOL 607 49.690 46.830 26.390 1.00 0.00 H

HETATM 5515 HW2 SOL 607 48.040 47.030 26.510 1.00 0.00 H

HETATM 5516 OW SOL 608 37.360 49.890 20.740 1.00 0.00 O

HETATM 5517 HW1 SOL 608 37.510 50.540 21.490 1.00 0.00 H

HETATM 5518 HW2 SOL 608 36.540 50.250 20.220 1.00 0.00 H

HETATM 5519 OW SOL 609 29.520 38.030 31.290 1.00 0.00 O

HETATM 5520 HW1 SOL 609 30.200 37.730 31.970 1.00 0.00 H

HETATM 5521 HW2 SOL 609 29.630 37.300 30.580 1.00 0.00 H

HETATM 5522 OW SOL 610 47.480 44.270 26.100 1.00 0.00 O

HETATM 5523 HW1 SOL 610 46.950 44.950 26.650 1.00 0.00 H

HETATM 5524 HW2 SOL 610 48.060 44.820 25.490 1.00 0.00 H

HETATM 5525 OW SOL 611 18.170 54.070 47.710 1.00 0.00 O

HETATM 5526 HW1 SOL 611 17.570 53.600 48.350 1.00 0.00 H

HETATM 5527 HW2 SOL 611 18.000 53.710 46.780 1.00 0.00 H

HETATM 5528 OW SOL 612 47.650 50.730 71.870 1.00 0.00 O

HETATM 5529 HW1 SOL 612 48.410 51.320 71.560 1.00 0.00 H

HETATM 5530 HW2 SOL 612 48.040 49.830 72.100 1.00 0.00 H

HETATM 5531 OW SOL 613 47.320 64.240 29.750 1.00 0.00 O

HETATM 5532 HW1 SOL 613 48.250 63.940 30.000 1.00 0.00 H

HETATM 5533 HW2 SOL 613 46.710 63.770 30.410 1.00 0.00 H

HETATM 5534 OW SOL 614 67.160 46.770 51.030 1.00 0.00 O

HETATM 5535 HW2 SOL 614 67.720 47.120 50.240 1.00 0.00 H

HETATM 5536 OW SOL 615 66.920 44.520 32.610 1.00 0.00 O

HETATM 5537 HW1 SOL 615 66.980 45.330 32.010 1.00 0.00 H

HETATM 5538 HW2 SOL 615 66.050 44.050 32.380 1.00 0.00 H

HETATM 5539 OW SOL 616 43.830 25.900 44.350 1.00 0.00 O

HETATM 5540 HW1 SOL 616 43.250 26.240 45.110 1.00 0.00 H

HETATM 5541 HW2 SOL 616 44.010 26.720 43.790 1.00 0.00 H

HETATM 5542 HW1 SOL 617 44.780 22.700 30.730 1.00 0.00 H

HETATM 5543 OW SOL 618 45.390 63.870 69.950 1.00 0.00 O

HETATM 5544 HW1 SOL 618 45.950 64.630 70.350 1.00 0.00 H

HETATM 5545 HW2 SOL 618 45.860 63.000 70.110 1.00 0.00 H

HETATM 5546 OW SOL 619 47.080 67.530 54.450 1.00 0.00 O

HETATM 5547 HW1 SOL 619 47.430 67.150 55.310 1.00 0.00 H

HETATM 5548 HW2 SOL 619 46.080 67.440 54.410 1.00 0.00 H

HETATM 5549 OW SOL 620 50.600 70.270 36.770 1.00 0.00 O

HETATM 5550 HW1 SOL 620 51.330 70.740 37.300 1.00 0.00 H

HETATM 5551 HW2 SOL 620 50.080 71.010 36.330 1.00 0.00 H

HETATM 5552 OW SOL 621 20.640 50.200 66.280 1.00 0.00 O

HETATM 5553 HW1 SOL 621 21.390 50.640 66.820 1.00 0.00 H

HETATM 5554 HW2 SOL 621 20.360 49.380 66.780 1.00 0.00 H

HETATM 5555 OW SOL 622 32.500 56.980 64.170 1.00 0.00 O

HETATM 5556 HW1 SOL 622 31.770 57.120 64.850 1.00 0.00 H

HETATM 5557 HW2 SOL 622 33.210 57.660 64.390 1.00 0.00 H

HETATM 5558 OW SOL 623 38.620 59.790 72.800 1.00 0.00 O

HETATM 5559 HW1 SOL 623 37.790 60.060 72.280 1.00 0.00 H

HETATM 5560 HW2 SOL 623 39.180 59.260 72.170 1.00 0.00 H

HETATM 5561 OW SOL 624 44.760 67.960 66.890 1.00 0.00 O

HETATM 5562 HW1 SOL 624 44.570 68.940 67.000 1.00 0.00 H

HETATM 5563 HW2 SOL 624 43.890 67.460 66.850 1.00 0.00 H

HETATM 5564 OW SOL 625 51.430 61.260 47.770 1.00 0.00 O

HETATM 5565 HW1 SOL 625 52.190 60.620 47.890 1.00 0.00 H

HETATM 5566 HW2 SOL 625 50.580 60.730 47.650 1.00 0.00 H

HETATM 5567 OW SOL 626 42.550 66.010 47.310 1.00 0.00 O

HETATM 5568 HW1 SOL 626 43.440 66.060 47.810 1.00 0.00 H

HETATM 5569 HW2 SOL 626 42.840 66.290 46.370 1.00 0.00 H

HETATM 5570 OW SOL 627 40.650 61.340 44.000 1.00 0.00 O

HETATM 5571 HW1 SOL 627 41.360 61.350 44.730 1.00 0.00 H

HETATM 5572 HW2 SOL 627 40.710 60.430 43.560 1.00 0.00 H

HETATM 5573 OW SOL 628 43.990 58.710 48.920 1.00 0.00 O

HETATM 5574 HW1 SOL 628 43.300 59.360 49.250 1.00 0.00 H

HETATM 5575 HW2 SOL 628 44.290 59.010 48.000 1.00 0.00 H

HETATM 5576 OW SOL 629 35.890 61.170 43.380 1.00 0.00 O

HETATM 5577 HW1 SOL 629 35.000 61.620 43.290 1.00 0.00 H

HETATM 5578 HW2 SOL 629 35.770 60.250 42.920 1.00 0.00 H

HETATM 5579 OW SOL 630 32.650 34.470 62.350 1.00 0.00 O

HETATM 5580 HW1 SOL 630 32.190 35.320 62.050 1.00 0.00 H

HETATM 5581 HW2 SOL 630 32.220 33.710 61.860 1.00 0.00 H

HETATM 5582 OW SOL 631 36.380 27.550 49.480 1.00 0.00 O

HETATM 5583 HW1 SOL 631 37.140 26.950 49.760 1.00 0.00 H

HETATM 5584 HW2 SOL 631 35.700 26.970 49.010 1.00 0.00 H

HETATM 5585 OW SOL 632 57.520 60.350 45.670 1.00 0.00 O

HETATM 5586 HW1 SOL 632 57.540 59.380 45.960 1.00 0.00 H

HETATM 5587 HW2 SOL 632 56.810 60.350 44.950 1.00 0.00 H

HETATM 5588 OW SOL 633 59.380 54.870 61.920 1.00 0.00 O

HETATM 5589 HW1 SOL 633 60.320 55.240 61.950 1.00 0.00 H

HETATM 5590 HW2 SOL 633 59.080 54.880 60.960 1.00 0.00 H

HETATM 5591 OW SOL 634 60.160 49.850 33.640 1.00 0.00 O

HETATM 5592 HW1 SOL 634 59.240 49.450 33.690 1.00 0.00 H

HETATM 5593 HW2 SOL 634 60.040 50.620 32.980 1.00 0.00 H

HETATM 5594 OW SOL 635 55.600 63.160 26.900 1.00 0.00 O

HETATM 5595 HW1 SOL 635 55.200 63.430 27.800 1.00 0.00 H

HETATM 5596 HW2 SOL 635 54.890 62.700 26.380 1.00 0.00 H

HETATM 5597 OW SOL 636 53.310 50.680 27.400 1.00 0.00 O

HETATM 5598 HW1 SOL 636 53.280 51.540 26.860 1.00 0.00 H

HETATM 5599 HW2 SOL 636 53.060 49.930 26.780 1.00 0.00 H

HETATM 5600 OW SOL 637 52.710 23.960 37.570 1.00 0.00 O

HETATM 5601 HW1 SOL 637 53.410 24.320 36.920 1.00 0.00 H

HETATM 5602 HW2 SOL 637 52.340 24.740 38.090 1.00 0.00 H

HETATM 5603 OW SOL 638 47.610 68.250 43.330 1.00 0.00 O

HETATM 5604 HW1 SOL 638 48.550 68.030 43.060 1.00 0.00 H

HETATM 5605 HW2 SOL 638 47.190 68.860 42.650 1.00 0.00 H

HETATM 5606 OW SOL 639 44.110 66.550 44.940 1.00 0.00 O

HETATM 5607 HW1 SOL 639 45.110 66.560 45.090 1.00 0.00 H

HETATM 5608 HW2 SOL 639 43.930 66.700 43.960 1.00 0.00 H

HETATM 5609 OW SOL 640 23.680 41.610 39.580 1.00 0.00 O

HETATM 5610 HW1 SOL 640 23.340 41.580 40.540 1.00 0.00 H

HETATM 5611 HW2 SOL 640 24.680 41.510 39.660 1.00 0.00 H

HETATM 5612 OW SOL 641 56.020 42.220 27.820 1.00 0.00 O

HETATM 5613 HW1 SOL 641 55.180 42.040 28.340 1.00 0.00 H

HETATM 5614 HW2 SOL 641 55.890 41.780 26.910 1.00 0.00 H

HETATM 5615 OW SOL 642 32.010 59.890 64.440 1.00 0.00 O

HETATM 5616 HW1 SOL 642 31.680 60.840 64.380 1.00 0.00 H

HETATM 5617 HW2 SOL 642 32.970 59.940 64.060 1.00 0.00 H

HETATM 5618 OW SOL 643 54.420 71.640 43.400 1.00 0.00 O

HETATM 5619 HW1 SOL 643 55.030 71.170 42.750 1.00 0.00 H

HETATM 5620 HW2 SOL 643 53.500 71.630 42.960 1.00 0.00 H

HETATM 5621 OW SOL 644 36.070 66.920 58.150 1.00 0.00 O

HETATM 5622 HW1 SOL 644 37.000 67.080 58.490 1.00 0.00 H

HETATM 5623 HW2 SOL 644 35.720 67.720 57.640 1.00 0.00 H

HETATM 5624 OW SOL 645 38.540 32.880 58.590 1.00 0.00 O

HETATM 5625 HW1 SOL 645 38.460 33.190 59.560 1.00 0.00 H

HETATM 5626 HW2 SOL 645 38.250 31.910 58.560 1.00 0.00 H

HETATM 5627 OW SOL 646 42.850 27.960 52.470 1.00 0.00 O

HETATM 5628 HW1 SOL 646 41.990 28.070 52.990 1.00 0.00 H

HETATM 5629 HW2 SOL 646 42.640 27.900 51.480 1.00 0.00 H

HETATM 5630 OW SOL 647 63.650 44.480 30.340 1.00 0.00 O

HETATM 5631 HW1 SOL 647 63.260 44.910 31.190 1.00 0.00 H

HETATM 5632 HW2 SOL 647 63.090 44.900 29.600 1.00 0.00 H

HETATM 5633 OW SOL 648 51.810 20.020 49.120 1.00 0.00 O

HETATM 5634 HW1 SOL 648 51.740 20.920 48.670 1.00 0.00 H

HETATM 5635 HW2 SOL 648 51.790 19.330 48.380 1.00 0.00 H

HETATM 5636 OW SOL 649 33.810 57.570 45.060 1.00 0.00 O

HETATM 5637 HW1 SOL 649 34.610 57.690 44.430 1.00 0.00 H

HETATM 5638 HW2 SOL 649 34.180 57.100 45.870 1.00 0.00 H

HETATM 5639 OW SOL 650 26.550 32.680 56.380 1.00 0.00 O

HETATM 5640 HW1 SOL 650 26.650 32.630 57.390 1.00 0.00 H

HETATM 5641 HW2 SOL 650 26.540 33.660 56.130 1.00 0.00 H

HETATM 5642 OW SOL 651 37.540 33.460 56.070 1.00 0.00 O

HETATM 5643 HW1 SOL 651 37.930 33.530 57.010 1.00 0.00 H

HETATM 5644 HW2 SOL 651 36.540 33.600 56.210 1.00 0.00 H

HETATM 5645 OW SOL 652 62.740 28.200 52.240 1.00 0.00 O

HETATM 5646 HW1 SOL 652 62.030 28.380 52.950 1.00 0.00 H

HETATM 5647 HW2 SOL 652 62.210 27.810 51.450 1.00 0.00 H

HETATM 5648 OW SOL 653 59.800 46.560 31.620 1.00 0.00 O

HETATM 5649 HW1 SOL 653 59.220 47.400 31.810 1.00 0.00 H

HETATM 5650 HW2 SOL 653 59.160 45.810 31.770 1.00 0.00 H

HETATM 5651 OW SOL 654 62.000 48.430 29.520 1.00 0.00 O

HETATM 5652 HW1 SOL 654 62.180 49.420 29.420 1.00 0.00 H

HETATM 5653 HW2 SOL 654 61.050 48.370 29.840 1.00 0.00 H

HETATM 5654 OW SOL 655 47.590 54.500 26.460 1.00 0.00 O

HETATM 5655 HW1 SOL 655 48.190 54.100 27.140 1.00 0.00 H

HETATM 5656 HW2 SOL 655 46.660 54.390 26.820 1.00 0.00 H

HETATM 5657 OW SOL 656 40.880 41.090 25.220 1.00 0.00 O

HETATM 5658 HW1 SOL 656 41.760 41.050 25.720 1.00 0.00 H

HETATM 5659 HW2 SOL 656 40.770 42.020 24.870 1.00 0.00 H

HETATM 5660 OW SOL 657 45.410 24.890 39.470 1.00 0.00 O

HETATM 5661 HW1 SOL 657 45.430 24.970 40.470 1.00 0.00 H

HETATM 5662 HW2 SOL 657 44.940 24.010 39.290 1.00 0.00 H

HETATM 5663 OW SOL 658 38.450 30.830 56.470 1.00 0.00 O

HETATM 5664 HW1 SOL 658 37.590 30.450 56.840 1.00 0.00 H

HETATM 5665 HW2 SOL 658 38.170 31.740 56.060 1.00 0.00 H

HETATM 5666 OW SOL 659 30.730 61.750 50.780 1.00 0.00 O

HETATM 5667 HW1 SOL 659 30.860 61.800 51.780 1.00 0.00 H

HETATM 5668 HW2 SOL 659 31.380 61.040 50.490 1.00 0.00 H

HETATM 5669 OW SOL 660 58.410 53.900 75.600 1.00 0.00 O

HETATM 5670 HW1 SOL 660 58.570 54.270 74.670 1.00 0.00 H

HETATM 5671 HW2 SOL 660 58.410 52.880 75.550 1.00 0.00 H

HETATM 5672 OW SOL 661 41.500 28.600 54.890 1.00 0.00 O

HETATM 5673 HW1 SOL 661 42.480 28.320 54.930 1.00 0.00 H

HETATM 5674 HW2 SOL 661 41.380 29.240 55.670 1.00 0.00 H

HETATM 5675 OW SOL 662 67.960 49.450 29.050 1.00 0.00 O

HETATM 5676 HW1 SOL 662 68.280 50.280 29.540 1.00 0.00 H

HETATM 5677 HW2 SOL 662 67.130 49.740 28.550 1.00 0.00 H

HETATM 5678 OW SOL 663 26.920 58.810 48.350 1.00 0.00 O

HETATM 5679 HW1 SOL 663 27.380 59.100 49.200 1.00 0.00 H

HETATM 5680 HW2 SOL 663 27.610 58.320 47.810 1.00 0.00 H

TER 5681 SOL 663

HETATM 5682 OW SOL 665 43.350 51.000 72.590 1.00 0.00 O

HETATM 5683 HW1 SOL 665 42.410 50.910 73.000 1.00 0.00 H

HETATM 5684 HW2 SOL 665 43.230 51.170 71.600 1.00 0.00 H

HETATM 5685 OW SOL 666 30.640 60.030 61.290 1.00 0.00 O

HETATM 5686 HW1 SOL 666 30.700 60.850 61.880 1.00 0.00 H

HETATM 5687 HW2 SOL 666 31.530 59.530 61.430 1.00 0.00 H

HETATM 5688 OW SOL 667 49.180 52.050 32.640 1.00 0.00 O

HETATM 5689 HW1 SOL 667 50.000 52.610 32.620 1.00 0.00 H

HETATM 5690 HW2 SOL 667 48.810 52.070 33.580 1.00 0.00 H

HETATM 5691 OW SOL 668 29.600 58.550 55.940 1.00 0.00 O

HETATM 5692 HW1 SOL 668 30.160 58.680 56.750 1.00 0.00 H

HETATM 5693 HW2 SOL 668 29.310 59.480 55.630 1.00 0.00 H

HETATM 5694 OW SOL 669 29.410 54.120 36.330 1.00 0.00 O

HETATM 5695 HW1 SOL 669 30.180 53.640 36.760 1.00 0.00 H

HETATM 5696 HW2 SOL 669 29.050 53.430 35.680 1.00 0.00 H

HETATM 5697 OW SOL 670 63.510 35.020 44.180 1.00 0.00 O

HETATM 5698 HW1 SOL 670 62.960 34.980 45.010 1.00 0.00 H

HETATM 5699 HW2 SOL 670 63.770 35.980 44.030 1.00 0.00 H

HETATM 5700 OW SOL 671 55.980 49.040 28.190 1.00 0.00 O

HETATM 5701 HW1 SOL 671 56.030 49.330 29.190 1.00 0.00 H

HETATM 5702 HW2 SOL 671 54.990 48.990 28.010 1.00 0.00 H

HETATM 5703 OW SOL 672 50.780 63.950 30.310 1.00 0.00 O

HETATM 5704 HW1 SOL 672 51.530 64.200 29.670 1.00 0.00 H

HETATM 5705 HW2 SOL 672 50.640 62.950 30.280 1.00 0.00 H

HETATM 5706 OW SOL 673 52.280 65.700 28.610 1.00 0.00 O

HETATM 5707 HW1 SOL 673 53.130 66.220 28.740 1.00 0.00 H

HETATM 5708 OW SOL 674 51.160 38.490 23.880 1.00 0.00 O

HETATM 5709 HW1 SOL 674 51.530 37.730 24.430 1.00 0.00 H

HETATM 5710 HW2 SOL 674 50.280 38.770 24.270 1.00 0.00 H

HETATM 5711 OW SOL 675 39.330 53.780 24.220 1.00 0.00 O

HETATM 5712 HW1 SOL 675 39.590 54.150 25.130 1.00 0.00 H

HETATM 5713 HW2 SOL 675 40.190 53.470 23.810 1.00 0.00 H

HETATM 5714 OW SOL 676 49.220 22.940 51.320 1.00 0.00 O

HETATM 5715 HW2 SOL 676 48.600 22.790 50.520 1.00 0.00 H

HETATM 5716 OW SOL 677 61.330 57.890 23.630 1.00 0.00 O

HETATM 5717 HW1 SOL 677 60.420 57.910 24.060 1.00 0.00 H

HETATM 5718 HW2 SOL 677 61.200 57.600 22.680 1.00 0.00 H

HETATM 5719 OW SOL 678 44.070 28.440 56.560 1.00 0.00 O

HETATM 5720 HW1 SOL 678 44.400 29.380 56.650 1.00 0.00 H

HETATM 5721 HW2 SOL 678 44.750 27.960 55.980 1.00 0.00 H

HETATM 5722 OW SOL 679 19.080 42.760 59.500 1.00 0.00 O

HETATM 5723 HW1 SOL 679 19.660 41.950 59.670 1.00 0.00 H

HETATM 5724 HW2 SOL 679 18.150 42.400 59.350 1.00 0.00 H

HETATM 5725 OW SOL 680 51.970 52.090 48.030 1.00 0.00 O

HETATM 5726 HW1 SOL 680 52.480 51.350 47.540 1.00 0.00 H

HETATM 5727 HW2 SOL 680 52.080 52.920 47.430 1.00 0.00 H

TER 5728 SOL 680

HETATM 5729 HW1 SOL 1209 50.420 18.590 42.700 1.00 0.00 H

HETATM 5730 HW2 SOL 1209 49.220 18.420 43.790 1.00 0.00 H

TER 5731 SOL 1209

HETATM 5732 OW SOL 1778 75.170 34.310 32.090 1.00 0.00 O

HETATM 5733 HW1 SOL 1778 74.960 35.230 31.700 1.00 0.00 H

TER 5734 SOL 1778

HETATM 5735 OW SOL 1906 76.750 35.610 42.010 1.00 0.00 O

HETATM 5736 HW1 SOL 1906 77.050 36.210 42.770 1.00 0.00 H

HETATM 5737 HW2 SOL 1906 77.500 35.660 41.310 1.00 0.00 H

TER 5738 SOL 1906

HETATM 5739 OW SOL 1910 80.640 36.430 51.600 1.00 0.00 O

HETATM 5740 HW1 SOL 1910 79.740 36.260 52.060 1.00 0.00 H

HETATM 5741 HW2 SOL 1910 80.790 37.440 51.700 1.00 0.00 H

TER 5742 SOL 1910

HETATM 5743 OW SOL 1915 74.780 34.580 38.560 1.00 0.00 O

HETATM 5744 HW1 SOL 1915 74.620 33.590 38.760 1.00 0.00 H

HETATM 5745 HW2 SOL 1915 74.420 35.090 39.380 1.00 0.00 H

TER 5746 SOL 1915

HETATM 5747 OW SOL 1937 75.740 36.710 52.950 1.00 0.00 O

HETATM 5748 HW1 SOL 1937 75.750 36.700 53.980 1.00 0.00 H

HETATM 5749 HW2 SOL 1937 75.110 37.450 52.680 1.00 0.00 H

TER 5750 SOL 1937

HETATM 5751 OW SOL 1943 77.940 36.710 45.590 1.00 0.00 O

HETATM 5752 HW2 SOL 1943 78.590 37.440 45.870 1.00 0.00 H

TER 5753 SOL 1943

HETATM 5754 OW SOL 1954 77.030 36.470 49.470 1.00 0.00 O

HETATM 5755 HW1 SOL 1954 77.550 35.800 50.010 1.00 0.00 H

HETATM 5756 HW2 SOL 1954 76.830 36.080 48.570 1.00 0.00 H

TER 5757 SOL 1954

HETATM 5758 OW SOL 1973 15.610 33.760 44.100 1.00 0.00 O

HETATM 5759 HW1 SOL 1973 15.120 33.400 44.930 1.00 0.00 H

HETATM 5760 HW2 SOL 1973 14.880 33.860 43.370 1.00 0.00 H

TER 5761 SOL 1973

HETATM 5762 OW SOL 1977 7.750 30.110 47.770 1.00 0.00 O

HETATM 5763 HW2 SOL 1977 7.990 30.950 47.240 1.00 0.00 H

TER 5764 SOL 1977

HETATM 5765 OW SOL 1984 11.330 32.030 54.070 1.00 0.00 O

HETATM 5766 HW2 SOL 1984 10.740 31.180 54.120 1.00 0.00 H

TER 5767 SOL 1984

HETATM 5768 OW SOL 1989 18.460 36.180 40.420 1.00 0.00 O

HETATM 5769 HW1 SOL 1989 18.250 36.000 41.400 1.00 0.00 H

HETATM 5770 HW2 SOL 1989 18.800 37.150 40.360 1.00 0.00 H

TER 5771 SOL 1989

HETATM 5772 OW SOL 1998 14.350 32.690 46.210 1.00 0.00 O

HETATM 5773 HW1 SOL 1998 14.360 32.800 47.200 1.00 0.00 H

HETATM 5774 HW2 SOL 1998 14.320 31.670 46.030 1.00 0.00 H

HETATM 5775 OW SOL 1999 16.880 35.740 42.620 1.00 0.00 O

HETATM 5776 HW1 SOL 1999 16.330 35.520 41.770 1.00 0.00 H

HETATM 5777 HW2 SOL 1999 16.480 35.110 43.330 1.00 0.00 H

TER 5778 SOL 1999

HETATM 5779 OW SOL 2015 12.110 34.120 52.340 1.00 0.00 O

HETATM 5780 HW1 SOL 2015 12.070 33.370 53.040 1.00 0.00 H

HETATM 5781 HW2 SOL 2015 11.150 34.370 52.110 1.00 0.00 H

TER 5782 SOL 2015

HETATM 5783 OW SOL 2026 74.400 31.830 39.210 1.00 0.00 O

HETATM 5784 HW1 SOL 2026 73.460 31.900 38.840 1.00 0.00 H

HETATM 5785 OW SOL 2027 8.720 33.770 49.130 1.00 0.00 O

HETATM 5786 OW SOL 2027 75.420 36.510 46.760 1.00 0.00 O

HETATM 5787 HW1 SOL 2027 8.520 33.500 48.150 1.00 0.00 H

HETATM 5788 HW1 SOL 2027 75.130 35.640 46.290 1.00 0.00 H

HETATM 5789 HW2 SOL 2027 8.540 32.950 49.690 1.00 0.00 H

HETATM 5790 HW2 SOL 2027 76.300 36.760 46.310 1.00 0.00 H

TER 5791 SOL 2027

HETATM 5792 OW SOL 2036 12.130 34.480 45.270 1.00 0.00 O

HETATM 5793 HW2 SOL 2036 13.040 34.150 45.570 1.00 0.00 H

HETATM 5794 OW SOL 2037 48.520 25.600 25.500 1.00 0.00 O

HETATM 5795 HW1 SOL 2037 49.080 26.450 25.400 1.00 0.00 H

HETATM 5796 HW2 SOL 2037 48.180 25.640 26.470 1.00 0.00 H

HETATM 5797 OW SOL 2038 12.650 36.510 43.300 1.00 0.00 O

HETATM 5798 HW1 SOL 2038 13.160 37.180 42.700 1.00 0.00 H

HETATM 5799 OW SOL 2039 44.250 28.810 25.150 1.00 0.00 O

HETATM 5800 HW1 SOL 2039 44.520 28.400 26.020 1.00 0.00 H

HETATM 5801 HW2 SOL 2039 43.360 29.300 25.330 1.00 0.00 H

HETATM 5802 OW SOL 2040 40.230 33.310 29.340 1.00 0.00 O

HETATM 5803 HW1 SOL 2040 39.570 32.690 28.890 1.00 0.00 H

HETATM 5804 HW2 SOL 2040 40.900 33.640 28.650 1.00 0.00 H

TER 5805 SOL 2040

HETATM 5806 OW SOL 2043 39.460 35.820 23.440 1.00 0.00 O

HETATM 5807 HW1 SOL 2043 39.650 36.790 23.720 1.00 0.00 H

HETATM 5808 HW2 SOL 2043 40.200 35.620 22.760 1.00 0.00 H

HETATM 5809 HW1 SOL 2044 39.350 34.040 23.940 1.00 0.00 H

TER 5810 SOL 2044

HETATM 5811 OW SOL 2048 55.570 36.190 21.960 1.00 0.00 O

HETATM 5812 HW1 SOL 2048 56.110 35.840 22.750 1.00 0.00 H

TER 5813 SOL 2048

HETATM 5814 OW SOL 2050 17.500 30.760 52.990 1.00 0.00 O

HETATM 5815 HW1 SOL 2050 18.440 30.550 52.610 1.00 0.00 H

HETATM 5816 HW2 SOL 2050 17.670 31.460 53.690 1.00 0.00 H

HETATM 5817 OW SOL 2051 15.740 35.210 53.830 1.00 0.00 O

HETATM 5818 HW1 SOL 2051 15.820 35.290 52.820 1.00 0.00 H

HETATM 5819 HW2 SOL 2051 16.610 34.810 54.140 1.00 0.00 H

HETATM 5820 OW SOL 2052 42.990 32.470 26.020 1.00 0.00 O

HETATM 5821 HW1 SOL 2052 42.630 31.530 25.850 1.00 0.00 H

HETATM 5822 HW2 SOL 2052 43.140 32.850 25.070 1.00 0.00 H

TER 5823 SOL 2052

HETATM 5824 OW SOL 2058 52.700 35.480 21.580 1.00 0.00 O

HETATM 5825 HW1 SOL 2058 53.660 35.640 21.870 1.00 0.00 H

HETATM 5826 OW SOL 2059 50.990 23.170 35.420 1.00 0.00 O

HETATM 5827 HW1 SOL 2059 51.570 23.840 35.900 1.00 0.00 H

HETATM 5828 HW2 SOL 2059 51.660 22.590 34.940 1.00 0.00 H

TER 5829 SOL 2059

HETATM 5830 OW SOL 2061 38.000 26.860 26.350 1.00 0.00 O

HETATM 5831 HW1 SOL 2061 38.850 26.630 26.910 1.00 0.00 H

HETATM 5832 HW2 SOL 2061 37.640 27.730 26.710 1.00 0.00 H

TER 5833 SOL 2061

HETATM 5834 HW1 SOL 2067 38.360 21.860 33.520 1.00 0.00 H

TER 5835 SOL 2067

HETATM 5836 OW SOL 2069 41.860 35.890 21.850 1.00 0.00 O

HETATM 5837 HW1 SOL 2069 42.320 36.520 22.520 1.00 0.00 H

HETATM 5838 HW2 SOL 2069 42.590 35.620 21.170 1.00 0.00 H

TER 5839 SOL 2069

HETATM 5840 OW SOL 2072 52.620 20.810 35.900 1.00 0.00 O

HETATM 5841 HW1 SOL 2072 52.420 21.370 36.720 1.00 0.00 H

TER 5842 SOL 2072

HETATM 5843 OW SOL 2075 45.740 33.420 30.760 1.00 0.00 O

HETATM 5844 HW1 SOL 2075 46.350 33.610 29.980 1.00 0.00 H

HETATM 5845 HW2 SOL 2075 46.280 32.920 31.470 1.00 0.00 H

TER 5846 SOL 2075

HETATM 5847 OW SOL 2077 78.350 35.810 52.950 1.00 0.00 O

HETATM 5848 HW1 SOL 2077 77.510 36.340 52.690 1.00 0.00 H

HETATM 5849 HW2 SOL 2077 77.960 35.010 53.450 1.00 0.00 H

HETATM 5850 OW SOL 2078 10.690 32.060 45.840 1.00 0.00 O

HETATM 5851 HW2 SOL 2078 11.280 32.760 45.390 1.00 0.00 H

TER 5852 SOL 2078

HETATM 5853 OW SOL 2081 41.880 29.980 25.720 1.00 0.00 O

HETATM 5854 HW1 SOL 2081 41.130 29.750 26.360 1.00 0.00 H

HETATM 5855 HW2 SOL 2081 41.440 29.890 24.780 1.00 0.00 H

TER 5856 SOL 2081

HETATM 5857 HW1 SOL 2084 80.870 37.490 46.440 1.00 0.00 H

TER 5858 SOL 2084

HETATM 5859 HW1 SOL 2088 9.020 35.010 50.980 1.00 0.00 H

HETATM 5860 OW SOL 2089 53.970 31.570 24.940 1.00 0.00 O

HETATM 5861 HW1 SOL 2089 54.960 31.430 25.180 1.00 0.00 H

HETATM 5862 HW2 SOL 2089 53.830 32.580 24.960 1.00 0.00 H

TER 5863 SOL 2089

HETATM 5864 OW SOL 2091 13.040 29.030 49.370 1.00 0.00 O

HETATM 5865 HW1 SOL 2091 13.570 28.560 48.620 1.00 0.00 H

HETATM 5866 HW2 SOL 2091 12.270 28.390 49.490 1.00 0.00 H

TER 5867 SOL 2091

HETATM 5868 OW SOL 2094 41.270 26.780 31.690 1.00 0.00 O

HETATM 5869 HW1 SOL 2094 42.220 26.600 31.430 1.00 0.00 H

HETATM 5870 HW2 SOL 2094 40.820 25.850 31.590 1.00 0.00 H

TER 5871 SOL 2094

HETATM 5872 OW SOL 2096 38.030 34.310 31.920 1.00 0.00 O

HETATM 5873 HW1 SOL 2096 37.420 35.110 31.870 1.00 0.00 H

HETATM 5874 HW2 SOL 2096 38.980 34.610 31.970 1.00 0.00 H

TER 5875 SOL 2096

HETATM 5876 OW SOL 2098 8.250 31.560 51.170 1.00 0.00 O

HETATM 5877 HW1 SOL 2098 8.030 31.900 52.120 1.00 0.00 H

HETATM 5878 HW2 SOL 2098 7.330 31.550 50.730 1.00 0.00 H

HETATM 5879 OW SOL 2099 49.940 32.070 22.340 1.00 0.00 O

HETATM 5880 HW1 SOL 2099 50.570 32.840 22.570 1.00 0.00 H

HETATM 5881 HW2 SOL 2099 49.080 32.210 22.840 1.00 0.00 H

TER 5882 SOL 2099

HETATM 5883 OW SOL 2101 41.610 26.100 34.720 1.00 0.00 O

HETATM 5884 HW1 SOL 2101 41.480 26.490 33.800 1.00 0.00 H

HETATM 5885 HW2 SOL 2101 41.840 25.120 34.600 1.00 0.00 H

TER 5886 SOL 2101

HETATM 5887 OW SOL 2103 8.260 28.750 51.020 1.00 0.00 O

HETATM 5888 HW1 SOL 2103 8.370 28.580 50.030 1.00 0.00 H

HETATM 5889 HW2 SOL 2103 8.230 29.760 51.150 1.00 0.00 H

TER 5890 SOL 2103

HETATM 5891 OW SOL 2106 37.410 31.750 31.410 1.00 0.00 O

HETATM 5892 HW1 SOL 2106 38.150 31.110 31.710 1.00 0.00 H

HETATM 5893 HW2 SOL 2106 37.720 32.710 31.670 1.00 0.00 H

HETATM 5894 OW SOL 2107 44.690 25.580 28.980 1.00 0.00 O

HETATM 5895 HW1 SOL 2107 45.270 24.780 28.690 1.00 0.00 H

HETATM 5896 HW2 SOL 2107 44.030 25.750 28.240 1.00 0.00 H

TER 5897 SOL 2107

HETATM 5898 OW SOL 2110 47.590 25.920 28.090 1.00 0.00 O

HETATM 5899 HW1 SOL 2110 47.660 25.890 29.120 1.00 0.00 H

HETATM 5900 HW2 SOL 2110 47.120 25.020 27.870 1.00 0.00 H

HETATM 5901 HW1 SOL 2111 13.890 27.890 50.630 1.00 0.00 H

TER 5902 SOL 2111

HETATM 5903 OW SOL 2113 40.070 26.340 27.910 1.00 0.00 O

HETATM 5904 HW1 SOL 2113 40.950 26.780 28.190 1.00 0.00 H

HETATM 5905 HW2 SOL 2113 40.160 25.360 28.220 1.00 0.00 H

TER 5906 SOL 2113

HETATM 5907 OW SOL 2115 14.670 30.550 53.530 1.00 0.00 O

HETATM 5908 OW SOL 2115 42.200 22.950 36.920 1.00 0.00 O

HETATM 5909 HW1 SOL 2115 41.920 23.650 37.600 1.00 0.00 H

HETATM 5910 HW1 SOL 2116 48.030 22.890 36.100 1.00 0.00 H

TER 5911 SOL 2116

HETATM 5912 OW SOL 2119 41.930 34.460 27.510 1.00 0.00 O

HETATM 5913 HW1 SOL 2119 42.730 34.900 27.930 1.00 0.00 H

HETATM 5914 HW2 SOL 2119 42.330 33.720 26.900 1.00 0.00 H

HETATM 5915 OW SOL 2120 47.500 26.040 30.750 1.00 0.00 O

HETATM 5916 HW1 SOL 2120 47.920 26.310 31.630 1.00 0.00 H

HETATM 5917 HW2 SOL 2120 46.650 25.530 30.970 1.00 0.00 H

HETATM 5918 OW SOL 2121 37.730 23.990 29.560 1.00 0.00 O

HETATM 5919 HW1 SOL 2121 38.730 23.920 29.410 1.00 0.00 H

HETATM 5920 HW2 SOL 2121 37.590 24.570 30.380 1.00 0.00 H

HETATM 5921 OW SOL 2122 39.120 23.950 35.790 1.00 0.00 O

HETATM 5922 HW1 SOL 2122 39.760 23.440 35.200 1.00 0.00 H

HETATM 5923 HW2 SOL 2122 38.740 24.680 35.190 1.00 0.00 H

TER 5924 SOL 2122

HETATM 5925 OW SOL 2124 53.590 24.600 30.440 1.00 0.00 O

HETATM 5926 HW1 SOL 2124 53.510 24.030 31.280 1.00 0.00 H

HETATM 5927 HW2 SOL 2124 54.540 24.410 30.100 1.00 0.00 H

TER 5928 SOL 2124

HETATM 5929 OW SOL 2127 37.810 31.790 28.620 1.00 0.00 O

HETATM 5930 HW1 SOL 2127 37.670 31.700 29.630 1.00 0.00 H

HETATM 5931 HW2 SOL 2127 37.360 30.990 28.190 1.00 0.00 H

TER 5932 SOL 2127

HETATM 5933 OW SOL 2130 49.930 27.910 25.380 1.00 0.00 O

HETATM 5934 HW1 SOL 2130 50.730 27.270 25.580 1.00 0.00 H

HETATM 5935 OW SOL 2131 45.840 31.780 32.880 1.00 0.00 O

HETATM 5936 HW1 SOL 2131 45.640 32.090 33.810 1.00 0.00 H

HETATM 5937 HW2 SOL 2131 44.990 31.720 32.340 1.00 0.00 H

HETATM 5938 OW SOL 2132 43.680 34.930 20.070 1.00 0.00 O

HETATM 5939 HW1 SOL 2132 43.940 35.010 19.090 1.00 0.00 H

HETATM 5940 HW2 SOL 2132 43.200 34.010 20.150 1.00 0.00 H

TER 5941 SOL 2132

HETATM 5942 HW1 SOL 2135 49.290 33.740 19.240 1.00 0.00 H

TER 5943 SOL 2135

HETATM 5944 OW SOL 2138 47.480 29.370 25.660 1.00 0.00 O

HETATM 5945 HW1 SOL 2138 46.810 29.240 24.920 1.00 0.00 H

HETATM 5946 HW2 SOL 2138 48.370 28.980 25.330 1.00 0.00 H

TER 5947 SOL 2138

HETATM 5948 OW SOL 2140 43.220 33.430 23.490 1.00 0.00 O

HETATM 5949 HW1 SOL 2140 43.750 33.000 22.740 1.00 0.00 H

HETATM 5950 HW2 SOL 2140 42.690 34.170 23.050 1.00 0.00 H

HETATM 5951 OW SOL 2141 54.160 22.520 32.280 1.00 0.00 O

HETATM 5952 HW1 SOL 2141 54.780 23.170 32.750 1.00 0.00 H

TER 5953 SOL 2141

HETATM 5954 OW SOL 2146 49.050 32.010 19.730 1.00 0.00 O

HETATM 5955 HW1 SOL 2146 48.080 31.730 19.810 1.00 0.00 H

HETATM 5956 HW2 SOL 2146 49.420 31.990 20.690 1.00 0.00 H

HETATM 5957 OW SOL 2147 44.350 24.230 35.740 1.00 0.00 O

HETATM 5958 HW1 SOL 2147 44.680 24.830 36.470 1.00 0.00 H

HETATM 5959 OW SOL 2148 54.320 27.400 30.940 1.00 0.00 O

HETATM 5960 HW1 SOL 2148 53.950 26.470 31.110 1.00 0.00 H

HETATM 5961 HW2 SOL 2148 53.830 27.780 30.170 1.00 0.00 H

TER 5962 SOL 2148

HETATM 5963 OW SOL 2150 43.140 26.100 25.400 1.00 0.00 O

HETATM 5964 OW SOL 2151 55.010 21.640 35.050 1.00 0.00 O

HETATM 5965 HW1 SOL 2151 54.820 22.580 34.700 1.00 0.00 H

HETATM 5966 HW2 SOL 2151 54.110 21.300 35.400 1.00 0.00 H

HETATM 5967 OW SOL 2152 45.800 32.320 18.930 1.00 0.00 O

HETATM 5968 HW1 SOL 2152 45.620 32.660 19.870 1.00 0.00 H

HETATM 5969 HW2 SOL 2152 46.060 33.120 18.360 1.00 0.00 H

TER 5970 SOL 2152

HETATM 5971 HW1 SOL 2154 37.750 30.420 26.050 1.00 0.00 H

TER 5972 SOL 2154

HETATM 5973 OW SOL 2157 40.850 24.160 31.430 1.00 0.00 O

HETATM 5974 HW1 SOL 2157 41.180 23.460 32.100 1.00 0.00 H

HETATM 5975 HW2 SOL 2157 40.800 23.740 30.510 1.00 0.00 H

TER 5976 SOL 2157

HETATM 5977 OW SOL 2160 43.470 34.710 31.360 1.00 0.00 O

HETATM 5978 HW1 SOL 2160 43.880 35.390 31.970 1.00 0.00 H

HETATM 5979 HW2 SOL 2160 44.260 34.130 31.040 1.00 0.00 H

TER 5980 SOL 2160

HETATM 5981 OW SOL 2165 39.610 24.660 38.370 1.00 0.00 O

HETATM 5982 HW1 SOL 2165 38.740 25.040 38.700 1.00 0.00 H

HETATM 5983 HW2 SOL 2165 39.540 24.540 37.360 1.00 0.00 H

TER 5984 SOL 2165

HETATM 5985 OW SOL 2167 37.260 22.280 43.650 1.00 0.00 O

HETATM 5986 OW SOL 2168 54.870 24.710 45.700 1.00 0.00 O

HETATM 5987 HW1 SOL 2168 55.030 25.690 45.840 1.00 0.00 H

HETATM 5988 HW2 SOL 2168 55.290 24.260 46.500 1.00 0.00 H

TER 5989 SOL 2168

HETATM 5990 OW SOL 2170 53.200 23.090 44.500 1.00 0.00 O

HETATM 5991 HW1 SOL 2170 52.430 23.670 44.230 1.00 0.00 H

HETATM 5992 HW2 SOL 2170 53.840 23.730 45.000 1.00 0.00 H

HETATM 5993 OW SOL 2171 43.310 28.290 38.290 1.00 0.00 O

HETATM 5994 HW1 SOL 2171 43.880 28.840 37.680 1.00 0.00 H

HETATM 5995 HW2 SOL 2171 43.930 27.850 38.960 1.00 0.00 H

HETATM 5996 HW1 SOL 2172 52.860 20.230 51.130 1.00 0.00 H

HETATM 5997 HW2 SOL 2172 52.980 20.270 52.770 1.00 0.00 H

HETATM 5998 OW SOL 2173 38.520 25.970 53.010 1.00 0.00 O

HETATM 5999 HW1 SOL 2173 39.490 26.150 53.190 1.00 0.00 H

HETATM 6000 HW2 SOL 2173 38.070 26.850 53.300 1.00 0.00 H

HETATM 6001 HW1 SOL 2174 45.660 19.490 42.220 1.00 0.00 H

TER 6002 SOL 2174

HETATM 6003 OW SOL 2176 50.180 21.600 46.430 1.00 0.00 O

HETATM 6004 HW1 SOL 2176 49.200 21.360 46.630 1.00 0.00 H

HETATM 6005 HW2 SOL 2176 50.440 22.240 47.190 1.00 0.00 H

HETATM 6006 OW SOL 2177 45.540 24.480 51.940 1.00 0.00 O

HETATM 6007 HW2 SOL 2177 44.990 24.080 51.170 1.00 0.00 H

HETATM 6008 OW SOL 2178 50.900 23.220 54.320 1.00 0.00 O

HETATM 6009 HW1 SOL 2178 51.270 23.470 55.220 1.00 0.00 H

HETATM 6010 HW2 SOL 2178 51.620 22.660 53.870 1.00 0.00 H

HETATM 6011 HW1 SOL 2179 46.190 22.940 49.880 1.00 0.00 H

HETATM 6012 OW SOL 2180 46.230 22.110 39.310 1.00 0.00 O

HETATM 6013 HW1 SOL 2180 46.530 22.660 38.500 1.00 0.00 H

HETATM 6014 HW2 SOL 2180 46.150 21.140 38.960 1.00 0.00 H

HETATM 6015 OW SOL 2181 54.600 24.280 55.470 1.00 0.00 O

HETATM 6016 HW1 SOL 2181 54.440 24.990 54.780 1.00 0.00 H

HETATM 6017 HW2 SOL 2181 54.760 23.420 54.930 1.00 0.00 H

TER 6018 SOL 2181

HETATM 6019 OW SOL 2183 40.360 36.810 45.490 1.00 0.00 O

HETATM 6020 HW1 SOL 2183 41.230 36.330 45.360 1.00 0.00 H

HETATM 6021 HW2 SOL 2183 40.470 37.750 45.130 1.00 0.00 H

TER 6022 SOL 2183

HETATM 6023 OW SOL 2185 51.210 19.780 41.590 1.00 0.00 O

HETATM 6024 HW1 SOL 2185 51.110 19.460 40.610 1.00 0.00 H

HETATM 6025 HW2 SOL 2185 52.200 19.700 41.810 1.00 0.00 H

HETATM 6026 OW SOL 2186 52.650 20.880 54.480 1.00 0.00 O

TER 6027 SOL 2186

HETATM 6028 OW SOL 2191 46.650 20.800 41.660 1.00 0.00 O

HETATM 6029 HW1 SOL 2191 46.170 21.370 40.950 1.00 0.00 H

HETATM 6030 HW2 SOL 2191 47.110 20.070 41.120 1.00 0.00 H

HETATM 6031 OW SOL 2192 48.040 25.450 51.850 1.00 0.00 O

HETATM 6032 HW1 SOL 2192 47.090 25.060 51.940 1.00 0.00 H

HETATM 6033 HW2 SOL 2192 48.660 24.650 51.720 1.00 0.00 H

HETATM 6034 OW SOL 2193 47.530 23.990 37.400 1.00 0.00 O

HETATM 6035 HW1 SOL 2193 47.030 24.870 37.380 1.00 0.00 H

HETATM 6036 HW2 SOL 2193 48.100 23.990 38.230 1.00 0.00 H

HETATM 6037 HW1 SOL 2194 38.950 24.090 50.760 1.00 0.00 H

HETATM 6038 HW2 SOL 2194 38.020 24.940 51.770 1.00 0.00 H

TER 6039 SOL 2194

HETATM 6040 OW SOL 2196 47.380 19.140 44.630 1.00 0.00 O

HETATM 6041 HW1 SOL 2196 46.670 18.980 43.920 1.00 0.00 H

HETATM 6042 OW SOL 2197 42.660 21.220 40.550 1.00 0.00 O

HETATM 6043 HW1 SOL 2197 42.940 20.870 39.630 1.00 0.00 H

HETATM 6044 HW2 SOL 2197 42.360 22.190 40.430 1.00 0.00 H

TER 6045 SOL 2197

HETATM 6046 OW SOL 2199 41.210 26.600 50.140 1.00 0.00 O

HETATM 6047 HW1 SOL 2199 42.180 26.740 49.840 1.00 0.00 H

HETATM 6048 HW2 SOL 2199 41.060 25.590 50.230 1.00 0.00 H

HETATM 6049 OW SOL 2200 42.910 23.950 46.120 1.00 0.00 O

HETATM 6050 HW2 SOL 2200 43.550 24.330 46.810 1.00 0.00 H

TER 6051 SOL 2200

HETATM 6052 OW SOL 2205 40.520 23.790 47.470 1.00 0.00 O

TER 6053 SOL 2205

HETATM 6054 HW1 SOL 2207 47.990 22.770 54.770 1.00 0.00 H

HETATM 6055 OW SOL 2208 47.140 26.370 49.350 1.00 0.00 O

HETATM 6056 HW1 SOL 2208 47.460 26.110 50.290 1.00 0.00 H

HETATM 6057 HW2 SOL 2208 46.130 26.280 49.340 1.00 0.00 H

HETATM 6058 OW SOL 2209 37.640 24.240 48.270 1.00 0.00 O

HETATM 6059 HW1 SOL 2209 38.550 24.030 47.860 1.00 0.00 H

HETATM 6060 HW2 SOL 2209 37.800 24.160 49.280 1.00 0.00 H

TER 6061 SOL 2209

HETATM 6062 OW SOL 2211 44.360 23.290 49.760 1.00 0.00 O

HETATM 6063 HW1 SOL 2211 43.720 22.570 49.440 1.00 0.00 H

HETATM 6064 HW2 SOL 2211 44.080 24.180 49.340 1.00 0.00 H

TER 6065 SOL 2211

HETATM 6066 OW SOL 2213 54.480 20.020 42.380 1.00 0.00 O

HETATM 6067 HW1 SOL 2213 55.240 19.920 41.710 1.00 0.00 H

TER 6068 SOL 2213

HETATM 6069 OW SOL 2215 39.840 21.710 44.430 1.00 0.00 O

HETATM 6070 HW1 SOL 2215 38.930 22.070 44.110 1.00 0.00 H

HETATM 6071 OW SOL 2216 47.550 20.900 46.890 1.00 0.00 O

HETATM 6072 HW1 SOL 2216 47.380 20.360 46.040 1.00 0.00 H

HETATM 6073 OW SOL 2217 38.040 28.240 54.590 1.00 0.00 O

HETATM 6074 HW1 SOL 2217 37.410 28.640 55.260 1.00 0.00 H

HETATM 6075 HW2 SOL 2217 38.980 28.500 54.880 1.00 0.00 H

TER 6076 SOL 2217

HETATM 6077 OW SOL 2220 51.920 27.540 51.280 1.00 0.00 O

HETATM 6078 HW1 SOL 2220 51.070 27.780 50.810 1.00 0.00 H

HETATM 6079 HW2 SOL 2220 52.690 28.020 50.840 1.00 0.00 H

HETATM 6080 OW SOL 2221 37.870 20.150 40.300 1.00 0.00 O

HETATM 6081 HW1 SOL 2221 37.720 20.940 39.690 1.00 0.00 H

HETATM 6082 HW2 SOL 2221 38.790 19.790 40.000 1.00 0.00 H

HETATM 6083 HW1 SOL 2222 44.920 24.760 55.070 1.00 0.00 H

HETATM 6084 OW SOL 2223 40.790 36.570 53.020 1.00 0.00 O

HETATM 6085 HW1 SOL 2223 39.900 36.860 52.660 1.00 0.00 H

HETATM 6086 HW2 SOL 2223 40.750 35.570 53.040 1.00 0.00 H

HETATM 6087 OW SOL 2224 55.040 21.940 53.950 1.00 0.00 O

HETATM 6088 HW1 SOL 2224 54.910 22.240 52.990 1.00 0.00 H

HETATM 6089 HW2 SOL 2224 54.170 21.420 54.160 1.00 0.00 H

HETATM 6090 OW SOL 2225 55.520 20.570 49.710 1.00 0.00 O

HETATM 6091 HW2 SOL 2225 55.530 20.660 48.700 1.00 0.00 H

HETATM 6092 OW SOL 2226 40.450 24.010 50.190 1.00 0.00 O

HETATM 6093 HW2 SOL 2226 40.650 23.820 49.190 1.00 0.00 H

TER 6094 SOL 2226

HETATM 6095 OW SOL 2228 50.710 19.110 38.960 1.00 0.00 O

HETATM 6096 HW1 SOL 2228 51.020 18.820 38.030 1.00 0.00 H

HETATM 6097 HW2 SOL 2228 49.820 18.640 39.110 1.00 0.00 H

HETATM 6098 OW SOL 2229 52.950 20.790 46.020 1.00 0.00 O

HETATM 6099 HW1 SOL 2229 51.950 20.900 46.200 1.00 0.00 H

HETATM 6100 HW2 SOL 2229 53.160 21.560 45.380 1.00 0.00 H

HETATM 6101 OW SOL 2230 37.330 28.690 40.050 1.00 0.00 O

HETATM 6102 HW1 SOL 2230 38.110 29.320 39.870 1.00 0.00 H

HETATM 6103 HW2 SOL 2230 37.420 27.960 39.360 1.00 0.00 H

TER 6104 SOL 2230

HETATM 6105 OW SOL 2237 54.690 24.610 58.180 1.00 0.00 O

HETATM 6106 HW1 SOL 2237 54.760 24.420 57.170 1.00 0.00 H

HETATM 6107 HW2 SOL 2237 54.460 23.710 58.610 1.00 0.00 H

TER 6108 SOL 2237

HETATM 6109 HW2 SOL 2241 41.030 33.420 65.960 1.00 0.00 H

TER 6110 SOL 2241

HETATM 6111 OW SOL 2250 54.250 27.800 62.050 1.00 0.00 O

HETATM 6112 HW1 SOL 2250 55.120 27.340 62.300 1.00 0.00 H

HETATM 6113 HW2 SOL 2250 54.480 28.550 61.430 1.00 0.00 H

TER 6114 SOL 2250

HETATM 6115 HW1 SOL 2257 43.290 26.800 58.080 1.00 0.00 H

HETATM 6116 HW2 SOL 2257 42.450 26.050 59.300 1.00 0.00 H

TER 6117 SOL 2257

HETATM 6118 OW SOL 2268 51.060 29.940 61.250 1.00 0.00 O

HETATM 6119 HW1 SOL 2268 52.000 30.250 61.080 1.00 0.00 H

HETATM 6120 HW2 SOL 2268 50.430 30.700 61.030 1.00 0.00 H

TER 6121 SOL 2268

HETATM 6122 OW SOL 2270 55.190 29.010 64.680 1.00 0.00 O

HETATM 6123 HW1 SOL 2270 54.610 29.200 63.870 1.00 0.00 H

HETATM 6124 HW2 SOL 2270 54.550 29.130 65.490 1.00 0.00 H

TER 6125 SOL 2270

HETATM 6126 OW SOL 2277 39.790 36.510 68.090 1.00 0.00 O

HETATM 6127 HW2 SOL 2277 39.660 35.980 67.250 1.00 0.00 H

TER 6128 SOL 2277

HETATM 6129 OW SOL 2282 45.990 33.810 67.820 1.00 0.00 O

HETATM 6130 HW1 SOL 2282 45.790 33.390 66.900 1.00 0.00 H

TER 6131 SOL 2282

HETATM 6132 OW SOL 2284 45.390 32.520 65.310 1.00 0.00 O

HETATM 6133 HW1 SOL 2284 46.310 32.370 64.880 1.00 0.00 H

HETATM 6134 HW2 SOL 2284 44.790 32.840 64.550 1.00 0.00 H

TER 6135 SOL 2284

HETATM 6136 OW SOL 2290 41.930 30.140 63.190 1.00 0.00 O

HETATM 6137 HW1 SOL 2290 41.180 29.930 63.850 1.00 0.00 H

HETATM 6138 HW2 SOL 2290 41.570 29.870 62.270 1.00 0.00 H

HETATM 6139 OW SOL 2291 47.480 23.820 56.240 1.00 0.00 O

HETATM 6140 HW1 SOL 2291 47.450 24.720 55.790 1.00 0.00 H

HETATM 6141 HW2 SOL 2291 48.160 23.910 57.010 1.00 0.00 H

TER 6142 SOL 2291

HETATM 6143 OW SOL 2296 51.740 30.150 64.610 1.00 0.00 O

HETATM 6144 HW2 SOL 2296 51.830 30.460 63.660 1.00 0.00 H

TER 6145 SOL 2296

HETATM 6146 OW SOL 2303 53.520 29.580 66.690 1.00 0.00 O

HETATM 6147 HW2 SOL 2303 52.740 29.830 66.060 1.00 0.00 H

TER 6148 SOL 2303

HETATM 6149 OW SOL 2309 40.970 29.580 60.690 1.00 0.00 O

HETATM 6150 HW1 SOL 2309 40.800 30.330 60.050 1.00 0.00 H

HETATM 6151 HW2 SOL 2309 40.720 28.740 60.160 1.00 0.00 H

TER 6152 SOL 2309

HETATM 6153 OW SOL 2311 52.850 25.370 60.450 1.00 0.00 O

HETATM 6154 HW1 SOL 2311 52.620 25.540 61.430 1.00 0.00 H

HETATM 6155 HW2 SOL 2311 53.840 25.150 60.390 1.00 0.00 H

TER 6156 SOL 2311

HETATM 6157 OW SOL 2319 49.140 24.040 58.490 1.00 0.00 O

HETATM 6158 HW1 SOL 2319 49.340 23.190 59.030 1.00 0.00 H

HETATM 6159 HW2 SOL 2319 48.680 24.670 59.150 1.00 0.00 H

TER 6160 SOL 2319

HETATM 6161 HW2 SOL 2325 52.920 22.990 60.130 1.00 0.00 H

TER 6162 SOL 2325

HETATM 6163 OW SOL 2331 47.820 31.810 64.370 1.00 0.00 O

HETATM 6164 HW1 SOL 2331 47.800 30.960 63.770 1.00 0.00 H

HETATM 6165 HW2 SOL 2331 48.630 32.320 64.080 1.00 0.00 H

TER 6166 SOL 2331

HETATM 6167 OW SOL 2337 38.090 36.560 65.220 1.00 0.00 O

HETATM 6168 HW1 SOL 2337 37.730 35.650 64.860 1.00 0.00 H

HETATM 6169 HW2 SOL 2337 39.020 36.630 64.820 1.00 0.00 H

HETATM 6170 OW SOL 2338 42.080 34.430 65.100 1.00 0.00 O

HETATM 6171 HW1 SOL 2338 42.740 35.170 65.280 1.00 0.00 H

HETATM 6172 HW2 SOL 2338 42.530 33.860 64.390 1.00 0.00 H

TER 6173 SOL 2338

HETATM 6174 OW SOL 2358 53.380 35.830 69.820 1.00 0.00 O

HETATM 6175 HW1 SOL 2358 54.110 35.830 69.120 1.00 0.00 H

HETATM 6176 HW2 SOL 2358 53.230 36.820 70.080 1.00 0.00 H

TER 6177 SOL 2358

HETATM 6178 OW SOL 2364 47.870 29.580 62.890 1.00 0.00 O

HETATM 6179 HW1 SOL 2364 47.090 29.100 63.360 1.00 0.00 H

TER 6180 SOL 2364

HETATM 6181 OW SOL 2378 43.150 25.840 62.580 1.00 0.00 O

HETATM 6182 HW1 SOL 2378 43.490 26.780 62.680 1.00 0.00 H

HETATM 6183 HW2 SOL 2378 42.420 25.850 61.860 1.00 0.00 H

TER 6184 SOL 2378

HETATM 6185 OW SOL 2382 46.020 28.320 64.350 1.00 0.00 O

HETATM 6186 HW2 SOL 2382 45.490 28.820 65.070 1.00 0.00 H

HETATM 6187 OW SOL 2383 55.460 34.480 64.710 1.00 0.00 O

HETATM 6188 HW1 SOL 2383 55.200 33.970 63.880 1.00 0.00 H

HETATM 6189 HW2 SOL 2383 55.050 33.970 65.520 1.00 0.00 H

TER 6190 SOL 2383

HETATM 6191 OW SOL 2385 37.950 30.960 62.560 1.00 0.00 O

HETATM 6192 HW2 SOL 2385 38.800 30.700 62.080 1.00 0.00 H

TER 6193 SOL 2385

HETATM 6194 OW SOL 2389 54.300 33.250 66.750 1.00 0.00 O

HETATM 6195 HW1 SOL 2389 53.310 33.250 66.960 1.00 0.00 H

HETATM 6196 HW2 SOL 2389 54.720 32.700 67.490 1.00 0.00 H

TER 6197 SOL 2389

HETATM 6198 OW SOL 2627 75.730 55.710 15.850 1.00 0.00 O

HETATM 6199 HW1 SOL 2627 75.560 55.260 16.740 1.00 0.00 H

TER 6200 SOL 2627

HETATM 6201 OW SOL 2665 45.740 45.090 18.110 1.00 0.00 O

HETATM 6202 HW1 SOL 2665 45.920 45.880 18.720 1.00 0.00 H

HETATM 6203 HW2 SOL 2665 46.070 45.440 17.190 1.00 0.00 H

TER 6204 SOL 2665

HETATM 6205 OW SOL 2699 46.760 46.110 15.830 1.00 0.00 O

TER 6206 SOL 2699

HETATM 6207 HW1 SOL 2723 52.650 39.810 18.280 1.00 0.00 H

HETATM 6208 OW SOL 2724 77.300 52.940 17.490 1.00 0.00 O

HETATM 6209 HW1 SOL 2724 76.560 53.120 18.170 1.00 0.00 H

HETATM 6210 HW2 SOL 2724 77.130 51.970 17.270 1.00 0.00 H

HETATM 6211 OW SOL 2725 44.980 40.090 17.570 1.00 0.00 O

HETATM 6212 HW2 SOL 2725 44.290 39.810 18.270 1.00 0.00 H

TER 6213 SOL 2725

HETATM 6214 OW SOL 2751 17.080 42.960 36.920 1.00 0.00 O

HETATM 6215 HW1 SOL 2751 16.700 43.780 36.420 1.00 0.00 H

HETATM 6216 HW2 SOL 2751 17.340 42.300 36.180 1.00 0.00 H

TER 6217 SOL 2751

HETATM 6218 HW1 SOL 2755 74.820 47.250 17.630 1.00 0.00 H

TER 6219 SOL 2755

HETATM 6220 OW SOL 2777 76.770 50.100 18.560 1.00 0.00 O

HETATM 6221 HW1 SOL 2777 76.210 49.370 18.130 1.00 0.00 H

HETATM 6222 HW2 SOL 2777 76.080 50.830 18.800 1.00 0.00 H

TER 6223 SOL 2777

HETATM 6224 OW SOL 2782 40.760 46.360 18.150 1.00 0.00 O

HETATM 6225 HW1 SOL 2782 41.610 46.680 18.620 1.00 0.00 H

HETATM 6226 HW2 SOL 2782 41.060 45.760 17.370 1.00 0.00 H

TER 6227 SOL 2782

HETATM 6228 OW SOL 2788 42.120 41.500 17.800 1.00 0.00 O

HETATM 6229 HW1 SOL 2788 42.120 41.710 18.810 1.00 0.00 H

HETATM 6230 HW2 SOL 2788 42.950 41.960 17.430 1.00 0.00 H

HETATM 6231 HW1 SOL 2789 48.110 41.550 17.390 1.00 0.00 H

TER 6232 SOL 2789

HETATM 6233 OW SOL 2792 17.570 49.590 34.260 1.00 0.00 O

HETATM 6234 HW1 SOL 2792 18.080 49.100 34.990 1.00 0.00 H

TER 6235 SOL 2792

HETATM 6236 HW1 SOL 2794 75.950 43.610 20.240 1.00 0.00 H

TER 6237 SOL 2794

HETATM 6238 HW1 SOL 2796 73.690 41.350 25.550 1.00 0.00 H

HETATM 6239 OW SOL 2797 80.200 50.190 30.500 1.00 0.00 O

HETATM 6240 HW1 SOL 2797 79.280 50.170 30.070 1.00 0.00 H

HETATM 6241 HW2 SOL 2797 80.320 51.170 30.780 1.00 0.00 H

TER 6242 SOL 2797

HETATM 6243 OW SOL 2803 83.190 44.930 36.840 1.00 0.00 O

HETATM 6244 HW1 SOL 2803 82.730 45.590 37.440 1.00 0.00 H

HETATM 6245 HW2 SOL 2803 83.420 45.430 35.970 1.00 0.00 H

HETATM 6246 OW SOL 2804 81.400 47.400 25.070 1.00 0.00 O

HETATM 6247 HW1 SOL 2804 82.080 47.150 25.800 1.00 0.00 H

HETATM 6248 HW2 SOL 2804 80.590 47.780 25.570 1.00 0.00 H

HETATM 6249 OW SOL 2805 81.750 48.990 36.930 1.00 0.00 O

HETATM 6250 HW1 SOL 2805 82.200 49.860 37.240 1.00 0.00 H

HETATM 6251 HW2 SOL 2805 80.810 49.300 36.660 1.00 0.00 H

HETATM 6252 OW SOL 2806 77.860 51.460 29.440 1.00 0.00 O

HETATM 6253 HW1 SOL 2806 77.330 50.850 28.840 1.00 0.00 H

HETATM 6254 HW2 SOL 2806 78.340 52.140 28.860 1.00 0.00 H

TER 6255 SOL 2806

HETATM 6256 OW SOL 2808 78.970 41.050 33.120 1.00 0.00 O

HETATM 6257 HW1 SOL 2808 79.750 40.470 33.410 1.00 0.00 H

HETATM 6258 HW2 SOL 2808 78.120 40.650 33.520 1.00 0.00 H

HETATM 6259 OW SOL 2809 16.070 45.010 35.460 1.00 0.00 O

HETATM 6260 HW2 SOL 2809 16.600 45.540 34.740 1.00 0.00 H

HETATM 6261 OW SOL 2810 77.070 54.260 23.560 1.00 0.00 O

HETATM 6262 HW1 SOL 2810 77.130 55.210 23.940 1.00 0.00 H

HETATM 6263 HW2 SOL 2810 77.480 54.300 22.640 1.00 0.00 H

TER 6264 SOL 2810

HETATM 6265 OW SOL 2812 77.330 48.120 27.870 1.00 0.00 O

HETATM 6266 HW1 SOL 2812 77.450 47.100 27.970 1.00 0.00 H

HETATM 6267 HW2 SOL 2812 76.540 48.330 28.480 1.00 0.00 H

HETATM 6268 OW SOL 2813 80.220 55.120 33.060 1.00 0.00 O

HETATM 6269 HW1 SOL 2813 80.590 54.620 33.880 1.00 0.00 H

HETATM 6270 HW2 SOL 2813 80.610 56.070 33.170 1.00 0.00 H

HETATM 6271 HW2 SOL 2814 80.790 47.340 20.810 1.00 0.00 H

TER 6272 SOL 2814

HETATM 6273 OW SOL 2816 17.230 46.970 37.070 1.00 0.00 O

HETATM 6274 OW SOL 2816 75.950 43.450 24.270 1.00 0.00 O

HETATM 6275 HW1 SOL 2816 16.870 46.190 36.530 1.00 0.00 H

HETATM 6276 HW1 SOL 2816 75.410 42.620 24.490 1.00 0.00 H

HETATM 6277 HW2 SOL 2816 75.490 44.260 24.670 1.00 0.00 H

TER 6278 SOL 2816

HETATM 6279 HW2 SOL 2820 19.360 52.440 35.080 1.00 0.00 H

TER 6280 SOL 2820

HETATM 6281 OW SOL 2824 77.490 39.730 36.590 1.00 0.00 O

HETATM 6282 HW1 SOL 2824 78.300 40.370 36.570 1.00 0.00 H

HETATM 6283 HW2 SOL 2824 76.730 40.310 36.940 1.00 0.00 H

TER 6284 SOL 2824

HETATM 6285 OW SOL 2830 82.840 42.970 33.190 1.00 0.00 O

HETATM 6286 HW1 SOL 2830 82.320 43.000 34.080 1.00 0.00 H

HETATM 6287 HW2 SOL 2830 82.190 42.510 32.520 1.00 0.00 H

TER 6288 SOL 2830

HETATM 6289 OW SOL 2832 84.070 46.170 34.450 1.00 0.00 O

HETATM 6290 HW1 SOL 2832 84.640 45.390 34.090 1.00 0.00 H

HETATM 6291 HW2 SOL 2832 39.350 47.120 17.660 1.00 0.00 H

HETATM 6292 HW2 SOL 2832 83.650 46.550 33.580 1.00 0.00 H

TER 6293 SOL 2832

HETATM 6294 OW SOL 2839 84.530 51.630 23.270 1.00 0.00 O

HETATM 6295 HW1 SOL 2839 84.080 52.440 22.810 1.00 0.00 H

HETATM 6296 HW2 SOL 2839 84.500 51.830 24.260 1.00 0.00 H

HETATM 6297 OW SOL 2840 44.430 42.830 17.090 1.00 0.00 O

HETATM 6298 OW SOL 2840 77.980 55.350 27.260 1.00 0.00 O

HETATM 6299 HW1 SOL 2840 44.890 43.620 17.570 1.00 0.00 H

HETATM 6300 HW1 SOL 2840 78.490 54.460 27.280 1.00 0.00 H

HETATM 6301 HW2 SOL 2840 44.890 41.960 17.360 1.00 0.00 H

TER 6302 SOL 2840

HETATM 6303 OW SOL 2843 77.900 46.840 21.200 1.00 0.00 O

HETATM 6304 HW2 SOL 2843 77.300 46.140 20.800 1.00 0.00 H

TER 6305 SOL 2843

HETATM 6306 OW SOL 2845 79.250 50.140 36.250 1.00 0.00 O

HETATM 6307 HW1 SOL 2845 78.780 50.180 35.330 1.00 0.00 H

HETATM 6308 HW2 SOL 2845 78.570 49.770 36.900 1.00 0.00 H

TER 6309 SOL 2845

HETATM 6310 HW1 SOL 2847 75.840 40.850 33.420 1.00 0.00 H

HETATM 6311 HW2 SOL 2847 76.350 39.800 34.550 1.00 0.00 H

TER 6312 SOL 2847

HETATM 6313 OW SOL 2849 79.220 54.620 21.680 1.00 0.00 O

HETATM 6314 HW2 SOL 2849 80.020 54.370 21.090 1.00 0.00 H

TER 6315 SOL 2849

HETATM 6316 HW2 SOL 2852 77.520 44.170 23.580 1.00 0.00 H

TER 6317 SOL 2852

HETATM 6318 HW2 SOL 2854 38.940 50.250 19.500 1.00 0.00 H

TER 6319 SOL 2854

HETATM 6320 HW2 SOL 2858 82.220 51.250 27.100 1.00 0.00 H

TER 6321 SOL 2858

HETATM 6322 HW1 SOL 2864 84.450 43.510 33.240 1.00 0.00 H

TER 6323 SOL 2864

HETATM 6324 OW SOL 2866 79.050 48.540 25.860 1.00 0.00 O

HETATM 6325 HW1 SOL 2866 78.470 48.480 26.720 1.00 0.00 H

HETATM 6326 HW2 SOL 2866 78.550 47.920 25.230 1.00 0.00 H

TER 6327 SOL 2866

HETATM 6328 OW SOL 2869 75.290 42.580 32.750 1.00 0.00 O

HETATM 6329 HW1 SOL 2869 76.140 42.740 32.200 1.00 0.00 H

HETATM 6330 HW2 SOL 2869 75.110 43.450 33.230 1.00 0.00 H

TER 6331 SOL 2869

HETATM 6332 OW SOL 2871 17.230 46.660 33.680 1.00 0.00 O

HETATM 6333 HW1 SOL 2871 17.240 47.670 33.800 1.00 0.00 H

HETATM 6334 HW2 SOL 2871 17.150 46.480 32.670 1.00 0.00 H

HETATM 6335 OW SOL 2872 53.500 41.760 25.800 1.00 0.00 O

HETATM 6336 HW1 SOL 2872 52.760 41.870 25.100 1.00 0.00 H

HETATM 6337 HW2 SOL 2872 53.030 42.170 26.610 1.00 0.00 H

HETATM 6338 OW SOL 2873 43.520 54.960 32.870 1.00 0.00 O

HETATM 6339 HW1 SOL 2873 43.160 54.110 33.350 1.00 0.00 H

HETATM 6340 HW2 SOL 2873 43.060 55.690 33.400 1.00 0.00 H

HETATM 6341 OW SOL 2874 43.130 47.070 19.840 1.00 0.00 O

HETATM 6342 HW1 SOL 2874 43.530 47.720 19.140 1.00 0.00 H

HETATM 6343 HW2 SOL 2874 43.880 46.970 20.500 1.00 0.00 H

HETATM 6344 OW SOL 2875 55.760 54.930 21.810 1.00 0.00 O

HETATM 6345 HW1 SOL 2875 56.350 54.570 22.560 1.00 0.00 H

HETATM 6346 HW2 SOL 2875 55.920 55.940 21.820 1.00 0.00 H

HETATM 6347 OW SOL 2876 79.450 51.250 20.130 1.00 0.00 O

HETATM 6348 HW1 SOL 2876 78.480 51.060 20.320 1.00 0.00 H

HETATM 6349 HW2 SOL 2876 79.530 51.030 19.140 1.00 0.00 H

HETATM 6350 OW SOL 2877 54.540 46.450 24.800 1.00 0.00 O

HETATM 6351 HW1 SOL 2877 55.020 46.080 23.980 1.00 0.00 H

HETATM 6352 HW2 SOL 2877 53.960 47.190 24.410 1.00 0.00 H

TER 6353 SOL 2877

HETATM 6354 OW SOL 2880 53.300 53.530 21.400 1.00 0.00 O

HETATM 6355 HW1 SOL 2880 54.010 54.230 21.600 1.00 0.00 H

HETATM 6356 HW2 SOL 2880 52.920 53.680 20.470 1.00 0.00 H

HETATM 6357 OW SOL 2881 38.070 55.260 34.230 1.00 0.00 O

HETATM 6358 HW1 SOL 2881 38.200 55.210 35.250 1.00 0.00 H

HETATM 6359 HW2 SOL 2881 37.700 56.170 34.060 1.00 0.00 H

TER 6360 SOL 2881

HETATM 6361 OW SOL 2883 42.550 39.200 27.360 1.00 0.00 O

HETATM 6362 HW1 SOL 2883 42.630 39.080 28.380 1.00 0.00 H

HETATM 6363 HW2 SOL 2883 43.410 39.660 27.070 1.00 0.00 H

HETATM 6364 HW2 SOL 2884 47.220 51.940 24.300 1.00 0.00 H

HETATM 6365 OW SOL 2885 40.580 55.400 26.740 1.00 0.00 O

HETATM 6366 HW1 SOL 2885 40.830 54.410 26.790 1.00 0.00 H

HETATM 6367 HW2 SOL 2885 41.300 55.880 27.260 1.00 0.00 H

HETATM 6368 OW SOL 2886 52.510 44.550 26.190 1.00 0.00 O

HETATM 6369 OW SOL 2886 84.280 48.430 35.860 1.00 0.00 O

HETATM 6370 HW1 SOL 2886 51.740 44.080 26.660 1.00 0.00 H

HETATM 6371 HW1 SOL 2886 83.310 48.550 36.160 1.00 0.00 H

HETATM 6372 HW2 SOL 2886 52.200 45.500 26.050 1.00 0.00 H

HETATM 6373 HW2 SOL 2886 84.270 47.580 35.270 1.00 0.00 H

HETATM 6374 OW SOL 2887 81.610 47.900 22.380 1.00 0.00 O

HETATM 6375 HW1 SOL 2887 81.520 47.610 23.360 1.00 0.00 H

HETATM 6376 HW2 SOL 2887 82.400 47.300 22.050 1.00 0.00 H

HETATM 6377 OW SOL 2888 78.870 45.390 31.100 1.00 0.00 O

HETATM 6378 HW1 SOL 2888 79.180 45.530 32.050 1.00 0.00 H

HETATM 6379 HW2 SOL 2888 78.300 44.540 31.150 1.00 0.00 H

HETATM 6380 HW1 SOL 2889 42.220 55.200 22.450 1.00 0.00 H

HETATM 6381 OW SOL 2890 43.500 38.640 19.620 1.00 0.00 O

HETATM 6382 HW1 SOL 2890 44.460 38.280 19.790 1.00 0.00 H

HETATM 6383 OW SOL 2891 42.610 37.710 23.690 1.00 0.00 O

HETATM 6384 HW1 SOL 2891 41.970 38.390 23.270 1.00 0.00 H

HETATM 6385 HW2 SOL 2891 43.510 38.130 23.450 1.00 0.00 H

TER 6386 SOL 2891

HETATM 6387 OW SOL 2893 15.290 52.680 44.400 1.00 0.00 O

HETATM 6388 HW1 SOL 2893 15.210 51.780 44.850 1.00 0.00 H

HETATM 6389 OW SOL 2894 11.320 44.230 44.210 1.00 0.00 O

HETATM 6390 OW SOL 2894 42.830 38.930 30.140 1.00 0.00 O

HETATM 6391 HW1 SOL 2894 11.860 45.090 44.020 1.00 0.00 H

HETATM 6392 HW1 SOL 2894 43.780 38.660 30.340 1.00 0.00 H

HETATM 6393 HW2 SOL 2894 42.540 39.630 30.810 1.00 0.00 H

HETATM 6394 OW SOL 2895 17.700 43.110 39.610 1.00 0.00 O

HETATM 6395 OW SOL 2895 51.920 48.150 26.680 1.00 0.00 O

HETATM 6396 HW1 SOL 2895 17.520 43.170 38.600 1.00 0.00 H

HETATM 6397 HW1 SOL 2895 51.300 48.470 27.410 1.00 0.00 H

HETATM 6398 HW2 SOL 2895 17.190 42.310 39.960 1.00 0.00 H

HETATM 6399 HW2 SOL 2895 51.530 48.500 25.800 1.00 0.00 H

TER 6400 SOL 2895

HETATM 6401 OW SOL 2897 42.300 51.300 20.180 1.00 0.00 O

HETATM 6402 HW1 SOL 2897 41.500 51.280 20.800 1.00 0.00 H

HETATM 6403 HW2 SOL 2897 79.770 45.760 29.770 1.00 0.00 H

HETATM 6404 OW SOL 2898 54.000 50.170 24.660 1.00 0.00 O

HETATM 6405 HW1 SOL 2898 54.980 50.110 24.970 1.00 0.00 H

HETATM 6406 HW2 SOL 2898 53.720 51.150 24.770 1.00 0.00 H

HETATM 6407 OW SOL 2899 51.200 42.970 19.300 1.00 0.00 O

HETATM 6408 HW1 SOL 2899 50.710 42.810 20.170 1.00 0.00 H

HETATM 6409 HW2 SOL 2899 52.160 42.680 19.450 1.00 0.00 H

HETATM 6410 OW SOL 2900 42.120 39.700 21.650 1.00 0.00 O

HETATM 6411 HW1 SOL 2900 42.740 39.230 20.980 1.00 0.00 H

HETATM 6412 HW2 SOL 2900 42.120 40.660 21.330 1.00 0.00 H

HETATM 6413 OW SOL 2901 81.700 43.530 29.150 1.00 0.00 O

HETATM 6414 HW1 SOL 2901 50.350 51.510 22.750 1.00 0.00 H

HETATM 6415 HW2 SOL 2901 81.350 44.480 28.940 1.00 0.00 H

HETATM 6416 HW1 SOL 2902 52.720 43.850 24.200 1.00 0.00 H

HETATM 6417 OW SOL 2903 40.170 38.250 24.630 1.00 0.00 O

HETATM 6418 HW1 SOL 2903 40.170 39.280 24.640 1.00 0.00 H

HETATM 6419 HW2 SOL 2903 41.160 38.030 24.760 1.00 0.00 H

HETATM 6420 OW SOL 2904 53.440 40.980 22.290 1.00 0.00 O

HETATM 6421 HW1 SOL 2904 53.490 40.280 23.010 1.00 0.00 H

HETATM 6422 HW2 SOL 2904 53.190 41.870 22.760 1.00 0.00 H

HETATM 6423 OW SOL 2905 18.390 54.850 40.430 1.00 0.00 O

HETATM 6424 OW SOL 2905 52.540 40.060 19.880 1.00 0.00 O

HETATM 6425 HW1 SOL 2905 19.080 54.400 41.040 1.00 0.00 H

HETATM 6426 HW1 SOL 2905 51.920 39.260 20.070 1.00 0.00 H

HETATM 6427 HW2 SOL 2905 18.710 55.820 40.380 1.00 0.00 H

HETATM 6428 HW2 SOL 2905 52.910 40.360 20.790 1.00 0.00 H

HETATM 6429 OW SOL 2906 50.100 45.530 18.900 1.00 0.00 O

HETATM 6430 HW1 SOL 2906 50.530 44.600 18.920 1.00 0.00 H

HETATM 6431 OW SOL 2907 16.400 46.710 43.350 1.00 0.00 O

HETATM 6432 OW SOL 2907 42.000 42.200 20.370 1.00 0.00 O

HETATM 6433 HW1 SOL 2907 17.160 46.110 43.580 1.00 0.00 H

HETATM 6434 HW1 SOL 2907 42.490 43.060 20.530 1.00 0.00 H

HETATM 6435 HW2 SOL 2907 16.840 47.640 43.280 1.00 0.00 H

HETATM 6436 HW2 SOL 2907 41.040 42.400 20.670 1.00 0.00 H

HETATM 6437 OW SOL 2908 50.520 45.700 21.570 1.00 0.00 O

HETATM 6438 HW1 SOL 2908 49.690 45.850 22.120 1.00 0.00 H

HETATM 6439 HW2 SOL 2908 50.210 45.650 20.580 1.00 0.00 H

HETATM 6440 OW SOL 2909 46.090 37.980 19.710 1.00 0.00 O

HETATM 6441 HW1 SOL 2909 46.710 38.030 18.890 1.00 0.00 H

HETATM 6442 HW2 SOL 2909 46.580 37.480 20.430 1.00 0.00 H

HETATM 6443 OW SOL 2910 54.880 38.630 24.140 1.00 0.00 O

HETATM 6444 HW1 SOL 2910 55.170 38.450 23.180 1.00 0.00 H

HETATM 6445 HW2 SOL 2910 15.930 47.380 39.970 1.00 0.00 H

HETATM 6446 HW2 SOL 2910 54.170 37.920 24.370 1.00 0.00 H

HETATM 6447 OW SOL 2911 38.150 55.310 36.920 1.00 0.00 O

HETATM 6448 OW SOL 2911 77.680 45.460 28.030 1.00 0.00 O

HETATM 6449 HW1 SOL 2911 38.040 54.480 37.470 1.00 0.00 H

HETATM 6450 HW1 SOL 2911 78.700 45.490 28.120 1.00 0.00 H

HETATM 6451 HW2 SOL 2911 38.870 55.860 37.370 1.00 0.00 H

HETATM 6452 HW2 SOL 2911 77.440 44.520 28.320 1.00 0.00 H

TER 6453 SOL 2911

HETATM 6454 OW SOL 2913 79.670 41.260 36.690 1.00 0.00 O

HETATM 6455 HW1 SOL 2913 79.440 41.670 37.600 1.00 0.00 H

HETATM 6456 HW2 SOL 2913 80.170 42.000 36.170 1.00 0.00 H

TER 6457 SOL 2913

HETATM 6458 OW SOL 2915 12.860 40.580 46.540 1.00 0.00 O

HETATM 6459 OW SOL 2915 40.980 50.210 32.880 1.00 0.00 O

HETATM 6460 HW1 SOL 2915 11.880 40.340 46.690 1.00 0.00 H

HETATM 6461 HW1 SOL 2915 41.510 51.040 33.170 1.00 0.00 H

HETATM 6462 HW2 SOL 2915 13.360 40.140 47.290 1.00 0.00 H

HETATM 6463 HW2 SOL 2915 40.080 50.320 33.310 1.00 0.00 H

HETATM 6464 OW SOL 2916 75.330 55.190 27.910 1.00 0.00 O

HETATM 6465 HW1 SOL 2916 74.920 54.470 27.340 1.00 0.00 H

HETATM 6466 HW2 SOL 2916 38.420 41.400 18.540 1.00 0.00 H

HETATM 6467 HW2 SOL 2916 76.330 55.230 27.670 1.00 0.00 H

HETATM 6468 OW SOL 2917 79.550 52.950 27.710 1.00 0.00 O

HETATM 6469 HW2 SOL 2917 80.150 52.170 27.870 1.00 0.00 H

HETATM 6470 OW SOL 2918 13.860 41.950 53.980 1.00 0.00 O

HETATM 6471 OW SOL 2918 84.400 45.070 30.780 1.00 0.00 O

HETATM 6472 HW1 SOL 2918 84.940 44.850 31.620 1.00 0.00 H

HETATM 6473 HW2 SOL 2918 14.480 41.150 54.100 1.00 0.00 H

HETATM 6474 HW2 SOL 2918 83.460 44.790 31.040 1.00 0.00 H

HETATM 6475 OW SOL 2919 9.570 46.100 53.130 1.00 0.00 O

HETATM 6476 HW1 SOL 2919 10.170 45.330 52.810 1.00 0.00 H

HETATM 6477 HW2 SOL 2919 9.170 46.510 52.270 1.00 0.00 H

HETATM 6478 OW SOL 2920 47.780 46.950 19.430 1.00 0.00 O

HETATM 6479 HW1 SOL 2920 47.690 47.630 18.680 1.00 0.00 H

HETATM 6480 HW2 SOL 2920 48.640 46.430 19.240 1.00 0.00 H

TER 6481 SOL 2920

HETATM 6482 OW SOL 2922 50.770 37.990 19.980 1.00 0.00 O

HETATM 6483 OW SOL 2922 81.530 41.770 31.190 1.00 0.00 O

HETATM 6484 HW1 SOL 2922 51.010 37.450 19.140 1.00 0.00 H

HETATM 6485 HW2 SOL 2922 50.560 37.310 20.690 1.00 0.00 H

HETATM 6486 HW2 SOL 2922 81.470 42.450 30.420 1.00 0.00 H

HETATM 6487 OW SOL 2923 16.730 42.970 55.520 1.00 0.00 O

HETATM 6488 OW SOL 2923 50.730 42.250 25.160 1.00 0.00 O

HETATM 6489 HW1 SOL 2923 17.090 43.890 55.330 1.00 0.00 H

HETATM 6490 HW1 SOL 2923 49.730 42.290 25.040 1.00 0.00 H

HETATM 6491 HW2 SOL 2923 17.180 42.320 54.870 1.00 0.00 H

HETATM 6492 HW2 SOL 2923 50.880 42.000 26.150 1.00 0.00 H

HETATM 6493 OW SOL 2924 53.530 39.690 27.720 1.00 0.00 O

HETATM 6494 HW1 SOL 2924 14.560 48.700 42.340 1.00 0.00 H

HETATM 6495 HW1 SOL 2924 52.700 39.930 28.260 1.00 0.00 H

HETATM 6496 HW2 SOL 2924 53.390 40.160 26.830 1.00 0.00 H

TER 6497 SOL 2924

HETATM 6498 OW SOL 2926 52.580 41.710 44.540 1.00 0.00 O

HETATM 6499 HW1 SOL 2926 52.450 41.830 43.550 1.00 0.00 H

HETATM 6500 HW2 SOL 2926 53.560 41.780 44.750 1.00 0.00 H

HETATM 6501 OW SOL 2927 38.150 43.730 42.780 1.00 0.00 O

HETATM 6502 HW1 SOL 2927 37.610 42.920 43.020 1.00 0.00 H

HETATM 6503 HW2 SOL 2927 38.380 44.230 43.620 1.00 0.00 H

HETATM 6504 OW SOL 2928 12.060 52.840 52.310 1.00 0.00 O

HETATM 6505 OW SOL 2928 46.840 54.060 46.070 1.00 0.00 O

HETATM 6506 HW1 SOL 2928 12.940 52.290 52.380 1.00 0.00 H

HETATM 6507 HW1 SOL 2928 47.840 53.800 46.070 1.00 0.00 H

HETATM 6508 HW2 SOL 2928 11.480 52.240 51.710 1.00 0.00 H

HETATM 6509 HW2 SOL 2928 46.550 53.780 45.150 1.00 0.00 H

HETATM 6510 OW SOL 2929 46.640 39.350 41.950 1.00 0.00 O

HETATM 6511 OW SOL 2929 81.250 54.030 35.310 1.00 0.00 O

HETATM 6512 HW1 SOL 2929 46.140 39.890 41.270 1.00 0.00 H

HETATM 6513 HW2 SOL 2929 47.470 39.860 42.180 1.00 0.00 H

HETATM 6514 OW SOL 2930 49.450 53.390 45.550 1.00 0.00 O

HETATM 6515 HW1 SOL 2930 49.670 53.960 44.730 1.00 0.00 H

HETATM 6516 HW2 SOL 2930 49.730 52.440 45.290 1.00 0.00 H

HETATM 6517 OW SOL 2931 49.950 55.280 43.550 1.00 0.00 O

HETATM 6518 HW1 SOL 2931 50.540 55.670 42.800 1.00 0.00 H

HETATM 6519 HW2 SOL 2931 49.120 55.840 43.550 1.00 0.00 H

HETATM 6520 OW SOL 2932 39.850 40.150 44.860 1.00 0.00 O

HETATM 6521 HW1 SOL 2932 39.510 40.710 44.090 1.00 0.00 H

HETATM 6522 HW2 SOL 2932 39.930 40.770 45.650 1.00 0.00 H

HETATM 6523 OW SOL 2933 43.250 52.370 42.550 1.00 0.00 O

HETATM 6524 HW1 SOL 2933 43.760 51.750 41.950 1.00 0.00 H

HETATM 6525 HW2 SOL 2933 14.970 38.400 41.710 1.00 0.00 H

HETATM 6526 HW2 SOL 2933 43.170 53.260 42.090 1.00 0.00 H

HETATM 6527 OW SOL 2934 52.480 54.650 46.980 1.00 0.00 O

HETATM 6528 OW SOL 2934 82.620 49.970 32.850 1.00 0.00 O

HETATM 6529 HW1 SOL 2934 52.080 55.030 46.130 1.00 0.00 H

HETATM 6530 HW1 SOL 2934 82.270 50.380 33.730 1.00 0.00 H

HETATM 6531 HW2 SOL 2934 53.060 55.360 47.360 1.00 0.00 H

HETATM 6532 HW2 SOL 2934 81.850 50.100 32.210 1.00 0.00 H

HETATM 6533 OW SOL 2935 44.230 48.700 73.950 1.00 0.00 O

HETATM 6534 OW SOL 2935 81.290 53.330 20.170 1.00 0.00 O

HETATM 6535 HW2 SOL 2935 43.510 49.220 73.460 1.00 0.00 H

HETATM 6536 HW2 SOL 2935 80.610 52.560 20.200 1.00 0.00 H

HETATM 6537 OW SOL 2936 15.460 39.720 54.480 1.00 0.00 O

HETATM 6538 OW SOL 2936 41.780 41.110 70.300 1.00 0.00 O

HETATM 6539 HW1 SOL 2936 15.460 39.890 55.510 1.00 0.00 H

HETATM 6540 HW1 SOL 2936 42.510 40.500 70.670 1.00 0.00 H

HETATM 6541 HW2 SOL 2936 14.990 38.800 54.420 1.00 0.00 H

HETATM 6542 HW2 SOL 2936 40.900 40.800 70.710 1.00 0.00 H

HETATM 6543 OW SOL 2937 53.060 38.370 70.560 1.00 0.00 O

HETATM 6544 HW1 SOL 2937 53.480 39.120 70.010 1.00 0.00 H

HETATM 6545 HW2 SOL 2937 52.890 38.760 71.490 1.00 0.00 H

HETATM 6546 OW SOL 2938 39.480 47.030 68.620 1.00 0.00 O

HETATM 6547 HW1 SOL 2938 40.180 46.290 68.590 1.00 0.00 H

HETATM 6548 HW2 SOL 2938 38.610 46.640 68.350 1.00 0.00 H

TER 6549 SOL 2938

HETATM 6550 OW SOL 2940 13.750 43.100 51.370 1.00 0.00 O

HETATM 6551 OW SOL 2940 45.450 42.960 70.340 1.00 0.00 O

HETATM 6552 OW SOL 2940 83.160 47.360 32.180 1.00 0.00 O

HETATM 6553 HW1 SOL 2940 13.720 42.840 52.360 1.00 0.00 H

HETATM 6554 HW1 SOL 2940 83.390 47.270 31.210 1.00 0.00 H

HETATM 6555 HW2 SOL 2940 13.390 44.050 51.300 1.00 0.00 H

HETATM 6556 HW2 SOL 2940 44.580 42.820 69.850 1.00 0.00 H

HETATM 6557 HW2 SOL 2940 82.960 48.340 32.390 1.00 0.00 H

HETATM 6558 OW SOL 2941 47.130 41.440 68.230 1.00 0.00 O

HETATM 6559 HW1 SOL 2941 46.670 41.900 69.000 1.00 0.00 H

HETATM 6560 HW2 SOL 2941 46.490 40.780 67.840 1.00 0.00 H

HETATM 6561 OW SOL 2942 54.420 43.280 73.990 1.00 0.00 O

HETATM 6562 HW1 SOL 2942 55.260 43.790 73.720 1.00 0.00 H

HETATM 6563 HW2 SOL 2942 54.490 42.390 73.480 1.00 0.00 H

HETATM 6564 OW SOL 2943 49.600 52.280 70.580 1.00 0.00 O

HETATM 6565 OW SOL 2943 78.320 50.300 33.740 1.00 0.00 O

HETATM 6566 HW1 SOL 2943 49.740 52.550 71.560 1.00 0.00 H

HETATM 6567 HW1 SOL 2943 78.910 50.310 32.920 1.00 0.00 H

HETATM 6568 HW2 SOL 2943 49.400 53.160 70.100 1.00 0.00 H

HETATM 6569 HW2 SOL 2943 77.500 49.730 33.540 1.00 0.00 H

TER 6570 SOL 2943

HETATM 6571 OW SOL 2947 10.680 43.670 52.390 1.00 0.00 O

HETATM 6572 OW SOL 2947 46.320 42.200 64.700 1.00 0.00 O

HETATM 6573 HW1 SOL 2947 9.800 43.400 51.940 1.00 0.00 H

HETATM 6574 HW1 SOL 2947 46.530 41.580 63.930 1.00 0.00 H

HETATM 6575 HW2 SOL 2947 11.340 43.900 51.660 1.00 0.00 H

HETATM 6576 HW2 SOL 2947 47.020 42.030 65.410 1.00 0.00 H

HETATM 6577 OW SOL 2948 47.960 44.130 70.700 1.00 0.00 O

HETATM 6578 HW2 SOL 2948 48.600 43.620 70.120 1.00 0.00 H

HETATM 6579 OW SOL 2949 38.030 42.880 69.570 1.00 0.00 O

HETATM 6580 HW1 SOL 2949 38.930 42.440 69.740 1.00 0.00 H

HETATM 6581 HW2 SOL 2949 37.950 43.640 70.230 1.00 0.00 H

HETATM 6582 OW SOL 2950 54.760 49.570 71.830 1.00 0.00 O

HETATM 6583 HW1 SOL 2950 55.160 48.880 71.190 1.00 0.00 H

HETATM 6584 HW2 SOL 2950 54.810 50.460 71.360 1.00 0.00 H

HETATM 6585 HW1 SOL 2951 46.590 37.900 68.910 1.00 0.00 H

HETATM 6586 OW SOL 2952 46.950 47.960 73.000 1.00 0.00 O

HETATM 6587 HW1 SOL 2952 46.120 48.450 73.360 1.00 0.00 H

HETATM 6588 HW2 SOL 2952 46.820 47.920 72.000 1.00 0.00 H

HETATM 6589 OW SOL 2953 41.280 45.020 68.570 1.00 0.00 O

HETATM 6590 HW1 SOL 2953 42.060 45.690 68.730 1.00 0.00 H

HETATM 6591 HW2 SOL 2953 41.160 44.610 69.500 1.00 0.00 H

HETATM 6592 OW SOL 2954 50.290 51.680 67.670 1.00 0.00 O

HETATM 6593 OW SOL 2954 81.740 42.850 35.690 1.00 0.00 O

HETATM 6594 HW1 SOL 2954 49.930 52.030 68.540 1.00 0.00 H

HETATM 6595 HW1 SOL 2954 82.190 43.600 36.230 1.00 0.00 H

HETATM 6596 HW2 SOL 2954 49.690 50.930 67.370 1.00 0.00 H

HETATM 6597 OW SOL 2955 52.870 43.840 60.400 1.00 0.00 O

HETATM 6598 HW1 SOL 2955 52.940 44.330 61.270 1.00 0.00 H

HETATM 6599 HW2 SOL 2955 53.560 44.200 59.770 1.00 0.00 H

TER 6600 SOL 2955

HETATM 6601 OW SOL 2958 10.660 50.800 51.060 1.00 0.00 O

HETATM 6602 HW1 SOL 2958 9.720 50.430 51.240 1.00 0.00 H

HETATM 6603 HW2 SOL 2958 11.230 49.980 50.850 1.00 0.00 H

HETATM 6604 OW SOL 2959 11.220 49.860 48.230 1.00 0.00 O

HETATM 6605 OW SOL 2959 50.280 45.890 66.420 1.00 0.00 O

HETATM 6606 HW1 SOL 2959 10.950 49.010 48.710 1.00 0.00 H

HETATM 6607 HW1 SOL 2959 50.070 46.090 65.460 1.00 0.00 H

HETATM 6608 HW2 SOL 2959 51.270 45.600 66.420 1.00 0.00 H

HETATM 6609 OW SOL 2960 44.450 43.170 66.540 1.00 0.00 O

HETATM 6610 HW1 SOL 2960 45.180 42.990 65.850 1.00 0.00 H

HETATM 6611 HW2 SOL 2960 44.230 44.170 66.500 1.00 0.00 H

HETATM 6612 OW SOL 2961 9.980 48.280 54.640 1.00 0.00 O

HETATM 6613 HW1 SOL 2961 9.010 48.600 54.730 1.00 0.00 H

HETATM 6614 HW1 SOL 2961 41.490 46.660 74.320 1.00 0.00 H

HETATM 6615 HW1 SOL 2961 82.700 51.270 19.810 1.00 0.00 H

HETATM 6616 HW2 SOL 2961 9.940 47.550 53.920 1.00 0.00 H

TER 6617 SOL 2961

HETATM 6618 HW2 SOL 2963 49.610 41.250 72.820 1.00 0.00 H

HETATM 6619 OW SOL 2964 12.600 40.910 50.110 1.00 0.00 O

HETATM 6620 HW1 SOL 2964 13.050 41.760 50.480 1.00 0.00 H

HETATM 6621 HW2 SOL 2964 49.260 39.780 69.800 1.00 0.00 H

TER 6622 SOL 2964

HETATM 6623 OW SOL 2966 38.270 54.780 74.700 1.00 0.00 O

HETATM 6624 HW1 SOL 2966 38.920 54.010 74.820 1.00 0.00 H

HETATM 6625 HW2 SOL 2966 38.700 55.600 75.110 1.00 0.00 H

HETATM 6626 OW SOL 2967 44.010 39.280 70.810 1.00 0.00 O

HETATM 6627 OW SOL 2967 81.230 51.280 34.760 1.00 0.00 O

HETATM 6628 HW1 SOL 2967 44.900 38.930 70.440 1.00 0.00 H

HETATM 6629 HW1 SOL 2967 80.520 50.820 35.330 1.00 0.00 H

HETATM 6630 HW2 SOL 2967 81.190 52.260 35.030 1.00 0.00 H

HETATM 6631 OW SOL 2968 37.820 40.640 56.190 1.00 0.00 O

HETATM 6632 HW1 SOL 2968 13.290 51.650 49.590 1.00 0.00 H

HETATM 6633 HW1 SOL 2968 36.960 40.410 55.710 1.00 0.00 H

HETATM 6634 HW2 SOL 2968 38.260 41.390 55.700 1.00 0.00 H

TER 6635 SOL 2968

HETATM 6636 OW SOL 2970 54.710 40.930 72.560 1.00 0.00 O

HETATM 6637 HW1 SOL 2970 54.930 40.810 71.580 1.00 0.00 H

HETATM 6638 HW2 SOL 2970 53.890 40.320 72.730 1.00 0.00 H

HETATM 6639 OW SOL 2971 77.710 42.910 31.500 1.00 0.00 O

HETATM 6640 HW1 SOL 2971 78.270 42.220 32.040 1.00 0.00 H

HETATM 6641 HW2 SOL 2971 77.810 42.660 30.510 1.00 0.00 H

HETATM 6642 OW SOL 2972 82.530 53.040 25.360 1.00 0.00 O

HETATM 6643 HW1 SOL 2972 82.900 53.640 24.630 1.00 0.00 H

HETATM 6644 HW2 SOL 2972 81.600 53.380 25.530 1.00 0.00 H

TER 6645 SOL 2972

HETATM 6646 OW SOL 2974 13.640 45.820 47.480 1.00 0.00 O

HETATM 6647 HW1 SOL 2974 12.770 46.230 47.190 1.00 0.00 H

HETATM 6648 HW2 SOL 2974 13.420 45.150 48.190 1.00 0.00 H

HETATM 6649 OW SOL 2975 52.470 52.420 81.680 1.00 0.00 O

HETATM 6650 OW SOL 2975 82.380 50.490 21.780 1.00 0.00 O

HETATM 6651 HW1 SOL 2975 52.420 52.130 82.670 1.00 0.00 H

HETATM 6652 HW1 SOL 2975 82.200 49.480 21.780 1.00 0.00 H

HETATM 6653 HW2 SOL 2975 51.480 52.500 81.450 1.00 0.00 H

HETATM 6654 HW2 SOL 2975 83.310 50.610 22.180 1.00 0.00 H

HETATM 6655 OW SOL 2976 80.640 52.750 31.380 1.00 0.00 O

HETATM 6656 HW1 SOL 2976 80.530 53.620 31.890 1.00 0.00 H

HETATM 6657 OW SOL 2977 16.150 40.930 40.830 1.00 0.00 O

HETATM 6658 HW1 SOL 2977 16.450 40.190 41.460 1.00 0.00 H

HETATM 6659 HW2 SOL 2977 15.600 41.580 41.380 1.00 0.00 H

HETATM 6660 OW SOL 2978 54.740 43.410 76.740 1.00 0.00 O

HETATM 6661 HW1 SOL 2978 10.160 44.970 47.800 1.00 0.00 H

HETATM 6662 HW1 SOL 2978 54.600 43.300 75.730 1.00 0.00 H

HETATM 6663 HW2 SOL 2979 10.170 51.980 53.830 1.00 0.00 H

HETATM 6664 OW SOL 2980 83.250 53.740 22.160 1.00 0.00 O

HETATM 6665 HW1 SOL 2980 82.570 53.600 21.400 1.00 0.00 H

HETATM 6666 OW SOL 2981 76.820 39.500 47.410 1.00 0.00 O

HETATM 6667 HW1 SOL 2981 76.660 39.140 48.340 1.00 0.00 H

HETATM 6668 HW2 SOL 2981 75.900 39.550 47.010 1.00 0.00 H

TER 6669 SOL 2981

HETATM 6670 HW2 SOL 2987 80.690 49.050 54.890 1.00 0.00 H

HETATM 6671 OW SOL 2988 9.960 45.130 49.450 1.00 0.00 O

HETATM 6672 HW1 SOL 2988 10.770 45.460 50.000 1.00 0.00 H

HETATM 6673 HW2 SOL 2988 9.230 44.870 50.100 1.00 0.00 H

TER 6674 SOL 2988

HETATM 6675 OW SOL 2990 16.850 43.170 49.610 1.00 0.00 O

HETATM 6676 HW1 SOL 2990 15.940 42.930 49.970 1.00 0.00 H

HETATM 6677 HW2 SOL 2990 16.860 42.960 48.630 1.00 0.00 H

HETATM 6678 OW SOL 2991 80.300 55.370 51.190 1.00 0.00 O

HETATM 6679 HW1 SOL 2991 80.060 54.560 51.750 1.00 0.00 H

HETATM 6680 HW2 SOL 2991 80.610 56.120 51.800 1.00 0.00 H

HETATM 6681 OW SOL 2992 80.950 46.800 38.540 1.00 0.00 O

HETATM 6682 HW1 SOL 2992 81.190 47.570 37.920 1.00 0.00 H

HETATM 6683 HW2 SOL 2992 81.040 47.160 39.500 1.00 0.00 H

TER 6684 SOL 2992

HETATM 6685 OW SOL 2994 75.000 43.740 43.020 1.00 0.00 O

HETATM 6686 HW1 SOL 2994 75.200 42.780 42.820 1.00 0.00 H

HETATM 6687 HW2 SOL 2994 75.640 44.050 43.730 1.00 0.00 H

HETATM 6688 OW SOL 2995 13.200 45.730 40.230 1.00 0.00 O

HETATM 6689 OW SOL 2996 53.590 48.420 78.660 1.00 0.00 O

HETATM 6690 HW1 SOL 2996 54.410 48.000 78.220 1.00 0.00 H

HETATM 6691 HW2 SOL 2996 53.920 48.930 79.490 1.00 0.00 H

TER 6692 SOL 2996

HETATM 6693 OW SOL 2999 17.120 38.650 42.250 1.00 0.00 O

HETATM 6694 HW1 SOL 2999 17.920 38.630 41.610 1.00 0.00 H

HETATM 6695 HW2 SOL 2999 16.950 37.680 42.530 1.00 0.00 H

TER 6696 SOL 2999

HETATM 6697 OW SOL 3001 12.750 46.470 43.770 1.00 0.00 O

HETATM 6698 HW1 SOL 3001 13.660 46.090 44.000 1.00 0.00 H

HETATM 6699 HW2 SOL 3001 13.010 47.330 43.260 1.00 0.00 H

HETATM 6700 OW SOL 3002 77.760 39.440 55.590 1.00 0.00 O

HETATM 6701 HW1 SOL 3002 78.350 40.260 55.380 1.00 0.00 H

TER 6702 SOL 3002

HETATM 6703 OW SOL 3004 16.960 54.790 51.310 1.00 0.00 O

HETATM 6704 HW1 SOL 3004 17.020 54.650 50.330 1.00 0.00 H

HETATM 6705 HW2 SOL 3004 16.350 55.600 51.490 1.00 0.00 H

HETATM 6706 OW SOL 3005 53.800 54.170 80.120 1.00 0.00 O

HETATM 6707 HW1 SOL 3005 53.420 55.080 79.880 1.00 0.00 H

HETATM 6708 HW2 SOL 3005 53.290 53.800 80.930 1.00 0.00 H

HETATM 6709 HW2 SOL 3005 79.770 44.890 40.490 1.00 0.00 H

TER 6710 SOL 3005

HETATM 6711 OW SOL 3007 8.300 47.250 50.990 1.00 0.00 O

HETATM 6712 HW1 SOL 3007 8.790 47.210 50.100 1.00 0.00 H

HETATM 6713 HW2 SOL 3007 8.260 48.260 51.220 1.00 0.00 H

TER 6714 SOL 3007

HETATM 6715 OW SOL 3013 75.680 56.160 53.090 1.00 0.00 O

HETATM 6716 HW1 SOL 3013 75.790 55.850 54.070 1.00 0.00 H

HETATM 6717 HW2 SOL 3013 75.270 55.360 52.630 1.00 0.00 H

TER 6718 SOL 3013

HETATM 6719 OW SOL 3017 51.110 48.660 80.040 1.00 0.00 O

HETATM 6720 OW SOL 3017 80.010 39.100 46.190 1.00 0.00 O

HETATM 6721 HW1 SOL 3017 51.870 48.460 79.380 1.00 0.00 H

HETATM 6722 HW1 SOL 3017 79.530 39.360 47.040 1.00 0.00 H

HETATM 6723 HW2 SOL 3017 50.310 48.970 79.510 1.00 0.00 H

HETATM 6724 HW2 SOL 3017 80.210 39.970 45.720 1.00 0.00 H

TER 6725 SOL 3017

HETATM 6726 OW SOL 3019 47.360 51.640 79.200 1.00 0.00 O

HETATM 6727 HW1 SOL 3019 47.040 52.450 78.670 1.00 0.00 H

HETATM 6728 OW SOL 3020 17.760 41.010 53.810 1.00 0.00 O

HETATM 6729 HW1 SOL 3020 17.950 40.770 52.850 1.00 0.00 H

HETATM 6730 HW2 SOL 3020 16.930 40.450 54.070 1.00 0.00 H

HETATM 6731 HW1 SOL 3021 84.950 54.680 39.900 1.00 0.00 H

TER 6732 SOL 3021

HETATM 6733 OW SOL 3023 14.120 55.750 54.550 1.00 0.00 O

HETATM 6734 OW SOL 3023 79.160 54.690 40.420 1.00 0.00 O

HETATM 6735 HW1 SOL 3023 13.170 55.630 54.160 1.00 0.00 H

HETATM 6736 HW1 SOL 3023 79.670 55.240 41.120 1.00 0.00 H

HETATM 6737 HW2 SOL 3023 14.690 55.090 54.030 1.00 0.00 H

HETATM 6738 HW2 SOL 3023 79.840 54.310 39.760 1.00 0.00 H

TER 6739 SOL 3023

HETATM 6740 OW SOL 3025 84.130 54.260 46.240 1.00 0.00 O

HETATM 6741 HW2 SOL 3025 83.570 53.790 45.510 1.00 0.00 H

TER 6742 SOL 3025

HETATM 6743 OW SOL 3027 41.800 54.670 77.560 1.00 0.00 O

HETATM 6744 HW1 SOL 3027 42.200 54.890 78.470 1.00 0.00 H

HETATM 6745 HW2 SOL 3027 42.030 53.700 77.340 1.00 0.00 H

TER 6746 SOL 3027

HETATM 6747 OW SOL 3029 83.280 52.470 49.300 1.00 0.00 O

HETATM 6748 HW1 SOL 3029 83.140 52.180 48.330 1.00 0.00 H

HETATM 6749 HW2 SOL 3030 10.940 37.340 48.670 1.00 0.00 H

HETATM 6750 OW SOL 3031 82.960 51.410 46.740 1.00 0.00 O

HETATM 6751 HW1 SOL 3031 83.900 51.090 46.470 1.00 0.00 H

HETATM 6752 HW2 SOL 3031 82.450 51.300 45.860 1.00 0.00 H

TER 6753 SOL 3031

HETATM 6754 HW1 SOL 3033 79.420 38.090 43.120 1.00 0.00 H

HETATM 6755 OW SOL 3034 12.700 41.730 44.010 1.00 0.00 O

HETATM 6756 HW1 SOL 3034 12.250 42.640 44.140 1.00 0.00 H

HETATM 6757 HW2 SOL 3034 12.890 41.370 44.950 1.00 0.00 H

HETATM 6758 OW SOL 3035 77.350 37.560 38.990 1.00 0.00 O

TER 6759 SOL 3035

HETATM 6760 OW SOL 3037 15.200 51.460 62.730 1.00 0.00 O

HETATM 6761 HW1 SOL 3037 15.230 52.010 63.580 1.00 0.00 H

HETATM 6762 HW2 SOL 3037 14.790 52.070 62.010 1.00 0.00 H

TER 6763 SOL 3037

HETATM 6764 OW SOL 3045 49.890 55.200 80.730 1.00 0.00 O

HETATM 6765 HW1 SOL 3045 50.120 55.650 79.850 1.00 0.00 H

HETATM 6766 OW SOL 3046 16.080 41.690 63.290 1.00 0.00 O

HETATM 6767 HW1 SOL 3046 15.270 41.810 62.680 1.00 0.00 H

HETATM 6768 HW2 SOL 3046 15.920 42.300 64.080 1.00 0.00 H

TER 6769 SOL 3046

HETATM 6770 HW2 SOL 3053 55.290 44.860 77.640 1.00 0.00 H

HETATM 6771 OW SOL 3054 42.050 51.630 76.350 1.00 0.00 O

HETATM 6772 HW1 SOL 3054 41.360 51.050 76.840 1.00 0.00 H

HETATM 6773 HW2 SOL 3054 41.780 51.430 75.380 1.00 0.00 H

HETATM 6774 OW SOL 3055 54.210 50.180 80.660 1.00 0.00 O

HETATM 6775 HW1 SOL 3055 55.140 49.920 81.020 1.00 0.00 H

HETATM 6776 HW2 SOL 3055 53.690 50.580 81.440 1.00 0.00 H

HETATM 6777 OW SOL 3056 51.190 43.400 75.450 1.00 0.00 O

HETATM 6778 OW SOL 3056 81.470 47.460 41.150 1.00 0.00 O

HETATM 6779 HW1 SOL 3056 50.530 42.900 76.050 1.00 0.00 H

HETATM 6780 HW1 SOL 3056 81.700 48.270 41.700 1.00 0.00 H

HETATM 6781 HW2 SOL 3056 51.920 42.730 75.240 1.00 0.00 H

TER 6782 SOL 3056

HETATM 6783 OW SOL 3058 14.670 51.440 65.550 1.00 0.00 O

HETATM 6784 HW2 SOL 3058 14.250 50.640 65.130 1.00 0.00 H

HETATM 6785 OW SOL 3059 86.870 50.750 41.140 1.00 0.00 O

HETATM 6786 HW2 SOL 3059 85.950 50.820 41.570 1.00 0.00 H

TER 6787 SOL 3059

HETATM 6788 OW SOL 3061 15.600 53.730 58.920 1.00 0.00 O

HETATM 6789 HW1 SOL 3061 16.460 54.240 58.800 1.00 0.00 H

HETATM 6790 HW2 SOL 3061 15.240 53.660 57.940 1.00 0.00 H

HETATM 6791 OW SOL 3062 78.850 44.810 53.450 1.00 0.00 O

HETATM 6792 HW1 SOL 3062 79.410 45.210 52.690 1.00 0.00 H

HETATM 6793 HW2 SOL 3062 78.730 43.840 53.220 1.00 0.00 H

TER 6794 SOL 3062

HETATM 6795 OW SOL 3065 79.530 37.550 54.740 1.00 0.00 O

HETATM 6796 HW1 SOL 3065 79.090 37.060 53.950 1.00 0.00 H

HETATM 6797 HW2 SOL 3065 78.840 38.290 54.940 1.00 0.00 H

TER 6798 SOL 3065

HETATM 6799 OW SOL 3068 81.700 43.450 47.740 1.00 0.00 O

HETATM 6800 HW1 SOL 3068 82.500 43.090 47.200 1.00 0.00 H

HETATM 6801 HW2 SOL 3068 81.680 44.450 47.600 1.00 0.00 H

TER 6802 SOL 3068

HETATM 6803 OW SOL 3071 85.150 44.620 46.890 1.00 0.00 O

HETATM 6804 HW1 SOL 3071 84.880 44.940 47.830 1.00 0.00 H

HETATM 6805 HW2 SOL 3071 84.640 43.740 46.760 1.00 0.00 H

HETATM 6806 HW2 SOL 3072 53.610 44.010 78.720 1.00 0.00 H

HETATM 6807 OW SOL 3073 14.800 44.790 63.140 1.00 0.00 O

HETATM 6808 HW1 SOL 3073 14.360 44.080 63.730 1.00 0.00 H

HETATM 6809 HW2 SOL 3073 15.660 45.000 63.610 1.00 0.00 H

HETATM 6810 HW2 SOL 3074 78.150 45.830 54.570 1.00 0.00 H

HETATM 6811 HW2 SOL 3075 11.430 54.450 57.370 1.00 0.00 H

TER 6812 SOL 3075

HETATM 6813 HW1 SOL 3077 85.250 49.750 45.230 1.00 0.00 H

HETATM 6814 OW SOL 3078 78.030 41.970 47.480 1.00 0.00 O

HETATM 6815 HW1 SOL 3078 77.470 41.160 47.220 1.00 0.00 H

HETATM 6816 HW2 SOL 3078 78.650 42.210 46.700 1.00 0.00 H

TER 6817 SOL 3078

HETATM 6818 OW SOL 3082 84.250 45.200 49.470 1.00 0.00 O

HETATM 6819 HW2 SOL 3082 83.400 44.710 49.700 1.00 0.00 H

HETATM 6820 OW SOL 3083 76.470 42.430 54.820 1.00 0.00 O

HETATM 6821 HW1 SOL 3083 77.160 41.920 54.300 1.00 0.00 H

HETATM 6822 HW2 SOL 3083 76.070 43.080 54.130 1.00 0.00 H

TER 6823 SOL 3083

HETATM 6824 OW SOL 3087 81.620 41.760 49.900 1.00 0.00 O

HETATM 6825 HW1 SOL 3087 80.850 41.110 49.990 1.00 0.00 H

HETATM 6826 HW2 SOL 3087 81.430 42.360 49.090 1.00 0.00 H

TER 6827 SOL 3087

HETATM 6828 OW SOL 3089 79.000 42.180 39.140 1.00 0.00 O

HETATM 6829 HW1 SOL 3089 79.490 43.050 39.380 1.00 0.00 H

HETATM 6830 HW2 SOL 3089 78.040 42.450 38.870 1.00 0.00 H

TER 6831 SOL 3089

HETATM 6832 OW SOL 3094 81.330 54.080 54.830 1.00 0.00 O

HETATM 6833 HW1 SOL 3094 80.750 54.890 54.630 1.00 0.00 H

HETATM 6834 HW2 SOL 3094 81.880 53.880 54.010 1.00 0.00 H

TER 6835 SOL 3094

HETATM 6836 OW SOL 3098 14.730 43.200 65.540 1.00 0.00 O

HETATM 6837 OW SOL 3098 36.890 50.440 76.400 1.00 0.00 O

HETATM 6838 HW1 SOL 3098 35.970 50.260 76.740 1.00 0.00 H

HETATM 6839 HW2 SOL 3098 15.320 43.190 66.390 1.00 0.00 H

HETATM 6840 HW2 SOL 3098 37.400 49.580 76.620 1.00 0.00 H

TER 6841 SOL 3098

HETATM 6842 HW2 SOL 3100 81.970 50.480 51.090 1.00 0.00 H

HETATM 6843 OW SOL 3101 80.810 53.390 38.530 1.00 0.00 O

HETATM 6844 HW2 SOL 3101 80.670 52.570 39.100 1.00 0.00 H

HETATM 6845 HW1 SOL 3102 18.440 49.210 71.340 1.00 0.00 H

HETATM 6846 HW2 SOL 3102 17.610 50.250 72.250 1.00 0.00 H

TER 6847 SOL 3102

HETATM 6848 HW1 SOL 3104 16.030 53.910 71.440 1.00 0.00 H

HETATM 6849 HW2 SOL 3104 16.830 53.350 72.730 1.00 0.00 H

HETATM 6850 HW1 SOL 3105 83.330 47.100 50.160 1.00 0.00 H

HETATM 6851 OW SOL 3106 16.080 48.090 66.710 1.00 0.00 O

HETATM 6852 HW1 SOL 3106 16.330 48.820 67.400 1.00 0.00 H

HETATM 6853 HW2 SOL 3106 15.080 48.010 66.850 1.00 0.00 H

TER 6854 SOL 3106

HETATM 6855 OW SOL 3109 85.090 48.260 44.470 1.00 0.00 O

HETATM 6856 HW1 SOL 3109 84.180 47.860 44.350 1.00 0.00 H

TER 6857 SOL 3109

HETATM 6858 OW SOL 3112 75.570 47.990 54.980 1.00 0.00 O

HETATM 6859 HW1 SOL 3112 74.710 47.470 55.230 1.00 0.00 H

HETATM 6860 HW2 SOL 3112 76.320 47.290 54.920 1.00 0.00 H

HETATM 6861 OW SOL 3113 50.090 45.750 77.430 1.00 0.00 O

HETATM 6862 OW SOL 3113 81.420 39.080 52.090 1.00 0.00 O

HETATM 6863 HW1 SOL 3113 49.430 45.480 78.140 1.00 0.00 H

HETATM 6864 HW1 SOL 3113 82.360 38.790 51.810 1.00 0.00 H

HETATM 6865 HW2 SOL 3113 50.380 44.900 76.970 1.00 0.00 H

HETATM 6866 HW2 SOL 3113 81.510 39.550 52.990 1.00 0.00 H

HETATM 6867 OW SOL 3114 12.560 50.840 59.470 1.00 0.00 O

HETATM 6868 HW1 SOL 3114 13.120 51.550 59.940 1.00 0.00 H

HETATM 6869 HW2 SOL 3114 11.620 50.960 59.870 1.00 0.00 H

HETATM 6870 HW1 SOL 3115 85.440 50.090 38.510 1.00 0.00 H

HETATM 6871 OW SOL 3116 80.740 45.570 51.640 1.00 0.00 O

HETATM 6872 HW1 SOL 3116 81.350 46.350 51.360 1.00 0.00 H

HETATM 6873 HW2 SOL 3116 16.150 44.070 59.870 1.00 0.00 H

HETATM 6874 HW2 SOL 3116 81.360 44.760 51.680 1.00 0.00 H

TER 6875 SOL 3116

HETATM 6876 OW SOL 3122 80.340 44.590 43.960 1.00 0.00 O

HETATM 6877 HW1 SOL 3122 80.840 45.340 43.510 1.00 0.00 H

HETATM 6878 HW2 SOL 3122 79.640 44.260 43.310 1.00 0.00 H

HETATM 6879 OW SOL 3123 13.550 45.850 66.050 1.00 0.00 O

HETATM 6880 HW1 SOL 3123 12.590 45.700 65.780 1.00 0.00 H

TER 6881 SOL 3123

HETATM 6882 OW SOL 3125 82.850 51.330 37.700 1.00 0.00 O

HETATM 6883 HW1 SOL 3125 83.150 51.420 38.670 1.00 0.00 H

TER 6884 SOL 3125

HETATM 6885 OW SOL 3129 76.870 50.150 55.820 1.00 0.00 O

HETATM 6886 HW1 SOL 3129 76.360 49.320 55.460 1.00 0.00 H

HETATM 6887 HW2 SOL 3129 76.440 50.330 56.720 1.00 0.00 H

TER 6888 SOL 3129

HETATM 6889 OW SOL 3131 78.840 41.660 42.590 1.00 0.00 O

HETATM 6890 HW2 SOL 3131 78.360 41.100 43.260 1.00 0.00 H

HETATM 6891 OW SOL 3132 12.960 45.510 56.330 1.00 0.00 O

HETATM 6892 HW1 SOL 3132 13.300 44.530 56.400 1.00 0.00 H

HETATM 6893 HW2 SOL 3132 13.360 45.840 55.460 1.00 0.00 H

HETATM 6894 OW SOL 3133 83.010 53.100 44.090 1.00 0.00 O

HETATM 6895 HW1 SOL 3133 83.320 53.470 43.210 1.00 0.00 H

HETATM 6896 HW2 SOL 3133 82.680 52.160 43.810 1.00 0.00 H

HETATM 6897 OW SOL 3134 39.970 50.410 77.600 1.00 0.00 O

HETATM 6898 HW1 SOL 3134 39.310 50.680 76.860 1.00 0.00 H

TER 6899 SOL 3134

HETATM 6900 OW SOL 3139 83.400 54.230 40.430 1.00 0.00 O

HETATM 6901 HW1 SOL 3139 83.060 53.280 40.610 1.00 0.00 H

HETATM 6902 HW2 SOL 3139 83.120 54.800 41.230 1.00 0.00 H

HETATM 6903 HW2 SOL 3140 76.760 43.220 56.290 1.00 0.00 H

HETATM 6904 OW SOL 3141 16.410 43.260 67.660 1.00 0.00 O

HETATM 6905 HW2 SOL 3141 17.370 43.000 67.380 1.00 0.00 H

TER 6906 SOL 3141

HETATM 6907 OW SOL 3147 12.870 45.860 59.350 1.00 0.00 O

HETATM 6908 HW2 SOL 3147 13.050 45.850 58.350 1.00 0.00 H

TER 6909 SOL 3147

HETATM 6910 HW2 SOL 3150 46.100 51.450 74.350 1.00 0.00 H

TER 6911 SOL 3150

HETATM 6912 OW SOL 3152 13.920 52.890 60.770 1.00 0.00 O

HETATM 6913 HW1 SOL 3152 14.580 53.230 60.040 1.00 0.00 H

HETATM 6914 OW SOL 3153 12.970 46.530 62.120 1.00 0.00 O

HETATM 6915 HW1 SOL 3153 13.610 45.800 62.470 1.00 0.00 H

HETATM 6916 HW2 SOL 3153 12.890 46.360 61.110 1.00 0.00 H

HETATM 6917 OW SOL 3154 48.540 47.260 75.100 1.00 0.00 O

HETATM 6918 HW1 SOL 3154 47.860 47.640 74.430 1.00 0.00 H

HETATM 6919 HW2 SOL 3154 48.420 46.260 75.080 1.00 0.00 H

HETATM 6920 HW1 SOL 3155 55.020 52.630 82.410 1.00 0.00 H

HETATM 6921 OW SOL 3156 16.220 54.430 69.800 1.00 0.00 O

HETATM 6922 HW1 SOL 3156 16.880 54.400 69.040 1.00 0.00 H

TER 6923 SOL 3156

HETATM 6924 HW2 SOL 3158 76.070 51.340 61.970 1.00 0.00 H

HETATM 6925 OW SOL 3159 11.850 53.240 56.200 1.00 0.00 O

HETATM 6926 HW1 SOL 3159 11.210 52.640 56.670 1.00 0.00 H

HETATM 6927 HW2 SOL 3159 11.380 53.420 55.300 1.00 0.00 H

TER 6928 SOL 3159

HETATM 6929 HW1 SOL 3161 9.920 46.830 58.090 1.00 0.00 H

HETATM 6930 OW SOL 3162 14.780 53.610 56.460 1.00 0.00 O

HETATM 6931 HW1 SOL 3162 13.870 53.730 56.040 1.00 0.00 H

HETATM 6932 HW2 SOL 3162 15.470 53.660 55.720 1.00 0.00 H

HETATM 6933 HW1 SOL 3163 16.910 49.990 69.220 1.00 0.00 H

HETATM 6934 HW2 SOL 3164 11.110 47.670 62.470 1.00 0.00 H

TER 6935 SOL 3164

HETATM 6936 OW SOL 3171 14.760 54.990 65.590 1.00 0.00 O

HETATM 6937 HW1 SOL 3171 15.040 55.810 65.050 1.00 0.00 H

HETATM 6938 HW2 SOL 3171 15.610 54.580 65.960 1.00 0.00 H

HETATM 6939 OW SOL 3172 45.910 53.640 78.010 1.00 0.00 O

HETATM 6940 HW1 SOL 3172 45.160 52.970 77.900 1.00 0.00 H

HETATM 6941 HW2 SOL 3172 45.620 54.390 78.630 1.00 0.00 H

TER 6942 SOL 3172

HETATM 6943 OW SOL 3174 16.720 46.010 68.410 1.00 0.00 O

HETATM 6944 HW1 SOL 3174 16.610 45.080 68.000 1.00 0.00 H

HETATM 6945 HW2 SOL 3174 16.610 46.710 67.670 1.00 0.00 H

TER 6946 SOL 3174

HETATM 6947 OW SOL 3179 8.630 50.910 56.210 1.00 0.00 O

TER 6948 SOL 3179

HETATM 6949 OW SOL 3183 11.090 47.400 56.870 1.00 0.00 O

HETATM 6950 HW1 SOL 3183 10.580 47.680 56.010 1.00 0.00 H

HETATM 6951 HW2 SOL 3183 11.670 46.590 56.600 1.00 0.00 H

TER 6952 SOL 3183

HETATM 6953 OW SOL 3187 16.860 52.100 67.100 1.00 0.00 O

HETATM 6954 HW1 SOL 3187 16.090 51.850 66.470 1.00 0.00 H

HETATM 6955 HW2 SOL 3187 17.040 51.260 67.640 1.00 0.00 H

TER 6956 SOL 3187

HETATM 6957 OW SOL 3230 79.310 51.410 57.410 1.00 0.00 O

HETATM 6958 HW1 SOL 3230 78.600 51.100 58.080 1.00 0.00 H

HETATM 6959 HW2 SOL 3230 78.900 51.130 56.530 1.00 0.00 H

TER 6960 SOL 3230

HETATM 6961 OW SOL 3294 75.360 49.820 65.990 1.00 0.00 O

HETATM 6962 HW1 SOL 3294 75.200 50.010 67.000 1.00 0.00 H

HETATM 6963 HW2 SOL 3294 74.670 49.130 65.720 1.00 0.00 H

TER 6964 SOL 3294

HETATM 6965 OW SOL 3340 75.140 49.540 62.500 1.00 0.00 O

HETATM 6966 HW1 SOL 3340 75.490 49.080 63.330 1.00 0.00 H

HETATM 6967 HW2 SOL 3340 75.790 49.260 61.770 1.00 0.00 H

TER 6968 SOL 3340

HETATM 6969 OW SOL 3440 41.690 59.930 33.590 1.00 0.00 O

HETATM 6970 HW1 SOL 3440 42.490 59.320 33.440 1.00 0.00 H

HETATM 6971 HW2 SOL 3440 40.860 59.340 33.630 1.00 0.00 H

HETATM 6972 OW SOL 3441 53.350 60.380 25.720 1.00 0.00 O

HETATM 6973 HW1 SOL 3441 52.540 60.410 25.100 1.00 0.00 H

HETATM 6974 HW2 SOL 3441 53.240 61.260 26.220 1.00 0.00 H

TER 6975 SOL 3441

HETATM 6976 OW SOL 3443 48.420 69.430 35.350 1.00 0.00 O

HETATM 6977 HW1 SOL 3443 49.090 69.510 36.120 1.00 0.00 H

HETATM 6978 HW2 SOL 3443 48.000 68.520 35.450 1.00 0.00 H

TER 6979 SOL 3443

HETATM 6980 OW SOL 3451 53.900 64.880 24.880 1.00 0.00 O

HETATM 6981 HW1 SOL 3451 54.890 64.780 24.980 1.00 0.00 H

TER 6982 SOL 3451

HETATM 6983 OW SOL 3453 38.320 63.300 34.420 1.00 0.00 O

HETATM 6984 HW1 SOL 3453 39.070 63.730 34.960 1.00 0.00 H

HETATM 6985 HW2 SOL 3453 37.630 64.030 34.300 1.00 0.00 H

TER 6986 SOL 3453

HETATM 6987 OW SOL 3455 50.080 68.250 32.850 1.00 0.00 O

HETATM 6988 HW1 SOL 3455 50.430 67.610 32.140 1.00 0.00 H

HETATM 6989 HW2 SOL 3455 50.730 68.140 33.650 1.00 0.00 H

HETATM 6990 OW SOL 3456 45.330 55.850 23.310 1.00 0.00 O

HETATM 6991 HW1 SOL 3456 45.840 56.710 23.530 1.00 0.00 H

HETATM 6992 HW2 SOL 3456 44.380 56.010 23.660 1.00 0.00 H

TER 6993 SOL 3456

HETATM 6994 HW1 SOL 3460 41.010 58.840 36.580 1.00 0.00 H

TER 6995 SOL 3460

HETATM 6996 HW1 SOL 3472 49.200 70.520 34.310 1.00 0.00 H

TER 6997 SOL 3472

HETATM 6998 OW SOL 3475 41.900 68.250 35.960 1.00 0.00 O

HETATM 6999 HW1 SOL 3475 41.690 68.540 35.000 1.00 0.00 H

TER 7000 SOL 3475

HETATM 7001 OW SOL 3477 39.180 58.830 33.900 1.00 0.00 O

HETATM 7002 HW2 SOL 3477 39.280 58.220 34.700 1.00 0.00 H

TER 7003 SOL 3477

HETATM 7004 OW SOL 3480 51.530 57.140 22.830 1.00 0.00 O

HETATM 7005 HW1 SOL 3480 51.230 56.830 21.900 1.00 0.00 H

HETATM 7006 HW2 SOL 3480 51.780 56.260 23.290 1.00 0.00 H

TER 7007 SOL 3480

HETATM 7008 OW SOL 3487 46.520 61.050 27.590 1.00 0.00 O

HETATM 7009 HW1 SOL 3487 46.270 60.840 26.600 1.00 0.00 H

HETATM 7010 HW2 SOL 3487 46.710 60.150 28.000 1.00 0.00 H

HETATM 7011 OW SOL 3488 42.620 56.410 23.550 1.00 0.00 O

HETATM 7012 HW1 SOL 3488 41.710 56.510 24.030 1.00 0.00 H

HETATM 7013 HW2 SOL 3488 42.690 57.260 22.990 1.00 0.00 H

TER 7014 SOL 3488

HETATM 7015 OW SOL 3500 54.030 68.600 24.950 1.00 0.00 O

HETATM 7016 HW1 SOL 3500 55.030 68.660 25.130 1.00 0.00 H

TER 7017 SOL 3500

HETATM 7018 OW SOL 3503 54.700 68.240 34.580 1.00 0.00 O

HETATM 7019 HW1 SOL 3503 55.510 67.780 34.160 1.00 0.00 H

HETATM 7020 HW2 SOL 3503 54.970 69.220 34.730 1.00 0.00 H

HETATM 7021 OW SOL 3504 47.790 69.410 31.920 1.00 0.00 O

HETATM 7022 HW1 SOL 3504 46.990 69.080 32.480 1.00 0.00 H

HETATM 7023 HW2 SOL 3504 48.620 69.000 32.380 1.00 0.00 H

TER 7024 SOL 3504

HETATM 7025 HW1 SOL 3506 47.920 68.290 30.380 1.00 0.00 H

TER 7026 SOL 3506

HETATM 7027 OW SOL 3508 41.500 63.990 31.200 1.00 0.00 O

HETATM 7028 HW1 SOL 3508 42.250 64.010 31.860 1.00 0.00 H

TER 7029 SOL 3508

HETATM 7030 HW2 SOL 3511 43.420 61.540 27.940 1.00 0.00 H

TER 7031 SOL 3511

HETATM 7032 OW SOL 3516 42.410 56.600 35.270 1.00 0.00 O

HETATM 7033 HW1 SOL 3516 42.480 55.590 35.470 1.00 0.00 H

HETATM 7034 HW2 SOL 3516 41.620 56.950 35.810 1.00 0.00 H

TER 7035 SOL 3516

HETATM 7036 OW SOL 3520 44.550 62.170 29.180 1.00 0.00 O

HETATM 7037 HW1 SOL 3520 45.380 61.930 28.610 1.00 0.00 H

HETATM 7038 HW2 SOL 3520 44.240 63.110 28.930 1.00 0.00 H

HETATM 7039 OW SOL 3521 40.190 56.860 24.580 1.00 0.00 O

HETATM 7040 HW2 SOL 3521 40.200 56.230 25.400 1.00 0.00 H

HETATM 7041 HW1 SOL 3522 53.040 58.880 22.740 1.00 0.00 H

HETATM 7042 OW SOL 3523 46.940 71.850 35.520 1.00 0.00 O

HETATM 7043 HW1 SOL 3523 46.890 72.080 34.520 1.00 0.00 H

HETATM 7044 HW2 SOL 3523 47.340 70.920 35.600 1.00 0.00 H

TER 7045 SOL 3523

HETATM 7046 OW SOL 3529 40.360 61.210 28.640 1.00 0.00 O

HETATM 7047 HW1 SOL 3529 40.250 60.210 28.570 1.00 0.00 H

HETATM 7048 HW2 SOL 3529 41.240 61.360 28.100 1.00 0.00 H

HETATM 7049 OW SOL 3530 42.080 60.070 36.680 1.00 0.00 O

HETATM 7050 HW1 SOL 3530 42.540 60.360 37.530 1.00 0.00 H

HETATM 7051 HW2 SOL 3530 42.650 60.350 35.900 1.00 0.00 H

TER 7052 SOL 3530

HETATM 7053 HW1 SOL 3534 38.580 61.360 29.250 1.00 0.00 H

HETATM 7054 OW SOL 3535 39.020 61.080 35.880 1.00 0.00 O

HETATM 7055 HW1 SOL 3535 39.760 60.680 35.310 1.00 0.00 H

HETATM 7056 HW2 SOL 3535 38.640 61.860 35.330 1.00 0.00 H

TER 7057 SOL 3535

HETATM 7058 OW SOL 3538 43.420 60.460 30.970 1.00 0.00 O

HETATM 7059 HW1 SOL 3538 43.640 59.560 30.550 1.00 0.00 H

HETATM 7060 HW2 SOL 3538 43.890 61.150 30.380 1.00 0.00 H

TER 7061 SOL 3538

HETATM 7062 HW1 SOL 3541 38.680 66.840 34.200 1.00 0.00 H

HETATM 7063 OW SOL 3542 50.650 66.640 30.690 1.00 0.00 O

HETATM 7064 HW1 SOL 3542 50.550 65.620 30.760 1.00 0.00 H

HETATM 7065 HW2 SOL 3542 49.770 67.010 30.290 1.00 0.00 H

TER 7066 SOL 3542

HETATM 7067 OW SOL 3547 44.020 72.700 36.200 1.00 0.00 O

HETATM 7068 HW2 SOL 3547 44.730 72.440 35.530 1.00 0.00 H

TER 7069 SOL 3547

HETATM 7070 OW SOL 3552 45.480 68.570 33.080 1.00 0.00 O

HETATM 7071 HW1 SOL 3552 45.300 68.850 34.050 1.00 0.00 H

HETATM 7072 HW2 SOL 3552 44.650 68.820 32.560 1.00 0.00 H

TER 7073 SOL 3552

HETATM 7074 OW SOL 3555 39.780 59.180 25.870 1.00 0.00 O

HETATM 7075 HW2 SOL 3555 39.600 58.870 26.810 1.00 0.00 H

TER 7076 SOL 3555

HETATM 7077 OW SOL 3563 38.150 66.390 35.830 1.00 0.00 O

HETATM 7078 HW1 SOL 3563 37.310 66.080 36.360 1.00 0.00 H

HETATM 7079 HW2 SOL 3563 38.910 65.820 36.190 1.00 0.00 H

HETATM 7080 OW SOL 3564 44.140 58.050 33.790 1.00 0.00 O

HETATM 7081 HW1 SOL 3564 44.470 57.260 33.270 1.00 0.00 H

HETATM 7082 HW2 SOL 3564 43.520 57.620 34.500 1.00 0.00 H

TER 7083 SOL 3564

HETATM 7084 OW SOL 3569 51.930 67.990 34.820 1.00 0.00 O

HETATM 7085 HW1 SOL 3569 51.720 68.320 35.750 1.00 0.00 H

HETATM 7086 HW2 SOL 3569 52.950 68.010 34.740 1.00 0.00 H

TER 7087 SOL 3569

HETATM 7088 HW1 SOL 3571 50.190 58.350 23.040 1.00 0.00 H

TER 7089 SOL 3571

HETATM 7090 HW2 SOL 3578 55.240 71.020 27.950 1.00 0.00 H

TER 7091 SOL 3578

HETATM 7092 OW SOL 3582 54.150 70.940 29.410 1.00 0.00 O

HETATM 7093 HW1 SOL 3582 53.290 70.610 28.940 1.00 0.00 H

HETATM 7094 HW2 SOL 3582 54.110 70.520 30.330 1.00 0.00 H

HETATM 7095 HW2 SOL 3583 40.910 61.140 30.650 1.00 0.00 H

TER 7096 SOL 3583

HETATM 7097 OW SOL 3588 50.200 60.410 25.530 1.00 0.00 O

HETATM 7098 HW2 SOL 3588 49.920 60.050 26.430 1.00 0.00 H

TER 7099 SOL 3588

HETATM 7100 OW SOL 3591 39.220 62.180 38.460 1.00 0.00 O

HETATM 7101 HW1 SOL 3591 38.360 62.090 38.980 1.00 0.00 H

HETATM 7102 HW2 SOL 3591 39.100 61.700 37.570 1.00 0.00 H

TER 7103 SOL 3591

HETATM 7104 OW SOL 3593 54.850 61.960 46.060 1.00 0.00 O

HETATM 7105 HW1 SOL 3593 55.320 62.840 45.820 1.00 0.00 H

HETATM 7106 HW2 SOL 3593 55.300 61.680 46.940 1.00 0.00 H

HETATM 7107 OW SOL 3594 44.890 67.500 47.800 1.00 0.00 O

HETATM 7108 HW1 SOL 3594 44.160 67.930 48.380 1.00 0.00 H

HETATM 7109 HW2 SOL 3594 45.230 68.270 47.240 1.00 0.00 H

TER 7110 SOL 3594

HETATM 7111 OW SOL 3597 53.270 60.260 44.740 1.00 0.00 O

HETATM 7112 HW1 SOL 3597 52.570 60.850 44.280 1.00 0.00 H

HETATM 7113 HW2 SOL 3597 53.830 60.940 45.280 1.00 0.00 H

TER 7114 SOL 3597

HETATM 7115 HW2 SOL 3599 48.120 68.720 53.920 1.00 0.00 H

TER 7116 SOL 3599

HETATM 7117 OW SOL 3601 39.660 66.460 46.290 1.00 0.00 O

HETATM 7118 HW1 SOL 3601 40.380 66.070 46.900 1.00 0.00 H

HETATM 7119 HW2 SOL 3601 39.200 67.220 46.780 1.00 0.00 H

TER 7120 SOL 3601

HETATM 7121 HW1 SOL 3603 37.490 61.890 42.900 1.00 0.00 H

HETATM 7122 HW2 SOL 3603 38.330 63.130 43.530 1.00 0.00 H

TER 7123 SOL 3603

HETATM 7124 OW SOL 3606 43.370 69.790 44.690 1.00 0.00 O

HETATM 7125 HW1 SOL 3606 42.820 68.920 44.760 1.00 0.00 H

HETATM 7126 HW2 SOL 3606 43.200 70.080 43.710 1.00 0.00 H

HETATM 7127 OW SOL 3607 39.180 65.670 49.720 1.00 0.00 O

HETATM 7128 HW1 SOL 3607 40.050 65.270 49.380 1.00 0.00 H

HETATM 7129 HW2 SOL 3607 38.460 65.280 49.140 1.00 0.00 H

HETATM 7130 OW SOL 3608 49.150 73.930 53.310 1.00 0.00 O

HETATM 7131 OW SOL 3609 53.020 73.020 40.370 1.00 0.00 O

HETATM 7132 HW2 SOL 3609 52.890 72.520 39.500 1.00 0.00 H

HETATM 7133 OW SOL 3610 38.170 64.370 44.750 1.00 0.00 O

HETATM 7134 HW1 SOL 3610 39.150 64.310 44.980 1.00 0.00 H

HETATM 7135 HW2 SOL 3610 37.830 65.160 45.310 1.00 0.00 H

TER 7136 SOL 3610

HETATM 7137 OW SOL 3612 54.730 61.780 55.210 1.00 0.00 O

HETATM 7138 HW1 SOL 3612 53.950 62.100 55.760 1.00 0.00 H

HETATM 7139 HW2 SOL 3612 54.490 60.930 54.740 1.00 0.00 H

TER 7140 SOL 3612

HETATM 7141 OW SOL 3614 55.300 66.930 45.990 1.00 0.00 O

HETATM 7142 HW1 SOL 3614 54.840 66.140 45.510 1.00 0.00 H

HETATM 7143 HW2 SOL 3614 54.670 66.970 46.810 1.00 0.00 H

TER 7144 SOL 3614

HETATM 7145 OW SOL 3616 40.270 65.580 39.680 1.00 0.00 O

HETATM 7146 HW1 SOL 3616 41.170 65.880 39.340 1.00 0.00 H

HETATM 7147 HW2 SOL 3616 40.290 64.570 39.750 1.00 0.00 H

HETATM 7148 OW SOL 3617 41.940 68.590 54.700 1.00 0.00 O

HETATM 7149 HW1 SOL 3617 41.660 68.690 53.700 1.00 0.00 H

HETATM 7150 HW2 SOL 3617 41.030 68.590 55.170 1.00 0.00 H

HETATM 7151 OW SOL 3618 53.680 69.700 37.810 1.00 0.00 O

HETATM 7152 HW1 SOL 3618 53.320 70.660 37.830 1.00 0.00 H

HETATM 7153 HW2 SOL 3618 53.100 69.180 38.450 1.00 0.00 H

TER 7154 SOL 3618

HETATM 7155 OW SOL 3620 51.390 56.740 41.690 1.00 0.00 O

HETATM 7156 HW1 SOL 3620 51.080 56.710 40.710 1.00 0.00 H

HETATM 7157 HW2 SOL 3620 52.030 57.510 41.770 1.00 0.00 H

TER 7158 SOL 3620

HETATM 7159 HW1 SOL 3622 47.220 72.220 46.570 1.00 0.00 H

HETATM 7160 OW SOL 3623 40.800 63.030 41.580 1.00 0.00 O

HETATM 7161 HW1 SOL 3623 41.420 62.600 42.240 1.00 0.00 H

HETATM 7162 HW2 SOL 3623 39.870 62.790 41.930 1.00 0.00 H

TER 7163 SOL 3623

HETATM 7164 OW SOL 3626 42.950 56.430 42.390 1.00 0.00 O

HETATM 7165 HW1 SOL 3626 41.950 56.540 42.540 1.00 0.00 H

HETATM 7166 HW2 SOL 3626 43.220 57.240 41.870 1.00 0.00 H

TER 7167 SOL 3626

HETATM 7168 OW SOL 3628 50.140 74.270 43.700 1.00 0.00 O

HETATM 7169 HW1 SOL 3628 50.420 74.660 42.810 1.00 0.00 H

HETATM 7170 HW2 SOL 3628 49.190 74.550 43.880 1.00 0.00 H

HETATM 7171 OW SOL 3629 56.310 64.150 40.940 1.00 0.00 O

HETATM 7172 HW1 SOL 3629 56.390 63.560 41.760 1.00 0.00 H

HETATM 7173 HW2 SOL 3629 55.490 63.810 40.450 1.00 0.00 H

HETATM 7174 OW SOL 3630 42.040 70.230 38.720 1.00 0.00 O

HETATM 7175 HW1 SOL 3630 41.310 69.810 39.300 1.00 0.00 H

HETATM 7176 HW2 SOL 3630 42.390 69.470 38.160 1.00 0.00 H

HETATM 7177 OW SOL 3631 54.800 68.290 53.130 1.00 0.00 O

HETATM 7178 HW1 SOL 3631 55.580 67.810 52.690 1.00 0.00 H

HETATM 7179 OW SOL 3632 47.830 69.600 50.690 1.00 0.00 O

HETATM 7180 HW1 SOL 3632 46.930 69.220 51.050 1.00 0.00 H

HETATM 7181 HW2 SOL 3632 48.530 68.890 50.930 1.00 0.00 H

TER 7182 SOL 3632

HETATM 7183 OW SOL 3634 53.550 66.950 48.050 1.00 0.00 O

HETATM 7184 HW1 SOL 3634 53.480 67.580 48.870 1.00 0.00 H

HETATM 7185 HW2 SOL 3634 52.830 66.260 48.220 1.00 0.00 H

TER 7186 SOL 3634

HETATM 7187 OW SOL 3636 40.750 66.970 42.170 1.00 0.00 O

HETATM 7188 HW1 SOL 3636 41.150 67.640 41.530 1.00 0.00 H

HETATM 7189 HW2 SOL 3636 40.640 66.130 41.610 1.00 0.00 H

HETATM 7190 OW SOL 3637 49.910 70.670 48.820 1.00 0.00 O

HETATM 7191 HW1 SOL 3637 50.610 70.490 49.530 1.00 0.00 H

HETATM 7192 HW2 SOL 3637 49.280 69.880 48.800 1.00 0.00 H

TER 7193 SOL 3637

HETATM 7194 OW SOL 3640 47.020 71.500 54.400 1.00 0.00 O

TER 7195 SOL 3640

HETATM 7196 OW SOL 3642 47.860 69.440 45.790 1.00 0.00 O

HETATM 7197 HW1 SOL 3642 47.730 68.720 45.070 1.00 0.00 H

HETATM 7198 HW2 SOL 3642 48.770 69.840 45.600 1.00 0.00 H

TER 7199 SOL 3642

HETATM 7200 OW SOL 3648 38.900 67.340 51.690 1.00 0.00 O

HETATM 7201 HW1 SOL 3648 39.020 66.690 52.440 1.00 0.00 H

HETATM 7202 HW2 SOL 3648 39.040 66.730 50.840 1.00 0.00 H

TER 7203 SOL 3648

HETATM 7204 OW SOL 3650 49.670 73.320 48.440 1.00 0.00 O

HETATM 7205 HW1 SOL 3650 50.510 73.440 47.870 1.00 0.00 H

HETATM 7206 HW2 SOL 3650 49.650 72.300 48.590 1.00 0.00 H

TER 7207 SOL 3650

HETATM 7208 OW SOL 3653 54.610 57.130 42.460 1.00 0.00 O

HETATM 7209 HW1 SOL 3653 55.160 56.670 41.760 1.00 0.00 H

HETATM 7210 HW2 SOL 3653 54.020 56.440 42.880 1.00 0.00 H

TER 7211 SOL 3653

HETATM 7212 OW SOL 3655 45.410 68.840 51.550 1.00 0.00 O

HETATM 7213 HW2 SOL 3655 44.670 68.690 50.870 1.00 0.00 H

HETATM 7214 OW SOL 3656 43.600 72.380 38.830 1.00 0.00 O

HETATM 7215 HW1 SOL 3656 43.880 72.470 37.830 1.00 0.00 H

HETATM 7216 HW2 SOL 3656 43.050 71.490 38.800 1.00 0.00 H

TER 7217 SOL 3656

HETATM 7218 OW SOL 3658 55.540 57.350 46.990 1.00 0.00 O

HETATM 7219 HW1 SOL 3658 55.890 56.860 46.190 1.00 0.00 H

HETATM 7220 HW2 SOL 3658 54.620 57.680 46.640 1.00 0.00 H

HETATM 7221 OW SOL 3659 52.430 72.100 37.740 1.00 0.00 O

HETATM 7222 HW1 SOL 3659 51.810 72.890 37.880 1.00 0.00 H

HETATM 7223 OW SOL 3660 40.900 69.030 52.300 1.00 0.00 O

HETATM 7224 HW2 SOL 3660 40.170 68.400 51.960 1.00 0.00 H

HETATM 7225 OW SOL 3661 53.250 68.570 50.170 1.00 0.00 O

HETATM 7226 HW1 SOL 3661 54.040 68.540 50.800 1.00 0.00 H

HETATM 7227 OW SOL 3662 43.170 70.730 42.180 1.00 0.00 O

HETATM 7228 HW1 SOL 3662 43.640 69.920 41.740 1.00 0.00 H

HETATM 7229 HW2 SOL 3662 42.600 71.140 41.460 1.00 0.00 H

TER 7230 SOL 3662

HETATM 7231 OW SOL 3664 38.000 59.160 37.440 1.00 0.00 O

HETATM 7232 HW1 SOL 3664 36.990 59.150 37.470 1.00 0.00 H

HETATM 7233 HW2 SOL 3664 38.240 59.980 36.860 1.00 0.00 H

HETATM 7234 OW SOL 3665 43.540 64.890 51.340 1.00 0.00 O

HETATM 7235 HW1 SOL 3665 44.110 65.590 50.910 1.00 0.00 H

HETATM 7236 HW2 SOL 3665 43.960 64.000 51.160 1.00 0.00 H

HETATM 7237 OW SOL 3666 52.110 73.440 47.070 1.00 0.00 O

HETATM 7238 HW1 SOL 3666 52.090 73.930 46.180 1.00 0.00 H

HETATM 7239 HW2 SOL 3666 53.040 73.550 47.440 1.00 0.00 H

HETATM 7240 OW SOL 3667 52.020 68.340 53.770 1.00 0.00 O

HETATM 7241 HW1 SOL 3667 51.920 67.730 54.550 1.00 0.00 H

HETATM 7242 HW2 SOL 3667 52.980 68.280 53.450 1.00 0.00 H

TER 7243 SOL 3667

HETATM 7244 OW SOL 3670 51.320 74.420 54.770 1.00 0.00 O

HETATM 7245 HW1 SOL 3670 50.490 74.270 54.170 1.00 0.00 H

HETATM 7246 HW2 SOL 3670 52.020 73.770 54.380 1.00 0.00 H

HETATM 7247 OW SOL 3671 55.740 71.490 45.840 1.00 0.00 O

HETATM 7248 HW1 SOL 3671 55.170 71.480 44.980 1.00 0.00 H

HETATM 7249 HW2 SOL 3671 55.940 70.530 46.070 1.00 0.00 H

HETATM 7250 OW SOL 3672 39.570 68.860 55.830 1.00 0.00 O

HETATM 7251 HW1 SOL 3672 38.620 68.730 55.500 1.00 0.00 H

HETATM 7252 HW2 SOL 3672 39.550 69.010 56.830 1.00 0.00 H

TER 7253 SOL 3672

HETATM 7254 OW SOL 3675 55.610 58.030 49.660 1.00 0.00 O

HETATM 7255 HW1 SOL 3675 55.750 57.150 50.110 1.00 0.00 H

HETATM 7256 HW2 SOL 3675 55.420 57.850 48.670 1.00 0.00 H

HETATM 7257 OW SOL 3676 54.320 70.920 48.740 1.00 0.00 O

HETATM 7258 HW1 SOL 3676 53.570 70.620 48.130 1.00 0.00 H

HETATM 7259 HW2 SOL 3676 54.700 70.060 49.140 1.00 0.00 H

HETATM 7260 HW1 SOL 3677 45.570 72.020 43.100 1.00 0.00 H

HETATM 7261 HW2 SOL 3677 44.700 71.090 44.080 1.00 0.00 H

HETATM 7262 OW SOL 3678 51.850 57.950 48.430 1.00 0.00 O

HETATM 7263 HW1 SOL 3678 52.090 57.230 49.090 1.00 0.00 H

HETATM 7264 HW2 SOL 3678 50.960 58.300 48.780 1.00 0.00 H

HETATM 7265 OW SOL 3679 47.580 56.180 48.580 1.00 0.00 O

HETATM 7266 HW1 SOL 3679 46.950 56.210 47.770 1.00 0.00 H

HETATM 7267 HW2 SOL 3679 48.060 55.300 48.600 1.00 0.00 H

TER 7268 SOL 3679

HETATM 7269 OW SOL 3681 50.620 56.460 39.110 1.00 0.00 O

HETATM 7270 HW1 SOL 3681 51.210 56.140 38.360 1.00 0.00 H

HETATM 7271 HW2 SOL 3681 49.860 55.800 39.180 1.00 0.00 H

HETATM 7272 OW SOL 3682 53.150 57.890 46.030 1.00 0.00 O

TER 7273 SOL 3682

HETATM 7274 OW SOL 3684 45.620 72.660 40.640 1.00 0.00 O

HETATM 7275 HW1 SOL 3684 44.820 72.500 40.010 1.00 0.00 H

HETATM 7276 HW2 SOL 3684 45.390 73.380 41.320 1.00 0.00 H

HETATM 7277 OW SOL 3685 44.040 67.440 74.280 1.00 0.00 O

HETATM 7278 HW1 SOL 3685 44.870 67.890 73.930 1.00 0.00 H

TER 7279 SOL 3685

HETATM 7280 OW SOL 3687 51.970 71.030 69.290 1.00 0.00 O

HETATM 7281 HW2 SOL 3687 51.180 71.140 69.940 1.00 0.00 H

TER 7282 SOL 3687

HETATM 7283 OW SOL 3689 39.550 70.250 61.230 1.00 0.00 O

TER 7284 SOL 3689

HETATM 7285 OW SOL 3692 55.640 73.450 59.190 1.00 0.00 O

HETATM 7286 HW1 SOL 3692 55.340 73.560 60.160 1.00 0.00 H

HETATM 7287 HW2 SOL 3692 56.000 74.380 58.900 1.00 0.00 H

TER 7288 SOL 3692

HETATM 7289 OW SOL 3694 38.550 63.290 71.700 1.00 0.00 O

HETATM 7290 HW1 SOL 3694 39.080 63.500 72.510 1.00 0.00 H

HETATM 7291 HW2 SOL 3694 37.850 64.020 71.590 1.00 0.00 H

HETATM 7292 OW SOL 3695 43.560 69.790 63.110 1.00 0.00 O

HETATM 7293 HW1 SOL 3695 43.030 68.930 63.320 1.00 0.00 H

HETATM 7294 HW2 SOL 3695 43.290 70.050 62.150 1.00 0.00 H

HETATM 7295 OW SOL 3696 39.300 65.570 68.620 1.00 0.00 O

HETATM 7296 HW1 SOL 3696 40.080 65.310 68.040 1.00 0.00 H

HETATM 7297 HW2 SOL 3696 38.520 64.990 68.340 1.00 0.00 H

HETATM 7298 HW2 SOL 3697 49.100 73.190 71.530 1.00 0.00 H

HETATM 7299 OW SOL 3698 54.110 72.860 61.440 1.00 0.00 O

HETATM 7300 HW1 SOL 3698 53.480 72.820 60.640 1.00 0.00 H

HETATM 7301 HW2 SOL 3698 53.580 72.560 62.240 1.00 0.00 H

HETATM 7302 OW SOL 3699 52.820 73.220 58.800 1.00 0.00 O

HETATM 7303 HW1 SOL 3699 53.840 73.330 58.740 1.00 0.00 H

HETATM 7304 HW2 SOL 3699 52.610 72.680 57.940 1.00 0.00 H

HETATM 7305 OW SOL 3700 38.090 64.180 64.090 1.00 0.00 O

HETATM 7306 HW1 SOL 3700 39.040 64.430 63.820 1.00 0.00 H

HETATM 7307 HW2 SOL 3700 37.620 65.030 64.390 1.00 0.00 H

TER 7308 SOL 3700

HETATM 7309 OW SOL 3703 49.750 71.580 70.830 1.00 0.00 O

HETATM 7310 HW1 SOL 3703 49.660 71.200 71.770 1.00 0.00 H

HETATM 7311 HW2 SOL 3703 48.890 71.250 70.400 1.00 0.00 H

TER 7312 SOL 3703

HETATM 7313 OW SOL 3707 41.920 67.540 63.330 1.00 0.00 O

HETATM 7314 HW1 SOL 3707 40.950 67.560 63.700 1.00 0.00 H

HETATM 7315 HW2 SOL 3707 41.860 67.500 62.310 1.00 0.00 H

HETATM 7316 OW SOL 3708 42.380 69.840 57.560 1.00 0.00 O

HETATM 7317 HW1 SOL 3708 41.450 69.750 57.990 1.00 0.00 H

HETATM 7318 HW2 SOL 3708 42.280 69.460 56.620 1.00 0.00 H

HETATM 7319 HW2 SOL 3709 54.120 71.810 72.430 1.00 0.00 H

TER 7320 SOL 3709

HETATM 7321 OW SOL 3713 47.440 71.540 72.640 1.00 0.00 O

HETATM 7322 HW1 SOL 3713 47.080 72.040 71.820 1.00 0.00 H

HETATM 7323 HW2 SOL 3713 46.860 70.710 72.710 1.00 0.00 H

HETATM 7324 OW SOL 3714 55.690 71.260 71.860 1.00 0.00 O

HETATM 7325 HW1 SOL 3714 56.110 71.380 70.950 1.00 0.00 H

HETATM 7326 HW2 SOL 3714 56.460 71.160 72.520 1.00 0.00 H

HETATM 7327 OW SOL 3715 40.790 64.850 73.880 1.00 0.00 O

HETATM 7328 HW1 SOL 3715 41.490 65.150 74.570 1.00 0.00 H

HETATM 7329 HW2 SOL 3715 41.290 64.780 73.010 1.00 0.00 H

HETATM 7330 HW2 SOL 3716 37.560 68.410 57.400 1.00 0.00 H

TER 7331 SOL 3716

HETATM 7332 HW1 SOL 3718 42.840 72.290 65.130 1.00 0.00 H

TER 7333 SOL 3718

HETATM 7334 OW SOL 3720 46.640 72.760 70.370 1.00 0.00 O

HETATM 7335 HW1 SOL 3720 46.160 72.300 69.610 1.00 0.00 H

HETATM 7336 HW2 SOL 3720 46.430 73.760 70.270 1.00 0.00 H

TER 7337 SOL 3720

HETATM 7338 OW SOL 3722 54.950 73.430 67.410 1.00 0.00 O

HETATM 7339 HW1 SOL 3722 55.630 73.680 66.690 1.00 0.00 H

HETATM 7340 HW2 SOL 3722 54.670 72.460 67.180 1.00 0.00 H

HETATM 7341 OW SOL 3723 49.650 73.120 67.140 1.00 0.00 O

HETATM 7342 HW1 SOL 3723 50.590 73.460 67.010 1.00 0.00 H

HETATM 7343 HW2 SOL 3723 49.670 72.500 67.940 1.00 0.00 H

TER 7344 SOL 3723

HETATM 7345 OW SOL 3727 45.200 68.260 70.300 1.00 0.00 O

HETATM 7346 HW1 SOL 3727 44.910 68.900 71.040 1.00 0.00 H

HETATM 7347 HW2 SOL 3727 44.850 68.630 69.440 1.00 0.00 H

HETATM 7348 HW2 SOL 3728 43.320 71.240 57.400 1.00 0.00 H

HETATM 7349 HW1 SOL 3729 54.120 73.280 68.930 1.00 0.00 H

HETATM 7350 OW SOL 3730 52.080 72.110 56.420 1.00 0.00 O

HETATM 7351 HW1 SOL 3730 51.330 71.680 55.900 1.00 0.00 H

HETATM 7352 HW2 SOL 3730 52.800 72.190 55.690 1.00 0.00 H

TER 7353 SOL 3730

HETATM 7354 OW SOL 3732 53.880 69.150 68.920 1.00 0.00 O

HETATM 7355 HW1 SOL 3732 54.060 68.650 69.780 1.00 0.00 H

HETATM 7356 HW2 SOL 3732 53.060 69.750 69.110 1.00 0.00 H

HETATM 7357 OW SOL 3733 43.100 70.680 60.520 1.00 0.00 O

HETATM 7358 HW1 SOL 3733 43.350 70.390 59.580 1.00 0.00 H

HETATM 7359 HW2 SOL 3733 42.460 71.450 60.380 1.00 0.00 H

HETATM 7360 OW SOL 3734 39.930 69.500 58.690 1.00 0.00 O

HETATM 7361 HW1 SOL 3734 38.930 69.580 58.480 1.00 0.00 H

HETATM 7362 HW2 SOL 3734 39.940 69.750 59.700 1.00 0.00 H

TER 7363 SOL 3734

HETATM 7364 OW SOL 3736 48.960 69.040 56.740 1.00 0.00 O

HETATM 7365 HW1 SOL 3736 47.950 69.280 56.710 1.00 0.00 H

HETATM 7366 HW2 SOL 3736 49.210 68.950 57.700 1.00 0.00 H

TER 7367 SOL 3736

HETATM 7368 OW SOL 3738 46.370 69.880 56.550 1.00 0.00 O

HETATM 7369 HW1 SOL 3738 45.370 69.750 56.610 1.00 0.00 H

HETATM 7370 HW2 SOL 3738 46.490 70.460 55.700 1.00 0.00 H

HETATM 7371 OW SOL 3739 43.660 73.740 65.240 1.00 0.00 O

HETATM 7372 HW1 SOL 3739 43.570 74.740 65.070 1.00 0.00 H

HETATM 7373 HW2 SOL 3739 44.640 73.530 65.440 1.00 0.00 H

TER 7374 SOL 3739

HETATM 7375 OW SOL 3741 47.550 65.690 56.710 1.00 0.00 O

HETATM 7376 HW1 SOL 3741 46.980 65.550 55.880 1.00 0.00 H
[truncated: 213,005 more chars]
